# Photochemical C4-Selective C–H Amination of Quinolines via *N*‑Shift of Heteroaryl Azides

**DOI:** 10.1021/acs.orglett.6c01213

**Published:** 2026-04-29

**Authors:** Alessandro Dimasi, Arianna Montoli, Giovanni Macetti, Leonardo Lo Presti, Daniele Passarella, Valerio Fasano

**Affiliations:** Department of Chemistry, 9304 Università degli Studi di Milano, Via Camillo Golgi 19, 20133, Milano, Italy

## Abstract

Access to 3,4-diaminoazines is limited by regioselectivity challenges in azine functionalization. We report a photochemical strategy enabling the direct synthesis of 3,4-diaminoazines through C3-to-C4 nitrogen migration (*N*-shift). Blue LED irradiation of 3-azidoquinolines at room temperature allows C4-selective C–H amination while concurrently installing diverse amines at the C3*-*position. In contrast, *N-*shifts in 3-azidopyridines preferentially form diazepines, reflecting divergent reactivity governed by *N*-heterocycle electronics, as supported by computational analysis.

## Graphical abstract

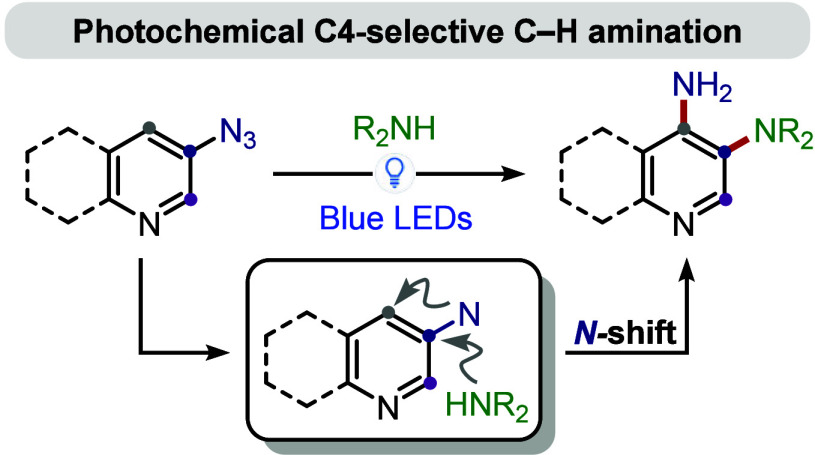

*N-*Heterocycles are a key component of medicinal chemistry, appearing in a substantial proportion of drug molecules.^1,2^ Within this chemical space, 3,4-diaminoazines are particularly valuable: the simplest example, 3,4-diaminopyridine, is marketed as the neuromuscular enhancer amifampridine, while more substituted analogues are key precursors to imidazoquinolines found in antiviral and antiallergic agents (Scheme 1A).^3,4^ Despite their importance, efficient access to richly substituted 3,4-diaminoazines remains challenging mainly because of site-selectivity issues in azine functionalization (C2 vs C4). Conventional approaches rely on stepwise installation of the two amino groups: typically, a harsh nitration and subsequent reduction to install the C3*-*NH_2_ group, and an S_N_Ar reaction for the C4-amine.^5–7^ These multistep routes require forcing conditions and extensive functional group manipulations, which becomes problematic for incorporating elaborated amines. Alternatively, direct functionalization of diamino heterocycles suffers from low regioselectivity,^8,9^ whereas selective C–H amination at distal positions, despite recent advances^10–24^ (Scheme 1B), remains rare and is often incompatible with adjacent amino substituents, underscoring the need for milder, more direct methods. Motivated by our interest in selective heteroaromatic manipulation,^25–27^ we questioned whether skeletal editing could offer a more streamlined entry to 3,4-diaminoazines. In this context, the use of nitrenes has emerged as an innovative approach to remodel aromatic scaffolds, as recently demonstrated by the conversion of phenyl azides to aminopyridines or *ortho*-aminophenols (Scheme 1C).^28–35^ In these reports, visible-light irradiation of phenyl azides generates a singlet nitrene, which undergoes a dearomative–rearomative sequence (via an azepine intermediate),^36,37^ furnishing, depending on reaction conditions, the desired product. Despite their success, these reactions have been limited to phenyl azides, with unsymmetrical ones often suffering poor regioselectivity.^33^ We envisaged that this approach would allow the remodelling of heterocyclic azides into 3,4-diaminoazines if (i) nitrene insertion (*N*-shift) occurs selectively at the C4-position, (ii) the nitrene nitrogen is not retained in the aromatic ring (as observed for aminopyridines),^32^ and (iii) rearomatization is still favored over diazepine formation, despite pyridines being less aromatic than benzenes.

**Scheme 1:**
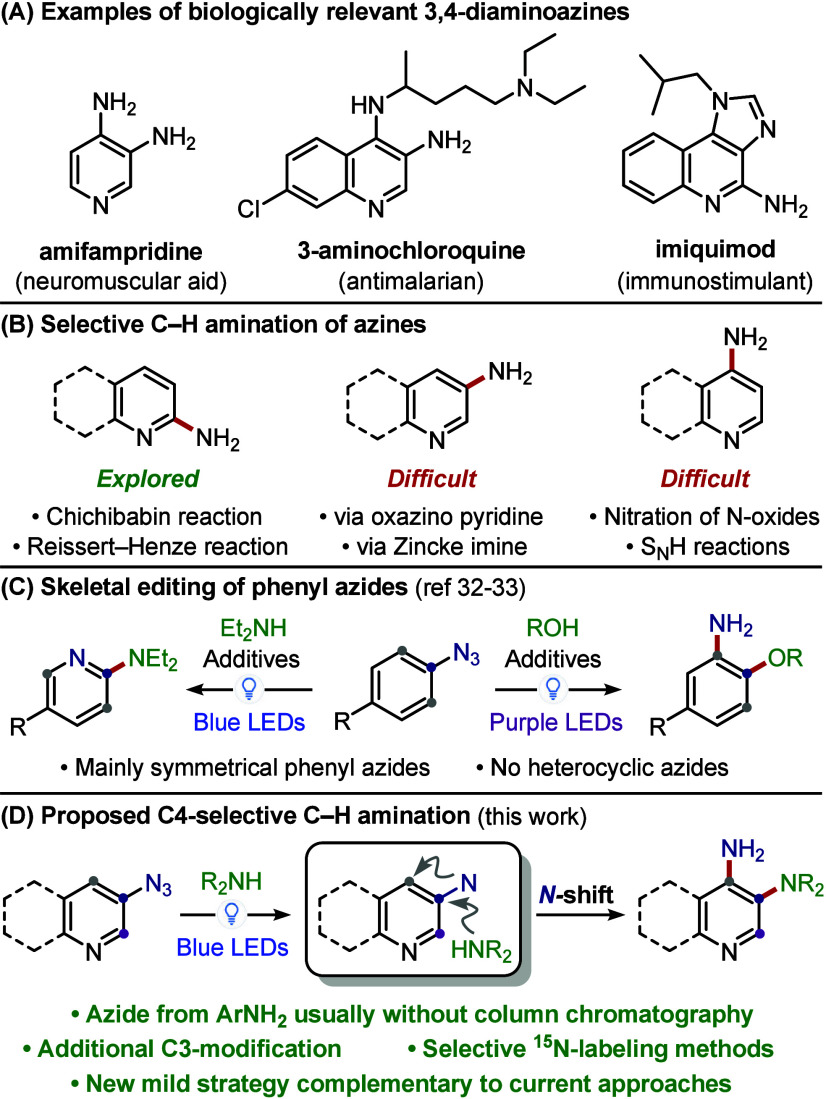
(A) Examples of Bioactive 3,4-Diaminoazines; (B) Strategies for the Selective C–H Amination of Azines; (C) Visible-Light Remodelling of Phenyl Azides;^32,33^ (D) C4-Selective C–H Amination of Heteroaryl Azides

Herein, we present a fundamentally new strategy for selective *N*-heterocycle functionalization that enables direct access to 3,4-diaminoazines via formal C3*-*to-C4 amino migration. This process replaces the migrating group with a second amine, including NH_2_, providing a concise and modular route to these pharmaceutical scaffolds.

In 1982, Suschitzky and co-workers examined the photolysis of azidoquinolines in primary and secondary aliphatic amines.^38^ UV irradiation with a mercury lamp gave complex mixtures of diazepines, *ortho*-diamines, and aminoquinolines with product distributions governed by both the position of the azide and the nature of the amine solvent. Given the poor functional group tolerance of UV irradiation (only a single example with 3-azidoquinoline) and the impractical requirement for amines as reaction media (especially expensive or solid ones), we endeavored to develop a milder and more practical method. Our investigation, therefore, began with the blue-light irradiation (50 W Kessil Lamp, in analogy with previous work)^32,33^ of commercially available 3-azidoquinoline **Az-1** in the presence of diethylamine as the nucleophile, since primary amines were ineffective in Suschitzky’s study and ammonia was unsuitable for optimization due to its volatility (Scheme 2).

**Scheme 2:**
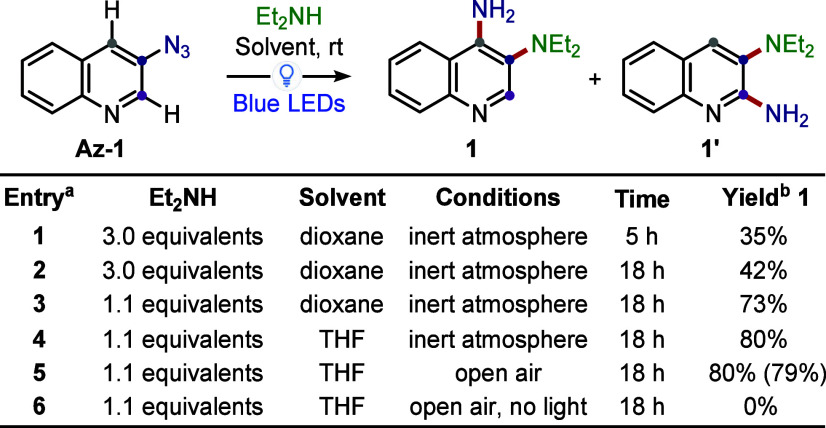
Optimization of the C4-Selective C–H Amination of **Az-1** Using a 50 W Kessil Lamp

a All reactions were performed on 0.2 mmol scale (1 equiv) of **Az-1**.
b NMR yield using CH_2_Br_2_ as internal standard. In brackets, isolated yield.

In an initial attempt, the reaction was performed with 3 equiv of the amine under oxygen-free conditions achieved by freeze–pump–thaw cycles. After 5 h (entry 1), the reaction delivered **1** in 35% yield, along with 22% of 3-aminoquinoline (via a triplet nitrene pathway).^39^ The presence of **1** was identified in the ^1^H NMR spectrum in CDCl_3_ by a characteristic downfield signal at 8.48 ppm that can be attributed to the C2-proton and a broad singlet at 5.71 ppm corresponding to the free amino group (connectivity further confirmed by bidimensional NMR analysis of the isolated product). Optimization of the model reaction involved extending the reaction time, reducing the equivalents of Et_2_NH, and switching the solvent from dioxane to THF (entries 2–4). Conducting the reaction under air to suppress the undesired triplet pathway had no significant impact on the outcome (entry 5), although it did enhance the operational simplicity of the protocol. The formation of **1** is noteworthy, as phenyl azides typically require an external activator (e.g., excess of TFAA) to promote rearomatization of the azepine, as reported by Leonori, Ruffoni, and co-workers.^33^ In contrast, in our case, no significant formation of 1,4-benzodiazepines was detected, at least not for quinoline substrates (see Scheme 5 for further details). Importantly, compound **1′**, as well as any related 1,3-benzodiazepine precursors, was not observed in the crude reaction mixture. This outcome highlights a remarkable C4-selectivity nitrene insertion into the pyridine framework, despite disubstituted phenyl azides usually furnishing mixtures of regioisomers. With a successful protocol in hand, the substrate scope of the reaction was explored (Scheme 3). First, ammonia was used as the nucleophile so that a free amino group would be restored in the C3*-*position. While 5.5 equiv of NH_3_ (7 M in THF) gave reduced reactivity (44% yield), a large excess (35 equiv) of this volatile amine gave product **2** in 66% yield. A one-pot protocol from 3-aminoquinoline **A1** (Sandmeyer reaction, followed by *N*-shift) resulted in an overall yield of 64%, showing the possibility for a direct C–H amination. Primary amines (unsuccessful in Suschitzky’s protocol)^38^ were also compatible with our method, with the alkyl substituent being a primary carbon (**3**), a secondary carbon (**4**–**5**) or a tertiary carbon (**6**–**7**). It has to be noted that *tert*-butylamine and amantadine are of particular interest since they cannot be introduced via reductive amination on 3-aminoquinoline, whereas their steric hindrance negatively impacts S_N_Ar reactions or Buchwald–Hartwig aminations on 3-bromoquinoline. Other relevant primary amines are benzylamines and allylamines since the deprotection of the corresponding products (**8**–**11**) provides a free amino group alternative to the use of ammonia. Moving then to secondary amines, acyclic and cyclic amines (four- to seven-membered cycles) could be incorporated into the desired product (**13**–**17**). Morpholine and thiomorpholine, abundant motifs in many synthetic drugs, could also be incorporated at the C3*-*position (compounds **18**–**19**), with the 3,4-diaminoquinoline structure of **18** further confirmed by X-ray diffraction (XRD) analysis. Similarly, unsymmetrical piperazine could also be used (compounds **20**–**22**), including a Boc-protected one, which allows for further derivatization upon deprotection. Piperidines bearing an ester, a primary alcohol, or tetrahydroisoquinoline were also employed as substrates, further expanding the tolerance of the reaction to these functional groups (compounds **23**–**25**). It has to be noted that many of the resulting 3,4-diaminoquinolines had never previously been synthesized, despite the simplicity of their structures; moreover, in the case of compound **16**, a longer synthetic route was needed using established methodologies.^40^ On the other hand, unsuccessful substrates include anilines (even activated ones such as trimethoxyaniline) and bulky secondary amines like *i*Pr_2_NH and *i*Bu_2_NH, highlighting the negative effect of the reduced nucleophilicity of the amine. The C4-selective C–H amination was also tested on other substrates, such as functionalized quinolines and pyridines. In the first case, the desired diaminoquinolines **26**–**31** were isolated, including 7-chloro-4-aminoquinoline **29** (the regioisomer of chloroquine-based antimalarial agents). Unexpectedly, C2- or C3*-*substituted 3-azidopyridines yielded, after prolonged irradiation (48 h), predominantly 1,3- or 1,4-diazepines, such as **32** or **33**. These examples are still successful outcomes of the *N*-shift mechanism, yet with opposite regioselectivity and without rearomatization of the seven-membered rings.^41^ Finally, the protocol is also amenable to the incorporation at the C3*-*position of complex amines, as shown for compounds **34**, **35**, **36**, deriving from trimetazidine, ephedrine and desloratadine, respectively. Further applications of photocatalyzed C–H amination are reported in Scheme 4. First, a selective isotopic labeling method was developed, useful for the synthesis of labeled drugs for binding studies via ^15^N NMR analysis.^42,43^ This required only the use of commercially available ^15^NH_3_ as the nucleophile and to access A-1*. Impressively, both ^15^N-labeled 3,4-diaminoquinolines 2a* and 2b* could be accessed, highlighting selective and predictable isotopic labeling. Second, the C–H amination was tested on **Az-1** (34 or 1000 mg) using *i*BuNH_2_ as the nucleophile, observing minimal erosion of the yield (81% vs 69%, respectively). This showed the potential scalability of the method, furnishing product **38** as a strategic intermediate for further derivatizations. Indeed, **38** was converted into imidazoquinoline **39**, a regioisomer of imiquimod in which the *i*Bu group is installed on the C3*-*nitrogen.

**Scheme 3:**
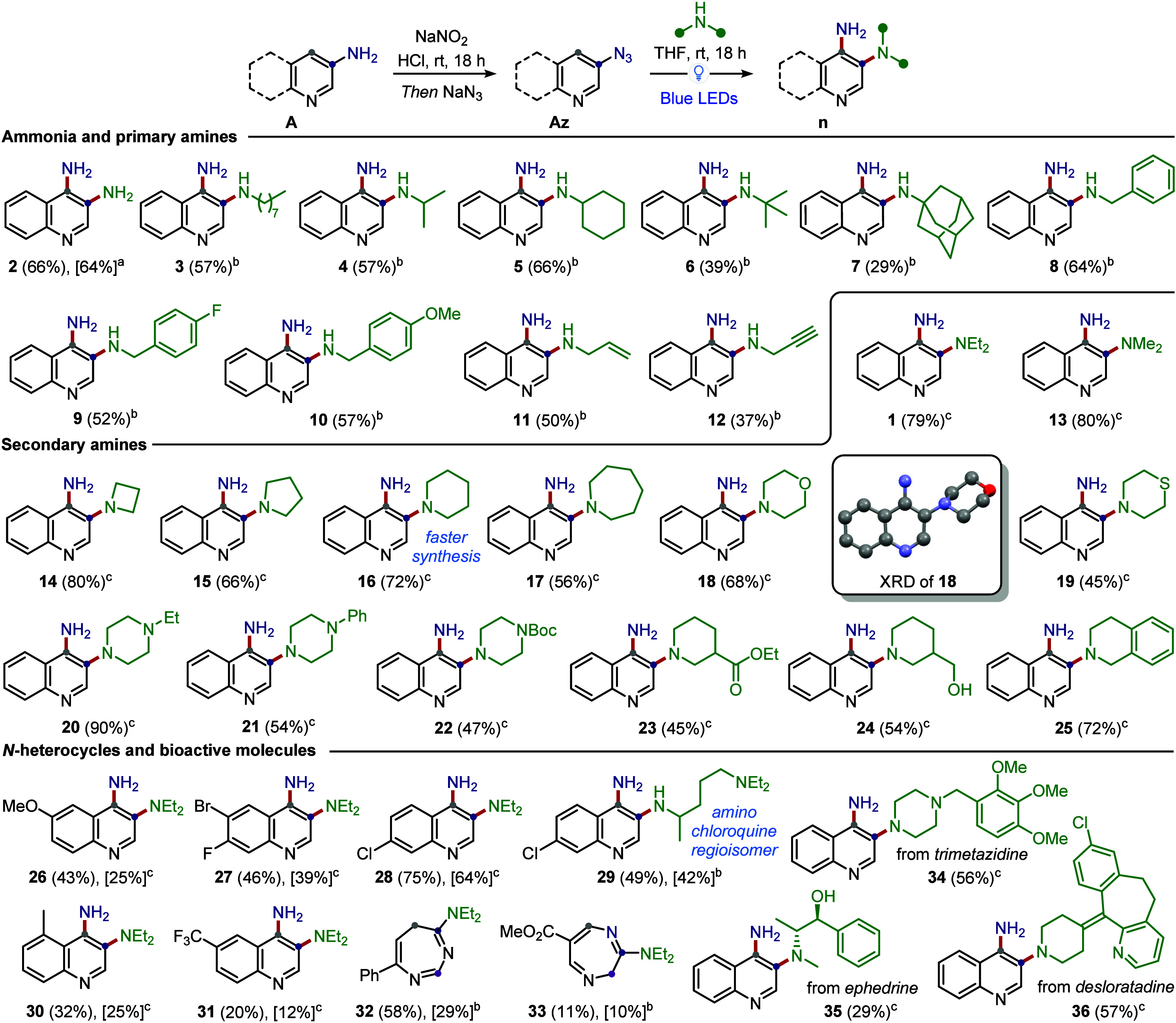
Substrate Scope of the Photocatalyzed C4-Selective C–H Amination of Azines, with Isolated Yield from **Az** (in Brackets) or from **A** over 2 Steps (in Square Brackets); Reaction Performed Dissolving the Heteroaryl Azide (1.0 equiv) and the Amine in THF (0.2 M), Followed by Irradiation at 456 nm with a 50 W Kessil Lamp

a 35.0 equiv of amine.
b 2.2 equiv of amine.
c 1.1 equiv of amine. In the molecular structure of **18**, hydrogen atoms are omitted for clarity.

**Scheme 4:**
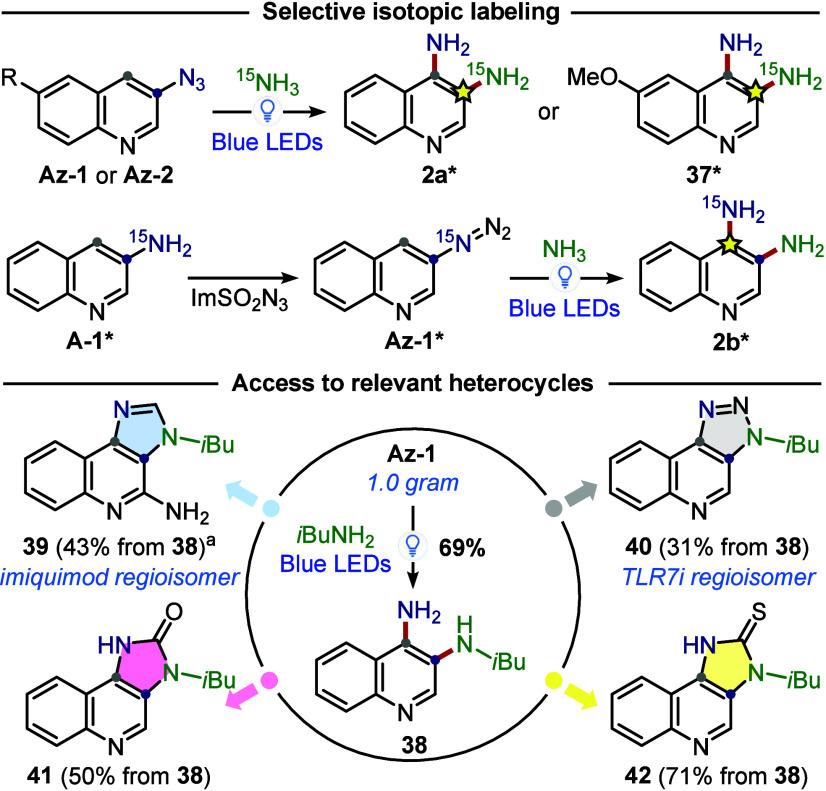
Applications of the Photocatalyzed C4-Selective C–H Amination (See SI for Reaction Conditions)

a Yield over 3 steps.

This regioisomer is generally inaccessible using conventional routes, as exemplified by the syntheses of imiquimod and related drugs (resiquimod, PF-4878691), where a free amino group is typically obtained in the C3*-*position via reduction of 4-chloro-3-nitroquinoline.^6,7,44,45^ The complementarity of our methodology was further demonstrated in the synthesis of **40**, a regioisomer of a triazoloquinoline with immunosuppressive activity, or in the synthesis of imidazoquinol­(thio)­ones **41**–**42**.^5^ Having investigated the versatility of the photocatalyzed C4-selective C–H amination, we then focused our attention on the mechanism of the reaction (Scheme 5A). We hypothesize that visible-light irradiation of **Az-1** induces N_2_ extrusion to generate singlet nitrene. This highly reactive intermediate undergoes insertion (*N*-shift) to form the C4-azirine, which subsequently tautomerizes to form the C4-ketenimine. Nucleophilic addition to this species furnishes the 1,4-benzodiazepine, which then undergoes a thermal electrocyclization to give the C4-aziridine, ultimately rearranging to the more stable product **1**.^46^ Notably, a similar mechanism has been reported for phenyl azides, whereas for naphthyl azides, direct nucleophilic addition to azirines has also been proposed.^47–49^ To rationalize the observed regioselectivity, the regiodetermining nitrene insertion was investigated computationally (Scheme 5B). Following the same approach recently reported by Leonori, Ruffoni, and co-workers for the *ortho*-isomerization of aryl azides, DFT calculations were carried out at the uM06-2X/def2QZVPP//def2TZVP level of theory.^34^ The singlet nitrene derived from **Az-1** was found to display pronounced open-shell singlet character, as evidenced by significant spin contamination (⟨S^2^⟩ = 0.98). Analysis of the total spin density revealed localization at the nitrogen center as well as at the C2*-* and C4-position, consistent with an open-shell singlet description. However, the *N*-shift was predicted to occur preferentially at the C4-position, as indicated by the large energy difference between the transition states leading to the C2- and C4-azirines (ΔΔG­(TS_C2_-TS_C4_) = 7.7 kcal/mol). This preference was attributed to the *ortho*-quinoid character of the C2-azirine, which diminishes aromaticity in the fused benzene ring (not observed for the C4-azirine).^49^ Indeed, C2-azirine was located computationally in a flat region of the potential energy surface, resulting in a barrierless relaxation to the corresponding C2-ketenimine. In line with this interpretation, no regioselectivity was predicted for the *N*-shift of the pyridyl singlet nitrene (ΔΔG­(TS_C2_-TS_C4_) = – 0.8 kcal/mol), whereas replacing quinoline with naphthalene preserved the regiochemical preference (ΔΔG­(TS_C2_-TS_C4_) = 12.0 kcal/mol).

**Scheme 5:**
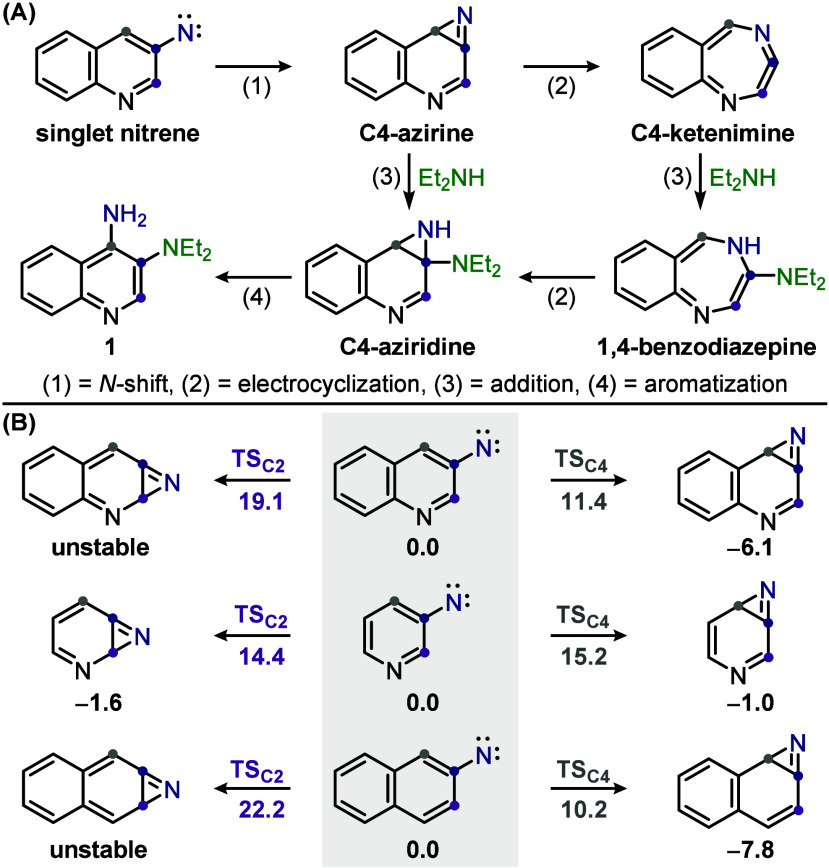
(A) Proposed Reaction Mechanism; (B) Investigation on the Origin of the Regioselectivity Based on Computed Gibbs Free Energies (in kcal/mol)

Furthermore, the observed longer reaction times and lower yield for pyridines are attributed to higher activation barriers, likely amplified by less efficient photoexcitation of the starting 3-azidopyridines, as suggested by their UV/Vis spectra.

Notably, for quinolines, the formation of C4-ketenimine may be disfavored due to its *ortho*-quinoid nature, implying that C4-aziridine formation could indeed proceed via direct nucleophilic addition to the C4-azirine. Instead, for pyridine substrates, the inability to generate *ortho*-quinoids makes the formation of the corresponding 1,4-diazepine more energetically favorable, as supported by computational studies (see Supporting Information).

In conclusion, a mild photochemical C4-selective C–H amination of azidoquinolines has been developed using blue LED irradiation at room temperature. The method introduces a C4–NH_2_ group while simultaneously installing a variety of amines, including −NH_2_, at the C3*-*position, thus allowing predictable functionalization (exploited in selective isotopic labeling). In contrast, 3-azidopyridines preferentially form valuable 1,3- or 1,4-diazepines, depending on the ring substituents. Computational studies suggest that unfavorable quinoid structures account for both the observed regioselectivity in quinolines and the divergent reactivity between quinolines and pyridines. Finally, the resulting 3,4-diaminoquinolines have been employed to synthesize regioisomeric analogues of prominent imidazoquinoline, triazoloquinoline, and imidazoquinol­(thio)­ones, moieties typically inaccessible through conventional methods, thereby opening previously unexplored chemical space.

## Supplementary Material



## Data Availability

The data underlying this study are available in the published article and its Supporting Information.

## Notes

NMR characterizations, DFT calculations, XRD analysis (PDF)
