# Supplementary material for: Photochemical C4-Selective C–H Amination of Quinolines via N‑Shift of Heteroaryl Azides
Source: Org Lett. 2026 Apr 29;28(18):5746–51. doi: 10.1021/acs.orglett.6c01213 (PMC13162312; doi:10.1021/acs.orglett.6c01213)
Supplement: Supplementary file 1 [file ol6c01213_si_001.pdf]

Supporting Information for

Photochemical C4-selective C–H amination of quinolines via N-shift of heteroaryl azides

Alessandro Dimasi,[a] Arianna Montoli,[a] Giovanni Macetti,[a] Leonardo Lo Presti,[a] Daniele Passarella,[a] and Valerio Fasano*[a]

Correspondence to: valerio.fasano@unimi.it

www.fasanolab.com

[a]Department of Chemistry, Università degli Studi di Milano, Via Camillo Golgi, 19, 20133 Milano, Italy

TABLE OF CONTENTS

1. MATERIALS AND GENERAL METHODS 4

1.1 General considerations 4

1.2 Naming of compounds 4

2. EXPERIMENTAL DATA 5

2.1. General Procedures 5

2.1.1 General Procedure A (GP-A): Synthesis of 3-azidoazines 5

2.1.2 General Procedure B (GP-B): Synthesis of 3,4-diaminoazines 5

2.2 Preparation of reagents and intermediates 6

2.2.1 Synthesis of desloratadine 6

2.2.2 Synthesis of trimetazidine 7

2.2.3 Synthesis of *N*[1]-(furan-2-ylmethyl)-*N*[2]-(2-methylnaphthalen-1-yl)oxalamide MNFO 8

2.2.4 Synthesis of 7-chloroquinolin-3-amine 9

2.2.5 Synthesis of 1*H*-imidazole-1-sulfonyl azide hydrochloride 10

2.3 Substrate Scope 11

2.3.1 Synthesis of 3-azidoazines 11

2.3.2 Synthesis of 3,4-diaminoazines 13

2.4 Isotopic labelling experiments 26

2.4.1 Determination of nitrogen NMR resonances before isotopic labeling 26

2.4.2 Synthesis of quinoline-3,4-diamine-^{15}N 2a* 26

2.4.3 Synthesis of 6-methoxyquinoline-3,4-diamine-^{15}N 37* 27

2.4.4 Synthesis of quinolin-3-amine-^{15}N A-1* 27

2.4.5 Synthesis of 3-(azido-1-^{15}N)quinoline Az-1* 28

2.4.6 Synthesis of quinoline-3,4-diamine-^{15}N 2b* 28

2.5 Synthesis of relevant heterocycles 29

2.5.1 Synthesis of *N*[3]-isobutylquinoline-3,4-diamine 38 29

2.5.2 Synthesis of 3-isobutyl-3H-imidazo[4,5-c]quinolin-4-amine 39 29

2.5.3 Synthesis of 3-isobutyl-3*H*-[1,2,3]triazolo[4,5-*c*]quinoline 40 30

2.5.4 Synthesis of 3-isobutyl-1,3-dihydro-2*H*-imidazo[4,5-*c*]quinolin-2-one 41 31

2.5.5 Synthesis of 3-isobutyl-1,3-dihydro-2H-imidazo[4,5-c]quinoline-2-thione 42.......... 32

2.6 Single-crystal X-ray diffraction analysis 32

2.7 UV/Vis analysis 34

3. DFT CALCULATIONS 36

4. SPECTROSCOPIC DATA 47

5. REFERENCES 115

1. MATERIALS AND GENERAL METHODS

1.1 General considerations

Unless stated, all starting materials and anhydrous solvents were obtained from commercial sources and used without purification. Diethylamine, isopropylamine, cyclohexylamine, and octylamine were dried and distilled using standard methods.[1] $^{15}NH_3$ (7M in MeOH) was purchased from Sigma-Aldrich. Reactions were carried out under air unless stated. Reaction progress was monitored by TLC, with ^{1}H NMR or LC-MS analyses taken from reaction samples. All the photochemical reactions were carried out with one Kessil Lamp PR160L (456 nm, 50W, 100% intensity). Column chromatography was performed on silica gel (230-400 mesh). NMR spectra were recorded with a Bruker AV-400 spectrometer (400 MHz ^{1}H; 101 MHz ^{13}C; 376 MHz ^{19}F; 41 MHz ^{15}N). ^{1}H NMR chemical shifts are reported in ppm relative to protio impurities in the deuterated solvents and reported as follow: chemical shift (multiplicity, coupling constants, number of protons). ^{13}C NMR chemical shifts are reported in ppm using the solvent resonance. ^{19}F NMR spectra were recorded using Cl_3CF as an external reference, while ^{15}N NMR spectra were recorded using CH_2NO_2 as internal standard. Coupling constants J are given in Hertz (Hz), while the multiplicity of the signals is indicated as “s”, “d”, “t”, “q”, “pent”, “sept” or “m” for singlet, doublet, triplet, quartet, pentet, septet or multiplet, respectively. Structural assignments were made with additional information from gCOSY, gHSQC, and gHMBC experiments. Mass spectra were recorded on a Waters QTOF mass spectrometer by Electrospray Ionization (ESI). X-ray diffraction analysis was obtained on a Rigaku XtaLAB Synergy-S 4-circle diffractometer using a microfocus sealed tube as a source and Hybrid Photon Counting (HPC) as a detector.

1.2 Naming of compounds

Compound names are those generated by ChemDraw Professional 20.0 software (PerkinElmer), following the IUPAC nomenclature.

2. EXPERIMENTAL DATA

2.1. General Procedures

2.1.1 General Procedure A (GP-A): Synthesis of 3-azidoazines

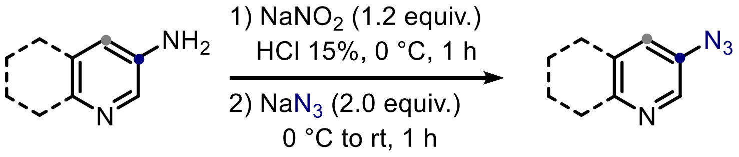

The procedure has been adapted from the literature.[2] In an ice bath, a round bottom flask equipped with a stir bar is charged with quinolin-3-amine (1.0 equiv.) and dissolved in HCl 15% in water (0.50 M). Then, a vial is charged with $NaNO_2$ (1.2 equiv.) dissolved in water (2 M). The obtained solution is added at 0 °C to the flask and maintained at this temperature for 1 hour. After that period, a vial is charged with NaN_3 (2.0 equiv.) dissolved in water (4 M). The obtained solution is added at 0 °C to the flask (Caution: nitrogen gas is generated). Then, the flask is removed from the ice bath and stirred at room temperature for 1 hour. After completion (monitored by TLC), the reaction is quenched with sat. $NaHCO_3$, diluted with AcOEt and the two phases are separated. The aqueous phase is extracted 3 times with AcOEt and the collected organic phases are dried over Na_2SO_4, filtered and concentrated under reduced pressure**.** If required, the crude is purified on flash chromatography column to provide 3-azidoazines.

2.1.2 General Procedure B (GP-B): Synthesis of 3,4-diaminoazines

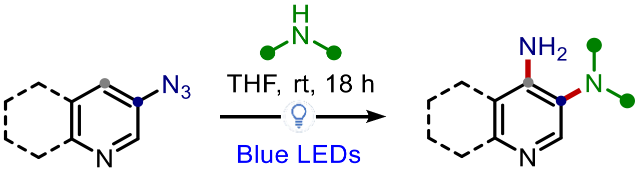

An 8-mL glass vial was charged with 3-azidoazine (1.0 equiv.) and diluted with THF (0.15 M). The corresponding amine (1.1 equiv. for secondary amines, 2.2 equiv. for primary amines, 35.0 or 5.5 equiv. for ammonia) was then added. The reaction vial was capped with a plastic septum, and a fan was placed above it. The reaction mixture was then irradiated with a Kessil lamp (456 nm, 5 cm from vial) for 18 hours. After that, the reaction is concentrated under reduced pressure and the crude is purified on flash chromatography column to provide 3,4-diaminoazines.

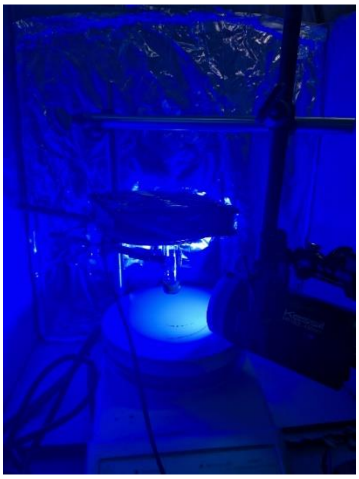

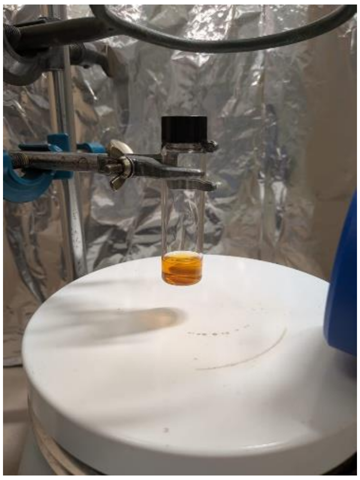

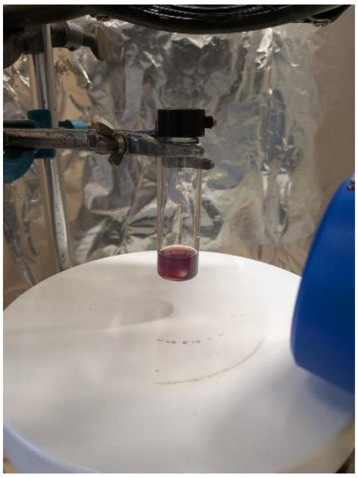

Reaction set-up photos *Reaction mixture before and after irradiation*

2.2 Preparation of reagents and intermediates

2.2.1 Synthesis of desloratadine

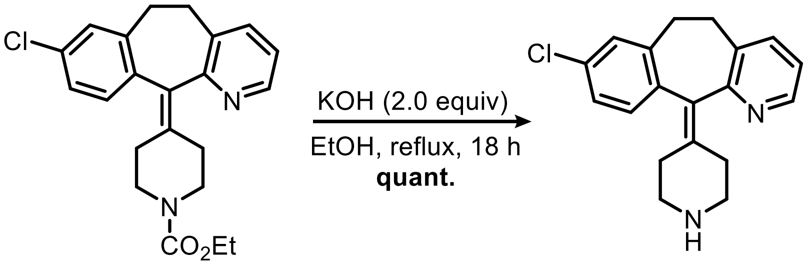

The procedure has been adapted from the literature.[3] A 25-mL double-necked flask was charged with loratadine (383 mg, 1.0 mmol, 1.0 equiv.) and dissolved in EtOH (3.33 mL, 0.3 M). Then KOH was added to the flask (112 mg, 2.0 mmol, 2.0 equiv.) and the reaction was heated at reflux for 18 hours. The reaction was concentrated under reduced pressure and diluted with AcOEt and water. The two phases were separated, and the aqueous phase was extracted 3 times with AcOEt. Organic phases were collected, dried over Na_2SO_4, filtered and concentrated under reduced pressure to provide desloratatdine as an orange solid (310 mg, **quant. yield**). ^{1}H NMR (400 MHz, $CDCl_3$) δ 8.39 (dd, J = 4.8, 1.6 Hz, 1H), 7.43 (dd, J = 7.7, 1.1 Hz, 1H), 7.17 – 7.13 (m, 1H), 7.12 (s, 2H), 7.07 (dd, J = 7.7, 4.8 Hz, 1H), 3.50 – 3.26 (m, 2H), 3.10 – 2.98 (m, 2H), 2.91 – 2.73 (m, 2H), 2.75 – 2.61 (m, 2H), 2.47 – 2.35 (m, 1H), 2.35 – 2.24 (m, 3H), 2.01 (br s, 1H). The spectroscopic data is in agreement with a reported synthesis.[3] (see spectrum)

2.2.2 Synthesis of trimetazidine

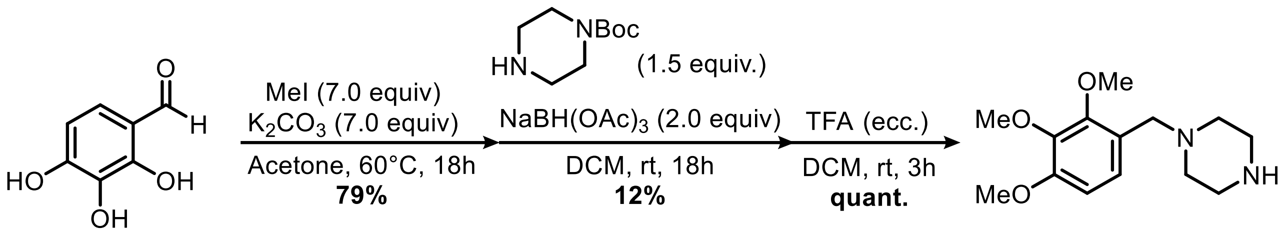

Step 1: synthesis of 2,3,4-trimethoxybenzaldehyde

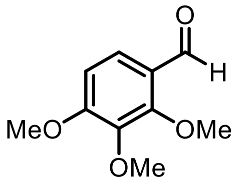

The procedure has been adapted from the literature.[4] A 25-mL double-necked flask was charged with 2,3,4-trihydroxybenzaldehyde (462 mg, 3.0 mmol, 1.0 equiv.), K_2CO_3 (2.902 g, 21 mmol, 7.0 equiv) and dissolved in acetone (12 mL, 0.25 M). Then MeI was added to the flask (1.274 mL, 21 mmol, 7.0 equiv.) and the reaction was heated at 60 °C for 18 hours. The reaction was concentrated under reduced pressure and diluted with AcOEt and water. The two phases were separated, and the aqueous phase was extracted 3 times with AcOEt. Organic phases were collected, dried over Na_2SO_4, filtered and concentrated under reduced pressure to provide 2,3,4-trimethoxybenzaldehyde as a white solid (465 mg, **79% yield**). ^{1}H NMR (400 MHz, $CDCl_3$) δ 10.17 (s, 1H), 7.53 (d, J = 8.8 Hz, 1H), 6.70 (d, J = 8.8 Hz, 1H), 3.97 (s, 3H), 3.88 (s, 3H), 3.82 (s, 3H). The spectroscopic data is in agreement with a reported synthesis.[4] (see spectrum)

Step 2: synthesis of tert-butyl 4-(2,3,4-trimethoxybenzyl)piperazine-1-carboxylate

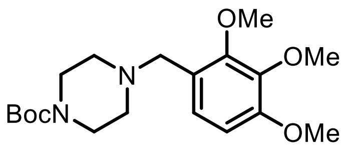

A 25-mL single-necked flask was charged with 2,3,4-trimethoxybenzaldehyde (465 mg, 2.37 mmol, 1.0 equiv.), tert-butyl piperazine-1-carboxylate (662 mg, 3.55 mmol, 1.5 equiv) and dissolved in DCM (4.74 mL, 0.50 M). Then $NaBH(OAc)_3$ was added portionwise to the flask (1.00 g, 4.73 mmol, 2.0 equiv.) and the reaction was stirred at room temperature for 18 hours. The reaction was quenched with 1 M NaOH until basic pH was reached. The two phases were separated, and the aqueous phase extracted 3 times with DCM. Organic phases were collected, washed with brine, dried over Na_2SO_4, filtered and concentrated under reduced pressure. The obtained crude was purified on flash chromatography column (DCM:MeOH 95:5) to provide tert-butyl 4-(2,3,4-trimethoxybenzyl)piperazine-1-carboxylate as a colourless oil (103 mg, **12% yield**). ^{1}H NMR (400 MHz, $CDCl_3$) δ 6.95 (d, J = 8.5 Hz, 1H), 6.61 (d, J = 8.5 Hz, 1H), 3.85 (s, 3H), 3.85 (s, 3H), 3.83 (s, 3H), 3.45 (s, 2H), 3.39 (t, J = 5.1 Hz, 4H), 2.38 (t, J = 5.0 Hz, 4H), 1.42 (s, 9H). The spectroscopic data is in agreement with a reported synthesis.[5] (see spectrum)

Step 3: synthesis of trimetazidine (1-(2,3,4-trimethoxybenzyl)piperazine)

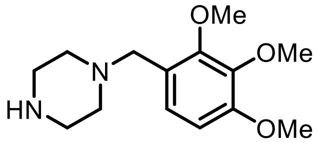

A 25-mL single-necked flask was charged with tert-butyl 4-(2,3,4-trimethoxybenzyl)piperazine-1-carboxylate (103 mg, 0.3 mmol, 1.0 equiv.) and dissolved in DCM (1.0 mL, 0.3 M). Then an excess of TFA was added dropwise to the flask and the reaction was stirred at room temperature for 18 hours. The reaction was quenched with sat. $NaHCO_3$ solution until neutral pH was reached. The two phases were separated, and the aqueous phase was extracted 3 times with DCM. Organic phases were collected, dried over Na_2SO_4, filtered and concentrated under reduced pressure to obtain trimetazidine as a yellow pale oil (88 mg, **quant**. **yield**). ^{1}H NMR (400 MHz, $CDCl_3$) δ 6.99 (d, J = 8.5 Hz, 1H), 6.63 (d, J = 8.5 Hz, 1H), 3.88 (s, 3H), 3.87 (s, 3H), 3.85 (s, 3H), 3.47 (s, 2H), 2.89 (t, J = 4.9 Hz, 4H), 2.54 – 2.39 (m, 4H). The spectroscopic data agrees with a reported synthesis.[6] (see spectrum)

2.2.3 Synthesis of *N*¹-(furan-2-ylmethyl)-*N*²-(2-methylnaphthalen-1-yl)oxalamide MNFO

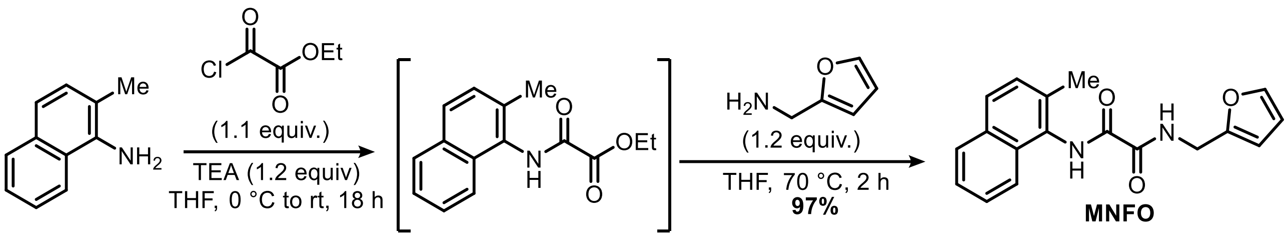

The procedure has been adapted from the literature.[7] A 10 mL double-necked flask equipped with a stirring bar was charged with 2-methylnaphthalen-1-amine (471 mg, 3.0 mmol, 1.0 equiv.) and dissolved in THF (6 mL). TEA was added (501 µL, 3.6 mmol, 1.2 equiv.) and this solution was brought to 0 °C in an ice bath. Ethyl chlorooxoacetate (369 µL, 3.3 mmol, 1.1 equiv.) was added dropwise at 0 °C and then reaction was stirred at room temperature for 18 hours. The mixture was then diluted with AcOEt and water. The two phases were separated, and the organic phase was washed with water, dried over Na_2SO_4, filtered and concentrated under reduced pressure. The obtained crude was transferred to a 10 mL double-necked flask and diluted with THF (3 mL). Furan-2-ylmethanamine (318 µL, 3.6 mmol, 1.2 equiv.) was added the reaction was heated at 70 °C and left stirring for 18 hours. The obtained crude was concentrated in vacuo and purified on flash chromatography column (from 100% Hex to Hex:AcOEt 7:3) to afford **MNFO** as a white solid (896 mg, **97% yield**). ^{1}H NMR (400 MHz, $CDCl_3$) δ 9.17 (s, 1H), 7.90 – 7.69 (m, 4H), 7.53 – 7.32 (m, 4H), 6.40 – 6.30 (m, 2H), 4.61 (d, J = 5.9 Hz, 2H), 2.41 (s, 3H). The spectroscopic data are in agreement with a reported synthesis.[7] (see spectrum).

2.2.4 Synthesis of 7-chloroquinolin-3-amine

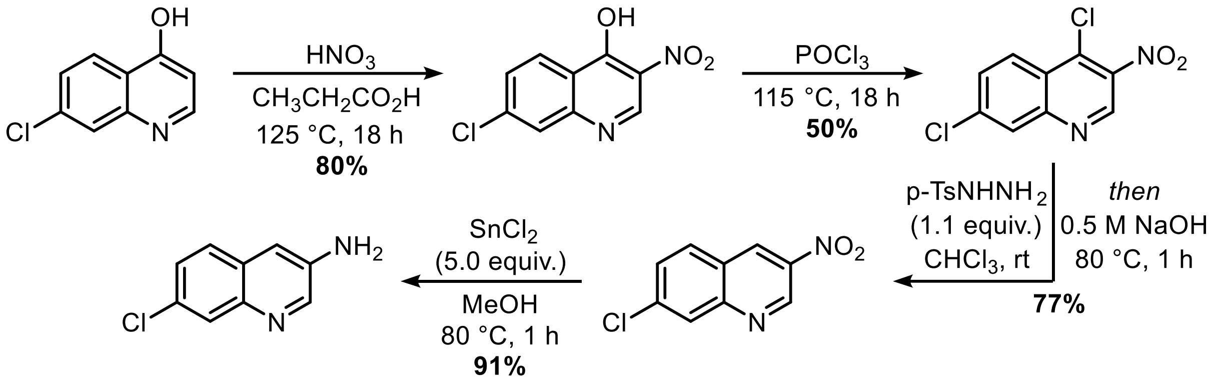

The procedures have been adapted from the literature.[8]

Step 1: Synthesis of 7-chloro-3-nitroquinolin-4-ol

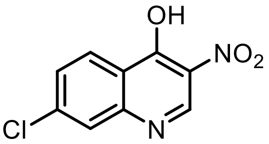

7-chloroquinolin-4-ol (4.0 g, 22.3 mmol, 1.0 equiv.) was dissolved in propionic acid (40 mL, 0.6 M) and heated to 125 °C. Nitric acid (65% conc, 1.9 mL) was added dropwise to the stirred solution. After 18 hours, the mixture was diluted with ethanol, and a solid was collected by filtration yielding the pure product (4.0 g, 17.8 mmol, **80% yield**). ^{1}H NMR (400 MHz, DMSO-d_6) δ 9.23 (s, 1H), 8.23 (d, J = 8.7 Hz, 1H), 7.75 (d, J = 2.0 Hz, 1H), 7.54 (dd, J = 8.7, 2.0 Hz, 1H). The data are consistent with those reported in the literature.[8] (see spectrum)

Step 2: Synthesis of 4,7-dichloro-3-nitroquinoline

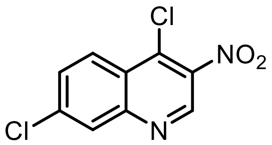

7-chloro-3-nitroquinolin-4-ol (2.0 g, 8.9 mmol, 1.0 equiv.) and $POCl_3$ (8.0 mL) were heated to 115 °C overnight. After completion, the solution was cooled to room temperature and poured into an ice/water mixture (50 mL). The precipitate was filtered, washed with water and dried. The crude was purified by automated direct phase chromatography (eluent mixture 8:2 hex/AcOEt) to afford the pure product (1.0 g, 4.5 mmol, **50% yield**). ^{1}H NMR (400 MHz, $CDCl_3$) δ 9.27 (s, 1H), 8.37 (d, J = 9.0 Hz, 1H), 8.22 (d, J = 2.0 Hz, 1H), 7.76 (dd, J = 9.1, 2.1 Hz, 1H). The data are consistent with those reported in the literature.[8] (see spectrum)

Step 3: Synthesis of 7-chloro-3-nitroquinoline

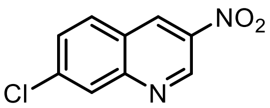

A solution of 4,7-dichloro-3-nitroquinoline (560 mg, 2.3 mmol, 1.1 equiv.) and p-toluenesulfonylhydrazine (472 mg, 2.5 mmol, 1.1 equiv.) in $CHCl_3$ (21 mL, 0.11 M) was stirred at room temperature for 24 hours. The solvent was evaporated and the residue was redissolved in 0.5 M NaOH (50 mL) and heated to 80 °C for 1 hour. The dark-red mixture was brought to room temperature, and the precipitate was filtered and washed with water, affording the pure product as a brown solid (370 mg, 1.8 mmol, **77% yield**). ^{1}H NMR (400 MHz, $CDCl_3$) δ 9.66 (d, J = 2.5 Hz, 1H), 9.03 (d, J = 2.5 Hz, 1H), 8.26 (d, J = 2.0 Hz, 1H), 7.99 (d, J

= 8.7 Hz, 1H), 7.70 (dd, J = 8.8, 2.1 Hz, 1H). The data are consistent with those reported in the literature.[8] (see spectrum)

Step 4: Synthesis of 7-chloroquinolin-3-amine

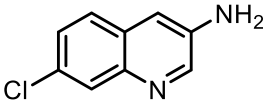

A solution of 7-chloro-3-nitroquinoline (370 mg, 1.8 mmol, 1.0 equiv.) and $SnCl_2$ (1.7 g, 8.9 mmol, 5.0 equiv.) in dry MeOH (5.0 mL, 0.36 M) was heated to reflux for 1 hour under nitrogen atmosphere. After completion, the solvent was evaporated and the residue was dissolved in AcOEt and sat. $NaHCO_3$. The resulting mixture was filtered through a celite pad and washed with AcOEt, followed by separation of the phases. The organic layer was collected, dried over anhydrous Na_2SO_4 and concentrated under reduced pressure to obtain the pure product (287 mg, 1.6 mmol, **91% yield**). ^{1}H NMR (400 MHz, $CDCl_3$) δ 8.56 (d, J = 2.7 Hz, 1H), 7.99 (d, J = 2.1 Hz, 1H), 7.54 (d, J = 8.8 Hz, 1H), 7.39 (dd, J = 8.8, 2.1 Hz, 1H), 7.25 (d, J = 2.8 Hz, 1H), 3.98 (brs, 2H). The data are consistent with those reported in the literature.[8] (see spectrum)

2.2.5 Synthesis of 1*H*-imidazole-1-sulfonyl azide hydrochloride

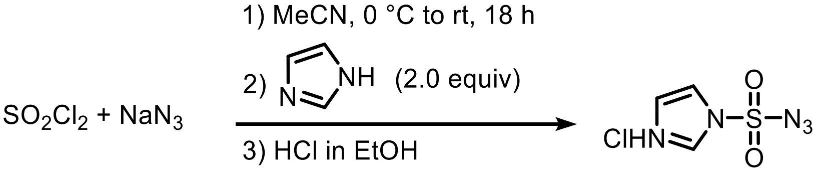

The procedure has been adapted from the literature.[9] Sulfuryl chloride (405 µL, 5 mmol, 1.0 equiv.) was added dropwise to an ice-cooled suspension of NaN_3 (325 mg, 5 mmol, 1.0 equiv) in anhydrous acetonitrile (5 mL) and the mixture was stirred overnight. Imidazole (681 mg, 10 mmol, 2.0 equiv) was added portionwise to the ice-cooled mixture and the slurry was stirred for 5 hours. The mixture was diluted with AcOEt and water. The organic layer was washed with water and then by $NaHCO_3$ saturated solution. The organic layer was dried over Na_2SO_4 and filtered. A solution of HCl in EtOH, obtained by dropwise addition at 0 °C of acyl chloride (533 µL, 7.5 mmol, 1.5 equiv) to ice-cooled dry ethanol (2 mL) was added dropwise to the filtrate with stirring, obtaining a white precipitate which was dried to get 1*H*-imidazole-1-sulfonyl azide hydrochloride as a white crystalline solid (664 mg, **63% yield**). ^{1}H NMR (400 MHz, D_2O) δ 9.17 (t, J = 1.3 Hz, 1H), 8.01 (t, J = 1.6 Hz, 1H), 7.59 (dd, J = 2.1, 1.2 Hz, 1H). The spectroscopic data are in agreement with those reported in the literature.[10] (see spectrum)

2.3 Substrate Scope

2.3.1 Synthesis of 3-azidoazines

Synthesis of 3-azidoquinoline Az-1

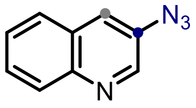

Az-1

Prepared according to general procedure A (GP-A) using 3-aminoquinoline (721 mg, 5.0 mmol. 1.0 equiv.), $NaNO_2$ (414 mg, 6 mmol, 1.2 equiv.) NaN_3 (630 mg, 10 mmol, 2.0 equiv.) and HCl 15% (5 mL). Compound **Az-1** is obtained without further purification as a pale-yellow solid (825 mg, **97% yield**). ^1H NMR (400 MHz, $CDCl_3$) δ 8.62 (d, *J* = 2.6 Hz, 1H), 8.09 (dd, *J* = 8.7, 0.8 Hz, 1H), 7.79 – 7.74 (m, 2H), 7.67 (ddd, *J* = 8.4, 6.9, 1.5 Hz, 1H), 7.57 (ddd, *J* = 8.2, 6.9, 1.2 Hz, 1H). The spectroscopic data agree with a reported synthesis.[2] (see spectrum)

Synthesis of 3-azido-6-methoxyquinoline Az-2

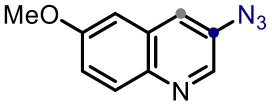

Az-2

Prepared according to general procedure A (GP-A) using 3-amino-6-methoxyquinoline (180 mg, 1.03 mmol, 1.0 equiv.), $NaNO_2$ (108 mg, 1.56 mmol, 1.2 equiv,) NaN_3 (134 mg, 2.06 mmol, 2.0 equiv.) and HCl 15% (3 mL). The obtained crude is purified on flash chromatography column (from 100% Hex to Hex:AcOEt 7:3) to obtain the title compound **Az-2** as a pale-yellow solid (122 mg, **59% yield**). ^1H NMR (400 MHz, $CDCl_3$) δ 8.45 (d, *J* = 2.6 Hz, 1H), 7.95 (d, *J* = 9.2 Hz, 1H), 7.62 (d, *J* = 2.6 Hz, 1H), 7.30 (dd, *J* = 9.2, 2.7 Hz, 1H), 6.99 (d, *J* = 2.8 Hz, 1H), 3.92 (s, 3H). ^{13}C NMR (101 MHz, $CDCl_3$) δ 158.9, 142.2, 141.3, 134.5, 131.0, 129.6, 121.7, 121.6, 104.5, 55.7. MS (ESI), m/z $[M+H]^+$: 201.51. HRMS (ESI), [M+H]+: Calculated for $[C_{10}H_8NO]^+$: 158.0606, found: 158.0605 (see spectra)

Synthesis of 3-azido-6-bromo-7-fluoroquinoline Az-3

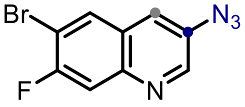

Az-3

Prepared according to general procedure A (GP-A) using 6-bromo-7-fluoroquinolin-3-amine (139 mg, 0.62 mmol, 1.0 equiv.), $NaNO_2$ (52 mg, 0.74 mmol, 1.2 equiv,) NaN_3 (81 mg, 1.24 mmol, 2.0 equiv.) and HCl 15% (3 mL). Compound **Az-3** is obtained without further purification as a pale-yellow solid (140 mg, **84% yield**). ^1H NMR (400 MHz, $CDCl_3$) δ 8.63 (d, *J* = 2.6 Hz, 1H), 8.01 (d, *J* = 7.2 Hz, 1H), 7.78 (d, *J* = 9.2 Hz, 1H), 7.65 (d, *J* = 2.6 Hz, 1H). ^{13}C NMR (101 MHz, $CDCl_3$) δ 158.4 (d, *J* = 251.3 Hz), 145.4 (d, *J* = 10.8 Hz), 145.2, 134.5, 131.1, 126.0, 121.4, 114.5 (d, *J* = 22.1 Hz), 112.0 (d, *J* = 24.5 Hz). ^{19}F NMR (376 MHz, $CDCl_3$) δ −105.1. MS (ESI), m/z $[M+H]^+$: 267.01. HRMS (ESI), [M+H]+: Calculated for $[C_9H_5BrFN_4]^+$: 266.0614, found: 266.0620 (see spectra).

Synthesis of 3-azido-7-chloroquinoline Az-4

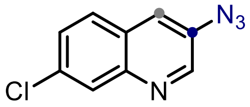

Az-4

Prepared according to general procedure A (GP-A) starting from 7-chloroquinolin-3-amine (287 mg, 1.6 mmol, 1.0 equiv.), $NaNO_2$ (139 mg, 2.0 mmol, 1.2 equiv.) and NaN_3 (218 mg, 3.3 mmol, 2.0 equiv.) and HCl 15% (3 mL). Compound **Az-4** is obtained without further purification as a brown solid (278 mg, 1.4 mmol,

85% yield). ^{1}H NMR (400 MHz, $CDCl_3$) δ 8.61 (d, J = 2.6 Hz, 1H), 8.07 (d, J = 2.1 Hz, 1H), 7.72 (dd, J = 2.6, 0.9 Hz, 1H), 7.70 (d, J = 8.8 Hz, 1H), 7.52 (dd, J = 8.8, 2.1 Hz, 1H). ^{13}C NMR (101 MHz, $CDCl_3$) δ 145.9, 144.8, 134.5, 134.3, 128.7, 128.4, 127.9, 126.5, 122.3. MS (ESI), m/z $[M+H]^+$: 205.44. HRMS (ESI), [M+H]+: Calculated for $[C_9H_6ClN_4]^+$: 204.0203, found: 204.0207 (see spectra)

Synthesis of 3-azido-5-methylquinoline Az-5

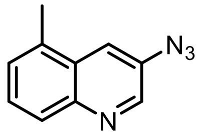

Az-5

Prepared according to general procedure A (GP-A) using 5-methylquinolin-3-amine (175 mg, 1.1 mmol, 1.0 equiv.), $NaNO_2$ (92 mg, 1.3 mmol, 1.2 equiv,) NaN_3 (143 mg, 2.2 mmol, 2.0 equiv.) and HCl 15% (2 mL) to afford compound **Az-5** (162 mg, 0.88 mmol, **79% yield**. ^{1}H NMR (400 MHz, $CDCl_3$) δ 8.53 (d, *J* = 2.6 Hz, 1H), 7.89 (d, *J* = 8.5 Hz, 1H), 7.65 (d, *J* = 3.2 Hz, 1H), 7.48 (dd, *J* = 8.5, 7.0 Hz, 1H), 7.30 (d, *J* = 7.1 Hz, 1H), 2.54 (s, 3H). ^{13}C NMR (101 MHz, $CDCl_3$) δ 145.8, 142.9, 133.5, 133.2, 128.2, 127.9, 127.4, 127.3, 119.0, 18.3. MS (ESI), m/z $[M+H]^+$: 185.90. HRMS (ESI), [M+H]+: Calculated for $[C_{10}H_8N_4]^+$: 184.0749, found: 184.0750 (see spectra)

Synthesis of 3-azido-6-(trifluoromethyl)quinoline Az-6

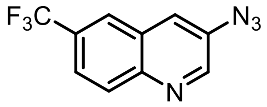

Az-6

Prepared according to general procedure A (GP-A) using 6-(trifluoromethyl)quinolin-3-amine (43 mg, 0.20 mmol, 1.0 equiv.), $NaNO_2$ (17 mg, 0.24 mmol, 1.2 equiv,) NaN_3 (26 mg, 0.40 mmol, 2.0 equiv.) and HCl 15% (1 mL). The crude is purified by automated direct phase chromatography (eluent mixture: hex:AcOEt) to afford compound **Az-6** (28 mg, 0.12 mmol, **60% yield**). ^{1}H NMR (400 MHz, $CDCl_3$) δ 8.72 (d, *J* = 2.6 Hz, 1H), 8.20 (dd, *J* = 9.1, 1.2 Hz, 1H), 8.14 – 8.03 (m, 1H), 7.91 – 7.71 (m, 2H). ^{13}C NMR (101 MHz, $CDCl_3$) δ 146.6, 146.2, 135.6, 130.8, 129.9 (q, *J* = 32.9 Hz), 127.4, 124.9 (q, *J* = 4.4 Hz), 124.6 (q, *J* = 3.1 Hz), 124.0 (q, *J* = 272.6 Hz), 123.2. ^{19}F NMR (376 MHz, $CDCl_3$) δ −62.57. MS (ESI), m/z $[M+H]^+$: 239.61. HRMS (ESI), [M+H]+: Calculated for $[C_{10}H_5F_3N_4]^+$: 238.0466, found: 238.0468 (see spectra)

Synthesis of 5-azido-2-phenylpyridine Az-7

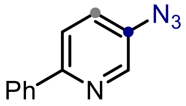

Az-7

Prepared according to general procedure A (GP-A) using 6-phenylpyridin-3-amine (30 mg ,0.17 mmol, 1.0 equiv.), $NaNO_2$ (14 mg, 0.20 mmol, 1.2 equiv.), NaN_3 (22mg, 0.34 mmol, 2.0 equiv) and HCl 15% (3 mL). The obtained crude is purified on flash chromatography column (from 100% Hex to Hex:AcOEt 7:3) to obtain the title compound **Az-7** as a white solid (19.5 mg, **58% yield**). ^{1}H NMR (400 MHz, $CDCl_3$) δ 8.43 (dd, J = 2.8, 0.6 Hz, 1H), 8.00 – 7.92 (m, 2H), 7.73 (dd, J = 8.6, 0.8 Hz, 1H), 7.51 – 7.37 (m, 4H). The spectroscopic data agree with a reported synthesis.[11] (see spectrum)

Synthesis of methyl 5-azidonicotinate Az-8

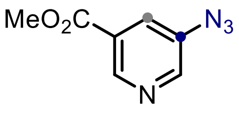

Az-8

Prepared according to general procedure A (GP-A) using methyl 5-aminonicotinate (130 mg, 0.85 mmol, 1,0 equiv.), $NaNO_2$ (70 mg, 1.02 mmol, 1.2 equiv.), NaN_3 (110 mg, 1.7 mmol, 2.0 equiv) and HCl 15% (3 mL). Compound **Az-8** is obtained without further purification as an orange solid (145 mg, **95% yield**). ^{1}H NMR (400 MHz, $CDCl_3$) δ 8.98 (d, *J* = 1.8 Hz, 1H), 8.50 (d, *J* = 2.7 Hz, 1H), 7.95 (dd, *J* = 2.7, 1.7 Hz, 1H), 3.97 (s, 3H). ^{13}C NMR (101 MHz, $CDCl_3$) δ 165.1, 146.9, 145.0, 137.6, 127.0, 126.7, 52.8. MS (ESI), m/z $[M-H]^-$: 179.04. HRMS (ESI), [M+H]+: Calculated for $[C_7H_9N_4O_2]^+$: 180.1687, found: 180.1678. (see spectra)

2.3.2 Synthesis of 3,4-diaminoazines

Synthesis of *N*^{3},*N*^{3}-diethylquinoline-3,4-diamine 1

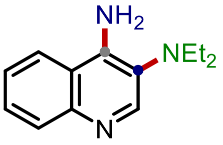

1

Prepared according to general procedure B (GP-B) using 3-azidoquinoline **Az-1** (34 mg, 0.2 mmol, 1.0 equiv.), diethylamine (23 µL, 0.22 mmol, 1.1 equiv.) and THF (1.30 mL). The obtained crude is purified on flash chromatography column (100% AcOEt + 1% TEA) to obtain the title compound **1** as a pale yellow solid (34 mg, **79% yield**). ^{1}H NMR (400 MHz, $CDCl_3$) δ 8.48 (s, 1H), 7.99 (d, *J* = 8.5 Hz, 1H), 7.87 (dd, *J* = 8.4, 1.4 Hz, 1H), 7.59 (ddd, *J* = 8.4, 6.8, 1.3 Hz, 1H), 7.42 (ddd, *J* = 8.2, 6.8, 1.2 Hz, 1H), 5.71 (brs, 2H), 3.05 (q, *J* = 7.1 Hz, 4H), 0.99 (t, *J* = 7.1 Hz, 6H). ^{13}C NMR (101 MHz, $CDCl_3$) δ 148.7, 145.8, 145.0, 129.2, 128.3, 125.9, 125.0, 121.6, 118.2, 49.1, 13.1. MS (ESI), m/z $[M+H]^+$: 216.225. HRMS (ESI), [M+H]+: Calculated for $[C_{13}H_{18}N_3]^+$: 216.1495, found: 216.1498. (see spectra)

Synthesis of quinoline-3,4-diamine 2

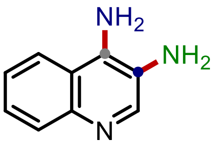

2

Prepared according to general procedure B (GP-B) using 3-azidoquinoline **Az-1** (34 mg, 0.2 mmol, 1.0 equiv.), ammonia (7.0 M in MeOH, 1.0 mL, 7 mmol, 35.0 equiv.) and THF (1.30 mL). The obtained crude is purified on flash chromatography column (AcOEt:MeOH 9:1 +1% TEA) to obtain the title compound **2** as a red/brownish solid (21 mg, **66% yield**). The same reaction repeated with 5.5 equiv. of ammonia gave the title compound **2** as a red/brownish solid (14 mg**, 44% yield**). ^{1}H NMR (400 MHz, DMSO) δ 8.21 (s, 1H), 8.02 – 7.94 (m, 1H), 7.70 – 7.61 (m, 1H), 7.36 – 7.22 (m, 2H), 5.82 (brs, 2H), 4.70 (brs, 2H). MS (ESI), m/z $[M+H]^+$: 160.89. The spectroscopic data agree with a reported synthesis.[12] (see spectra) *Some unidentified impurity could not be removed from this product even after a second purification on silica gel (direct phase or inverse phase).*

Synthesis of *N*^{3}-octylquinoline-3,4-diamine 3

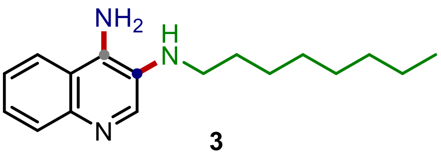

3

Prepared according to general procedure B (GP-B) using 3-azidoquinoline **Az-1** (34 mg, 0.2 mmol, 1.0 equiv.), octan-1-amine (73 µL, 0.44 mmol, 2.2 equiv.) and THF (1.30 mL). The obtained crude is purified on flash chromatography column

(100% AcOEt + 1% TEA) to obtain the title compound **3** as a yellow oil (31 mg, **57% yield**). ^{1}H NMR (400 MHz, $CDCl_3$) δ 8.44 (s, 1H), 7.95 (d, *J* = 8.4 Hz, 1H), 7.71 (d, *J* = 8.4 Hz, 1H), 7.51 (t, *J* = 7.1 Hz, 1H), 7.41 (t, *J* = 7.5 Hz, 1H), 4.74 (brs, 2H), 3.15 (t, *J* = 7.1 Hz, 2H), 2.75 (brs, 1H), 1.65 (p, *J* = 7.2 Hz, 2H), 1.43 (p, *J* = 7.0 Hz, 2H), 1.38 – 1.12 (m, 8H), 0.88 (t, *J* = 6.8 Hz, 3H). ^{13}C NMR (101 MHz, $CDCl_3$) δ 144.6, 142.0, 139.4, 129.5, 126.9, 126.2, 125.0, 119.9, 118.6, 46.4, 31.9, 30.4, 29.6, 29.4, 27.3, 22.7, 14.2. MS (ESI), m/z $[M+H]^+$: 272.447. HRMS (ESI), [M+H]+: Calculated for $[C_{17}H_{26}N_3]^+$: 272.4155, found: 272.4157. (see spectra)

Synthesis of *N*³- isopropylquinoline-3,4-diamine 4

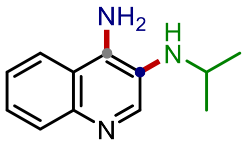

4

Prepared according to general procedure B (GP-B) using 3-azidoquinoline **Az-1** (34 mg, 0.2 mmol, 1.0 equiv.), propan-2-amine (38 µL, 0.44 mmol, 2.2 equiv.) and THF (1.30 mL). The obtained crude is purified on flash chromatography column (100% AcOEt + 1% TEA) to obtain the title compound **4** as a yellow oil (23 mg, **57% yield**). ^{1}H NMR (400 MHz, $CDCl_3$) δ 8.41 (s, 1H), 7.94 (d, *J* = 8.4 Hz, 1H), 7.72 (d, *J* = 8.4 Hz, 1H), 7.51 (t, *J* = 7.6 Hz, 1H), 7.40 (t, *J* = 7.6 Hz, 1H), 4.95 (brs, 2H), 3.51 (hept, *J* = 6.5 Hz, 1H), 2.94 (brs, 1H), 1.19 (d, *J* = 6.3 Hz, 6H). ^{13}C NMR (101 MHz, $CDCl_3$) δ 145.0, 144.9, 141.9, 129.4, 127.4, 124.9, 124.3, 120.2, 118.7, 47.2, 23.5. MS (ESI), m/z $[M+2H]^{2+}$: 202.131. HRMS (ESI), [M+H]+: Calculated for $[C_{12}H_{16}N_3]^+$: 202.2805, found: 202.2803. (see spectra)

Synthesis of *N*³-cyclohexylquinoline-3,4-diamine 5

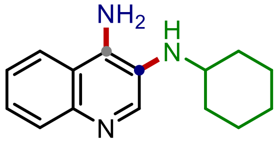

5

Prepared according to general procedure B (GP-B) using 3-azidoquinoline **Az-1** (34 mg, 0.2 mmol, 1.0 equiv.), cyclohexanamine (50 µL, 0.44 mmol, 2.2 equiv.) and THF (1.30 mL). The obtained crude is purified on flash chromatography column (100% AcOEt + 1% TEA) to obtain the title compound **5** as a yellow pale solid (35 mg, **66% yield**). ^{1}H NMR (400 MHz, $CDCl_3$) δ 8.37 (s, 1H), 7.93 (d, J = 8.4 Hz, 1H), 7.73 (d, J = 8.4 Hz, 1H), 7.50 (t, J = 7.6 Hz, 1H), 7.38 (t, J = 7.6 Hz, 1H), 5.06 (brs, 2H), 3.27 (brs, 1H), 3.07 (dt, J = 10.2, 5.9 Hz, 1H), 2.10 – 1.91 (m, 2H), 1.81 – 1.67 (m, 2H), 1.68 – 1.54 (m, 1H), 1.35 – 1.08 (m, 5H). ^{13}C NMR (101 MHz, $CDCl_3$) δ 147.0, 145.0, 141.8, 129.4, 127.3, 124.9, 124.1, 120.2, 118.7, 54.7, 34.2, 26.0, 25.1. MS (ESI), m/z $[M+H]^+$: 242.536. HRMS (ESI), [M+H]+: Calculated for $[C_{15}H_{20}N_3]^+$: 242.3455, found: 242.3450. (see spectra)

Synthesis of *N*³-(*tert*-butyl)quinoline-3,4-diamine 6

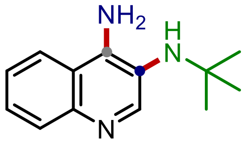

6

Prepared according to general procedure B (GP-B) using 3-azidoquinoline **Az-1** (34 mg, 0.2 mmol, 1.0 equiv.), 2-methylpropan-2-amine (46 µL, 0.44 mmol, 2.2 equiv.) and THF (1.30 mL). The obtained crude is purified on flash chromatography column (100% AcOEt + 1% TEA) to obtain the title compound **6** as a yellow solid (17 mg, **39% yield**). ^{1}H NMR (400 MHz, $CDCl_3$) δ 8.42 (s, 1H), 7.95 (dd, J = 8.4, 1.2 Hz, 1H), 7.74 (dd, J = 8.4, 1.4 Hz, 1H), 7.59 (ddd, J = 8.3, 6.8, 1.3 Hz, 1H), 7.42 (ddd, J = 8.2, 6.9, 1.2 Hz, 1H), 5.25 (brs, 2H), 2.65 (brs, 1H), 1.22 (s, 9H). ^{13}C NMR (101 MHz, $CDCl_3$) δ 151.7,

148.4, 146.6, 143.3, 129.6, 128.5, 124.8, 121.0, 118.8, 54.7, 30.1. MS (ESI), m/z $[M+H]^+$: 216.489. HRMS (ESI), [M+H]+: Calculated for $[C_{13}H_{18}N_3]^+$: 216.3075, found: 216.3077. (see spectra)

Synthesis of *N*^{3}-(adamantan-1-yl)quinoline-3,4-diamine 7

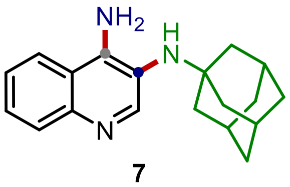

7

Prepared according to general procedure B (GP-B) using 3-azidoquinoline **Az-1** (34 mg, 0.2 mmol, 1.0 equiv.), adamantan-1-amine (67 mg, 0.44 mmol, 2.2 equiv.) and THF (1.30 mL). The obtained crude is purified on flash chromatography column (100% AcOEt + 1% TEA) to obtain the title compound **7** as a withe solid (17 mg, **29% yield**). ^{1}H NMR (400 MHz, $CDCl_3$) δ 8.39 (s, 1H), 7.96 (dd, J = 8.5, 1.2 Hz, 1H), 7.74 (dd, J = 8.4, 1.4 Hz, 1H), 7.58 (ddd, J = 8.4, 6.8, 1.4 Hz, 1H), 7.42 (ddd, J = 8.2, 6.8, 1.3 Hz, 1H), 5.29 (brs, 2H), 2.58 (brs, 1H), 2.06 (p, J = 3.0 Hz, 3H), 1.76 (d, J = 2.9 Hz, 6H), 1.69 – 1.59 (m, 3H), 1.59 – 1.51 (m, 3H). ^{13}C NMR (101 MHz, $CDCl_3$) δ 152.2, 148.4, 146.7, 129.7, 128.4, 124.7, 121.0, 119.6, 119.6, 54.9, 43.8, 36.5, 29.8. MS (ESI), m/z $[M+H]^+$: 294.293. HRMS (ESI), [M+H]+: Calculated for $[C_{19}H_{24}N_3]^+$: 294.4215, found: 294.4218. (see spectra)

Synthesis of *N*^{3}-benzylquinoline-3,4-diamine 8

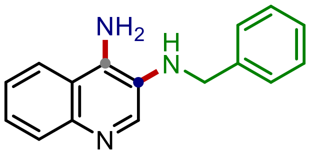

8

Prepared according to general procedure B (GP-B) using 3-azidoquinoline **Az-1** (34 mg, 0.2 mmol, 1.0 equiv.), phenylmethanamine (48 µL, 0.44 mmol, 2.2 equiv.) and THF (1.30 mL). The obtained crude is purified on flash chromatography column (100% AcOEt + 1% TEA) to obtain the title compound **8** as a yellow pale solid (32 mg, **64% yield**). ^{1}H NMR (400 MHz, $CDCl_3$) δ 8.40 (s, 1H), 7.94 (dd, J = 8.5, 1.2 Hz, 1H), 7.72 (dd, J = 8.4, 1.6 Hz, 1H), 7.52 (ddd, J = 8.4, 6.8, 1.4 Hz, 1H), 7.48 – 7.22 (m, 6H), 4.82 (brs, 2H), 4.34 (s, 2H). ^{13}C NMR (101 MHz, $CDCl_3$) δ 144.7, 142.2, 139.8, 139.3, 129.5, 128.8, 128.0, 127.6, 127.2, 125.5, 125.1, 119.9, 118.5, 50.6. MS (ESI), m/z $[M+H]^+$: 250.199. HRMS (ESI), [M+H]+: Calculated for $[C_{16}H_{16}N_3]^+$: 250.1339, found: 250.1342. (see spectra)

Synthesis of *N*^{3}-(4-fluorobenzyl)quinoline-3,4-diamine 9

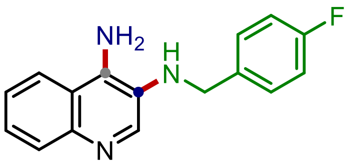

9

Prepared according to general procedure B (GP-B) using 3-azidoquinoline **Az-1** (80 mg, 0.47 mmol, 1.0 equiv.), (4-fluorophenyl)methanamine (118 µL, 1.03 mmol, 2.2 equiv.) and THF (1.30 mL). The obtained crude is purified on flash chromatography column (100% AcOEt + 1% TEA) to obtain the title compound **9** as a brown oil (65 mg, 52% yield). ^{1}H NMR (400 MHz, $CDCl_3$) δ 8.35 (s, 1H), 7.93 (dt, J = 8.6, 0.9 Hz, 1H), 7.72 (dt, J = 8.4, 1.1 Hz, 1H), 7.50 (ddd, J = 8.4, 6.8, 1.4 Hz, 1H), 7.40 (ddd, J = 8.2, 6.8, 1.3 Hz, 1H), 7.29 (dd, J = 8.5, 5.5 Hz, 2H), 7.05 – 6.94 (m, 2H), 4.90 (brs, 2H), 4.28 (s, 2H), 3.44 (bs, 1H). ^{13}C NMR (101 MHz, $CDCl_3$) δ 162.3 (d, J = 245.6 Hz), 145.1, 142.8, 139.7, 135.1 (d, J = 3.2 Hz), 129.9, 129.6 (d, J = 8.0 Hz), 127.3, 125.2, 125.1, 119.9, 118.7, 115.6 (d, J = 21.4 Hz), 49.9. MS

(ESI), m/z [M+H]$^+$: 268.32. HRMS (ESI), [M+H]+: Calculated for [$C_{16}H_{15}FN_3$]$^+$: 268.1245, found: 268.1243. (see spectra)

Synthesis of *N*^{3}-(4-methoxybenzyl)quinoline-3,4-diamine 10

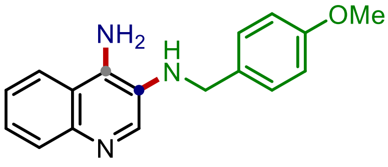

10

Prepared according to general procedure B (GP-B) using 3-azidoquinoline **Az-1** (34 mg, 0.2 mmol, 1.0 equiv.), (4-methoxyphenyl)methanamine (57 µL, 0.44 mmol, 2.2 equiv.) and THF (1.30 mL). The obtained crude is purified on flash chromatography column (100% AcOEt + 1% TEA) to obtain the title compound **10** as a yellow pale solid (32 mg, **57% yield**). ^{1}H NMR (400 MHz, $CDCl_3$) δ 8.39 (s, 1H), 7.93 (dd, J = 8.5, 1.4 Hz, 1H), 7.71 (dd, J = 8.5, 1.4 Hz, 1H), 7.50 (ddd, J = 8.4, 6.8, 1.4 Hz, 1H), 7.40 (ddd, J = 8.2, 6.8, 1.3 Hz, 1H), 7.27 (d, J = 8.6 Hz, 2H), 6.85 (d, J = 8.6 Hz, 2H), 4.83 (brs, 2H), 4.26 (s, 2H), 3.78 (s, 3H), 3.24 (s, 1H). ^{13}C NMR (101 MHz, $CDCl_3$) δ 159.2, 144.8, 142.4, 139.7, 131.5, 129.6, 129.3, 127.1, 125.5, 125.0, 119.9, 118.6, 114.2, 55.4, 50.1. MS (ESI), m/z [M+H]$^+$: 280.016. HRMS (ESI), [M+H]+: Calculated for [$C_{17}H_{18}N_3O$]$^+$: 280.1444, found: 280.1440. (see spectra)

Synthesis of *N*^{3}-allylquinoline-3,4-diamine 11

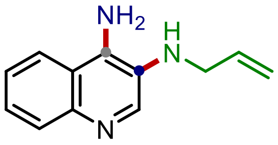

11

Prepared according to general procedure B (GP-B) using 3-azidoquinoline **Az-1** (34 mg, 0.2 mmol, 1.0 equiv.), prop-2-en-1-amine (33 µL, 0.44 mmol, 2.2 equiv.) and THF (1.30 mL). The obtained crude is purified on flash chromatography column (100% AcOEt + 1% TEA) to obtain the title compound **11** as a yellow oil (20 mg, **50% yield**). ^{1}H NMR (400 MHz, $CDCl_3$) δ 8.42 (s, 1H), 7.95 (dd, J = 8.5, 1.2 Hz, 1H), 7.71 (dd, J = 8.4, 1.4 Hz, 1H), 7.50 (ddd, J = 8.4, 6.7, 1.3 Hz, 1H), 7.40 (ddd, J = 8.1, 6.8, 1.3 Hz, 1H), 6.08 – 5.91 (m, 1H), 5.29 (dd, J = 17.2, 1.6 Hz, 1H), 5.16 (dd, J = 10.3, 1.5 Hz, 1H), 4.86 (brs, 2H), 3.79 (d, J = 4.8 Hz, 2H), 3.17 (brs, 1H). ^{13}C NMR (101 MHz, $CDCl_3$) δ 144.8, 142.5, 139.8, 135.7, 129.6, 127.1, 125.3, 125.0, 119.9, 118.6, 116.8, 48.8. MS (ESI), m/z [M+H]$^+$: 200.085. HRMS (ESI), [M+H]+: Calculated for [$C_{12}H_{14}N_3$]$^+$: 200.1182, found: 200.1178. (see spectra)

Synthesis of *N*^{3}-(prop-2-yn-1-yl)quinoline-3,4-diamine 12

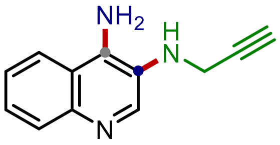

12

Prepared according to general procedure B (GP-B) using 3-azidoquinoline **Az-1** (34 mg, 0.2 mmol, 1.0 equiv.), prop-2-yn-1-amine (28 µL, 0.44 mmol, 2.2 equiv.) and THF (1.30 mL). The obtained crude is purified on flash chromatography column (100% AcOEt + 1% TEA) to obtain the title compound **12** as a yellow oil (15 mg, **37% yield**). ^{1}H NMR (400 MHz, $CDCl_3$) δ 8.55 (s, 1H), 7.97 (dd, J = 8.5, 1.2 Hz, 1H), 7.73 (dd, J = 8.3, 1.3 Hz, 1H), 7.56 (ddd, J = 8.0, 6.5, 1.2 Hz, 1H), 7.43 (ddd, J = 8.2, 6.9, 1.1 Hz, 1H), 4.89 (brs, 2H), 3.89 (d, J = 2.4 Hz, 2H), 3.16 (brs, 1H), 2.28 (t, J = 2.5 Hz, 1H). ^{13}C NMR (101 MHz, $CDCl_3$) δ 145.9, 145.1, 141.9, 129.8, 127.9, 125.1, 123.7, 120.2,

118.7, 81.4, 72.7, 36.5. MS (ESI), m/z [M+H]$^+$: 198.016. HRMS (ESI), [M+H]+: Calculated for [$C_{12}H_{12}N_3$]$^+$: 198.1026, found: 198.1023 (see spectra)

Synthesis of *N*[3],*N*[3]-dimethylquinoline-3,4-diamine 13

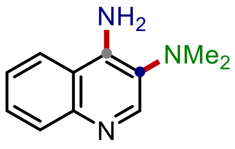

13

Prepared according to general procedure B (GP-B) using 3-azidoquinoline **Az-1** (34 mg, 0.2 mmol, 1.0 equiv.), dimethylamine (2.0 M in THF, 110 μL, 0.22 mmol, 1.1 equiv.) and THF (1.30 mL). The obtained crude is purified on flash chromatography column (100% AcOEt + 1% TEA) to obtain the title compound **13** as a yellow pale solid (30 mg, **80% yield**). ^{1}H NMR (400 MHz, $CDCl_3$) δ 8.59 (s, 1H), 7.95 (d, J = 8.5 Hz, 1H), 7.74 (d, J = 8.4 Hz, 1H), 7.55 (t, J = 7.7 Hz, 1H), 7.38 (t, J = 7.6 Hz, 1H), 5.24 (brs, 2H), 2.76 (s, 6H). ^{13}C NMR (101 MHz, $CDCl_3$) δ 146.0, 144.5, 144.0, 129.9, 129.4, 128.2, 124.7, 120.9, 118.5, 44.3. MS (ESI), m/z [M+H]$^+$: 189.037. HRMS (ESI), [M+H]$^+$: Calculated for [$C_{11}H_{14}N_3$]$^+$: 188.1182, found: 188.1178. (see spectra)

Synthesis of 3-(azetidin-1-yl)quinolin-4-amine 14

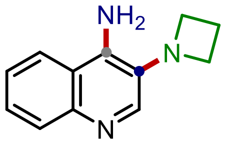

14

Prepared according to general procedure B (GP-B) using 3-azidoquinoline **Az-1** (34 mg, 0.2 mmol, 1.0 equiv.), azetidine (15 μL, 0.22 mmol, 1.1 equiv.) and THF (1.30 mL). The obtained crude is purified on flash chromatography column (100% AcOEt + 2% TEA) to obtain the title compound **14** as a yellow solid (32 mg, **80% yield**). ^{1}H NMR (400 MHz, $CDCl_3$) δ 8.37 (s, 1H), 7.95 (dd, J = 8.5, 1.3 Hz, 1H), 7.71 (dd, J = 8.4, 1.4 Hz, 1H), 7.50 (ddd, J = 8.3, 6.8, 1.4 Hz, 1H), 7.40 (ddd, J = 8.2, 6.7, 1.3 Hz, 1H), 4.68 (brs, 2H), 3.94 (t, J = 7.1 Hz, 4H), 2.35 (p, J = 7.1 Hz, 2H). ^{13}C NMR (101 MHz, $CDCl_3$) δ 144.8, 140.3, 139.5, 129.5, 129.1, 127.2, 125.1, 119.7, 118.5, 53.9, 18.2. MS (ESI), m/z [M+H]$^+$: 201.113. HRMS (ESI), [M+H]$^+$: Calculated for [$C_{12}H_{14}N_3$]$^+$: 200.1182, found: 200.1185. (see spectra)

Synthesis of 3-(pyrrolidin-1-yl)quinolin-4-amine 15

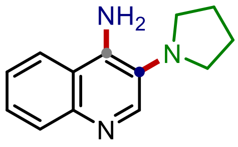

15

Prepared according to general procedure B (GP-B) using 3-azidoquinoline **Az-1** (34 mg, 0.2 mmol, 1.0 equiv.), pyrrolidine (18 μL, 0.22 mmol, 1.1 equiv.) and THF (1.30 mL). The obtained crude is purified on flash chromatography column (100% AcOEt + 1% TEA) to obtain the title compound **15** as a yellow solid (28 mg, **66% yield**). ^{1}H NMR (400 MHz, $CDCl_3$) δ 8.59 (s, 1H), 7.95 (dd, J = 8.6, 1.3 Hz, 1H), 7.75 (dd, J = 8.0, 1.1 Hz, 1H), 7.54 (ddd, J = 8.3, 6.8, 1.3 Hz, 1H), 7.38 (ddd, J = 8.3, 6.8, 1.2 Hz, 1H), 5.16 (brs, 2H), 3.20 – 3.03 (m, 4H), 2.03 – 1.86 (m, 4H). ^{13}C NMR (101 MHz, $CDCl_3$) δ 145.7, 144.4, 129.4, 128.0, 127.0, 124.8, 120.7, 118.5, 51.9, 24.5. One quaternary carbon overlaps with the tertiary carbon at 144.4 ppm. MS (ESI), m/z [M+H]$^+$: 214.60. HRMS (ESI), [M+H]$^+$: Calculated for [$C_{13}H_{16}N_3$]$^+$: 214.1339, found: 200.1343. (see spectra)

Synthesis of 3-(piperidin-1-yl)quinolin-4-amine 16

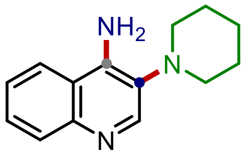

16

Prepared according to general procedure B (GP-B) using 3-azidoquinoline **Az-1** (34 mg, 0.2 mmol, 1.0 equiv.), piperidine (22 µL, 0.22 mmol, 1.1 equiv.) and THF (1.30 mL). The obtained crude is purified on flash chromatography column (100% AcOEt + 1% TEA) to obtain the title compound **16** as a yellow solid (33 mg, **72% yield**). ^{1}H NMR (400 MHz, $CDCl_3$) δ 8.57 (s, 1H), 7.95 (dd, J = 8.9, 1.1 Hz, 1H), 7.74 (dd, J = 8.5, 1.4 Hz, 1H), 7.55 (ddd, J = 8.4, 6.8, 1.4 Hz, 1H), 7.39 (ddd, J = 8.2, 6.8, 1.3 Hz, 1H), 5.19 (brs, 2H), 3.19 – 2.78 (m, 5H), 1.83 – 1.65 (m, 5H). ^{13}C NMR (101 MHz, $CDCl_3$) δ 146.1, 145.0, 144.0, 130.0, 129.6, 128.2, 124.7, 120.9, 118.6, 53.3, 27.2, 24.3. MS (ESI), m/z $[M+H]^+$: 229.18. HRMS (ESI), $[M+H]^+$: Calculated for $[C_{14}H_{18}N_3]^+$: 228.1495, found: 228.1496. (see spectra) *Some unidentified impurity could not be removed from this product even after a second purification on silica gel (direct phase or inverse phase).*

Synthesis of 3-(azepan-1-yl)quinolin-4-amine 17

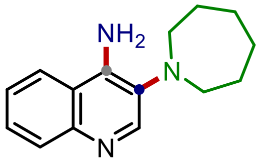

17

Prepared according to general procedure B (GP-B) using 3-azidoquinoline **Az-1** (34 mg, 0.2 mmol, 1.0 equiv.), azepane (25 µL, 0.22 mmol, 1.1 equiv.) and THF (1.30 mL), The obtained crude is purified on flash chromatography column (100% AcOEt + 1% TEA) to obtain the title compound **17** as a yellow oil (27 mg, **56% yield**). ^{1}H NMR (400 MHz, $CDCl_3$) δ 8.56 (s, 1H), 7.94 (dd, J = 8.6, 1.2 Hz, 1H), 7.76 (dd, J = 8.5, 1.4 Hz, 1H), 7.55 (ddd, J = 8.4, 6.8, 1.4 Hz, 1H), 7.39 (ddd, J = 8.2, 6.8, 1.3 Hz, 1H), 5.32 (brs, 2H), 3.23 – 3.01 (m, 4H), 1.87 – 1.63 (m, 8H). ^{13}C NMR (101 MHz, $CDCl_3$) δ 146.7, 145.7, 144.4, 132.1, 129.2, 128.3, 124.8, 121.1, 118.6, 56.3, 30.3, 27.2. MS (ESI), m/z $[M+H]^+$: 242.452. HRMS (ESI), $[M+H]^+$: Calculated for $[C_{15}H_{20}N_3]^+$: 242.1652, found: 242.1655. (see spectra) *Some unidentified impurity could not be removed from this product even after a second purification on silica gel (direct phase or inverse phase).*

Synthesis of 3-morpholinoquinolin-4-amine 18

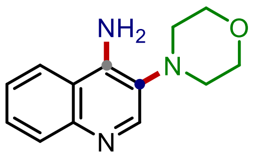

18

Prepared according to general procedure B (GP-B) using 3-azidoquinoline **Az-1** (34 mg, 0.2 mmol, 1.0 equiv.), morpholine (19 µL, 0.22 mmol, 1.1 equiv.) and THF (1.30 mL). The obtained crude is purified on flash chromatography column (100% AcOEt + 1% TEA) to obtain the title compound **18** as a pale yellow solid (31 mg, **68% yield**). ^{1}H NMR (400 MHz, $CDCl_3$) δ 8.58 (s, 1H), 7.96 (dd, J = 8.5, 1.2 Hz, 1H), 7.76 (dd, J = 8.6, 1.4 Hz, 1H), 7.57 (ddd, J = 8.4, 6.8, 1.4 Hz, 1H), 7.40 (ddd, J = 8.2, 6.8, 1.3 Hz, 1H), 5.29 (brs, 2H), 3.96 – 3.75 (m, 4H), 3.16 – 2.84 (m, 4H). ^{13}C NMR (101 MHz, $CDCl_3$) δ 146.3, 144.8, 144.5, 129.6, 128.6, 128.1, 124.9, 121.0, 118.5, 67.9, 52.1. MS (ESI), m/z $[M+H]^+$: 230.246. HRMS (ESI), $[M+H]^+$: Calculated for $[C_{13}H_{16}N_3O]^+$: 230.1288, found: 230.1285. (see spectra) *Some unidentified impurity could not be removed from this product even after a second purification on silica gel (direct phase or inverse phase).*

Synthesis of 3-thiomorpholinoquinolin-4-amine 19

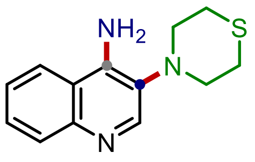

19

Prepared according to general procedure B (GP-B) using 3-azidoquinoline **Az-1** (34 mg, 0.2 mmol, 1.0 equiv.), thiomorpholine (21 µL, 0.22 mmol, 1.1 equiv.) and THF (1.30 mL). The obtained crude is purified on flash chromatography column (100% AcOEt) to obtain the title compound **19** as a pae yellow solid (22 mg, **45% yield**). ^{1}H NMR (400 MHz, $CDCl_3$) δ 8.56 (s, 1H), 7.96 (dd, J = 8.5, 1.2 Hz, 1H), 7.74 (dd, J = 8.4, 1.4 Hz, 1H), 7.59 (ddd, J = 8.4, 6.8, 1.4 Hz, 1H), 7.42 (ddd, J = 8.2, 6.8, 1.2 Hz, 1H), 5.21 (brs, 2H), 3.24 (t, J = 4.9 Hz, 4H), 3.20 – 2.50 (m, 4H). ^{13}C NMR (101 MHz, $CDCl_3$) δ 146.2, 145.2, 144.3, 129.5, 128.7, 125.0, 121.0, 118.5, 54.2, 29.2. One quaternary carbon overlaps with the tertiary carbon at 129.5 ppm. MS (ESI), m/z $[M+H]^+$: 247.205. HRMS (ESI), $[M+H]^+$: Calculated for $[C_{13}H_{16}N_3S]^+$: 246.1059, found: 246.1054. (see spectra)

Synthesis of 3-(4-ethylpiperazin-1-yl)quinolin-4-amine 20

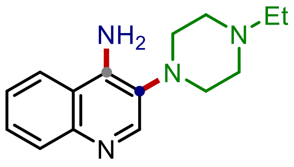

20

Prepared according to general procedure B (GP-B) using 3-azidoquinoline **Az-1** (34 mg, 0.2 mmol, 1.0 equiv.), 1-ethylpiperazine (28 µL, 0.22 mmol, 1.1 equiv.) and THF (1.30 mL). The obtained crude is purified on flash chromatography column (DCM:MeOH 95:5 + 1% TEA) to obtain the title compound **20** as a pale yellow solid (46 mg, **90% yield**). ^{1}H NMR (400 MHz, $CDCl_3$) δ 8.52 (s, 1H), 7.92 (d, *J* = 8.5 Hz, 1H), 7.85 (d, *J* = 8.4 Hz, 1H), 7.53 (t, *J* = 7.6 Hz, 1H), 7.36 (t, *J* = 7.6 Hz, 1H), 5.59 (brs, 2H), 3.01 (t, *J* = 4.9 Hz, 4H), 2.83 – 2.20 (m, 6H), 1.10 (t, *J* = 7.2 Hz, 3H). ^{13}C NMR (101 MHz, $CDCl_3$) δ 145.4, 145.3, 143.8, 128.8, 128.5, 128.3, 124.9, 121.5, 118.2, 53.7, 52.4, 51.6, 12.1. MS (ESI), m/z $[M+H]^+$: 257.460. HRMS (ESI), $[M+H]^+$: Calculated for $[C_{15}H_{21}N_4]^+$: 257.1761, found: 257.1756. (see spectra) *Some unidentified impurity could not be removed from this product even after a second purification on silica gel (direct phase or inverse phase).*

Synthesis of 3-(4-phenylpiperazin-1-yl)quinolin-4-amine 21

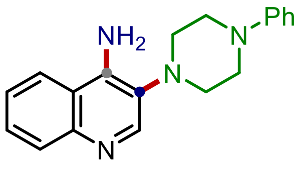

21

Prepared according to general procedure B (GP-B) using 3-azidoquinoline **Az-1** (34 mg, 0.2 mmol, 1.0 equiv.), 1-phenylpiperazine (35 µL, 0.22 mmol, 1.1 equiv.) and THF (1.30 mL). The obtained crude is purified on flash chromatography column (100% AcOEt + 1% TEA) to obtain the title compound **21** as a pale yellow solid (33 mg, **54% yield**). ^{1}H NMR (400 MHz, $CDCl_3$) δ 8.65 (s, 1H), 7.99 (dd, J = 8.5, 1.2 Hz, 1H), 7.77 (dd, J = 8.5, 1.4 Hz, 1H), 7.60 (ddd, J = 8.4, 6.8, 1.3 Hz, 1H), 7.43 (ddd, J = 8.2, 6.8, 1.3 Hz, 1H), 7.31 (dd, J = 8.7, 7.2 Hz, 2H), 7.00 (dd, J = 8.8, 1.1 Hz, 2H), 6.91 (tt, J = 7.3, 1.0 Hz, 1H), 5.26 (brs, 2H), 3.36 (s, 4H), 3.19 (t, J = 4.7 Hz, 4H). ^{13}C NMR (101 MHz, $CDCl_3$) δ 151.4, 146.3, 144.7, 144.4, 129.5, 129.3, 128.6, 128.3, 124.9, 120.9, 120.2, 118.4, 116.4, 51.8, 50.4. MS (ESI), m/z $[M+H]^+$: 306.31. HRMS (ESI), $[M+H]^+$: Calculated for $[C_{19}H_{21}N_4]^+$: 305.1761, found: 305.1757. (see spectra)

Synthesis of *tert*-butyl 4-(4-aminoquinolin-3-yl)piperazine-1-carboxylate 22

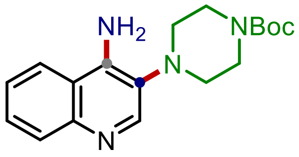

22

Prepared according to general procedure B (GP-B) using 3-azidoquinoline **Az-1** (34 mg, 0.2 mmol, 1.0 equiv.), *tert*-butyl piperazine-1-carboxylate (41 mg, 0.22 mmol, 1.1 equiv.) and THF (1.30 mL). The obtained crude is purified on flash chromatography column (100% AcOEt + 1% TEA) to obtain the title compound **22** as a white solid (31 mg, **47% yield**). ^{1}H NMR (400 MHz, $CDCl_3$) δ 8.54 (s, 1H), 7.95 (dd, J = 8.5, 1.2 Hz, 1H), 7.75 (dd, J = 8.9, 1.1 Hz, 1H), 7.58 (ddd, J = 8.4, 6.8, 1.3 Hz, 1H), 7.41 (ddd, J = 8.2, 6.8, 1.2 Hz, 1H), 5.25 (brs, 2H), 4.03 – 3.10 (m, 4H), 2.96 (t, J = 5.0 Hz, 4H), 1.49 (s, 9H). ^{13}C NMR (101 MHz, $CDCl_3$) δ 154.9, 146.3, 144.7, 144.3, 129.6, 128.7, 128.2, 125.0, 120.9, 118.5, 80.1, 51.7, 29.8, 28.5. MS (ESI), m/z $[M+H]^+$: 329.342. HRMS (ESI), $[M+H]^+$: Calculated for $[C_{18}H_{25}N_4O_2]^+$: 329.1972, found: 329.1970. (see spectra)

Synthesis of ethyl 1-(4-aminoquinolin-3-yl)piperidine-3-carboxylate 23

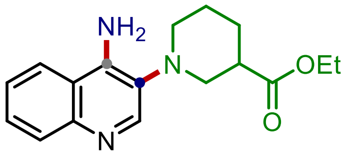

23

Prepared according to general procedure B (GP-B) using 3-azidoquinoline **Az-1** (34 mg, 0.2 mmol, 1.0 equiv.), ethyl piperidine-3-carboxylate (34 µL, 0.22 mmol, 1.1 equiv.) and THF (1.30 mL). The obtained crude is purified on flash chromatography column (100% AcOEt + 1% TEA) to obtain the title compound **23** as a yellow solid (28 mg, **45% yield**). ^{1}H NMR (400 MHz, $CDCl_3$) δ 8.53 (s, 1H), 7.95 (dd, J = 8.5, 1.2 Hz, 1H), 7.75 (dd, J = 8.5, 1.4 Hz, 1H), 7.56 (ddd, J = 8.4, 6.8, 1.3 Hz, 1H), 7.39 (ddd, J = 8.2, 6.8, 1.2 Hz, 1H), 5.60 (brs, 2H), 4.19 (q, J = 6.6 Hz, 2H), 3.20 – 3.05 (m, 1H), 3.04 – 2.74 (m, 2H), 2.77 – 2.63 (m, 1H), 1.88 – 1.63 (m, 3H), 1.28 (t, J = 7.1 Hz, 3H), 1.24 (s, 1H). ^{13}C NMR (101 MHz, $CDCl_3$) δ 174.6, 146.1, 144.6, 144.4, 129.4, 128.7, 128.4, 124.7, 120.8, 118.4, 62.3, 60.8, 53.8, 52.4, 29.8, 26.4, 14.4. MS (ESI), m/z $[M+H]^+$: 301.235. HRMS (ESI), $[M+H]^+$: Calculated for $[C_{17}H_{22}N_3O_2]^+$: 300.1707, found: 300.1704. (see spectra) *Some unidentified impurity could not be removed from this product even after a second purification on silica gel (direct phase or inverse phase).*

Synthesis of (1-(4-aminoquinolin-3-yl)piperidin-3-yl)methanol 24

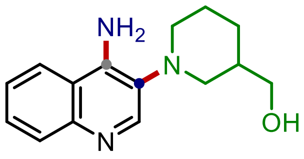

24

Prepared according to general procedure B (GP-B) using 3-azidoquinoline **Az-1** (34 mg, 0.2 mmol, 1.0 equiv.), piperidin-3-ylmethanol (25 µL, 0.22 mmol, 1.1 equiv.) and THF (1.30 mL). The obtained crude is purified on flash chromatography column (DCM:MeOH 95:5 + 1% TEA) to obtain the title compound **24** as a yellow solid (28 mg, **54% yield**). ^{1}H NMR (400 MHz, MeOD) δ 8.36 (s, 1H), 8.09 (d, J = 8.4 Hz, 1H), 7.78 (d, J = 8.5 Hz, 1H), 7.61 (ddd, J = 8.3, 6.6, 1.3 Hz, 1H), 7.43 (t, J = 7.6 Hz, 1H), 3.53 (s, 2H), 3.16 (dd, J = 9.4, 4.9 Hz, 1H), 3.02 (d, J = 11.1 Hz, 1H), 2.84 – 2.69 (m, 1H), 2.59 (s, 1H), 2.06 – 1.92 (m, 1H), 1.88 – 1.66 (m, 3H), 1.23 – 1.08 (m, 1H). ^{13}C NMR (101 MHz, MeOD) δ 149.0, 145.2, 143.2, 130.5, 130.3, 127.2, 125.7, 123.6, 119.1, 65.9,

63.5, 57.0, 54.1, 30.7, 28.0. MS (ESI), m/z $[M+H]^+$: 258.744. HRMS (ESI), $[M+H]^+$: Calculated for $[C_{15}H_{20}N_3O]^+$: 258.1601, found: 258.1597. (see spectra)

Synthesis of 3-(3,4-dihydroisoquinolin-2(1*H*)-yl)quinolin-4-amine 25

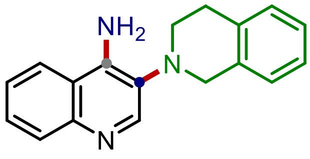

25

Prepared according to general procedure B (GP-B) using 3-azidoquinoline **Az-1** (34 mg, 0.2 mmol, 1.0 equiv.), 1,2,3,4-tetrahydroisoquinoline (28 µL, 0.22 mmol, 1.1 equiv.) and THF (1.30 mL). The obtained crude is purified on flash chromatography column (Hex:AcOEt 3:7 + 1% TEA) to obtain the title compound **25** as a pale yellow solid (40 mg, **72% yield**). ^1H NMR (400 MHz, $CDCl_3$) δ 8.63 (s, 1H), 7.99 (dd, J = 8.5, 1.2 Hz, 1H), 7.78 (dd, J = 8.5, 1.4 Hz, 1H), 7.60 (ddd, J = 8.3, 6.8, 1.3 Hz, 1H), 7.43 (ddd, J = 8.2, 6.8, 1.2 Hz, 1H), 7.25 – 7.13 (m, 3H), 7.07 (d, J = 6.3 Hz, 1H), 5.32 (brs, 2H), 4.18 (s, 2H), 3.37 (t, J = 5.8 Hz, 2H), 3.17 – 2.91 (m, 2H). ^{13}C NMR (101 MHz, $CDCl_3$) δ 146.2, 145.0, 144.6, 135.1, 134.1, 129.5, 129.2, 128.6, 128.5, 126.6, 126.5, 126.0, 124.9, 121.0, 118.5, 54.6, 49.9, 30.1. MS (ESI), m/z $[M+H]^+$: 276.642. HRMS (ESI), $[M+H]^+$: Calculated for $[C_{18}H_{18}N_3]^+$: 276.1495, found: 276.1492. (see spectra)

Synthesis of *N*³,*N*³-diethyl-6-methoxyquinoline-3,4-diamine 26

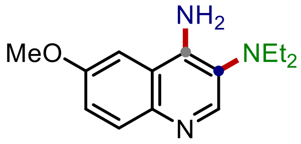

26

Prepared according to general procedure B (GP-B) using 3-azido-6-methoxyquinoline **Az-2** (40 mg, 0.2 mmol, 1.0 equiv.), diethylamine (23 µL, 0.22 mmol, 1.1 equiv.) and THF (1.30 mL). The obtained crude is purified on flash chromatography column (Hex:AcOEt 3:7 to 100% AcOEt + 1% TEA) to obtain the title compound **26** as a pale yellow solid (21 mg, **43% yield**). ^1H NMR (400 MHz, $CDCl_3$) δ 8.44 (s, 1H), 7.88 (d, J = 9.2 Hz, 1H), 7.25 (dd, J = 9.2, 2.7 Hz, 1H), 6.95 (d, J = 2.7 Hz, 1H), 5.12 (brs, 2H), 3.88 (s, 3H), 3.07 (q, J = 7.1 Hz, 4H), 1.00 (t, J = 7.1 Hz, 6H). ^{13}C NMR (101 MHz, $CDCl_3$) δ 156.9, 146.1, 145.3, 142.4, 131.4, 126.6, 120.5, 119.3, 99.8, 55.6, 49.1, 13.1. MS (ESI), m/z $[M+H]^+$: 246.678. HRMS (ESI), $[M+H]^+$: Calculated for $[C_{14}H_{20}N_3O]^+$: 246.1601, found: 246.1595. (see spectra)

Synthesis of 6-bromo-*N*³,*N*³-diethyl-7-fluoroquinoline-3,4-diamine 27

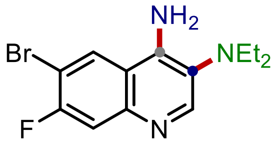

27

Prepared according to general procedure B (GP-B) using 3-azido-6-bromo-7-fluoroquinoline **Az-3** (54 mg, 0.2 mmol. 1.0 equiv.) diethylamine (23 µL, 0.22 mmol, 1.1 equiv.) and THF (1.30 mL). The obtained crude is purified on flash chromatography column (Hex:AcOEt 7:3) to obtain the title compound **27** as a pale yellow solid (29 mg, **46% yield**). ^1H NMR (400 MHz, $CDCl_3$) δ 8.52 (s, 1H), 7.96 (d, J = 7.1 Hz, 1H), 7.64 (d, J = 9.9 Hz, 1H), 5.25 (brs, 2H), 3.06 (q, J = 7.1 Hz, 4H), 1.00 (t, J = 7.1 Hz, 6H). ^{13}C NMR (101 MHz, $CDCl_3$) δ 158.5 (d, J = 249.4 Hz), 149.0, 146.5 (d, J = 10.1 Hz), 131.4, 126.5, 126.2 (d, J = 2.0 Hz), 116.7 (d, J = 1.7 Hz), 114.5 (d, J = 21.2 Hz), 107.8 (d, J = 24.1 Hz), 49.1, 13,1. ^{19}F NMR (376 MHz, $CDCl_3$) δ −107.3. MS (ESI), m/z $[M+H]^+$: 312.762. HRMS (ESI), $[M+H]^+$: Calculated for $[C_{13}H_{16}BrFN_3]^+$: 312.0506, found: 312.0501. (see spectra) *Some unidentified impurity*

could not be removed from this product even after a second purification on silica gel (direct phase or inverse phase).

Synthesis of 7-chloro-*N*3,*N*3-diethylquinoline-3,4-diamine 28

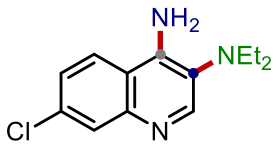

28

Prepared according to general procedure B (GP-B) using 3-azido-7-chloroquinoline **Az-4** (41 mg, 0.20 mmol, 1.0 equiv.), diethylamine (23 µL, 0.22 mmol, 1.1 equiv.) and THF (1.30 mL). The obtained crude is purified on flash chromatography column (Hex:AcOEt) to obtain title compound **28** (37 mg, 0.15 mmol, **75% yield**). ^{1}H NMR (400 MHz, $CDCl_3$) δ 8.49 (s, 1H), 7.95 (d, *J* = 2.1 Hz, 1H), 7.72 (d, *J* = 8.9 Hz, 1H), 7.34 (dd, *J* = 8.9, 2.0 Hz, 1H), 5.46 (brs, 2H), 3.05 (q, *J* = 7.1 Hz, 4H), 1.00 (t, *J* = 7.1 Hz, 6H). ^{13}C NMR (101 MHz, $CDCl_3$) δ 147.8, 147.8, 146.5, 134.6, 128.1, 126.4, 125.6, 122.8, 118.0, 49.0, 13.1. MS (ESI), m/z $[M+H]^+$: 250.49. HRMS (ESI), $[M+H]^+$: Calculated for $[C_{13}H_{16}ClN_3]^+$: 249.1033 found: 249.1035 (see spectra)

Synthesis of 7-chloro-*N*3-(5-(diethylamino)pentan-2-yl)quinoline-3,4-diamine 29

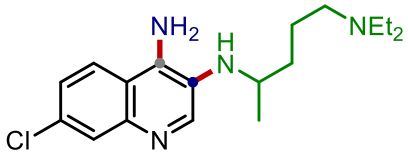

29

Prepared according to general procedure B (GP-B) using 3-azido-7-chloroquinoline **Az-4** (82 mg, 0.40 mmol, 1.0 equiv.), *N*1,*N*1-diethylpentane-1,4-diamine (139 mg, 0.88 mmol, 2.2 equiv.) and THF (2.50 mL). The obtained crude is purified on flash chromatography column (Hex:AcOEt + 1% TEA) to obtain title compound **29** (65 mg, 0.19 mmol, **49% yield**).). ^{1}H NMR (400 MHz, $CDCl_3$) δ 8.34 (s, 1H), 7.87 (d, *J* = 2.2 Hz, 1H), 7.63 (d, *J* = 9.0 Hz, 1H), 7.28 (dd, *J* = 9.2, 2.3 Hz, 1H), 5.06 (bs, 2H), 3.38 (q, *J* = 6.0 Hz, 1H), 2.58 (q, *J* = 7.2 Hz, 4H), 2.48 (td, *J* = 6.5, 4.8 Hz, 2H), 1.72 – 1.53 (m, 3H), 1.46 (ddd, *J* = 8.3, 6.8, 4.1 Hz, 1H), 1.13 (d, *J* = 6.2 Hz, 3H), 1.02 (t, *J* = 7.2 Hz, 6H). ^{13}C NMR (101 MHz, $CDCl_3$) δ 145.1, 144.5, 140.7, 132.6, 128.2, 125.5, 124.8, 121.7, 117.1, 52.9, 50.8, 46.9, 35.3, 23.6, 21.2, 11.1. MS (ESI), m/z $[M+H]^+$: 335.41. HRMS (ESI), $[M+H]^+$: Calculated for $[C_{18}H_{27}ClN_4]^+$: 334.1924, found: 334.1927. (see spectra)

Synthesis of *N*3,*N*3-diethyl-5-methylquinoline-3,4-diamine 30

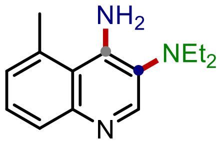

30

Prepared according to general procedure B (GP-B) using 3-azido-5-methylquinoline **Az-5** (37 mg, 0.20 mmol, 1.0 equiv.), diethylamine (23 µL, 0.22 mmol, 1.1 equiv.) and THF (1.30 mL). The obtained crude is purified on flash chromatography column (Hex:AcOEt) to obtain title compound **30** (15 mg, 0.061 mmol, **32% yield**). ^{1}H NMR (400 MHz, $CDCl_3$) δ 8.44 (s, 1H), 7.81 (d, *J* = 9.1 Hz, 1H), 7.41 (dd, *J* = 8.5, 7.0 Hz, 1H), 7.12 (dt, *J* = 7.0, 1.2 Hz, 1H), 5.76 (brs, 2H), 3.05 (q, *J* = 7.1 Hz, 4H), 2.96 (s, 3H), 1.02 (t, *J* = 7.1 Hz, 6H). ^{13}C NMR (101 MHz, $CDCl_3$) δ 147.9, 145.9, 142.6, 128.3, 128.1, 127.7, 126.7, 125.4, 118.8, 49.1, 24.6, 13.1. MS (ESI), m/z $[M+H]^+$: 231.15. HRMS (ESI), $[M+H]^+$: Calculated for $[C_{14}H_{19}N_3]^+$: 229.1579 found: 229.1577 (see spectra)

Synthesis of N^3,N^3-diethyl-6-(trifluoromethyl)quinoline-3,4-diamine 31

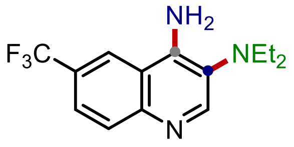

31

Prepared according to general procedure B (GP-B) using 3-azido-6-(trifluoromethyl)quinoline **Az-6** (26 mg, 0.10 mmol, 1.0 equiv.), diethylamine (11 µL, 0.11 mmol, 1.1 equiv.) and THF (0.7 mL). The obtained crude is purified on flash chromatography column (Hex:AcOEt) to obtain title compound **31** (6 mg, 0.020 mmol **20% yield**). ^{1}H NMR (400 MHz, $CDCl_3$) δ 8.60 (s, 1H), 8.10 (t, *J* = 4.4 Hz, 2H), 7.76 (dd, *J* = 8.9, 1.9 Hz, 1H), 5.56 (brs, 2H), 3.09 (q, *J* = 7.1 Hz, 4H), 1.02 (t, *J* = 7.1 Hz, 6H). ^{13}C NMR (101 MHz, $CDCl_3$) δ 148.6, 146.9, 135.9, 130.3, 129.7, 128.0, 124.5, 119.6 (q, *J* = 4.4 Hz), 117.4, 116.7 (q, *J* = 305.2 Hz), 49.0, 13.0. MS (ESI), m/z $[M+H]^+$: 284.54. HRMS (ESI), $[M+H]^+$: Calculated for $[C_{14}H_{16}F_3N_3]^+$: 283.1296 found: 283.1295 (see spectra) *Some unidentified impurity could not be removed from this product even after a second purification on silica gel (direct phase or inverse phase).*

Synthesis of *N,N*-diethyl-7-phenyl-5*H*-1,3-diazepin-4-amine 32

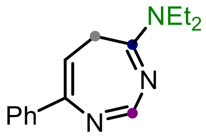

32

Prepared according to general procedure B (GP-B) using 5-azido-2-phenylpyridine **Az-7** (19.5 mg, 0.1 mmol, 1.0 equiv.), diethylamine (23 µL, 0.22 mmol, 2.2 equiv.) and THF (0.65 mL). The obtained crude is purified on flash chromatography column (100% AcOEt + 1% TEA) to obtain the title compound **32** as a brown solid (14 mg, **58% yield**). ^{1}H NMR (400 MHz, $CDCl_3$) δ 8.09 (s, 1H), 7.67 (d, J = 7.5 Hz, 2H), 7.42 – 7.29 (m, 2H), 7.29 – 7.21 (m, 1H), 5.40 (t, J = 7.4 Hz, 1H), 3.52 – 3.29 (m, 4H), 2.85 (brs, 1H), 2.12 (brs, 1H), 1.24 (t, J = 8.3 Hz, 3H), 1.09 (t, J = 7.2 Hz, 3H).

Note: At room temperature, the broad signals at 2.85 ppm and 2.12 ppm are caused by the tautomeric equilibrium between 5*H*- and 1*H*-1,3-diazepine.

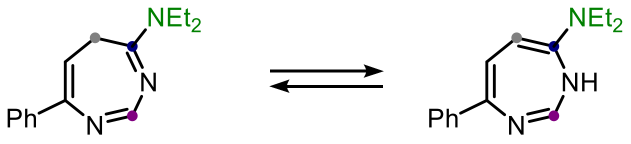

5*H*-1,3-diazepine 1*H*-1,3-diazepine

The equilibrium is frozen at low temperature, as confirmed by running the ^{1}H NMR in CD_2Cl_2 at −40 °C, in agreement with those reported in the literature.[11] ^{1}H NMR (400 MHz, CD_2Cl_2, −40 °C) δ 8.01 (s, 1H), 7.67 (d, J = 7.1 Hz, 2H), 7.33 (t, J = 7.5 Hz, 2H), 7.30 – 7.23 (m, 1H), 5.44 (t, J = 7.4 Hz, 1H), 3.48 – 3.32 (m, 4H), 2.91 (brs, 2H), 1.24 (t, J = 7.2 Hz, 3H), 1.08 (t, J = 7.0 Hz, 3H) (see spectra).

Synthesis of methyl 3-(diethylamino)-2*H*-1,4-diazepine-6-carboxylate 33

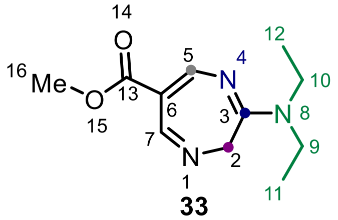

33

Prepared according to general procedure B (GP-B) using 5-azidonicotinate **Az-8** (36 mg, 0.2 mmol, 1.0 equiv.), diethylamine (46 µL, 0.44 mmol, 2.2 equiv.) and THF (1.30 mL). The obtained crude is purified on flash chromatography column (100% AcOEt + 1% TEA) to obtain the title compound **33** as a brown solid (5 mg, **11% yield**). ^{1}H NMR (400 MHz, $CDCl_3$) δ 8.49 (d, J = 1.4 Hz, 1H, H7), 8.27 (d, J = 1.8 Hz, 1H, H5), 3.80 (s, 3H, H16), 3.50 (s, 4H, H-9, H10), 1.34 (t, J = 7.2 Hz, 3H, H11-H12), 1.08 (t, J = 7.1 Hz, 3H, H11-H12). ^{13}C NMR (101 MHz, $CDCl_3$) δ 168.5 (C13), 161.9 (C7), 154.0 (C5), 148.3 (C3), 110.8 (C6), 53.7 (C2), 51.5 (C16), 44.2 (C9), 43.9 (C10), 14.4 (C11), 12.6 (C12). ^{13}C-^{1}H HMBC experiment show cross peaks relatives to H5-C6, H5-C13 and H7-C2, confirming the 1,4-diazepine structure. HRMS (ESI), $[M+H]^+$: Calculated for $[C_{11}H_{17}N_3O_2]^+$: 223.1321, found: 223.1218. (see spectra)

Note: At room temperature, the protons in position 2 are not observed due to the tautomeric equilibrium between 2*H*- and 4*H*-1,4-diazepine:

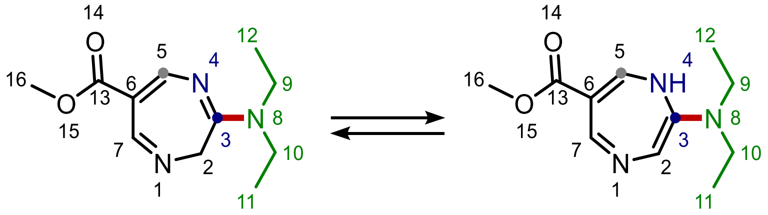

2*H*-1,4-diazepine 4*H*-1,4-diazepine

The equilibrium is frozen at low temperature, as confirmed by running the ^{1}H NMR in CD_2Cl_2 at -40 °C.[11] (see spectrum). ^{1}H NMR (400 MHz, CD_2Cl_2, −40 °C) δ 8.36 (d, J = 2.2 Hz, 1H, H7), 8.15 (d, J = 3.9 Hz, 1H, H5), 5.24 (d, J = 10.1 Hz, 1H, H2), 3.72 (s, 3H, H16), 3.67 (dd, J = 13.4, 6.7 Hz, 1H, H9- H10), 3.53 (dt, J = 14.6, 7.3 Hz, 1H, H9- H10), 3.35 (dt, J = 14.9, 7.3 Hz, 1H, H9- H10), 3.23 (dt, J = 14.0, 7.0 Hz, 1H, H9- H10), 2.76 (d, J = 9.6 Hz, 1H, H2), 1.26 (td, J = 7.1, 1.7 Hz, 3H, H11-H12), 1.00 (td, J = 7.1, 1.7 Hz, 3H, H11-H12).

Synthesis of 3-(4-(2,3,4-trimethoxybenzyl)piperazin-1-yl)quinolin-4-amine 34

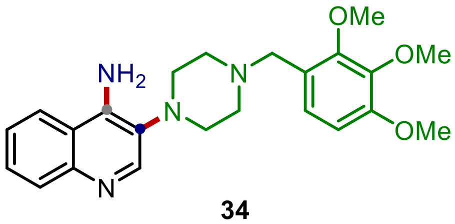

34

Prepared according to general procedure B (GP-B) using 3-azidoquinoline **Az-1** (34 mg, 0.2 mmol, 1.0 equiv.), trimetazidine (60 mg, 0.22 mmol, 1.1 equiv.) and THF (1.30 mL). The obtained crude is purified on flash chromatography column (DCM:MeOH 95:5 + 1% TEA) to obtain the title compound **34** as a pale yellow solid (46 mg, **56% yield**). ^{1}H NMR (400 MHz, $CDCl_3$) δ 8.50 (s, 1H), 7.98 (d, J = 8.5 Hz, 1H), 7.91 (d, J = 8.4 Hz, 1H), 7.58 (ddd, J = 8.3, 6.9, 1.2 Hz, 1H), 7.41 (ddd, J = 8.1, 6.8, 1.1 Hz, 1H), 7.04 (d, J = 8.5 Hz, 1H), 6.67 (d, J = 8.5 Hz, 1H), 5.80 (brs, 2H), 3.92 (s, 3H), 3.89 (s, 3H), 3.86 (s, 3H), 3.58 (s, 2H), 3.08 – 2.95 (m, 4H), 2.90 – 2.34 (m, 4H). ^{13}C NMR (101 MHz,

$CDCl_3$) δ 153.2, 152.8, 146.4, 144.1, 142.5, 129.5, 128.5, 127.6, 125.3, 125.2, 123.7, 121.7, 117.9, 107.2, 61.4, 61.0, 56.7, 56.1, 53.8, 51.9. One quaternary carbon overlaps with the tertiary carbon at 125.3 ppm. MS (ESI), m/z $[M+H]^+$: 410.026. HRMS (ESI), $[M+H]^+$: Calculated for $[C_{23}H_{29}N_4O_3]^+$: 409.2234, found: 409.2228. (see spectra)

Synthesis of (1*S*,2*R*)-2-((4-aminoquinolin-3-yl)(methyl)amino)-1-phenylpropan-1-ol 35

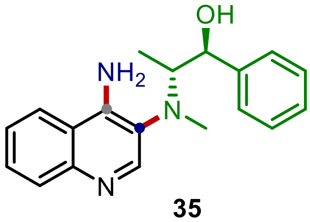

35

Prepared according to general procedure B (GP-B) using 3-azidoquinoline **Az-1** (34 mg, 0.2 mmol, 1.0 equiv.), *L*-ephedrine (36 mg, 0.22 mmol, 1.1 equiv.) and THF (1.30 mL). The obtained crude is purified on flash chromatography column (100% AcOEt + 1% TEA) to obtain the title compound **35** as a withe solid (17 mg, **29% yield**). 1H NMR (400 MHz, CDCl3) δ 8.53 (s, 1H), 7.94 (d, J = 8.4 Hz, 1H), 7.69 (d, J = 8.4 Hz, 1H), 7.56 (t, J = 7.6 Hz, 1H), 7.43 – 7.29 (m, 5H), 7.31 – 7.26 (m, 1H), 5.14 (brs, 2H), 4.90 (brs, 1H), 3.40 – 3.06 (m, 2H), 2.80 (s, 3H), 1.18 – 1.04 (m, 3H). ^{13}C NMR (101 MHz, $CDCl_3$) δ 146.7, 146.4, 145.3, 143.0, 129.0, 128.7, 128.4, 128.4, 128.1, 127.6, 126.3, 125.0, 121.1, 118.4, 74.7, 64.3, 35.6, 11.2. MS (ESI), m/z $[M+H]^+$: 308.475. HRMS (ESI), $[M+H]^+$: Calculated for $[C_{19}H_{22}N_3O]^+$: 308.1757, found: 308.1752. (see spectra) *Some unidentified impurity could not be removed from this product even after a second purification on silica gel (direct phase or inverse phase).*

Synthesis of 3-(4-(8-chloro-5,6-dihydro-11*H*-benzo[5,6]cyclohepta[1,2-*b*]pyridin-11-ylidene)piperidin-1-yl)quinolin-4-amine 36

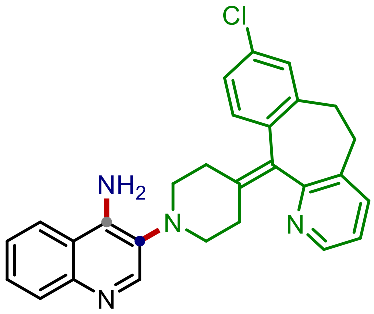

36

Prepared according to general procedure B (GP-B) using 3-azidoquinoline **Az-1** (34 mg, 0.2 mmol, 1.0 equiv.), desloratadine (68 mg, 0.22 mmol, 1.1 equiv.) and THF (1.30 mL). The obtained crude is purified on flash chromatography column (100% AcOEt + 1% TEA) to obtain the title compound **36** as a pink solid (53 mg, **57% yield**). ^1H NMR (400 MHz, $CDCl_3$) δ 8.51 (s, 1H), 8.41 (dd, J = 4.8, 1.6 Hz, 1H), 7.94 (d, J = 8.5 Hz, 1H), 7.78 (d, J = 8.4 Hz, 1H), 7.55 (ddd, J = 8.2, 6.8, 1.2 Hz, 1H), 7.44 (dd, J = 7.7, 1.7 Hz, 1H), 7.38 (ddd, J = 8.4, 6.7 Hz, 1.2 Hz 1H), 7.21 – 7.13 (m, 3H), 7.12 – 7.06 (m, 1H), 5.41 (brs, 2H), 3.55 – 3.36 (m, 2H), 3.21 – 3.05 (m, 2H), 2.93 – 2.75 (m, 4H), 2.75 – 2.62 (m, 1H), 2.62 – 2.42 (m, 3H). ^{13}C NMR (101 MHz, $CDCl_3$) δ 157.4, 146.7, 145.9, 144.5, 144.3, 139.7, 137.8, 137.6, 137.5, 133.9, 133.6, 132.9, 130.8, 129.2, 129.1, 128.7, 128.5, 126.2, 124.8, 122.4, 121.1, 118.4, 53.5 (2), 32.2, 32.1, 31.9, 31.6. MS (ESI), m/z $[M+H]^+$: 453.56. HRMS (ESI), $[M+H]^+$: Calculated for $[C_{28}H_{26}ClN_4]^+$: 453.1841, found: 453.1837. (see spectra) *Some unidentified impurity could not be removed from this product even after a second purification on silica gel (direct phase or inverse phase).*

2.4 Isotopic labelling experiments

2.4.1 Determination of nitrogen NMR resonances before isotopic labeling

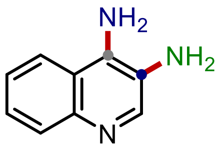

2

Unlabeled compound **2** (in DMSO-d_6) was analyzed by ^{15}N NMR experiments. The natural abundance of ^{15}N was insufficient for direct ^{15}N NMR analysis, but enough for a ^{15}N-^{1}H HMBC experiment exploiting the abundance of the proton. Three cross-peaks were identified between the C2-proton of the quinoline (8.2 ppm) and the three nitrogen atoms, with intensities decreasing with the increase of the distance between the proton and the nitrogen atom (276.2 ppm = quinoline nitrogen, 57.4 ppm = C4-nitrogen, 45.7 ppm = C3-nitrogen). The cross-peak at 5.8 ppm - 57.4 ppm (C4-nitrogen coupling with its protons) is an artefact of the acquisition since it should not be visible in the HMBC analysis, being a $^{1}J_{N-H}$ coupling. The assignment was then confirmed by the ^{15}N NMR analysis of compounds **2a*** and **2b*** (the ^{15}N-enrichment not only allowed direct ^{15}N NMR, but also caused splitting of the protons and carbons attached to the labelled nitrogen).

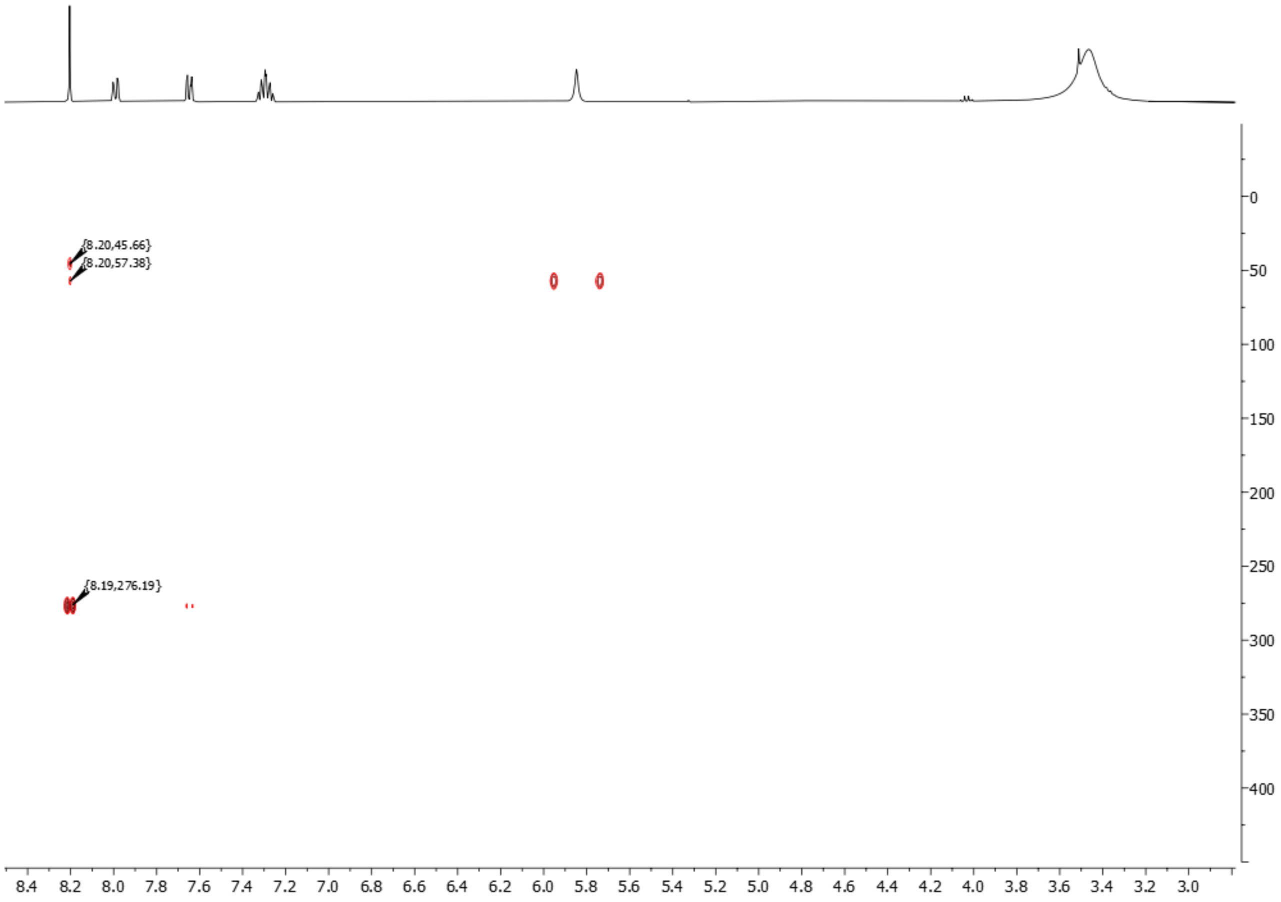

2.4.2 Synthesis of quinoline-3,4-diamine-^{15}N 2a*

This reaction has been performed with lower equivalents of $^{15}NH_3$ (5.5 equiv. rather than 35.0 equiv.) to minimize the consumption of labeled-ammonia.

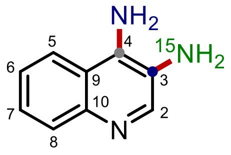

2a*

Prepared according to general procedure B (GP-B) using 3-azidoquinoline **Az-1** (34 mg, 0.2 mmol, 1.0 equiv.), $^{15}NH_3$ (7.0 M in MeOH, 157 µL, 1,1 mmol, 5.5 equiv.) and THF (1.30 mL). The obtained crude is purified on flash chromatography column (AcOEt:MeOH 9:1 +1% TEA) to obtain the title compound **2a*** as a red/brownish solid (14 mg, **44% yield**). ^{1}H NMR (400 MHz, DMSO-*d*$_6$) δ 8.21 (d, J = 1.2 Hz, 1H, H2), 8.03 – 7.95 (m, 1H, H5), 7.69 – 7.62 (m, 1H, H8), 7.36 – 7.21 (m, 2H, H6-H7), 5.85 (brs, 2H, H12), 4.70 (brd, J = 68.1 Hz, 2H, H11). ^{13}C NMR (101 MHz, DMSO-*d*$_6$) δ 142.8 (C10), 141.1 (d, J = 2.6 Hz, C2), 134.0 (d, J = 1.7 Hz, C4), 128.7 (C8), 125.1 (d, J = 11.5 Hz, C3), 124.4 (C7), 123.5 (C6), 121.0 (C5), 118.6 (C9).^{15}N NMR (41 MHz, DMSO-*d*$_6$) δ 45.0 (N11). MS (ESI), m/z [M+H]$^+$: 161.83. HRMS (ESI), [M+H]$^+$: Calculated for $[C_9H_{10}N_2{}^{15}N]^+$: 161.0840, found: 161.0832. (see spectra) *Some unidentified impurity could not be removed from this product even after a second purification on silica gel (direct phase or inverse phase).*

2.4.3 Synthesis of 6-methoxyquinoline-3,4-diamine-^{15}*N* 37*

This reaction has been performed with lower equivalents of $^{15}NH_3$ (5.5 equiv. rather than 35.0 equiv.) to minimize the consumption of labeled-ammonia.

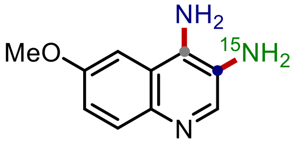

37*

Prepared according to general procedure B (GP-B) using 3-azido-6-methoxyquinoline **Az-2** (40 mg, 0.2 mmol, 1.0 equiv.), $^{15}NH_3$ (7.0 M in MeOH, 157 µL, 1,1 mmol, 5.5 equiv.) and THF (1.30 mL). The obtained crude is purified on flash chromatography column (AcOEt:MeOH 9:1 +1% TEA) to obtain title compound **37*** as a red/brownish solid (16 mg, **42% yield**). ^{1}H NMR (400 MHz DMSO-d$_6$) δ 8.08 (d, J = 1.2 Hz, 1H), 7.56 (d, J = 9.1 Hz, 1H), 7.32 (d, J = 2.7 Hz, 1H), 6.96 (dd, J = 9.1, 2.7 Hz, 1H), 5.69 (brs, 2H), 4.68 (brd, J = 76.1 Hz, 2H), 3.85 (s, 3H). ^{13}C NMR (101 MHz, DMSO-d$_6$) δ 155.8, 139.04 (d, J = 2.8 Hz), 138.7, 132.9, 130.2, 125.32 (d, J = 11.6 Hz), 119.1, 116.6, 99.7, 55.4. ^{15}N NMR (41 MHz, DMSO-d$_6$) δ 45.3. ^{15}N-^{1}H HMBC experiment shows cross peaks relative to C3-NH_2 (45.3 ppm) and C2-H (8.08 ppm), confirming the C3-labelling. MS (ESI), m/z [M+H]$^+$: 190.93. HRMS (ESI), [M+H]$^+$: Calculated for $[C_{10}H_{12}N_2{}^{15}NO]^+$: 191.0945, found: 191.0942. (see spectra)

2.4.4 Synthesis of quinolin-3-amine-^{15}*N* A-1*

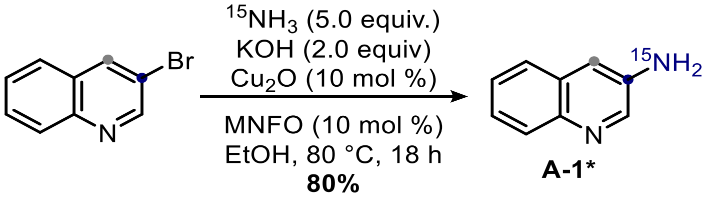

The procedure has been adapted from the literature.[7] 3-bromoquinoline (136 µL, 1.0 mmol, 1.0 equiv.), KOH (112 mg, 2.0 mmol, 2.0 equiv.), Cu_2O (14 mg, 0.10 mmol, 10 mol %) and **MNFO** (31 mg, 0.10 mmol, 10 mol %) were added to an 8-mL glass vial and dissolved in EtOH (4 mL). $^{15}NH_3$

(7.0 M in MeOH, 714 µL, 5.0 mmol, 5.0 equiv.) was added and the vial was closed with a plastic cap. The reaction mixture was heated at 80 °C under vigorous stirring. After completion, the solution was cooled to room temperature, concentrated under reduced pressure and purified on flash chromatography column (from Hex:AcOEt 1:1 to 100% AcOEt + 1% TEA) to obtain the title compound **A-1*** as white solid (116 mg, **80% yield**). ^{1}H NMR (400 MHz, $CDCl_3$) δ 8.51 (dd, J = 2.8, 1.1 Hz, 1H), 8.00 – 7.91 (m, 1H), 7.62 – 7.54 (m, 1H), 7.48 – 7.36 (m, 2H), 7.24 (ddd, J = 2.8, 1.8, 0.9 Hz, 1H), 3.91 (d, J = 80.3 Hz, 2H). ^{13}C NMR (101 MHz, $CDCl_3$) δ 143.3, 143.0, 139.9 (d, J = 11.4 Hz), 129.3, 128.9, 127.1, 126.0, 125.8, 115.1. ^{15}N NMR (41 MHz, $CDCl_3$) δ 50.6. MS (ESI), m/z $[M+H]^+$: 146.93. HRMS (ESI), $[M+H]^+$: Calculated for $[C_9H_9N^{15}N]^+$: 146.0731, found: 146.0728. (see spectra).

2.4.5 Synthesis of 3-(azido-1-^{15}*N*)quinoline Az-1*

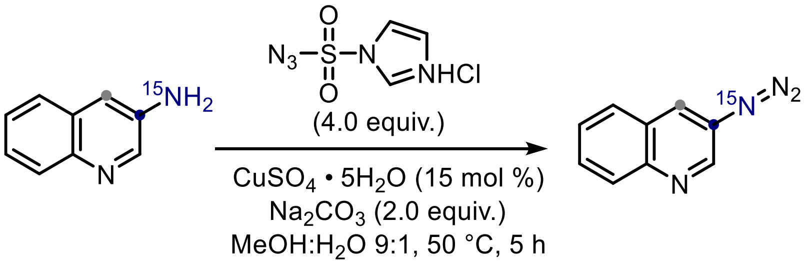

The procedure has been adapted from the literature.[9] Quinolin-3-amine-^{15}N (85 mg, 0.58 mmol, 1.0 equiv.), $CuSO_4·5H_2O$ (22 mg, 0.09 mmol, 15% mol), Na_2CO_3 (123 mg, 11.6 mmol, 2.0 equiv.) are added to a 25mL double-necked flask and dissolved in MeOH:H_2O 9:1 (4 mL). 1*H*-imidazole-1-sulfonyl azide hydrochloride (487 mg, 2.32 mmol, 4.0 equiv.) is added portionwise and the reaction is heated at 50 °C with vigorous stirring for 5 hours. Then, the reaction is concentrated under reduced pressure and purified on flash chromatography column (from 100% Hex to Hex:AcOEt 7:3) to obtain the title compound **Az-1*** as a pale-yellow solid (13 mg, **13% yield**). ^{1}H NMR (400 MHz, $CDCl_3$) δ 8.62 (d, J = 2.6 Hz, 1H), 8.09 (dd, J = 8.4, 1.1 Hz, 1H), 7.80 – 7.71 (m, 2H), 7.66 (ddd, J = 8.4, 6.9, 1.5 Hz, 1H), 7.56 (ddd, J = 8.2, 6.9, 1.2 Hz, 1H). ^{13}C NMR (101 MHz, CDCl3) δ 146.0, 144.0 (d, J = 8.4 Hz), 134.1, 129.6, 128.9, 128.3, 127.9, 127.0, 122.7. ^{15}N NMR (41 MHz, $CDCl_3$) δ 88.9. MS (ESI), m/z $[M+H]^+$: 172.19. HRMS (ESI), $[M+H]^+$: Calculated for $[C_9H_7N_3{}^{15}N]^+$: 172.0636, found: 172.0638. (see spectra)

2.4.6 Synthesis of quinoline-3,4-diamine-^{15}*N* 2b*

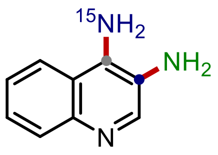

2b*

Prepared according to general procedure B (GP-B) using 3-(azido-1-^{15}*N*)quinoline **Az-1*** (28 mg, 0.16 mmol, 1.0 equiv.), NH_3 (7.0 M in MeOH, 700 µL, 4.8 mmol, 30.0 equiv.) and THF (1.30 mL). The obtained crude is purified on flash chromatography column (AcOEt:MeOH 9:1 +1% TEA) to obtain title compound **2b*** as a red/brownish solid (16 mg, **62% yield**). ^{1}H NMR (400 MHz, DMSO-d_6) δ 8.20 (d, J = 0.9 Hz, 1H), 8.02 – 7.96 (m, 1H), 7.69 – 7.60 (m, 1H), 7.36 – 7.22 (m, 2H), 5.85 (d, J = 85.8 Hz, 2H), 4.70

(brs, 2H). ^{13}C NMR (101 MHz, DMSO-d_6) δ 142.7, 140.9, 134.0 (d, J = 14.1 Hz), 128.5, 125.0, 124.4, 123.4, 120.9 (d, J = 1.6 Hz), 118.5. ^{15}N NMR (41 MHz, DMSO-d_6) δ 57.78. MS (ESI), m/z [M+H]+: 161.22. HRMS (ESI), $[M+H]^+$: Calculated for $[C_9H_{10}N_2{}^{15}N]^+$: 161.0840, found: 161.0837. (see spectra) *Some unidentified impurity could not be removed from this product even after a second purification on silica gel (direct phase or inverse phase).*

2.5 Synthesis of relevant heterocycles

2.5.1 Synthesis of *N*³-isobutylquinoline-3,4-diamine 38

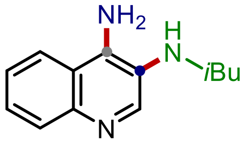

38

Prepared according to general procedure B (GP-B) using 3-azidoquinoline **Az-1** (34 mg, 0.2 mmol, 1.0 equiv.), 2-methylpropan-1-amine (44 µL, 0.44 mmol, 2.2 equiv.) and THF (1.30 mL). The obtained crude is purified on flash chromatography column (100% AcOEt + 1% TEA) to obtain the title compound **38** as a brown oil (35 mg, **81% yield**). ^{1}H NMR (400 MHz, $CDCl_3$) δ 8.44 (s, 1H), 7.95 (d, J = 8.4 Hz, 1H), 7.72 (d, J = 8.4 Hz, 1H), 7.51 (ddd, *J* = 8.4, 6.7, 1.4 Hz, 1H), 7.46 – 7.38 (m, 1H), 4.75 (brs, 2H), 2.98 (d, *J* = 6.7 Hz, 2H), 1.91 (dt, *J* = 13.2, 6.6 Hz, 1H), 1.04 (d, *J* = 6.6 Hz, 6H). ^{13}C NMR (101 MHz, CDCl3) δ 144.4, 141.7, 138.9, 129.4, 126.5, 126.2, 124.7, 119.9, 118.6, 53.9, 28.6, 20.5. MS (ESI), m/z [M+H]+: 216.44. HRMS (ESI), $[M+H]^+$: Calculated for $[C_{13}H_{18}N_3]^+$: 216.1495, found: 216.1492. (see spectra)

Gram scale synthesis of *N*³-isobutylquinoline-3,4-diamine 38

Prepared according to general procedure B (GP-B) using 3-azidoquinoline **Az-1** (1.0 g, 5.9 mmol, 1.0 equiv.), 2-methylpropan-1-amine (1.2 mL, 12.9 mmol, 2.2 equiv.) and THF (12 mL). The obtained crude is purified on flash chromatography column (100% AcOEt + 1% TEA) to obtain the title compound **38** as a brown oil (873 mg, **69% yield**).

2.5.2 Synthesis of 3-isobutyl-3H-imidazo[4,5-c]quinolin-4-amine 39

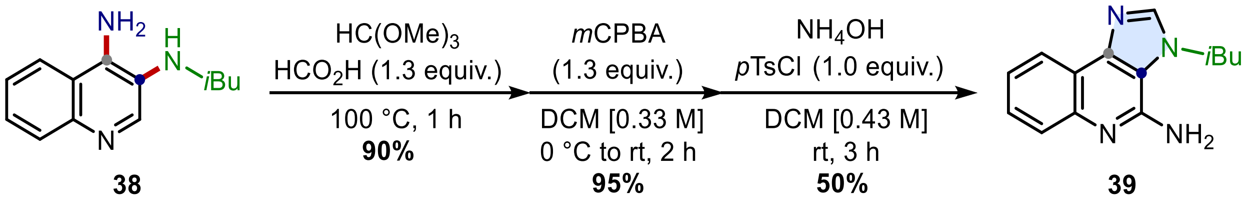

Step 1: Synthesis of 3-isobutyl-3*H*-imidazo[4,5-c]quinoline

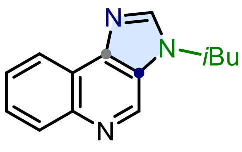

*N*³-isobutylquinoline-3,4-diamine **38** (240 mg, 1.1 mmol, 1.0 equiv.) was dissolved in trimethyl orthoformate (3.0 mL, 0.36 M) in an 8-mL glass vial. Formic acid (55 µL, 1.4 mmol, 1.3 equiv.) was added and the mixture was heated at 100 °C for 1 hour. After completion, the reaction was concentrated under reduced pressure and the crude was treated with saturated $NaHCO_3$ for 10 minutes, followed by extractions with AcOEt (3 x 10 mL). The collected organic phases were dried over Na_2SO_4, filtered

and concentrated under reduced pressure to give the desired product as a brown oil (225 mg, **90% yield**). ^{1}H NMR (400 MHz, $CDCl_3$) δ 9.13 (s, 1H), 8.61 (dd, *J* = 7.5, 2.2 Hz, 1H), 8.40 – 8.19 (m, 1H), 8.07 (s, 1H), 7.93 – 7.59 (m, 2H), 4.19 (d, *J* = 7.3 Hz, 2H), 2.32 (dt, *J* = 13.6, 6.8 Hz, 1H), 1.01 (d, *J* = 6.6 Hz, 6H). ^{13}C NMR (101 MHz, $CDCl_3$) δ 145.6, 144.5, 143.6, 135.6, 129.2, 127.9, 127.6, 127.4, 122.8, 121.9, 53.5, 30.0, 20.2. MS (ESI), m/z [M+H]+: 226.28. HRMS (ESI), $[M+H]^+$: Calculated for $[C_{14}H_{16}N_3]^+$: 226.1339, found: 226.1336. (see spectra)

Step 2: Synthesis of 3-isobutyl-3*H*-imidazo[4,5-*c*]quinoline 5-oxide

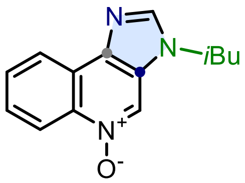

3-isobutyl-3*H*-imidazo[4,5-c]quinoline (150 mg, 0.66 mmol, 1.0 equiv.) was dissolved in DCM (5 mL, 0.33 M) and cooled to 0 °C. *m*CPBA (148 mg, 0.86 mmol, 1.3 equiv.) was added and reaction was allowed to warm to room temperature over 2 hours. The reaction was then quenched with saturated $NaHCO_3$ followed by extractions with AcOEt (3 x 10 mL). The collected organic phases were dried over Na_2SO_4, filtered and concentrated in vacuo to give the desired product as a brown oil (151 mg, **95% yield**), which was then directly used as a crude material in the following step. ^{1}H NMR (400 MHz, $CDCl_3$) δ 9.11 (s, 1H), 8.95 – 8.79 (m, 1H), 8.69 – 8.53 (m, 1H), 8.06 (s, 1H), 7.84 – 7.73 (m, 2H), 4.08 (d, *J* = 7.3 Hz, 2H), 2.27 (dp, *J* = 13.6, 6.8 Hz, 1H), 1.01 (d, *J* = 6.7 Hz, 6H). (see spectrum)

Step 3: Synthesis of 3-isobutyl-3*H*-imidazo[4,5-*c*]quinolin-4-amine 39

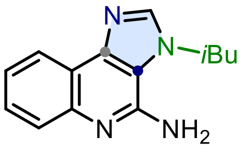

39

This procedure has been adapted from the literature.[13] To a solution of 3-isobutyl-3*H*-imidazo[4,5-c]quinoline 5-oxide (100 mg, 0.41 mmol, 1.0 equiv.) in DCM (1.0 mL, 0.43 M) was added concentrated ammonium hydroxide (321 µL, 1.3 M), followed by dropwise addition of a solution of *p*-toluenesulfonyl chloride (79 mg, 0.41 mmol, 1.0 equiv.) in DCM (320 µL). The reaction was stirred at room temperature for 3 hours. A precipitate was collected by vacuum filtration, washed first with DCM and then with water. The precipitate was then purified by direct phase chromatography (Hex/AcOEt + 1% TEA), affording product **39** (48 mg, 0.20 mmol, **50% yield**). ^{1}H NMR (400 MHz, $CDCl_3$) δ 8.37 (dd, *J* = 8.0, 1.6 Hz, 1H), 7.82 (s, 1H), 7.77 (d, *J* = 8.3 Hz, 1H), 7.54 (ddd, *J* = 8.5, 7.0, 1.5 Hz, 1H), 7.46 – 7.37 (m, 1H), 5.16 (brs, 2H), 4.08 (d, *J* = 7.5 Hz, 2H), 2.21 (dh, *J* = 14.2, 7.1 Hz, 1H), 0.94 (d, *J* = 6.6 Hz, 6H). ^{13}C NMR (101 MHz, $CDCl_3$) δ 147.3, 146.0, 144.5, 143.1, 127.9, 125.5, 123.7, 121.6, 120.2, 117.8, 54.9, 30.8, 19.8. MS (ESI), m/z $[M+H]^+$: 241.40. HRMS (ESI), $[M+H]^+$: Calculated for $[C_{14}H_{17}N_4]^+$: 241.1448, found: 241.1443. (see spectra)

2.5.3 Synthesis of 3-isobutyl-3*H*-[1,2,3]triazolo[4,5-*c*]quinoline 40

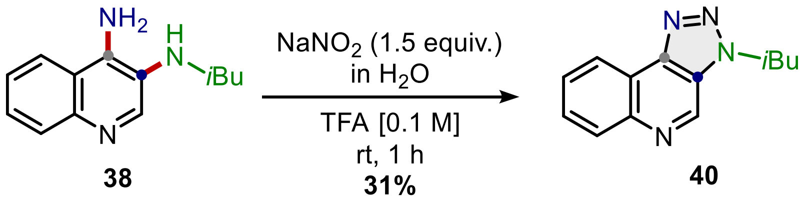

N^3-isobutylquinoline-3,4-diamine **38** (70 mg, 0.32 mmol, 1.0 equiv.) was dissolved in TFA (3.2 mL, 0.1 M). $NaNO_2$ (34 mg, 0.49 mmol, 1.5 equiv.) dissolved in 1 mL of H_2O was slowly added to the solution and stirred at room temperature for 1 hour. Upon completion, the reaction was neutralized with $NaHCO_3$ and extracted 3 times with AcOEt. The collected organic phases were dried over Na_2SO_4, filtered and concentrated in vacuo. The crude was purified by reverse phase chromatography (eluent mixture: H_2O/ACN) to afford pure compound **40** (23 mg, 0.10 mmol, **31% yield**). NB: HPLC-MS and NMR analysis of the crude confirmed the formation of the nitrosamine byproduct (N-(4-aminoquinolin-3-yl)-N-isobutylnitrous amide) in a 2:1 ratio with the product, accounting for the loss of yield. ^{1}H NMR (400 MHz, $CDCl_3$) δ 9.23 (s, 1H), 8.72 (dd, J = 6.2, 3.4 Hz, 1H), 8.27 (dd, J = 6.2, 3.4 Hz, 1H), 7.79 (dd, J = 6.2, 3.4 Hz, 2H), 4.63 (d, J = 7.3 Hz, 2H), 2.47 (dp, J = 13.7, 6.9 Hz, 1H), 1.02 (d, J = 6.7 Hz, 6H). ^{13}C NMR (101 MHz, $CDCl_3$) δ 146.2, 144.2, 136.0, 130.0, 128.9, 128.8, 128.0, 122.3, 120.3, 56.5, 30.1, 20.2. MS (ESI), m/z $[M+H]^+$: 227.13. HRMS (ESI), $[M+H]^+$: Calculated for $[C_{13}H_{15}N_4]^+$: 227.1291, found: 227.1288. (see spectra)

2.5.4 Synthesis of 3-isobutyl-1,3-dihydro-2*H*-imidazo[4,5-*c*]quinolin-2-one 41

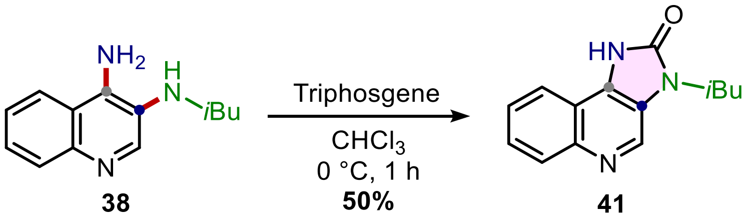

This procedure was adapted from the literature.[14] N^3-isobutylquinoline-3,4-diamine **38** (100 mg, 0.46 mmol, 1.0 equiv.) was dissolved in chloroform (3.3 mL, 0.14 M) and cooled to 0 °C. A solution of triphosgene (179 mg, 0.60 mmol, 1.3 equiv.) in chloroform (1.5 mL) was added and stirred for 1 hour. The reaction mixture was quenched with 10% NaOH solution to pH 12 at 0 °C. The mixture was washed with DCM to remove impurities; then the aqueous layer was acidified to pH 2.0 with a 2 N HCl solution and evaporated. The residue was purified by direct phase chromatography (Hex/AcOEt + 1% TEA) to afford product **41** as a white solid (55 mg, 0.23 mmol, **50% yield**). ^{1}H NMR (400 MHz, DMSO-d_6) δ 12.19 (brs, 1H), 8.86 (s, 1H), 8.10 (dd, J = 8.1, 1.7 Hz, 1H), 8.04 – 7.92 (m, 1H), 7.59 (dtd, J = 14.0, 6.8, 1.7 Hz, 2H), 3.79 (d, J = 7.4 Hz, 2H), 2.19 (dt, J = 13.8, 7.0 Hz, 1H), 0.93 (d, J = 6.7 Hz, 6H). ^{13}C NMR (101 MHz, $CDCl_3$) δ 154.0, 143.6, 133.2, 129.5, 128.9, 126.8, 126.0, 123.3, 121.1, 114.6, 47.5, 27.7, 19.7. MS (ESI), m/z $[M+H]^+$: 242.86. HRMS (ESI), $[M+H]^+$: Calculated for $[C_{14}H_{16}N_3O]^+$: 242.1288, found: 242.1282. (see spectrum)

2.5.5 Synthesis of 3-isobutyl-1,3-dihydro-2H-imidazo[4,5-c]quinoline-2-thione 42

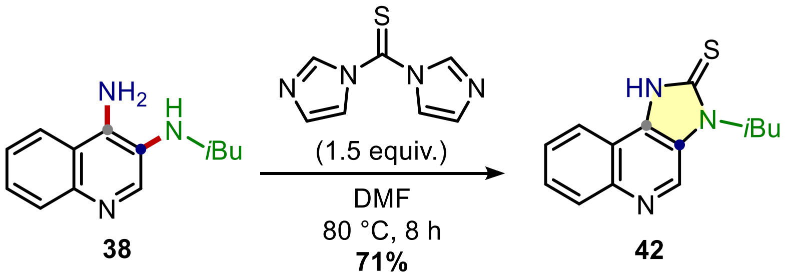

N^3-isobutylquinoline-3,4-diamine **38** (100 mg, 0.46 mmol, 1.0 equiv.) was dissolved in DMF (1.0 mL, 0.46 M), followed by the addition of di(1*H*-imidazol-1-yl)methanethione (124 mg, 0.70 mmol, 1.5 equiv.). The mixture was heated at 80 °C for 8 hours. The resulting precipitate was collected and purified by reverse-phase chromatography (eluent mixture H_2O/ACN) to afford product **42** as a light yellow solid (84 mg, 0.33 mmol, **71% yield**). ^1H NMR (400 MHz, DMSO-d_6) δ 9.08 (s, 1H), 8.40 – 8.29 (m, 1H), 8.15 – 8.00 (m, 1H), 7.77 – 7.60 (m, 2H), 4.23 (d, J = 7.6 Hz, 2H), 2.42 (dt, J = 13.9, 6.9 Hz, 1H), 0.95 (d, J = 6.7 Hz, 6H). ^{13}C NMR (101 MHz, DMSO-d_6) δ 168.5, 144.0, 134.7, 131.7, 129.5, 128.0, 127.0, 126.6, 121.5, 114.5, 50.5, 27.4, 19.7. MS (ESI), m/z $[M+H]^+$: 257.50. HRMS (ESI), $[M+H]^+$: Calculated for $[C_{14}H_{16}N_3S]^+$: 258.1059, found: 258.1053. (see spectra)

2.6 Single-crystal X-ray diffraction analysis

CCDC 2521323 contains the supplementary crystallographic data for this paper. The data is available from the Cambridge Crystallographic Data Centre via www.ccdc.cam.ac.uk/structures. *Sample specs*:

Sample preparation: slow evaporation from a dichloromethane solution
Sample description: plate, transparent, colorless with dimensions: 0.20 x 0.20 x 0.05 mm.

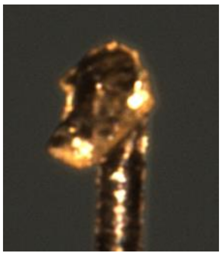

Mounting: on a glass fiber, with perfluorinated oil
Comments: The selected sample was obtained from a slow-evaporation batch and was chosen from various fragments produced by cracking a block of epitaxially grown plates crystals using a micro-scraper. The samples are particularly fragile and easily cracks when manipulated. A fragment of a larger plate, cut with a micro-knife, was selected as it showed strong pleochroism under polarized light, from colorless to blue.

Instrumental specs:

Device: Rigaku XtaLAB Synergy-S 4-circle diffractometer

Source: microfocus sealed tube

Detector: Hybrid Photon Counting (HPC)

Experiment temperature: 293(2) K

Cryostat: not used

Wavelength: Cu K_α (1.54184 Å).

Data collection extent: full sphere within $\sin\vartheta/\lambda = 0.60$ Å^{-1}

Data collections specs: Detector-to-sample distance: variable, several w-scan run.

Measured reflections: 64974, 2646 independents by symmetry

Maximum resolution (ϑ): 80.579 °

Completeness: 99.3 % (at full sphere resolution)

Data reduction programs:

Integration: CrysalisPro

Reduction: CrystalsPro

Structure solution and refinement: Shelxs2019, Shelxl 2019

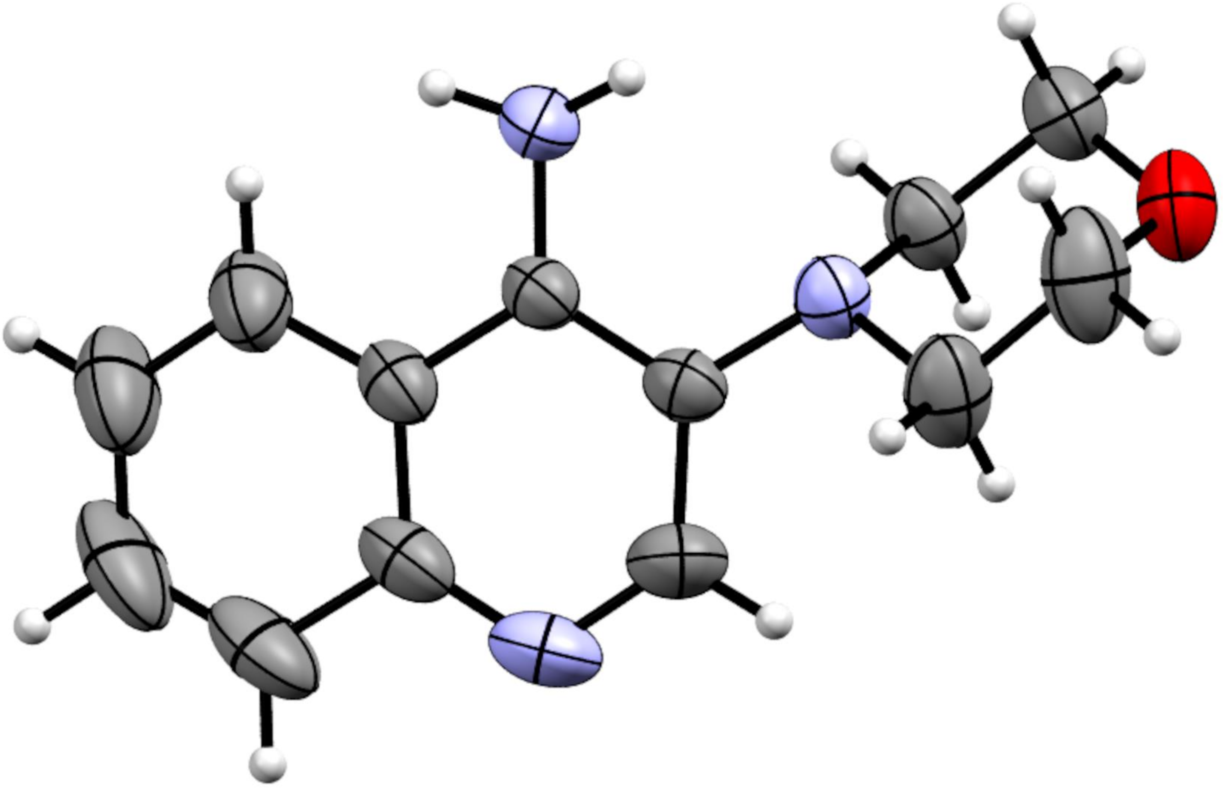

Molecular structure of **18** with thermal ellipsoid contour at 50% probability.

Unit cell, lattice and crystal system:

Bravais lattice: Orthorombic, Primitive

Space group: P n a 2_1, No. 33
Point group: mm2
Laue group: mmm, No. 3

Unit cell (Å, deg, Å^3): a = 13.4510(2), b = 10.11590(10), c = 8.93660(3), $\alpha = \beta = \gamma = 90.0$, V = 1215.99(3) as estimated from 38032 intense reflections among 5.4140 ° e 79.0750 ° of $\vartheta\vartheta$ (final integration result).

Formula units in cell (Z): 4
Formula units in the asymmetric unit (Z'): 1
Number of electrons in cell (F_{000}): 488
Computed density: 1.252 g/cm^3
Linear absorption coefficient (μ): 0.658 mm^{-1}

Main statistical results:
Final stats for the spherical atom model (Shelxl):
Scale factor: 8.48294(2)
BASF parameter: //
Secondary extinction coefficient: none
$<\Delta\Delta/\sigma/\sigma> = 0.000$
R1(F) = 0.0296 for 2513 > $4\sigma(F_o)$, 0.0311 for all the 2646 independent data
wR(F^2) = 0.0854 for all the measured data
Goodness-of-fit: 1.044
Flack's parameter: //
$\rho_{MAX/MIN}$ = +0.12 e/Å^3 at ~ 0.66 Å from the C5 carbon, –0.11 e/Å^3 at ~ 0.95 Å from the C8 carbon atom.

The least-squares refinement statistics indicate that the overall quality of the collected data is high, with an R1 value below 3% for observed reflections. A minor disorder is present for the hydrogen atoms attached to the N atoms, with the major component accounting for approximately 90%. Small residual electron-density peaks are observed near neighboring C atoms; however, these are not chemically significant and no additional disorder could be reliably modeled.

2.7 UV/Vis analysis

UV/Vis spectra of a solution of **Az-1** or **Az-8** (0.025 mM in THF, inhibitor free) have been acquired on a JASCO UV-Vis Spectrophotometer.

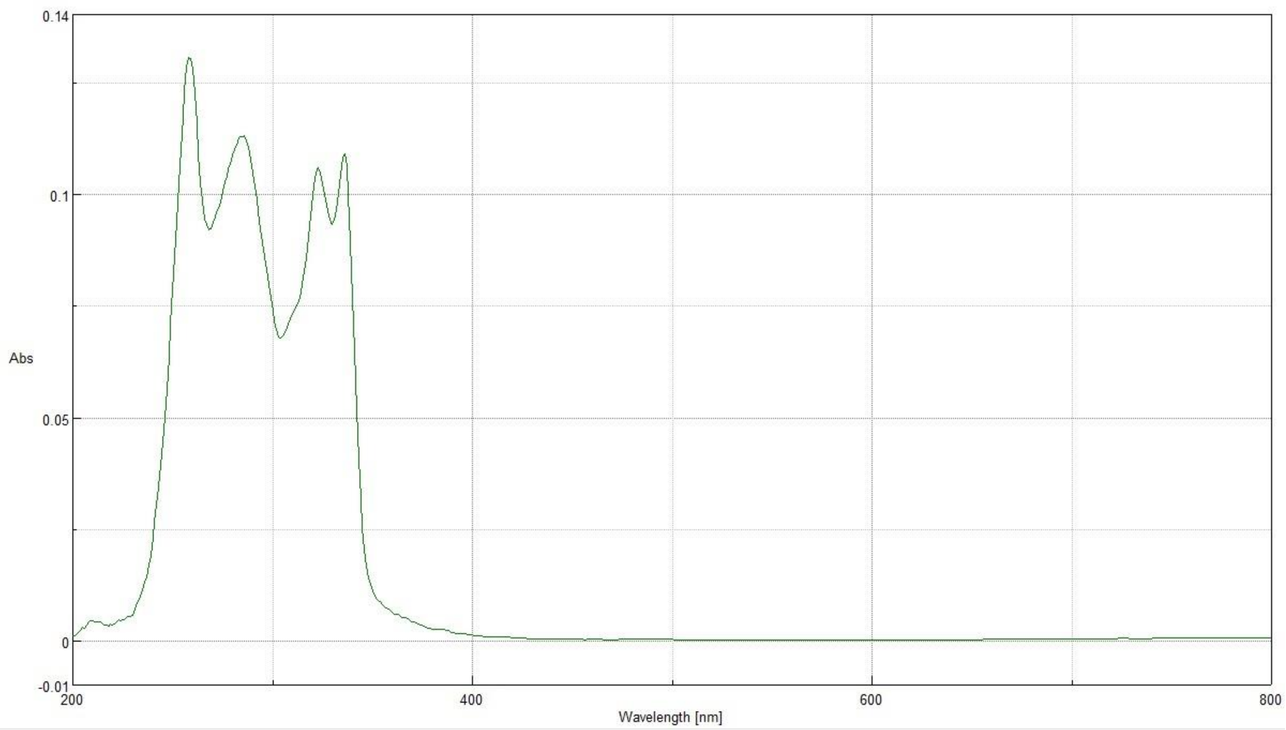

UV/Vis spectrum of **Az-1**.

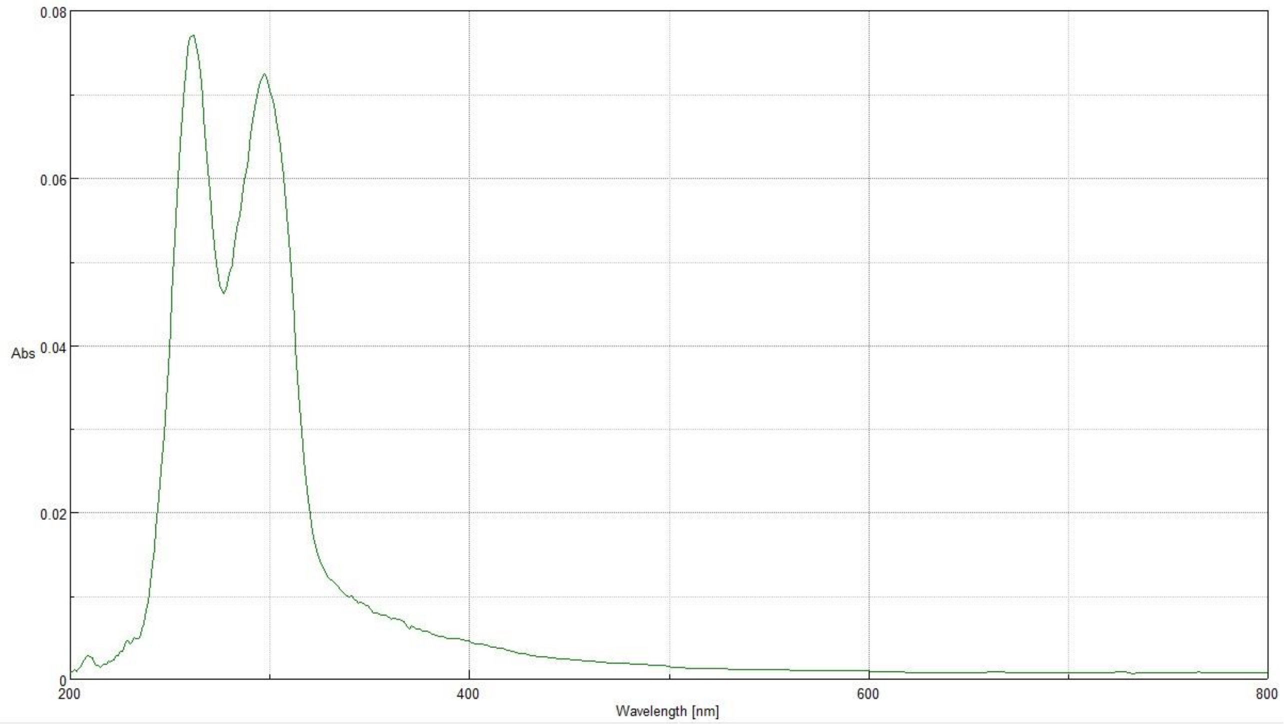

UV/Vis spectrum of **Az-8**.

3. DFT CALCULATIONS

Calculations were carried out following the same protocol reported in the literature for phenyl azides;[15] these reference calculations were independently repeated and yielded identical results. All computations were carried out in the gas phase, given the minimal solvation effects reported for phenyl azides. The *o*-azirine intermediate was found to lie in a flat region of the potential energy surface, leading to barrierless relaxation to the corresponding *o*-ketenimine. All calculations were performed with Gaussian 16 C.02 software package.[16] Geometry optimizations were carried out using the unrestricted formalism of M06-2X functional[17] in combination with the def2-TZVP basis set,[18] as implemented in Gaussian 16. The same level of theory was employed for vibrational frequency analyses to confirm that no negative eigenvalues in the Hessian matrix, with only one negative eigenvalue for transition states. No symmetry restrictions were applied. Further single-point energy calculations were performed at the UM06-2X/def2-QZVPP level of theory in the gas phase. Thermodynamic data were obtained via quasi-harmonic corrections to entropy using the rigid rotor/harmonic oscillator (RRHO) approximation, in which vibrational modes below 100 cm^{-1} were treated with a free rotor approximation interpolated with a damping function. These corrections were applied with *Goodvibes.py*,[19] assuming a solution-phase standard state (c = 1 mol^{-1}). All energy values are reported in kcal/mol. Cartesian coordinates are provided below.

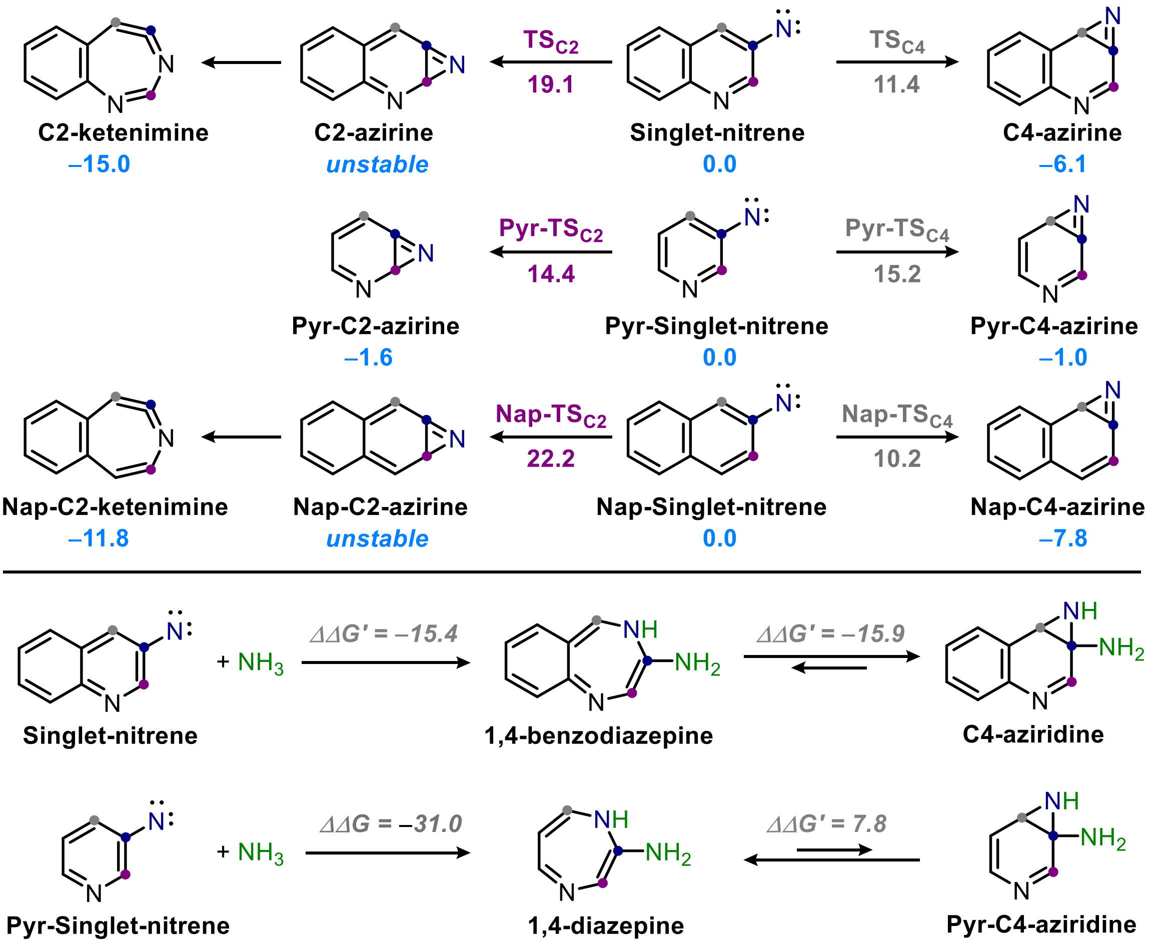

	E_SPC	E	ZPE	H_SPC	qh-H_SPC	T.S	G(T)_SPC	ΔG(T)_SPC
**Singlet-nitrene**	-455.976332	-455.929493	0.124020	-455.843611	-455.843855	0.041226	-455.884837	**0.0**
**TS_{C2}**	-455.942579	-455.905239	0.120760	-455.813001	-455.813245	0.041319	-455.854320	**19.1**
**C2-ketenimine**	-456.001011	-455.963875	0.12462	-455.86771	-455.867923	0.040994	-455.908704	**-15.0**
**TS_{C4}**	-455.956905	-455.919609	0.122590	-455.825801	-455.826076	0.040883	-455.866685	**11.4**
**C4-azirine**	-455.987522	-455.950713	0.125147	-455.853886	-455.854100	0.040740	-455.894626	**-6.1**
**1,4-benzodiazepine**	-512,581444	-512,536457	0,164557	-512,406029	-512,4067	0,045811	-512,45184	
**C4-aziridine**	-512,609168	-512,564869	0,165855	-512,433265	-512,433692	0,04393	-512,477195	
**Pyr-Singlet-nitrene**	-302.324405	-302.291306	0.07803	-302.240177	-302.240238	0.034968	-302.275144	**0.0**
**Pyr-TS_{C2}**	-302.299491	-302.274449	0.07591	-302.217582	-302.217613	0.034546	-302.252128	**14.4**
**Pyr-C2-azirine**	-302.327745	-302.303083	0.07855	-302.243119	-302.24315	0.03453	-302.277649	**-1.6**
**Pyr-TS_{C4}**	-302.298204	-302.273199	0.075935	-302.216234	-302.216264	0.034659	-302.250893	**15.2**
**Pyr-C4-azirine**	-302.327064	-302.302489	0.078693	-302.242347	-302.242377	0.034456	-302.276803	**-1.0**
**1,4-diazepine**	-358,955214	-358,92246	0,119304	-358,827772	-358,828077	0,039291	-358,867063	
**Pyr-C4-aziridine**	-358,943712	-358,911594	0,119451	-358,816667	-358,816759	0,037879	-358,854547	
**Nap-Singlet-nitrene**	-439.93489	-439.887605	0.13562	-439.79042	-439.790664	0.041442	-439.831863	**0.0**
**Nap-TS_{C2}**	-439.897088	-439.860083	0.133	-439.755364	-439.755608	0.041152	-439.796517	**22.2**
**Nap-C2-ketenimine**	-439.95449	-439.917679	0.13632	-439.809348	-439.809592	0.041266	-439.850614	**-11.8**
**Nap-TS_{C4}**	-439.917473	-439.880461	0.134306	-439.774529	-439.774773	0.041042	-439.815571	**10.2**
**Nap-C4-azirine**	-439.948598	-439.912056	0.136667	-439.803243	-439.803487	0.041049	-439.844291	**-7.8**
**NH_3**	-56,557997	-56,549631	0,033526	-56,520651	-56,520651	0,021846	-56,542497	

Singlet-nitrene

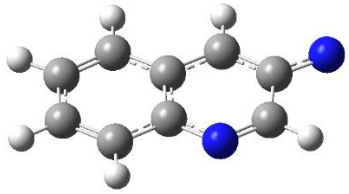

0 1
C 0.00000000 0.68178800 0.00000000
C 1.40024300 0.77590100 0.00000000
C -0.58201300 -0.61359800 0.00000000
H 1.89387800 1.74022300 0.00000000
C 2.16909300 -0.38621000 0.00000000
C 1.45892700 -1.64934600 0.00000000
H 2.04931800 -2.55972500 0.00000000
N 3.49919000 -0.35608200 0.00000000
N 0.17004800 -1.75565300 0.00000000
C -1.98586000 -0.73571900 0.00000000
H -2.40504600 -1.73288300 0.00000000
C -2.77482700 0.38298000 0.00000000
H -3.85239300 0.28237100 0.00000000
C -2.19984600 1.67344000 0.00000000
H -2.84170200 2.54429300 0.00000000
C -0.84233000 1.82124000 0.00000000
H -0.38904100 2.80501100 0.00000000

TS$_{C4}$

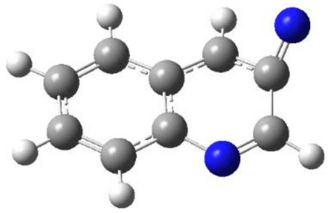

0 1
C 0.16777300 -0.65029600 -0.16218000
C -1.14069900 -1.15457400 -0.39869400
C 0.30349400 0.75695700 -0.03975800
H -1.35460800 -2.17301200 -0.67609000
C -2.21199700 -0.24608200 -0.04879700
C -1.96869700 1.20601300 -0.19255500
H -2.79352800 1.90069800 -0.31929500
N -2.86608000 -0.96850900 0.73237300
N -0.77359300 1.64512200 -0.18251700
C 1.56552000 1.30887100 0.15933900
H 1.64286600 2.38412200 0.25015700
C 2.68255800 0.49544200 0.21807300
H 3.65876500 0.93379300 0.37771500
C 2.56065500 -0.88681400 0.05295400
H 3.44229900 -1.51315900 0.08294800
C 1.31950900 -1.45356500 -0.13899600
H 1.21322700 -2.52445300 -0.26074300

C4-azirine

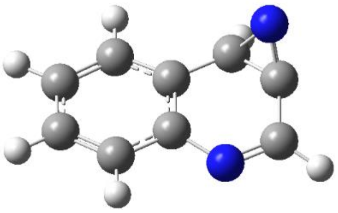

0 1
C 0.12926300 -0.66453600 -0.17387600
C -1.20635500 -1.24865700 -0.33828800
C 0.19324900 0.74197500 -0.01997600
H -1.39360600 -2.05676500 -1.03211000
C -2.21873300 -0.25173800 -0.00490500
C -2.10370200 1.17959100 -0.22201600
H -2.93009800 1.84606000 -0.43753800
N -2.14909600 -1.13957200 0.86953400
N -0.90442100 1.61098200 -0.18482800
C 1.43396000 1.35288900 0.18271400
H 1.45665500 2.42802600 0.30241900
C 2.59040700 0.60093800 0.20270000
H 3.54505000 1.08316500 0.36587200
C 2.53271300 -0.77560100 -0.01444100
H 3.44338700 -1.36026900 -0.01949000
C 1.31409200 -1.39697300 -0.21222600
H 1.26386700 -2.46741700 -0.37021000

TS$_{C2}$

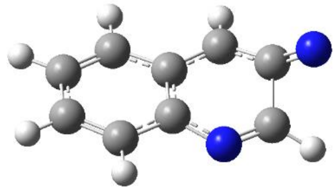

0 1
C -0.22563900 0.71365400 -0.11653800
C 0.98315200 1.38650600 -0.22774400
C -0.24419000 -0.71924800 -0.01130400
H 1.05884300 2.42718100 -0.50991500
C 2.13239600 0.59331000 0.02539100
C 2.01137800 -0.89163500 -0.40921600
H 2.89173300 -1.40864600 -0.74913000
N 3.08454500 0.30714200 0.75102000
N 0.86856700 -1.46306700 -0.25867900
C -1.48006200 -1.38262600 0.14494400
H -1.46357900 -2.46256400 0.20338100
C -2.64809500 -0.67734800 0.20623000
H -3.58817900 -1.19643500 0.33674100
C -2.64267600 0.73333800 0.06664400
H -3.57868000 1.27534100 0.09592500
C -1.47410800 1.40611700 -0.11329800
H -1.46486000 2.48419500 -0.21405500

C2-ketenimine

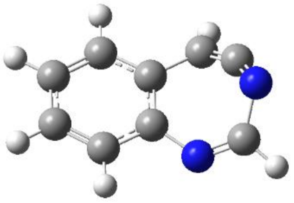

0 1
C -0.27831400 0.78237200 -0.17870500
C 0.91246500 1.63564500 -0.37533600
C -0.11411800 -0.62290900 -0.17672500
H 0.94849200 2.51696600 -0.99363800
C 1.92127200 1.06595700 0.23700200
C 2.24562200 -1.01711000 -0.08883000
H 3.09480000 -1.63782800 -0.35459000
N 2.53207700 0.12912500 0.74577300
N 1.07855700 -1.32881300 -0.44774300
C -1.24281500 -1.42937900 0.01234800
H -1.10234100 -2.50194300 -0.00637000
C -2.49245000 -0.87712200 0.19893900
H -3.34876700 -1.52135200 0.34792200
C -2.65466800 0.50702800 0.16729800
H -3.63847000 0.94262400 0.28214700
C -1.55669000 1.32224100 -0.01652800
H -1.66998100 2.39900200 -0.03844600

1,4-benzodiazepine

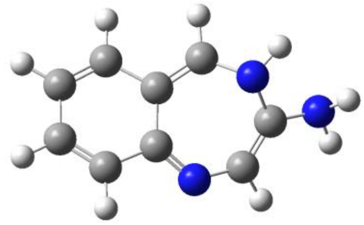

0 1
C 0.59540600 -0.69541600 -0.04649800
C -0.49956600 -1.42798400 0.23728000
C 0.65439000 0.77168800 0.12820400
H -0.45090600 -2.50692200 0.11951700
C -2.28092300 0.12397600 -0.00758300
C -1.61969100 1.25248400 -0.29318800
H -2.17185500 2.03440300 -0.80201000
N -1.69527100 -0.93306700 0.76270900
N -0.32460700 1.60456100 0.04813100
C 1.98742600 1.33099900 0.31748500
H 2.03066000 2.38054500 0.57639500
C 3.09423400 0.60670000 0.08403800
H 4.07007900 1.06644000 0.17807900
C 3.01411200 -0.78090900 -0.32491800
H 3.92411100 -1.32087500 -0.54908100
C 1.82255700 -1.39382900 -0.39328100
H 1.75116200 -2.44284900 -0.65634300
N -3.58117300 -0.16279400 -0.42781000
H -4.20615900 -0.39454800 0.33636900
H -3.98315400 0.57395400 -0.98978400
H -2.36425100 -1.67729200 0.89641600

C4-aziridine

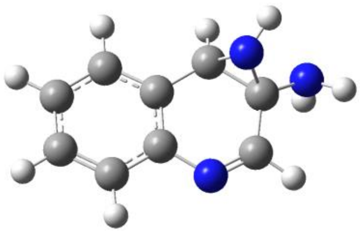

0 1
C 0.48330200 -0.58691600 -0.06153400
C -0.90939700 -1.06839700 -0.02261200
C 0.72197300 0.78947100 0.04980700
H -1.13393200 -2.00562500 -0.51730600
C -1.98734200 -0.03518700 -0.02222400
C -1.51506700 1.37550300 -0.03194000
H -2.28153700 2.14544700 -0.13487400
N -1.66069100 -0.76317200 1.19885200
N -0.30606700 1.75335400 0.03772600
C 2.03273700 1.25939000 0.07708400
H 2.18691000 2.32695000 0.16317700
C 3.09609100 0.37929200 -0.02375000
H 4.11126500 0.75298000 0.00182000
C 2.85823400 -0.98212500 -0.17571000

H 3.68688100 -1.67206700 -0.26686700
C 1.55606300 -1.45801200 -0.20057400
H 1.36607800 -2.51965400 -0.30738100
N -3.23038200 -0.35961600 -0.64995400
H -3.99543400 0.17114400 -0.24809100
H -3.20501000 -0.17397100 -1.64507300
H -2.37480200 -1.47727700 1.31693700

Pyr-Singlet-nitrene

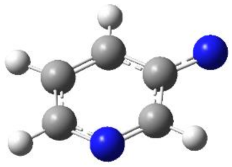

0 1
C 1.18132200 -1.05062200 0.00000000
C 1.21987200 0.32616400 0.00000000
C -0.05548200 -1.68887700 0.00000000
H 2.15022200 0.87816100 0.00000000
C 0.00000000 1.03979600 0.00000000
C -1.19885900 0.27595700 0.00000000
H -2.14830100 0.79881900 0.00000000
N -0.04415900 2.37308000 0.00000000
N -1.22183700 -1.04245100 0.00000000
H -0.11271800 -2.77147700 0.00000000
H 2.09165300 -1.63441400 0.00000000

Pyr-TS$_{C4}$

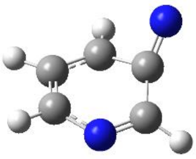

0 1
C -0.72724900 1.31216900 -0.01024600
C 0.57923200 1.04872800 -0.41347500
C -1.54869000 0.24191000 0.28041300
H 1.27691800 1.77747900 -0.78609000
C 1.04109100 -0.31897500 -0.12413800
C 0.01675000 -1.37373600 -0.23043100
H 0.25450600 -2.36520700 -0.60022700
N 2.05695500 -0.03234800 0.52977600
N -1.20279000 -1.05013300 0.00234800
H -2.55606200 0.38449200 0.64670400
H -1.12132500 2.32001800 0.00201200

Pyr-C4-azirine

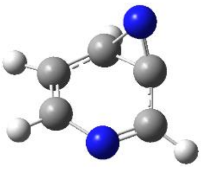

0 1
C 0.05363400 1.41806300 -0.10393000
C 1.12927000 0.51892600 -0.43385300

C -1.15048400 0.88805000 0.25217300
H 1.86670400 0.72729300 -1.19522400
C 0.70654700 -0.87610300 -0.09803900
C -0.65099200 -1.33935200 -0.22065400
H -0.94728200 -2.31473700 -0.58378600
N 1.56871500 -0.44138200 0.68346000
N -1.51716800 -0.42788200 0.03693700
H -1.96188300 1.52423300 0.57797900
H 0.15378100 2.49055100 -0.21593800

Pyr-TS$_{C2}$

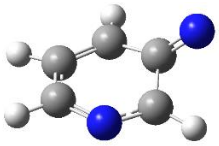

0 1
C -1.26811300 0.93305300 0.04311400
C -0.02825000 1.40081700 -0.21818900
C -1.49908300 -0.46788400 0.23886700
H 0.19251000 2.43224800 -0.45352200
C 1.00902200 0.38966800 -0.14781700
C 0.61713000 -1.01429100 -0.37393600
H 1.34779300 -1.73783100 -0.69205600
N 2.04874400 0.12676300 0.48860900
N -0.60616200 -1.40339300 -0.00810300
H -2.50283000 -0.80655500 0.46850700
H -2.11978500 1.60036800 0.06129300

Pyr-C2-azirine

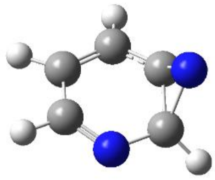

0 1
C 1.51126200 -0.30670000 0.07818200
C 0.70183300 -1.34915500 -0.22289700
C 0.99263100 1.04547500 0.22197200
H 1.02419200 -2.32984400 -0.53828700
C -0.64647800 -0.91017200 -0.10241900
C -1.12419000 0.46617100 -0.42207900
H -1.88044600 0.69297700 -1.15798000
N -1.53437900 -0.50710400 0.67241200
N -0.18706300 1.44693500 -0.12993100
H 1.71047500 1.81959300 0.47589600
H 2.58552800 -0.43525500 0.10644800

1,4-diazepine

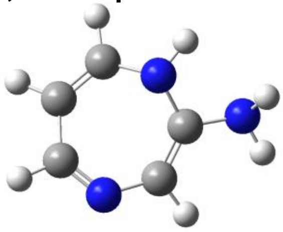

0 1
C -1.73165300 0.74974100 -0.30184800
C -0.66064500 1.51484100 -0.10332700
C -1.84718100 -0.65507600 0.10484900
H -0.68371500 2.56334200 -0.37949700
C 1.09552900 -0.13160100 0.03403200
C 0.40798200 -1.27255300 -0.11261100
H 0.97120400 -2.15180100 -0.39916500
N 0.54194100 1.09455400 0.48776500
N -0.93232700 -1.53248000 0.18971900
H -2.85487900 -0.99085700 0.34883300
H -2.61007200 1.21571200 -0.72830300
N 2.46083800 -0.01632600 -0.25086300
H 2.98811000 0.38439000 0.51684000
H 2.87396300 -0.89707700 -0.52273000
H 1.23802500 1.82394600 0.45109900

Pyr-C4-aziridine

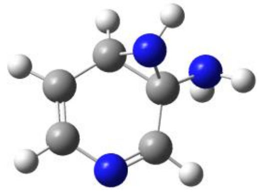

0 1
C 1.33306300 -1.05143800 -0.37220400
C -0.12093400 -1.15789600 -0.25874800
C 1.94629300 0.10019400 -0.07340500
H -0.64549500 -1.88735000 -0.86219800
C -0.86214700 0.13425800 0.02687800
C 0.00844400 1.32522500 0.13693500
H -0.48929900 2.29580400 0.14928300
N -0.68706200 -0.86899100 1.06258700
N 1.28185100 1.31170400 0.14678100
H 3.02482100 0.18263200 -0.09295900
H 1.90429800 -1.90641400 -0.70937100
N -2.18329800 0.27667100 -0.50052700
H -2.75600700 0.86622900 0.09344700
H -2.17457800 0.67505800 -1.43119200
H -1.57249000 -1.36370500 1.13437300

Nap-singlet-nitrene

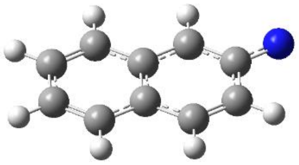

0 1
C 0.00000000 0.66820700 0.00000000
C 1.40551500 0.73667500 0.00000000
C -0.63215200 -0.60413400 0.00000000
H 1.90465800 1.69769300 0.00000000
C 2.18296400 -0.42205800 0.00000000
C 1.52616900 -1.70311100 0.00000000
H 2.14600400 -2.58921600 0.00000000

N 3.51674700 -0.33892000 0.00000000
C -2.04206200 -0.66525200 0.00000000
H -2.52268600 -1.63600300 0.00000000
C -2.79058700 0.48048300 0.00000000
H -3.87115400 0.42079900 0.00000000
C -2.16432300 1.74575400 0.00000000
H -2.77036900 2.64203300 0.00000000
C -0.80185400 1.83767900 0.00000000
H -0.31219300 2.80383000 0.00000000
C 0.17155100 -1.77828300 0.00000000
H -0.32281400 -2.74246600 0.00000000

Nap-TS$_{C4}$

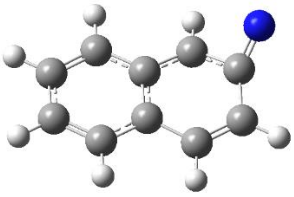

0 1
C 0.18528600 -0.63793000 -0.12720700
C -1.12532200 -1.14288100 -0.38642000
C 0.33984100 0.76964900 -0.02376800
H -1.31051800 -2.15599600 -0.70125400
C -2.23126200 -0.26643700 -0.06653300
C -2.07831100 1.18583000 -0.16063600
H -2.94389600 1.83242400 -0.18804600
N -2.90633200 -1.04606300 0.64696600
C 1.61971100 1.29844400 0.13026300
H 1.74026100 2.37230400 0.20929100
C 2.72894100 0.47128400 0.17675900
H 3.71482400 0.89827600 0.30463600
C 2.57981200 -0.91007100 0.04981100
H 3.45034500 -1.55202200 0.07806500
C 1.32312800 -1.45788100 -0.10478700
H 1.19795600 -2.52926500 -0.20182700
C -0.82072800 1.64708800 -0.13301600
H -0.63122100 2.71414900 -0.15641200

Nap-C4-azirine

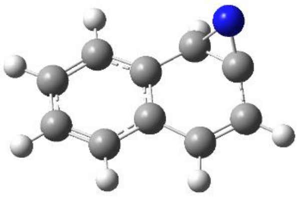

0 1
C 0.15118300 -0.65562700 -0.14675100
C -1.18576500 -1.24364900 -0.35648500
C 0.22495200 0.75339500 -0.01330600
H -1.32661800 -2.01671700 -1.10108100
C -2.22098200 -0.28147600 -0.02096900
C -2.21744100 1.15011900 -0.18883800
H -3.09695900 1.76724100 -0.29084800
N -2.19990400 -1.22225100 0.80060100

C 1.47938800 1.34856200 0.15639900
H 1.53975800 2.42494700 0.26704900
C 2.63261300 0.58785400 0.17403600
H 3.59344500 1.06535100 0.31363500
C 2.55716400 -0.79085100 -0.00398700
H 3.46059800 -1.38681500 -0.00318000
C 1.32646400 -1.40081000 -0.17371700
H 1.26277000 -2.47363200 -0.30950400
C -0.95701700 1.61459800 -0.14196900
H -0.77702800 2.68269600 -0.18675800

Nap-TS$_{C2}$

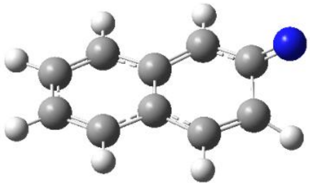

0 1
C 0.23578100 -0.70636400 -0.11603400
C -0.96545100 -1.38064400 -0.16690100
C 0.27522000 0.73655000 0.00869800
H -1.02145400 -2.45506500 -0.27224100
C -2.14129600 -0.57715300 -0.03694800
C -2.10892000 0.86243500 -0.38572600
H -2.97863100 1.35252800 -0.78378700
N -3.15602800 -0.40320900 0.67390700
C 1.53957500 1.37008700 0.16032700
H 1.56219700 2.44923600 0.25207400
C 2.69356300 0.65148000 0.17968400
H 3.64556900 1.15217600 0.29483300
C 2.66067400 -0.76609800 0.02963800
H 3.58957500 -1.32132600 0.03669100
C 1.48307600 -1.41733000 -0.12728400
H 1.45556400 -2.49424200 -0.23784700
C -0.89686800 1.48969400 -0.16473100
H -0.81275900 2.56321800 -0.29141200

C2-ketenimine

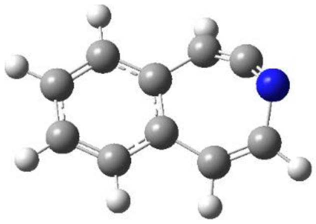

0 1
N 2.60342800 0.23825200 0.69335800
C -2.53655400 -0.87494300 0.17700000
C -1.28093600 -1.42591100 0.00369600
C -0.14128000 -0.62920200 -0.16149400
C -0.31255700 0.77565200 -0.15958200
C -1.58359600 1.32021700 0.01746700
C -2.69036800 0.50693100 0.17877200
C 1.13837500 -1.34553200 -0.36688600
C 0.86932700 1.63493500 -0.41451300

C 1.92007300 1.10849100 0.16018300
C 2.37874500 -0.98632700 -0.00896200
H 1.03265600 -2.34150200 -0.78251200
H -3.39630900 -1.51960500 0.30265100
H -1.16640900 -2.50332300 -0.00650700
H -1.69319200 2.39745200 0.00996800
H -3.67214200 0.94636600 0.29766300
H 0.86860200 2.47917700 -1.08449100
H 3.23542500 -1.63219800 -0.14436900

NH_3

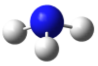

0 1
N   0.00000000   0.00000000   0.11116400
H   0.00000000   0.94215800 -0.25938200
H -0.81593300 -0.47107900 -0.25938200
H   0.81593300 -0.47107900 -0.25938200

4. SPECTROSCOPIC DATA

^{1}H NMR (400 MHz, $CDCl_3$) of desloratadine (see procedure)

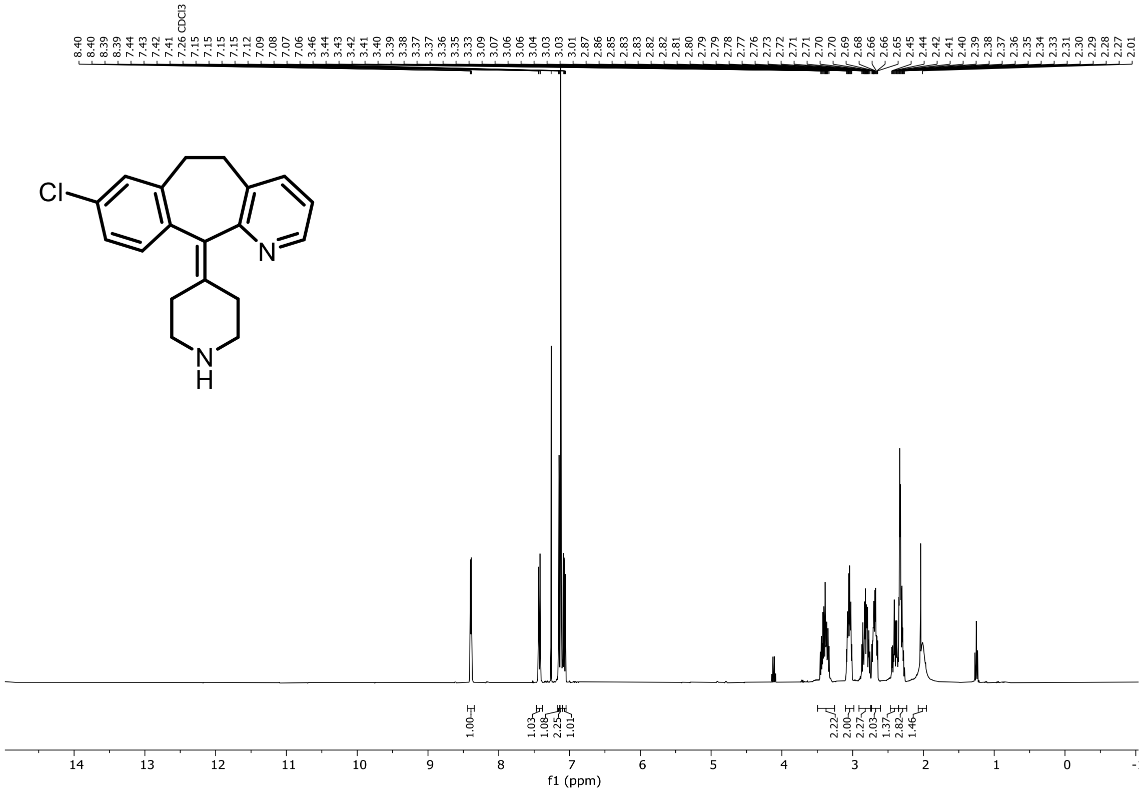

^{1}H NMR (400 MHz, $CDCl_3$) of 2,3,4-trimethoxybenzaldehyde (see procedure)

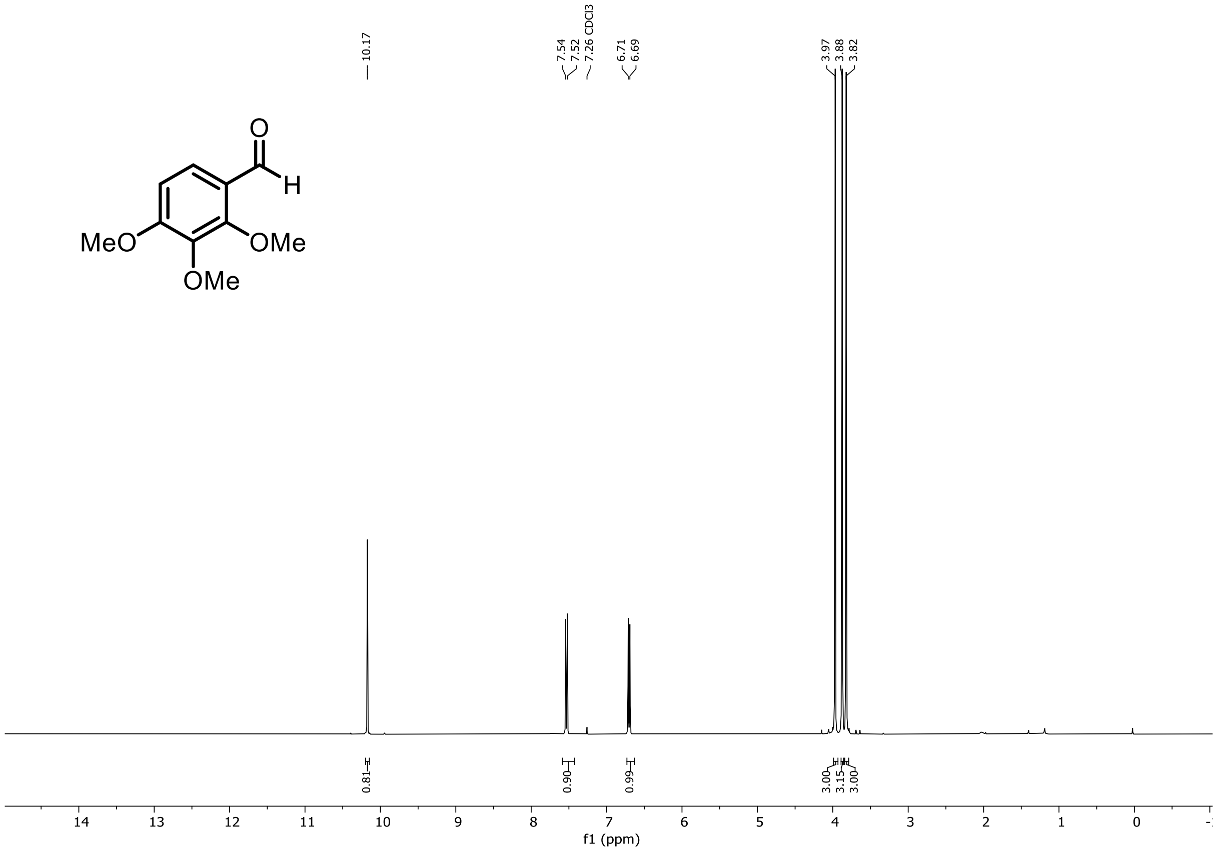

^{1}H NMR (400 MHz, $CDCl_3$) of tert-butyl 4-(2,3,4-trimethoxybenzyl)piperazine-1-carboxylate (see procedure)

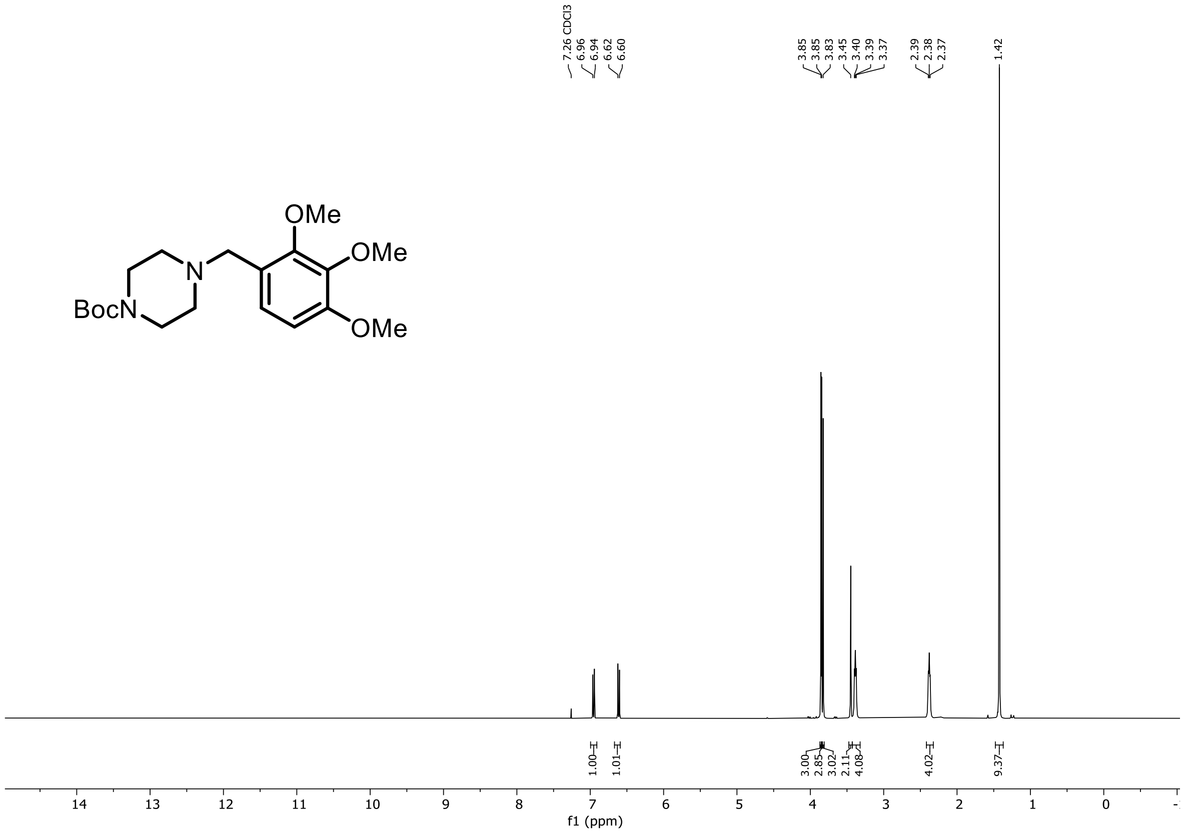

^{1}H NMR (400 MHz, $CDCl_3$) of trimetazidine (see procedure)

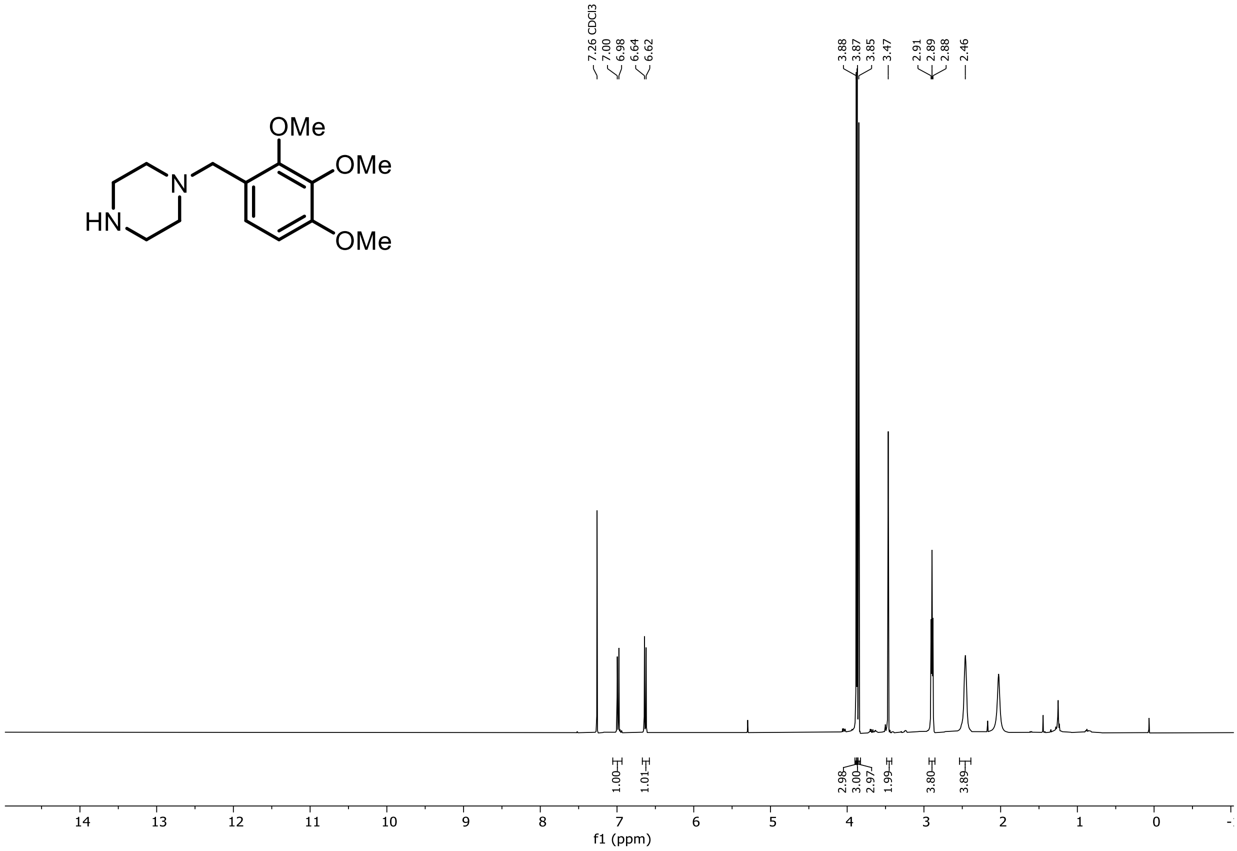

^1H NMR (400 MHz, $CDCl_3$) of MNFO (see procedure)

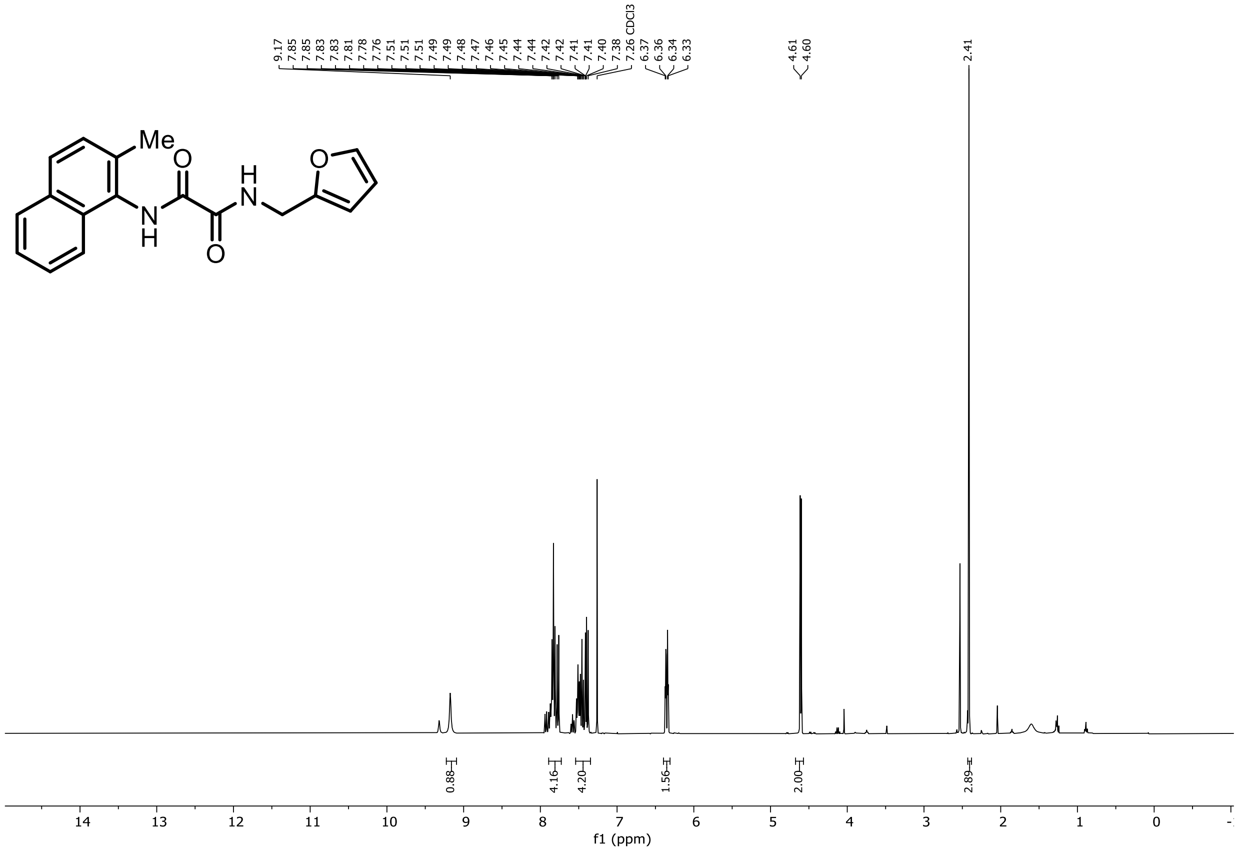

^1H NMR (400 MHz, DMSO-d_6) of 7-chloro-3-nitroquinolin-4-ol (see procedure)

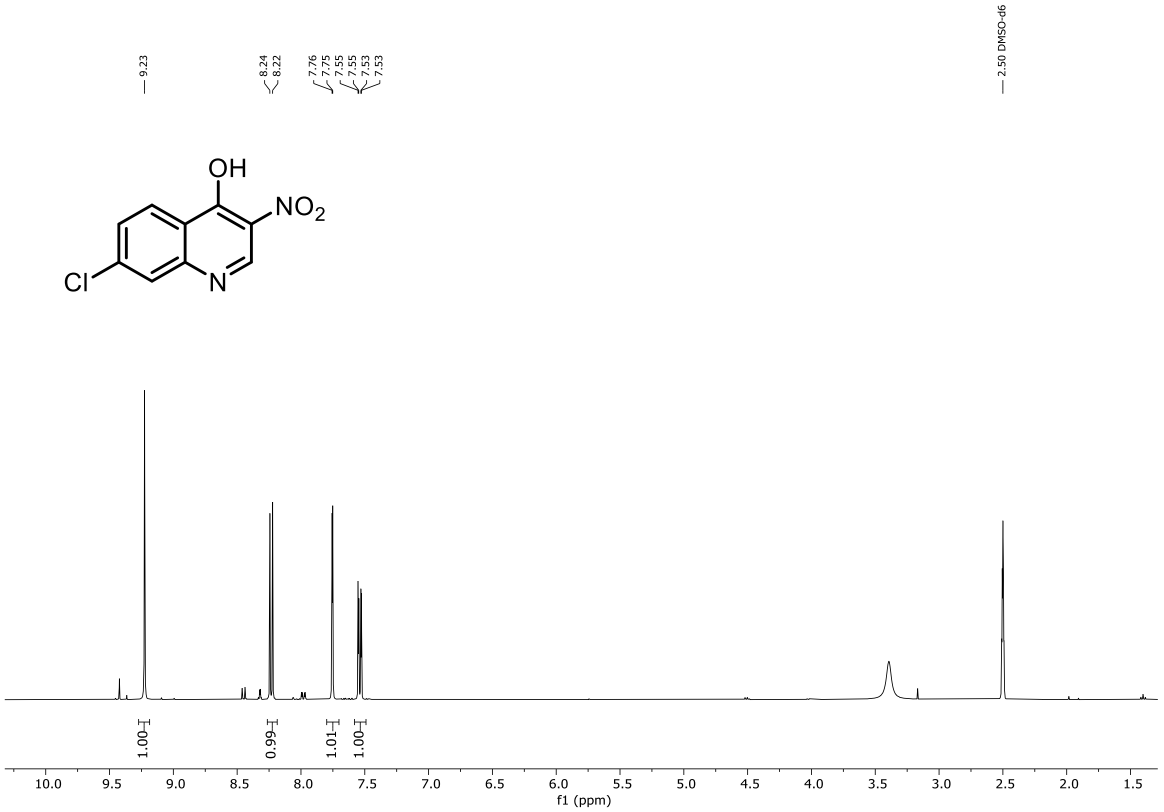

^{1}H NMR (400 MHz, $CDCl_3$) of 4,7-dichloro-3-nitroquinoline (see procedure)

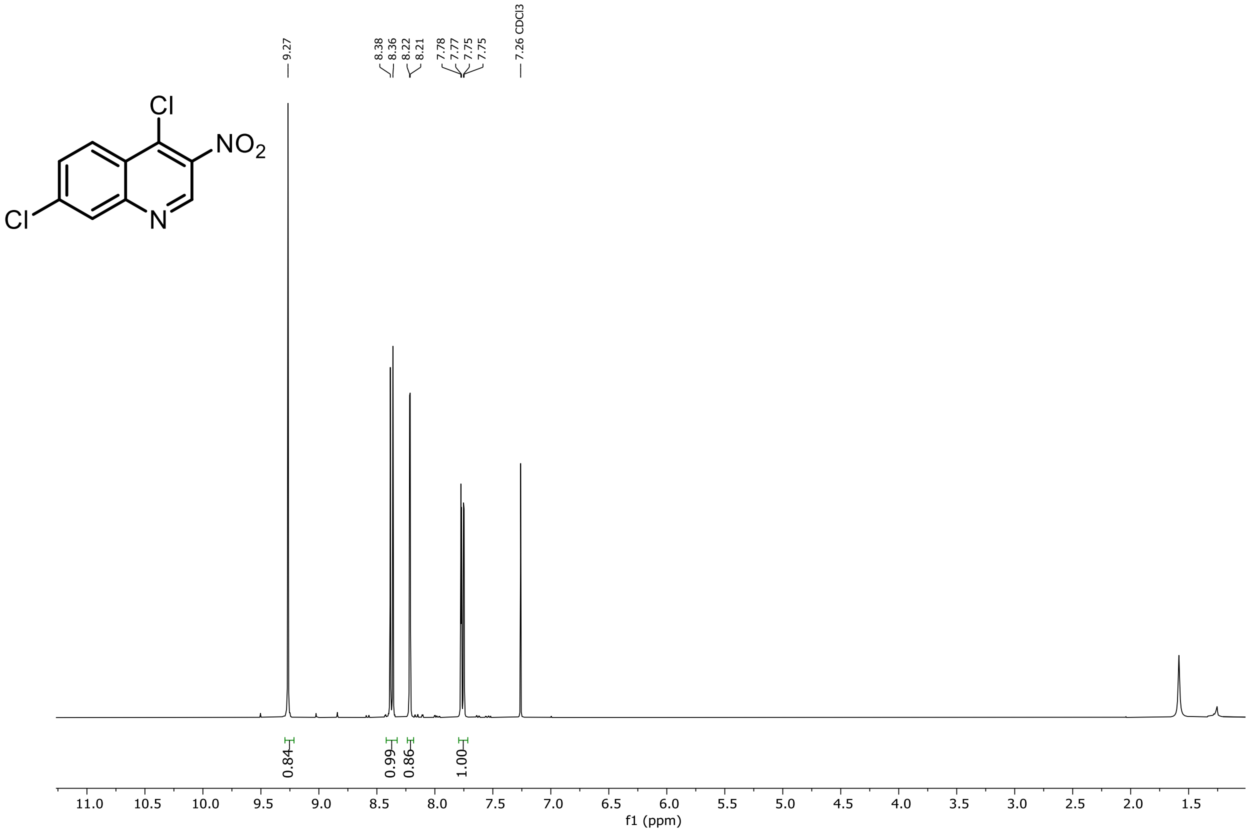

^{1}H NMR (400 MHz, $CDCl_3$) of 7-chloro-3-nitroquinoline (see procedure)

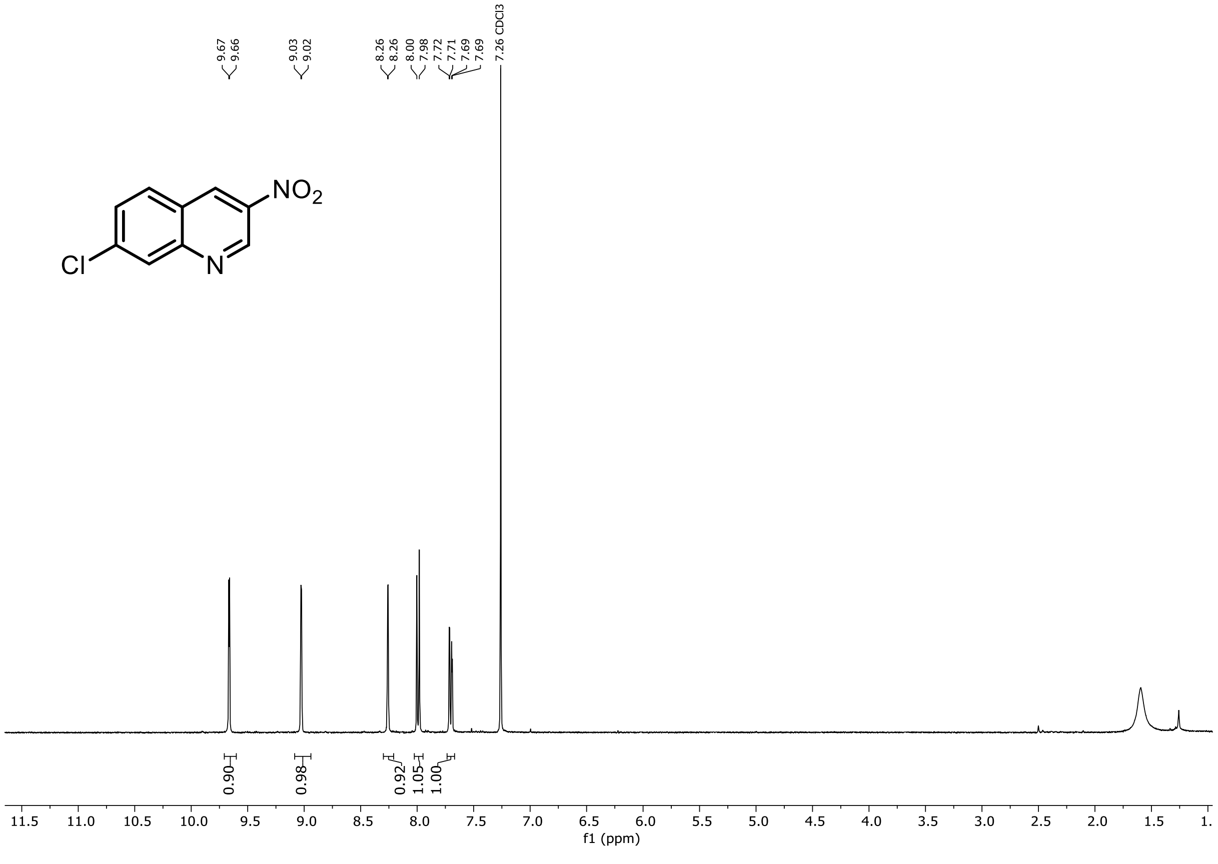

^{1}H NMR (400 MHz, $CDCl_3$) of 7-chloroquinolin-3-amine (see procedure)

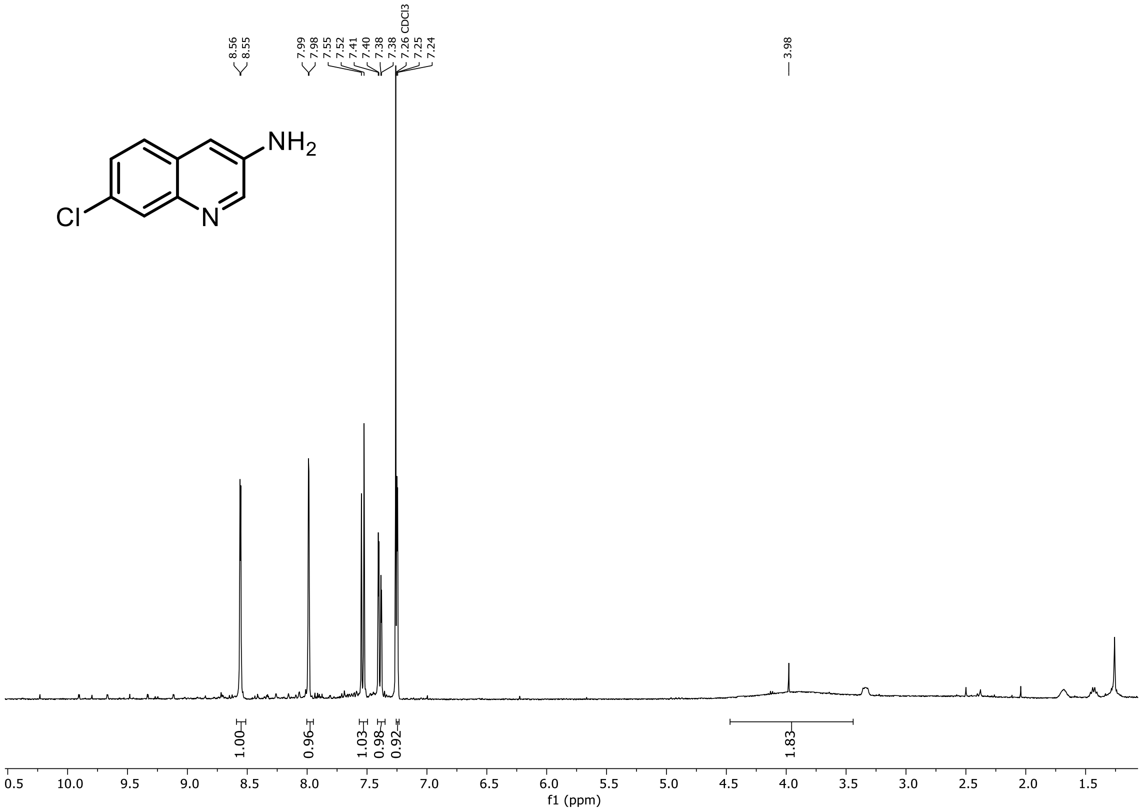

^{1}H NMR (400 MHz, $CDCl_3$) of 1*H*-imidazole-1-sulfonyl azide hydrochloride (see procedure)

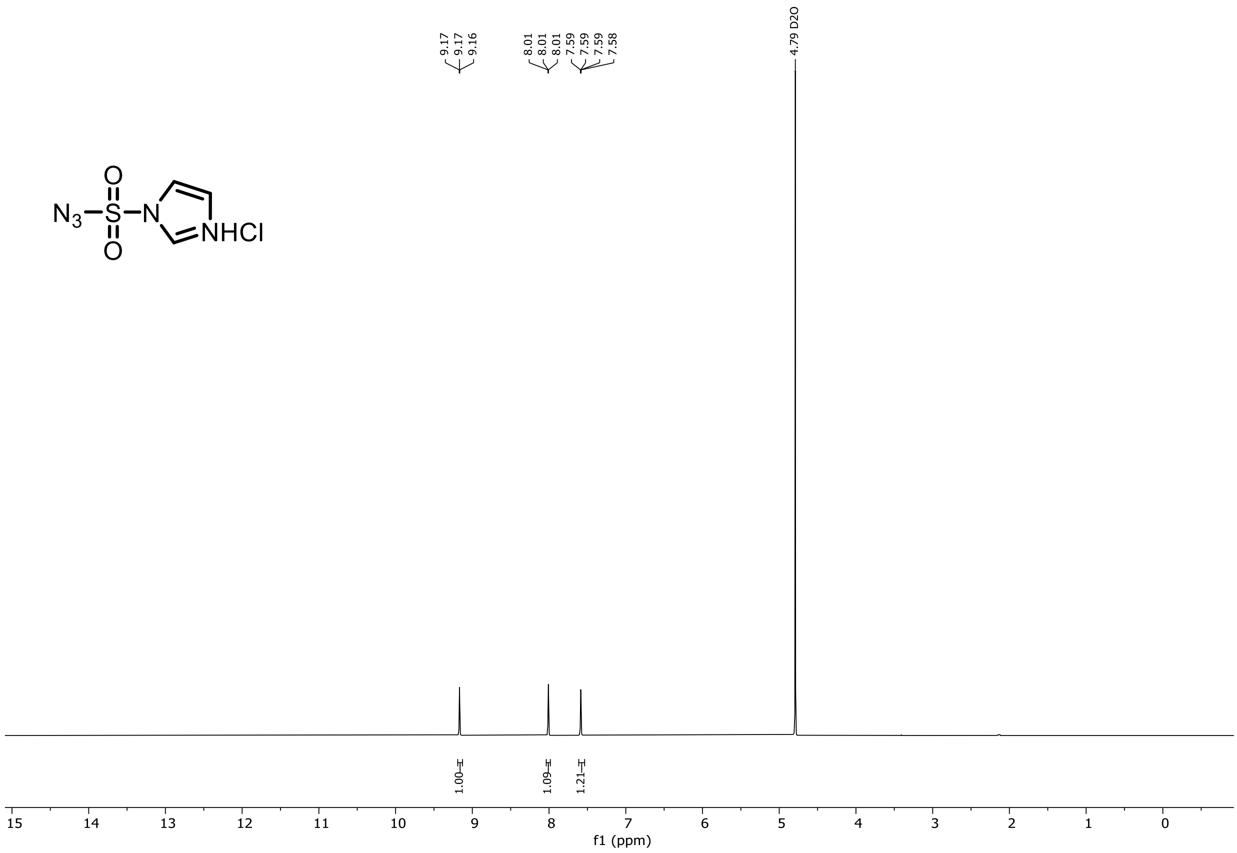

^{1}H NMR (400 MHz, $CDCl_3$) of Az-1 (see procedure)

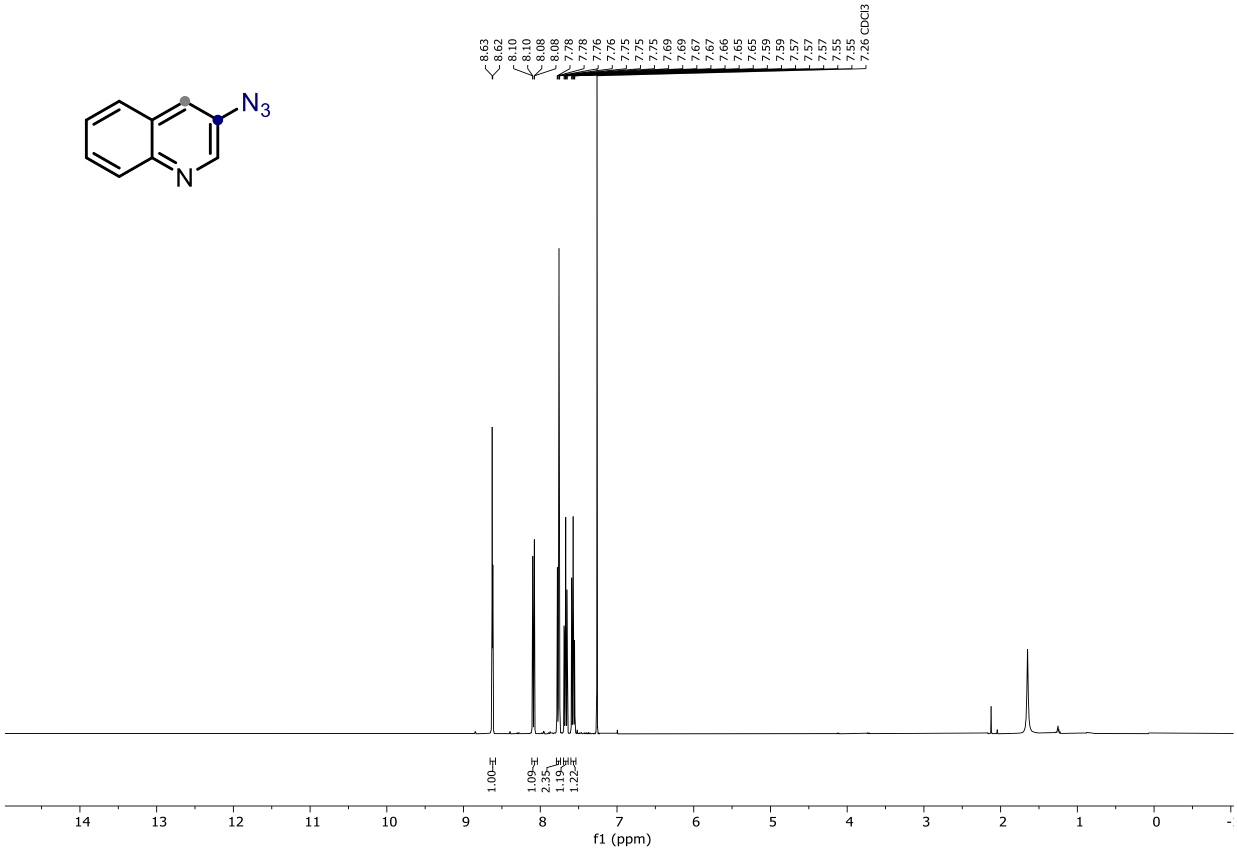

^{1}H NMR (400 MHz, $CDCl_3$) of Az-2 (see procedure)

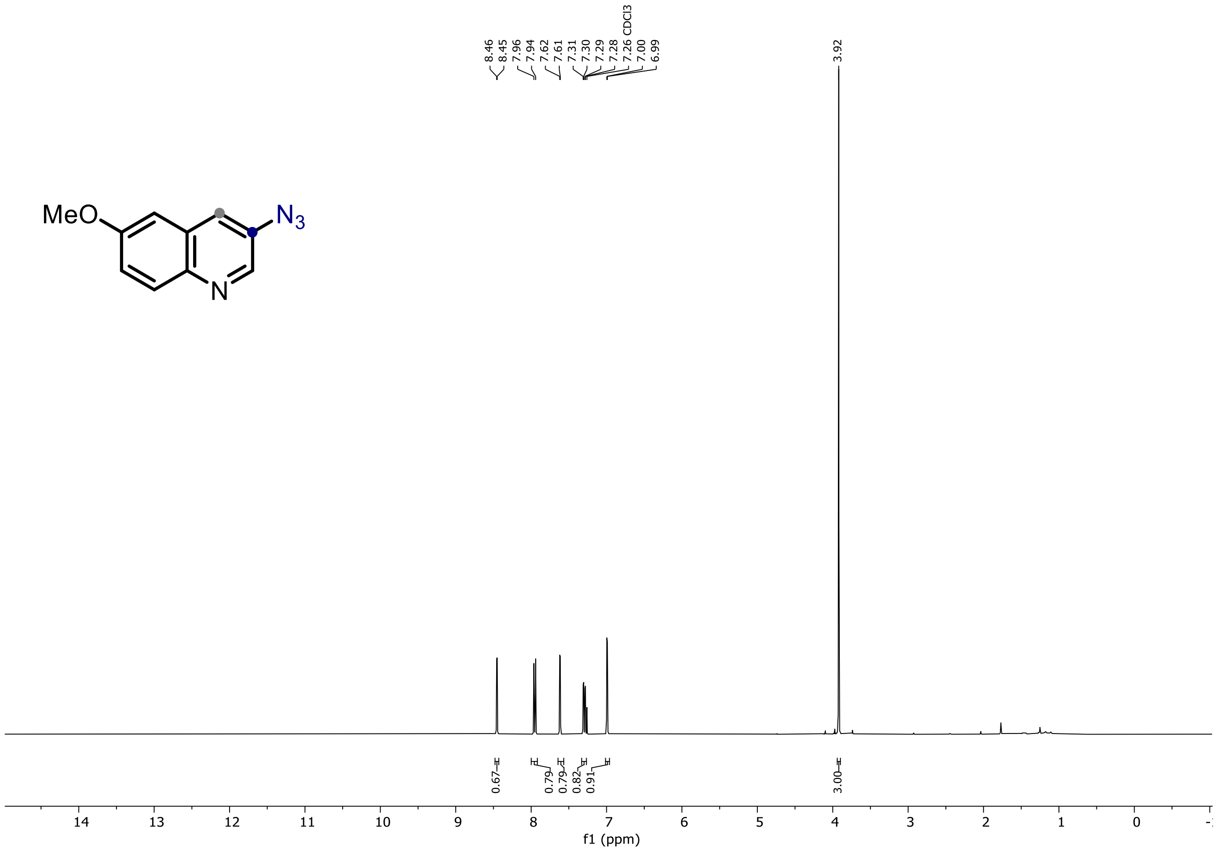

$^{13}C\{^1H\}$ NMR (101 MHz, $CDCl_3$) of Az-2

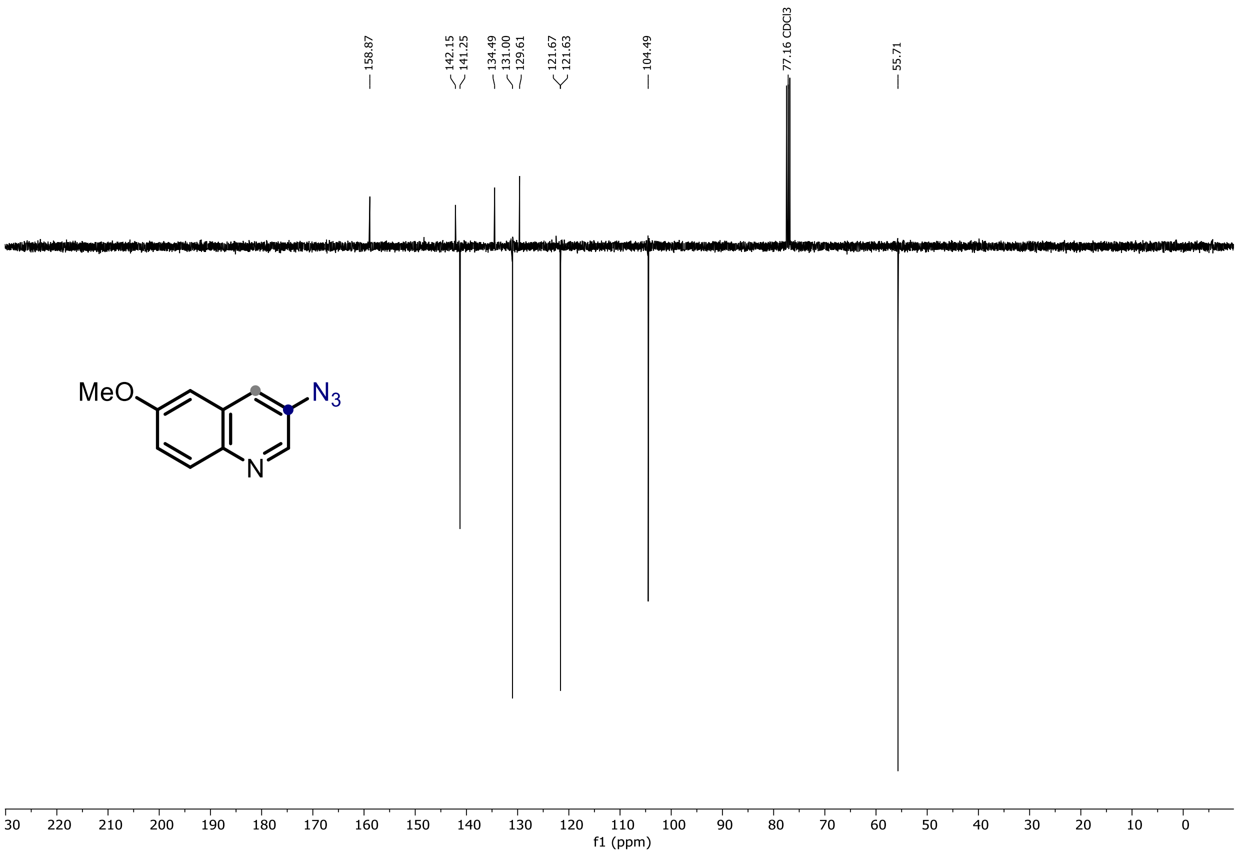

^1H NMR (400 MHz, $CDCl_3$) of Az-3 (see procedure)

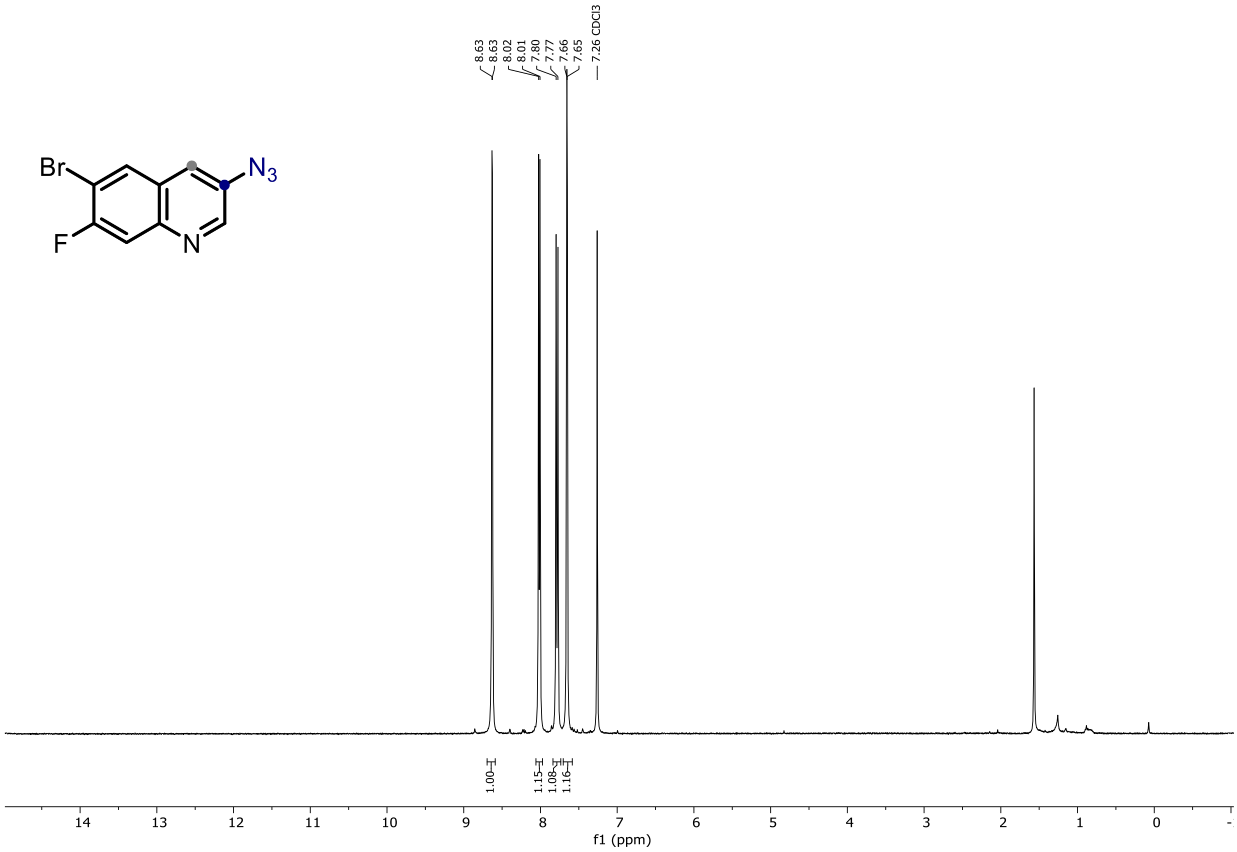

$^{13}C\{^1H\}$ NMR (101 MHz, $CDCl_3$) of Az-3

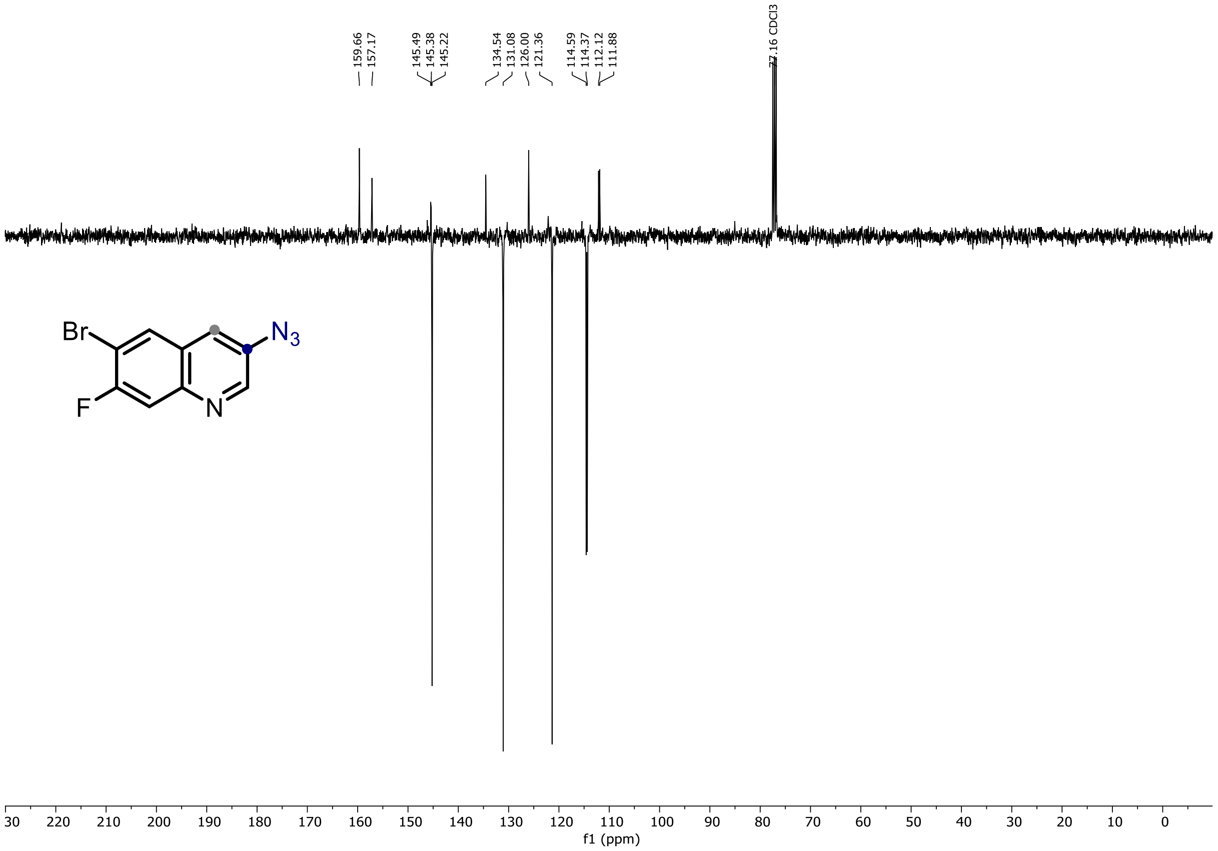

^{19}F NMR (376 MHz, $CDCl_3$) of Az-3

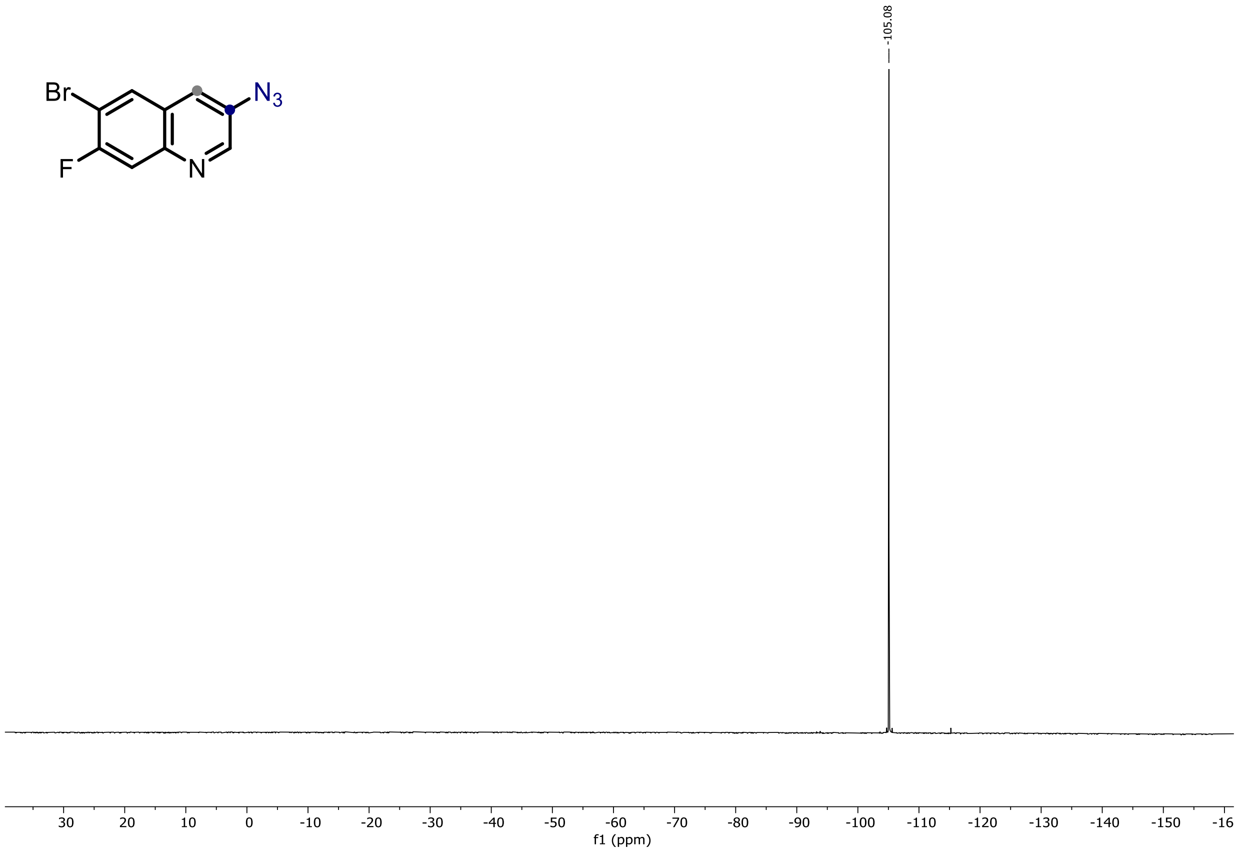

^{1}H NMR (400 MHz, $CDCl_3$) of Az-4 (see procedure)

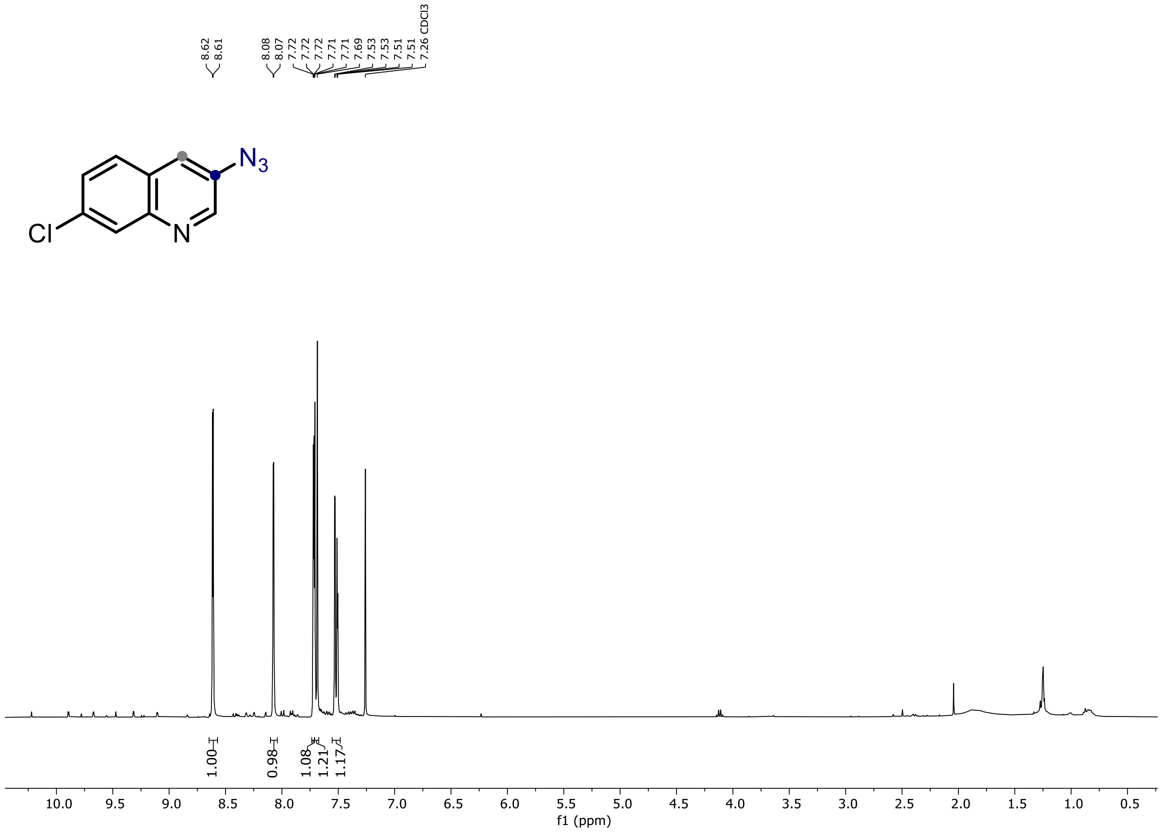

^{13}C{^{1}H} NMR (101 MHz, $CDCl_3$) of Az-4

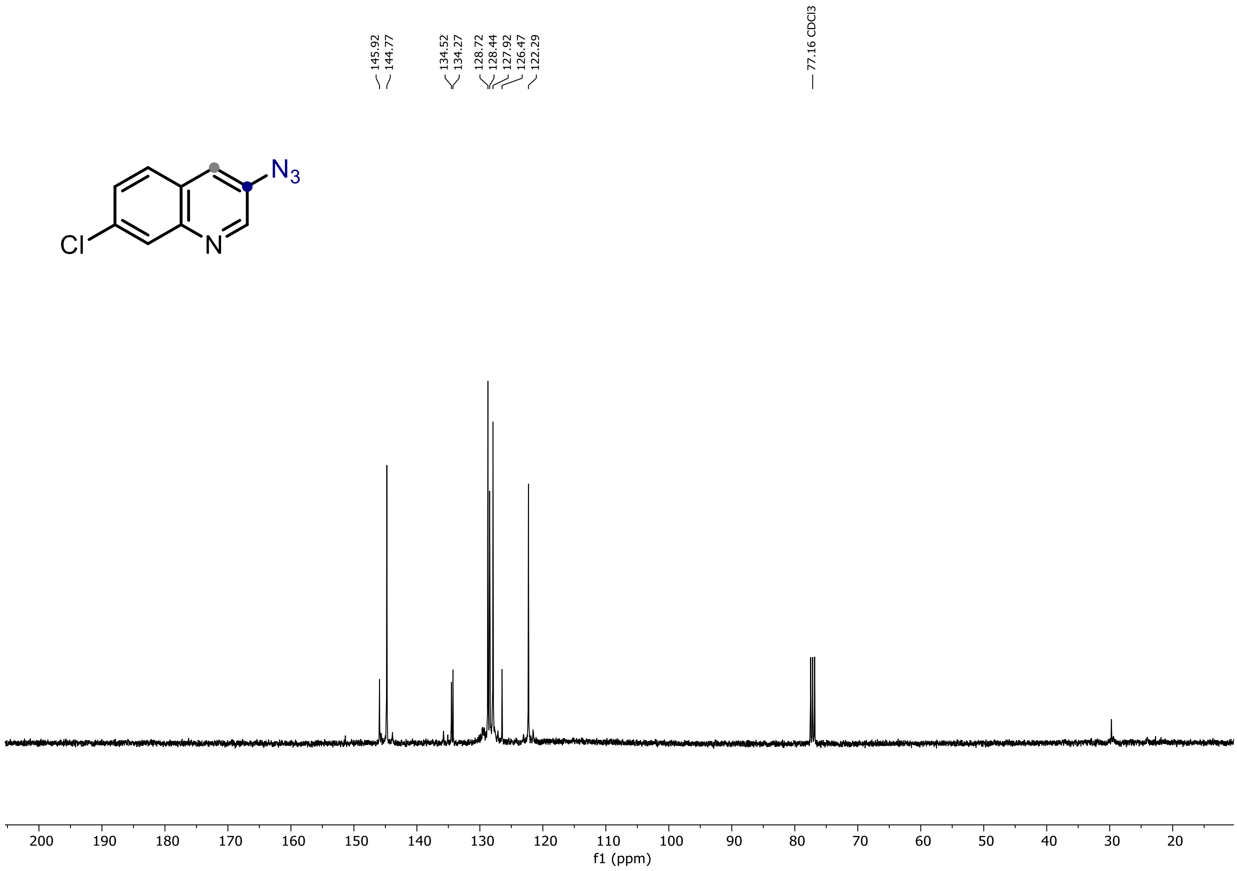

^{1}H NMR (400 MHz, $CDCl_3$) of Az-5 (see procedure)

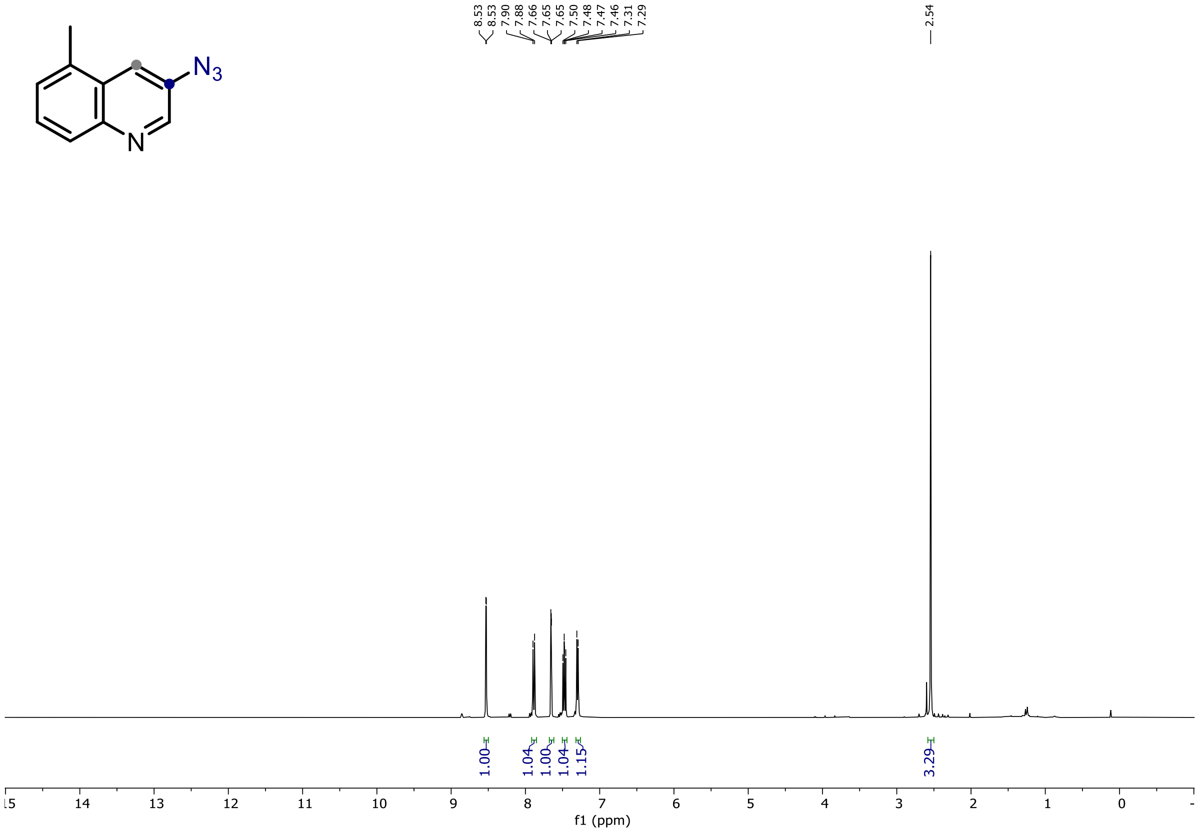

^{13}C{^{1}H} NMR (101 MHz, $CDCl_3$) of Az-5

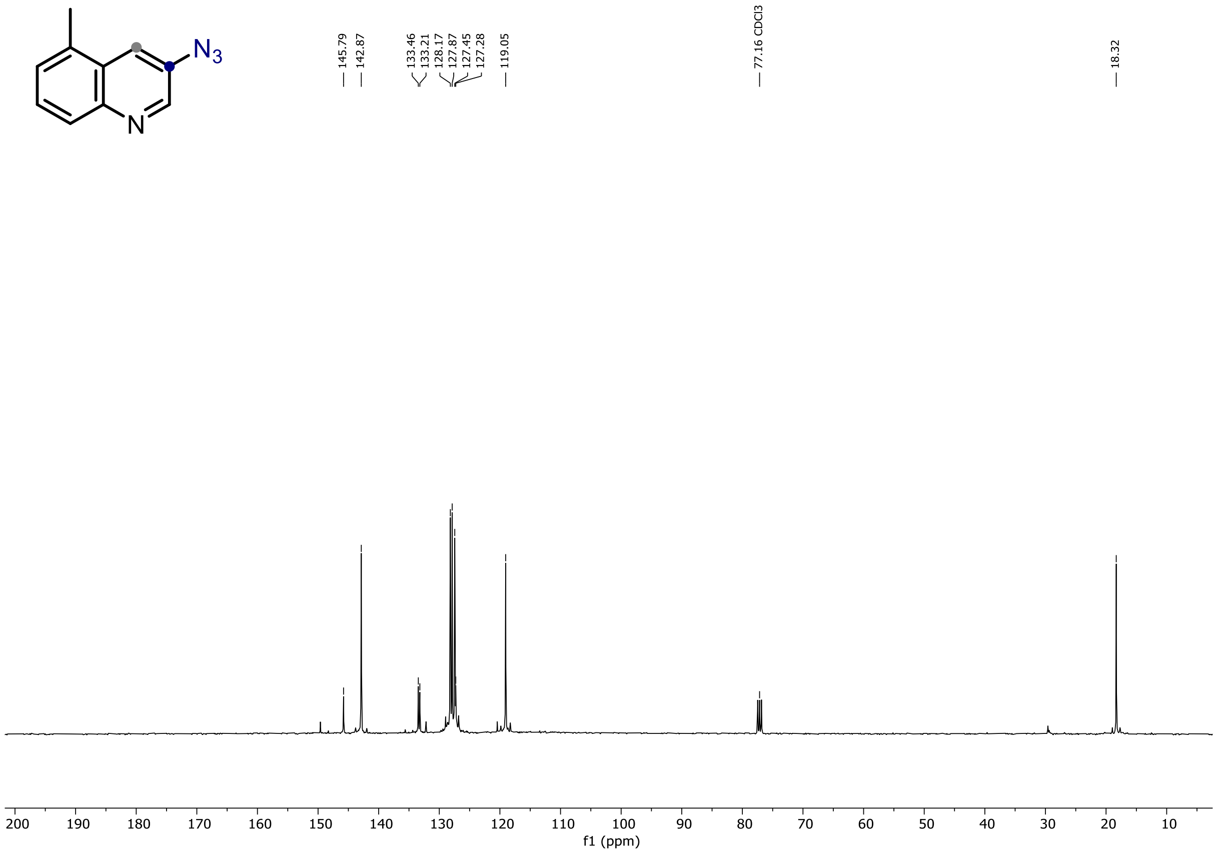

^{1}H NMR (400 MHz, $CDCl_3$) of Az-6 (see procedure)

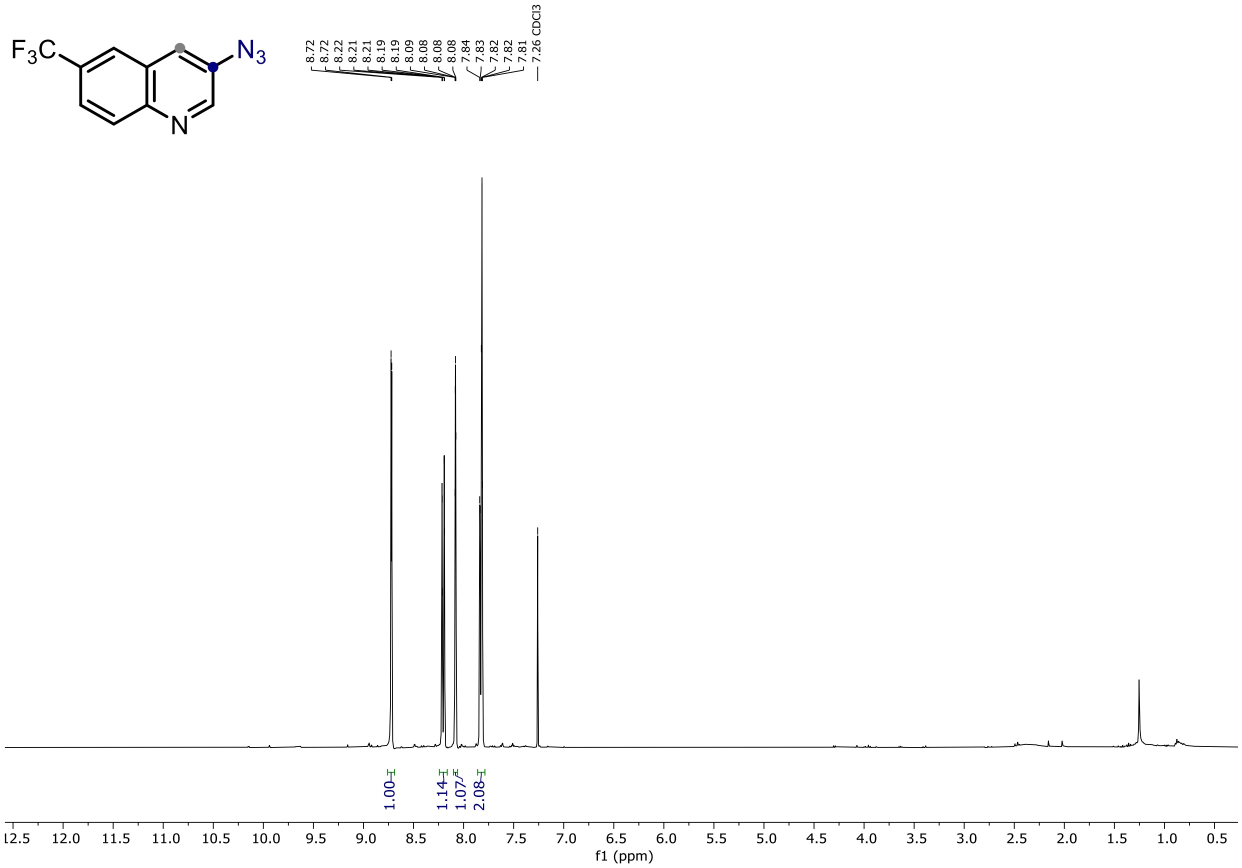

$^{13}C\{^{1}H\}$ NMR (400 MHz, $CDCl_3$) of Az-6

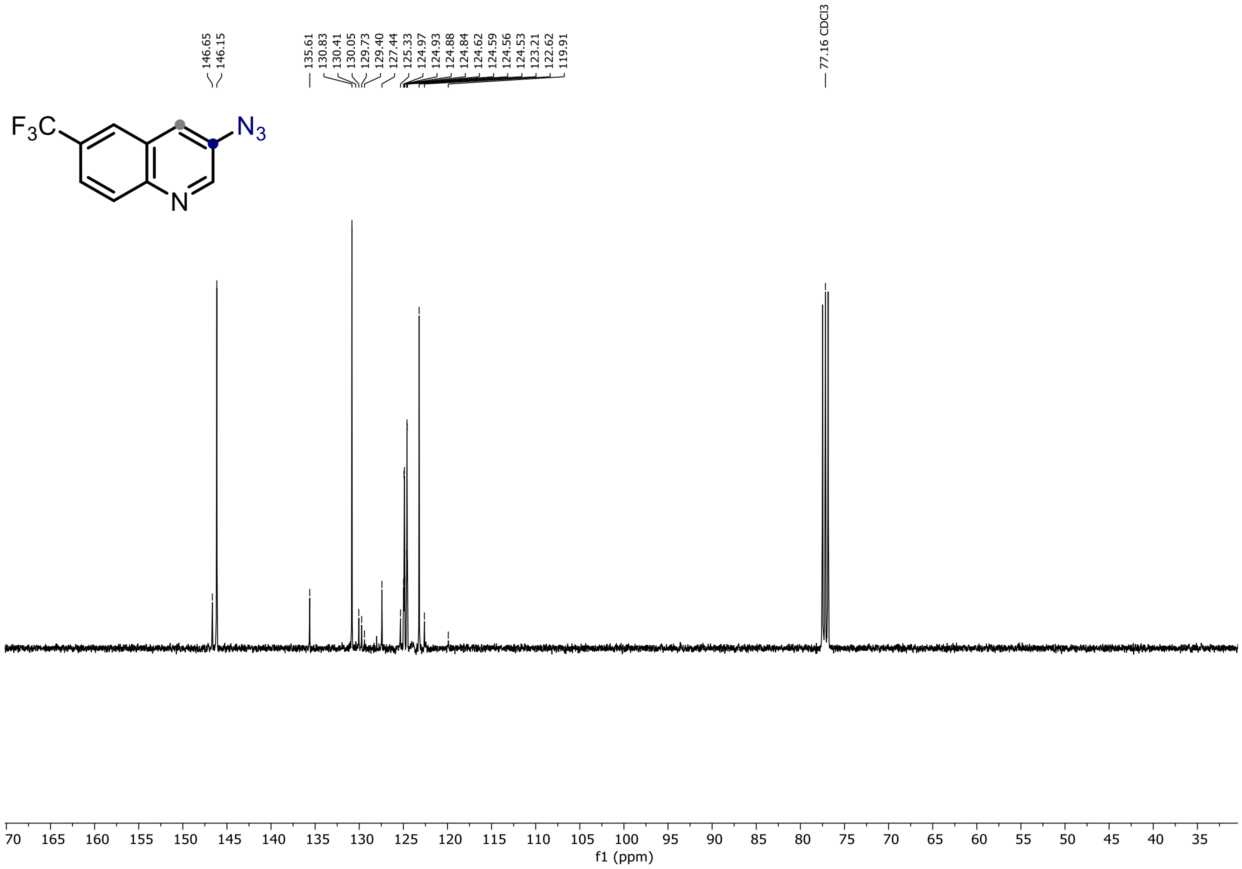

^{19}F NMR (376 MHz, $CDCl_3$) of Az-6

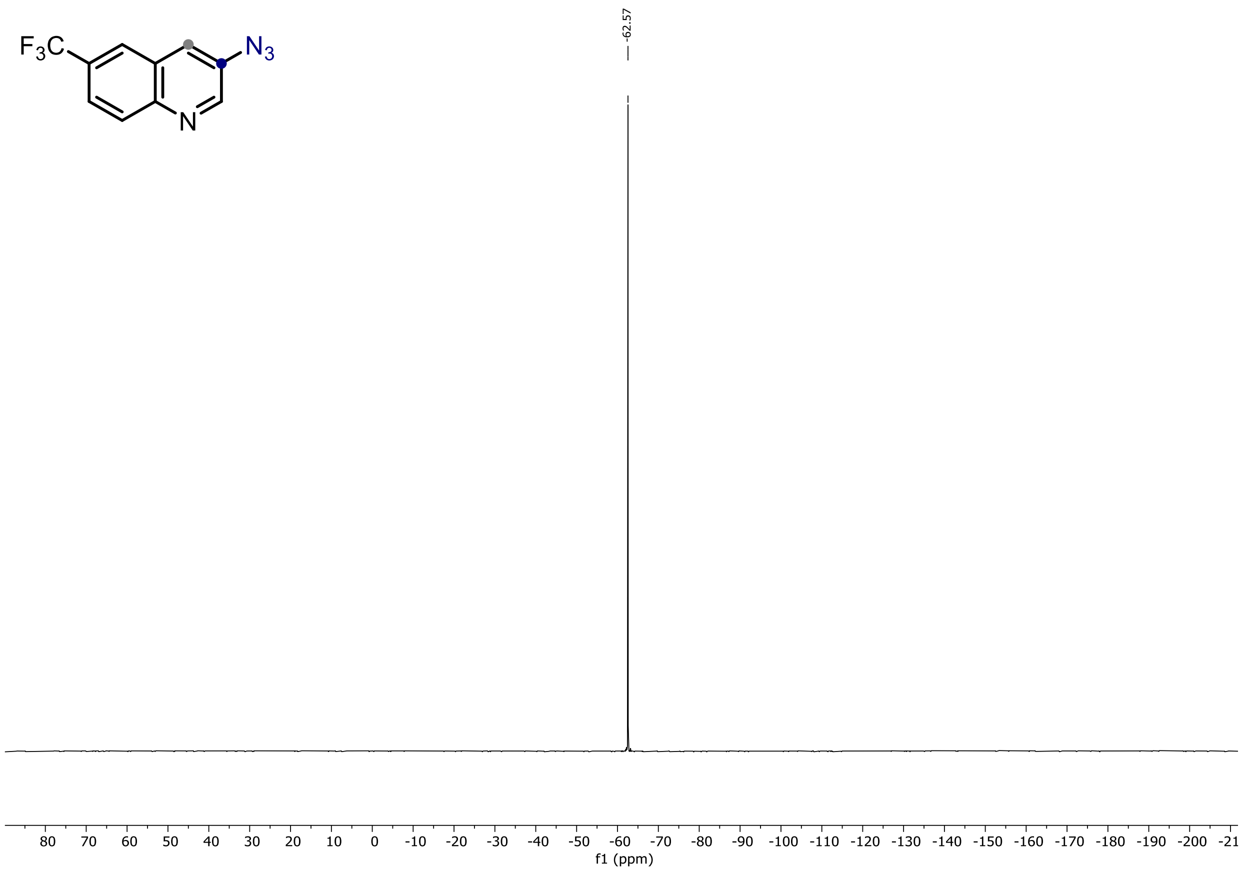

^{1}H NMR (400 MHz, $CDCl_3$) of Az-7 (see procedure)

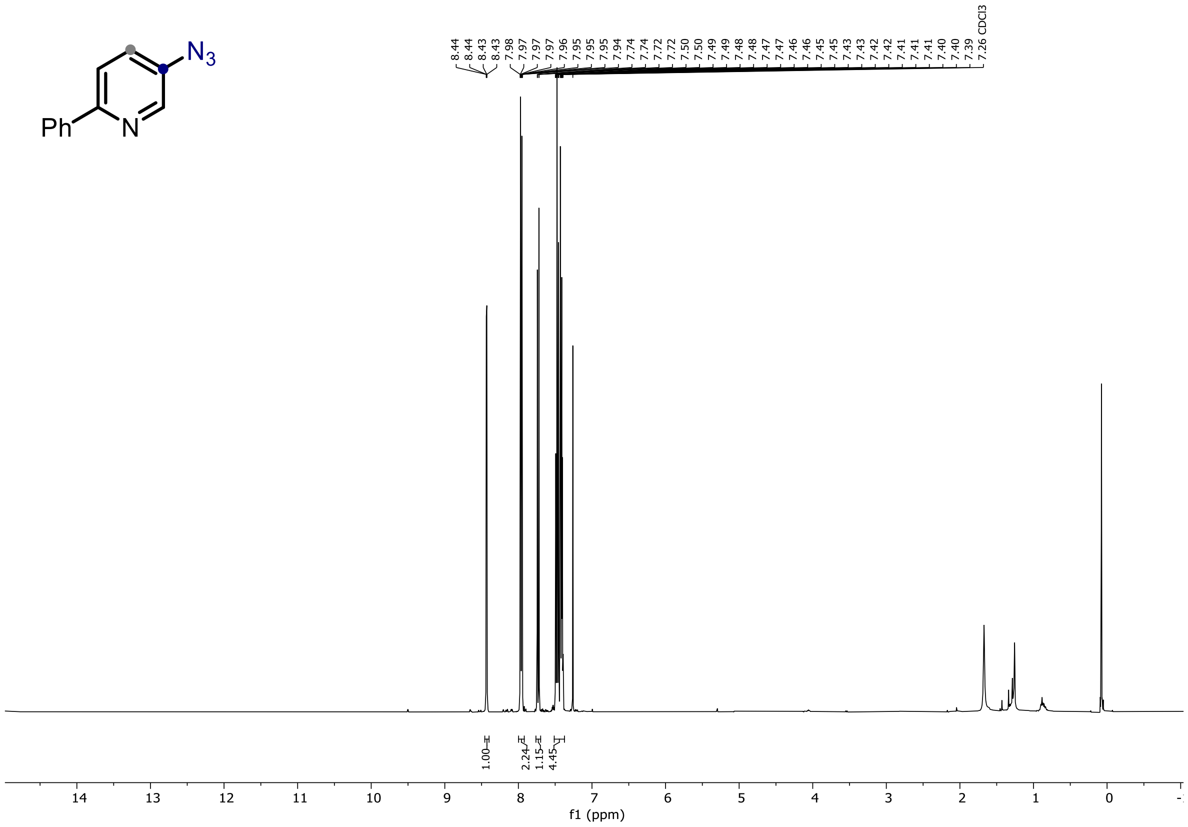

^{1}H NMR (400 MHz, $CDCl_3$) of Az-8 (see procedure)

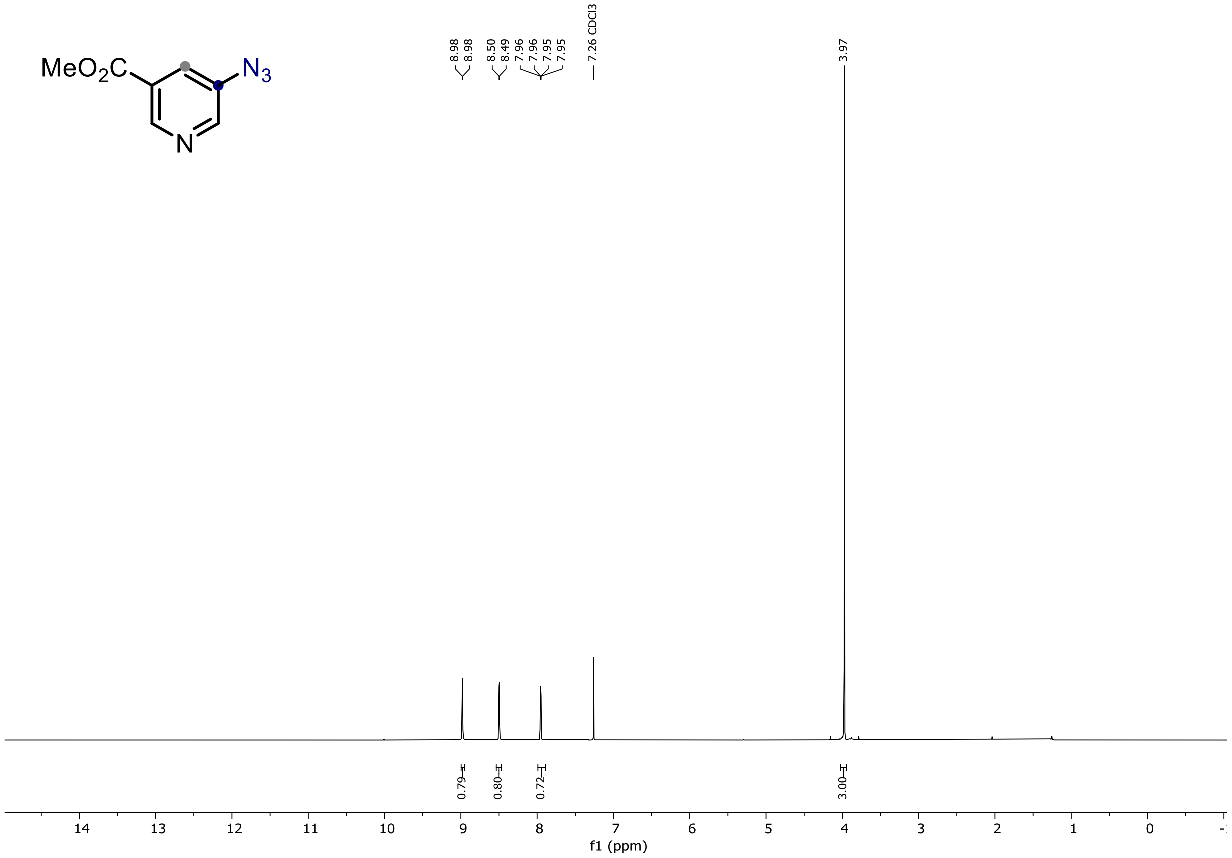

^{13}C{^{1}H} NMR (101 MHz, $CDCl_3$) of Az-8

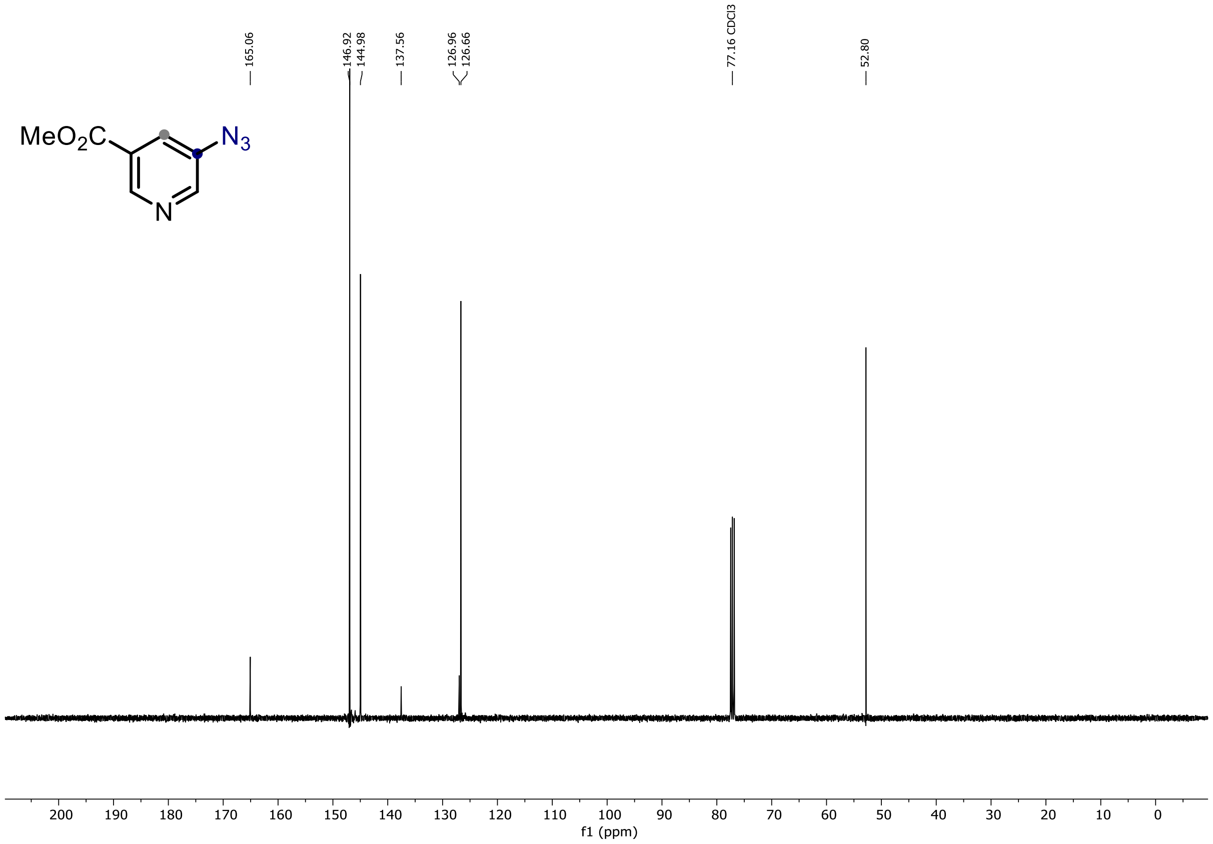

^{1}H NMR (400 MHz, $CDCl_3$) of 1 (see procedure)

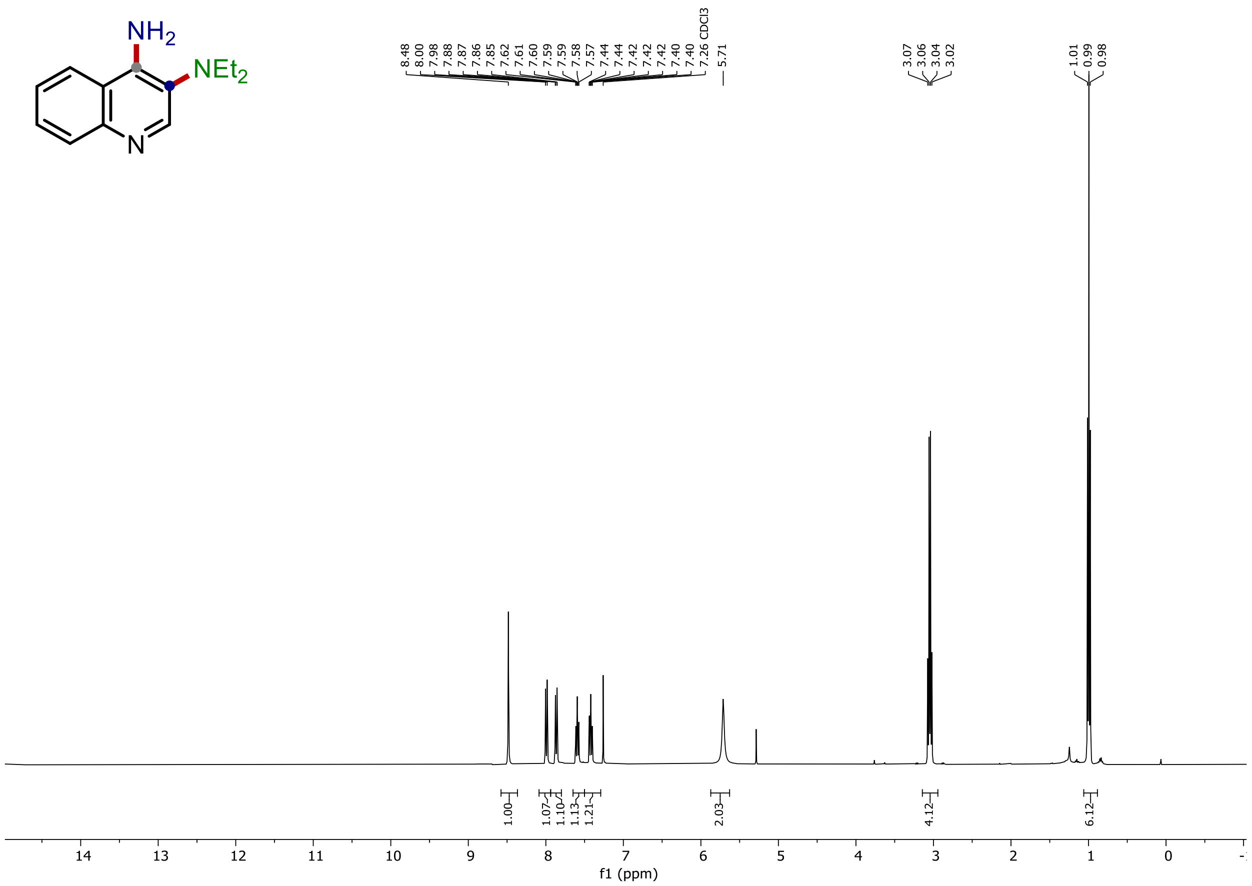

^{13}C{^{1}H} NMR (101 MHz, $CDCl_3$) of 1

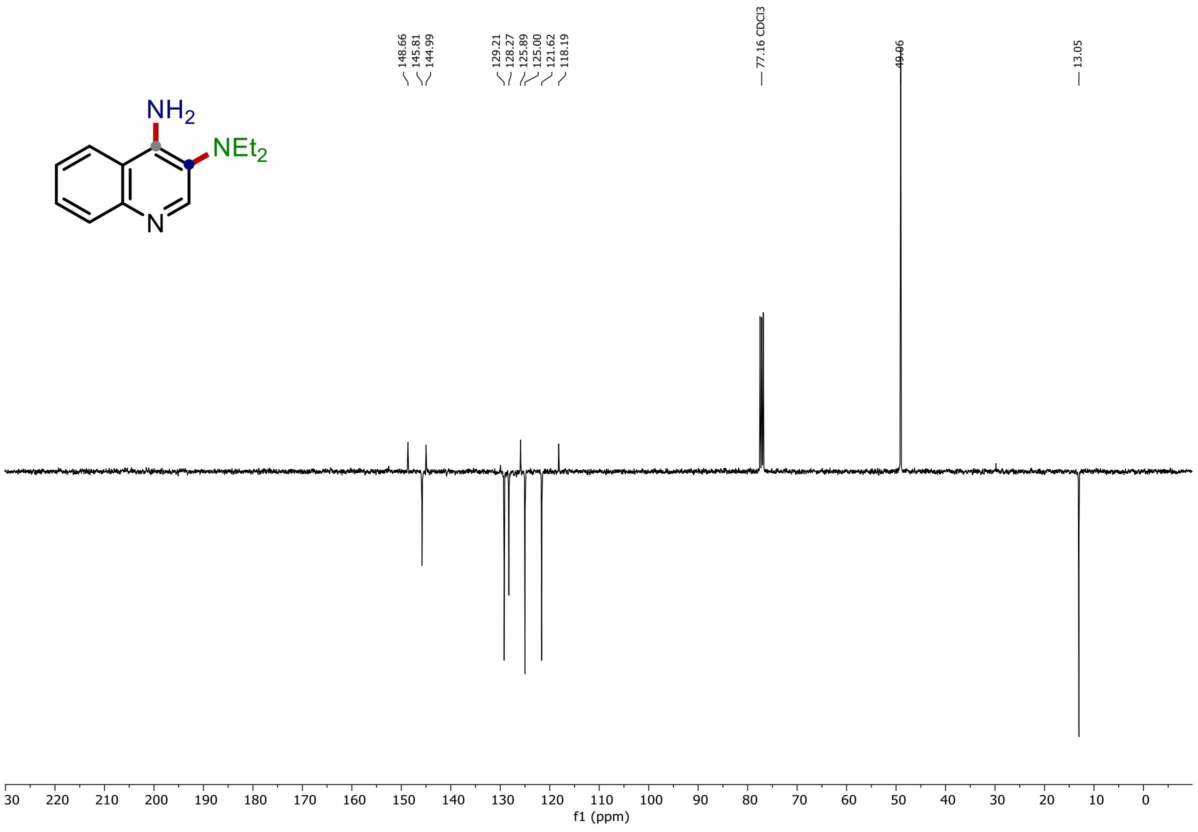

^{1}H NMR (400 MHz, $CDCl_3$) of 2 (see procedure)

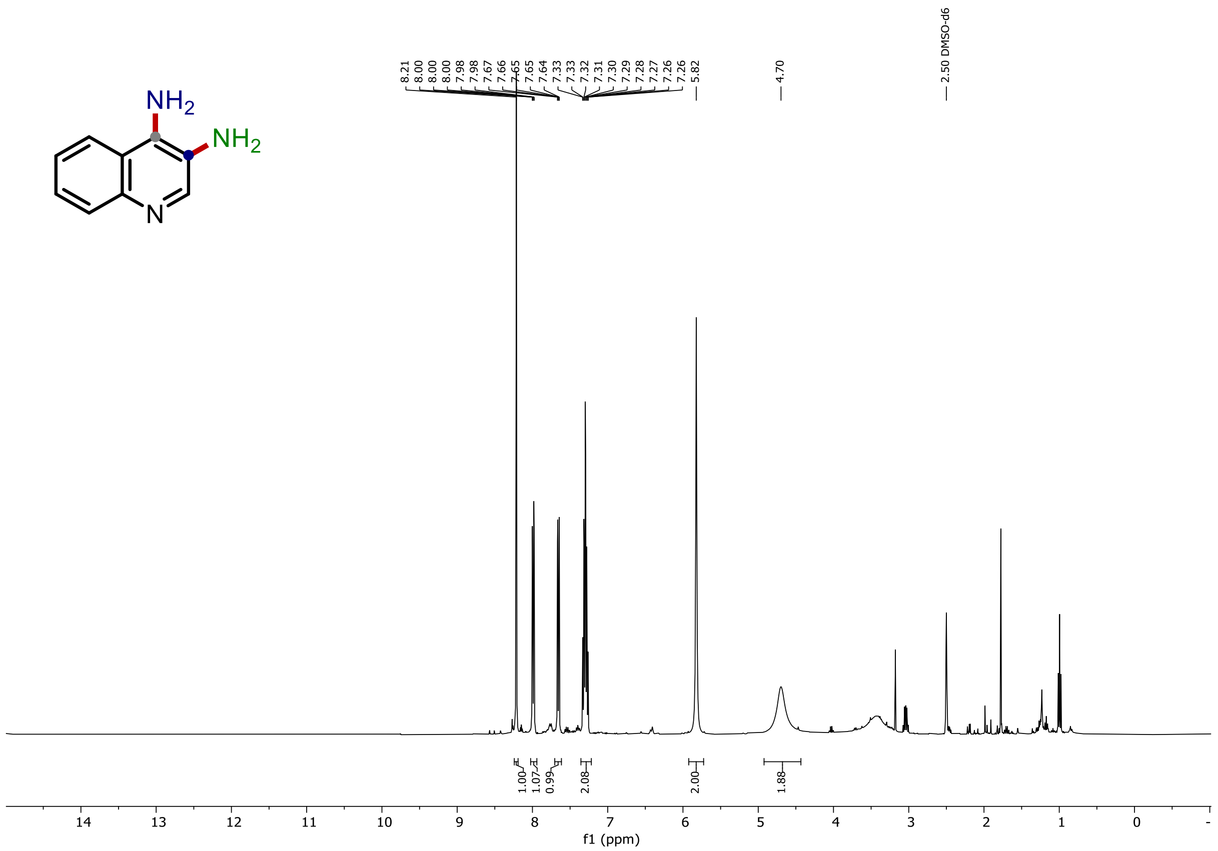

^{1}H NMR (400 MHz, $CDCl_3$) of 3 (see procedure)

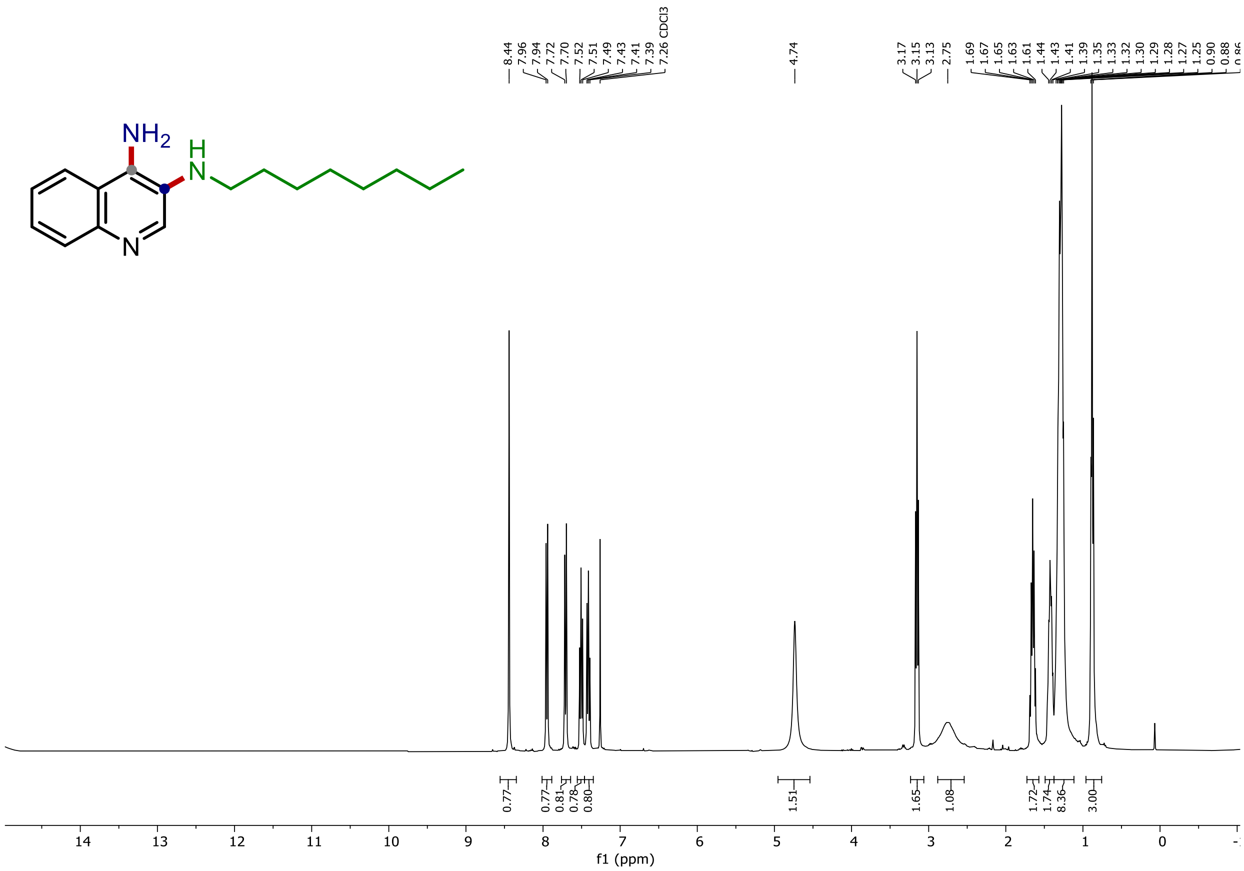

$^{13}C\{^1H\}$ NMR (101 MHz, $CDCl_3$) of 3

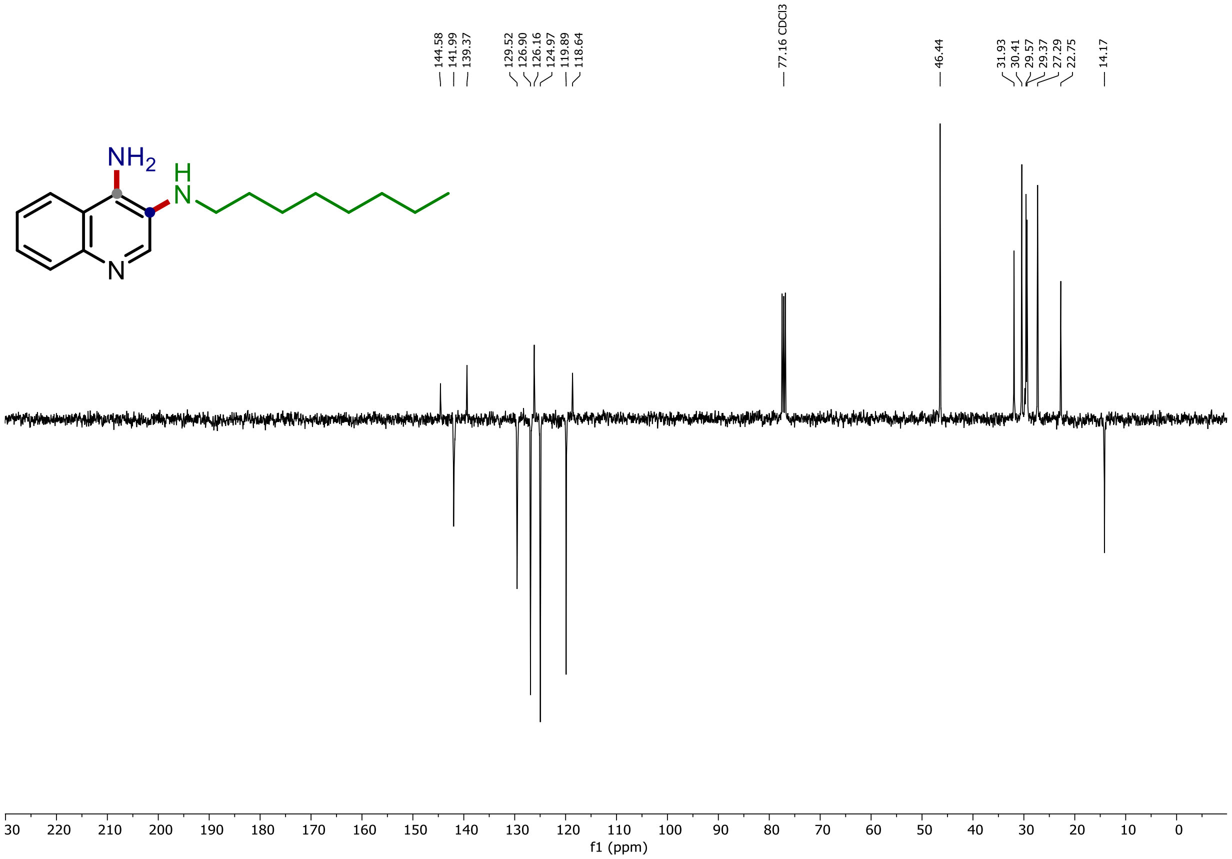

^1H NMR (400 MHz, $CDCl_3$) of 4 (see procedure)

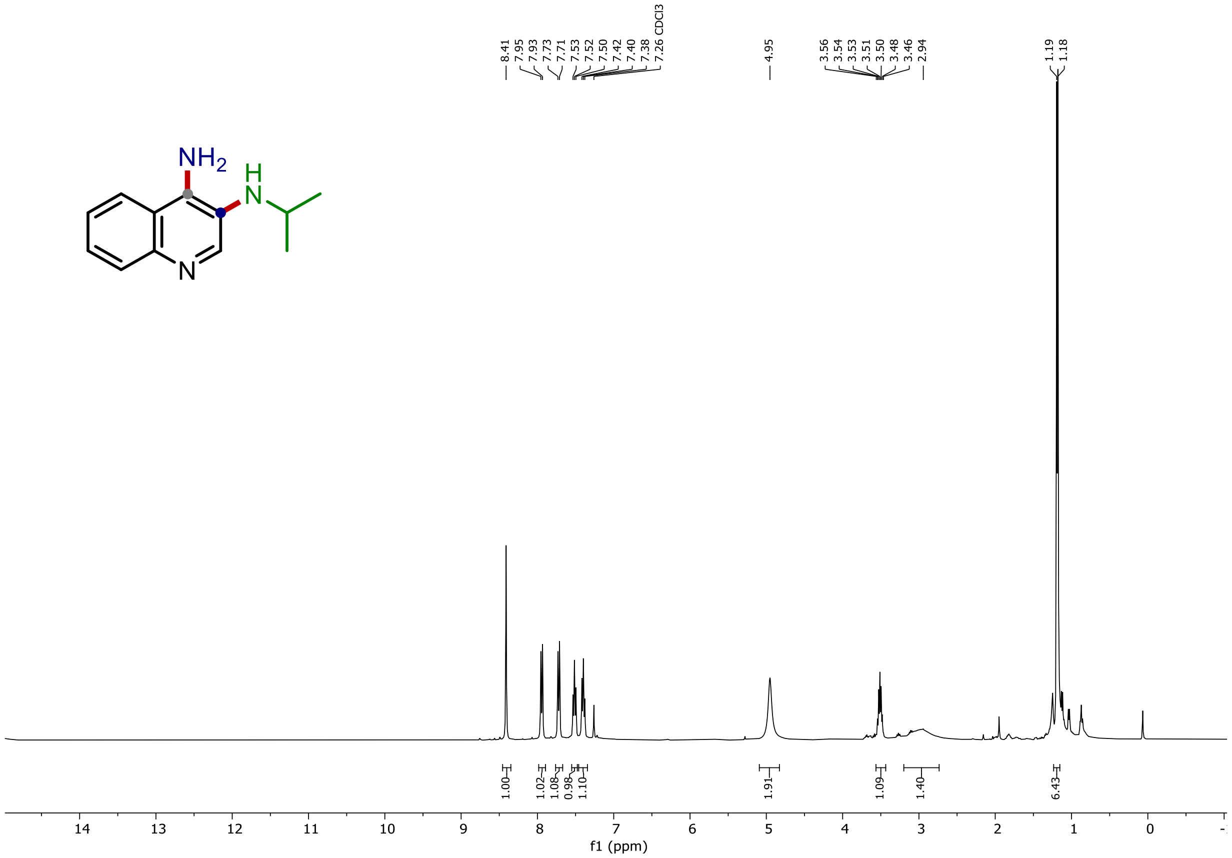

$^{13}C\{^1H\}$ NMR (101 MHz, $CDCl_3$) of 4

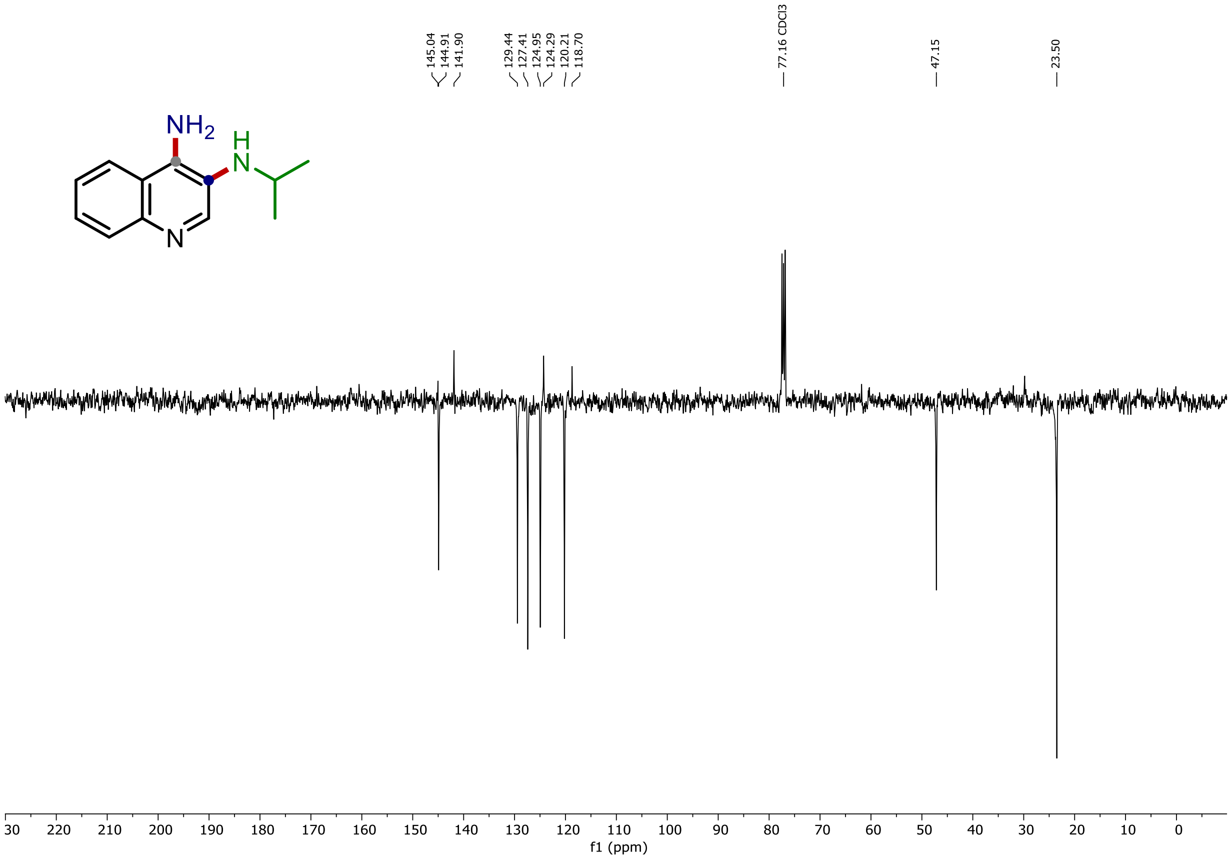

^1H NMR (400 MHz, $CDCl_3$) of 5 (see procedure)

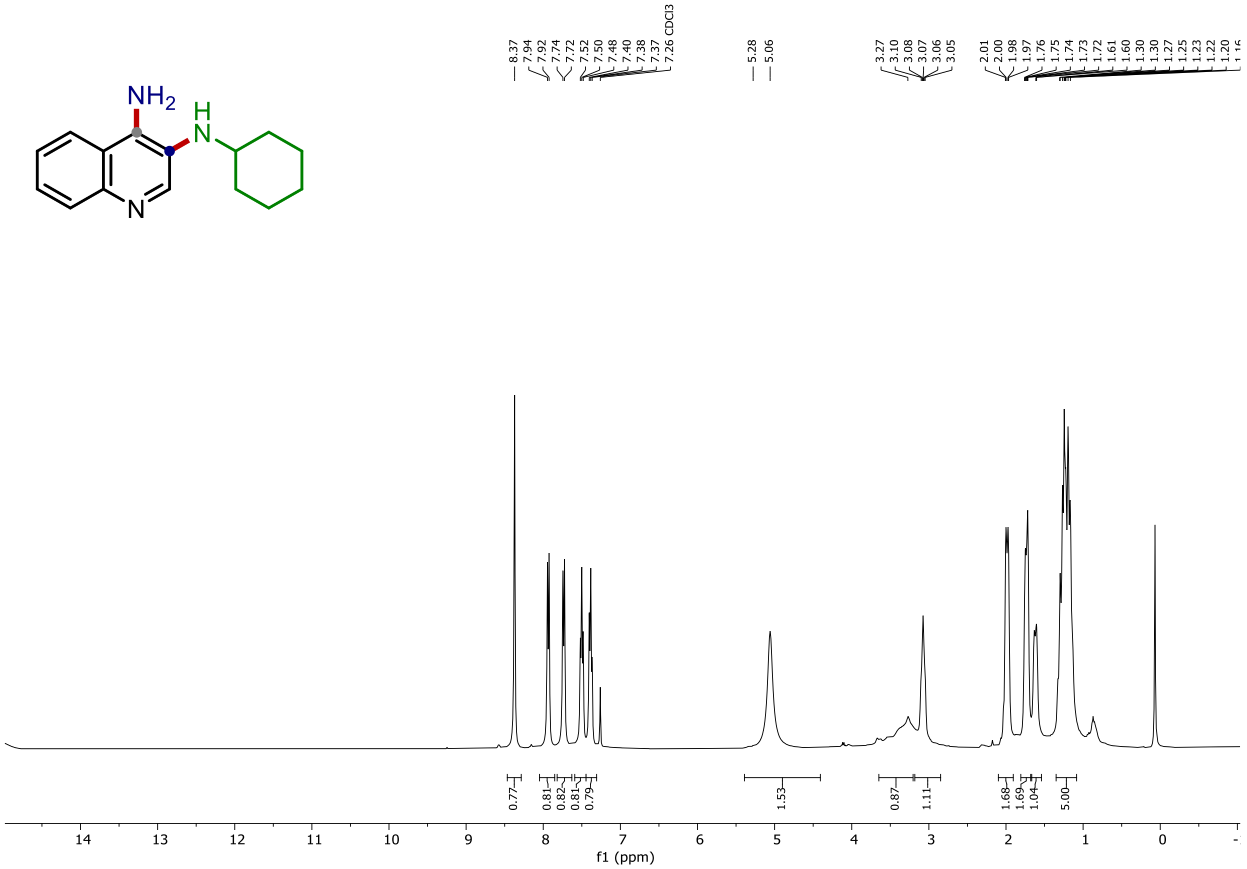

$^{13}C\{^{1}H\}$ NMR (101 MHz, $CDCl_3$) of 5

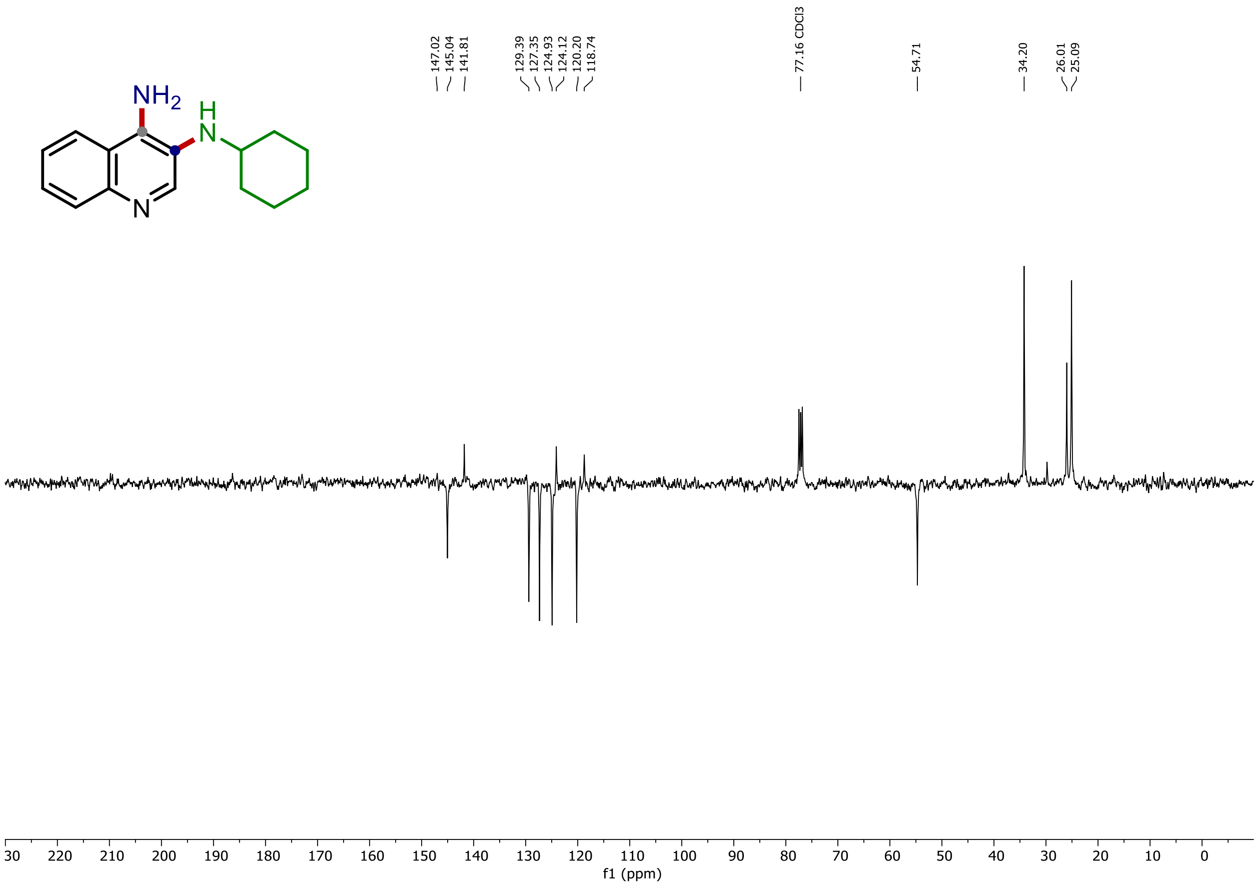

^{1}H NMR (400 MHz, $CDCl_3$) of 6 (see procedure)

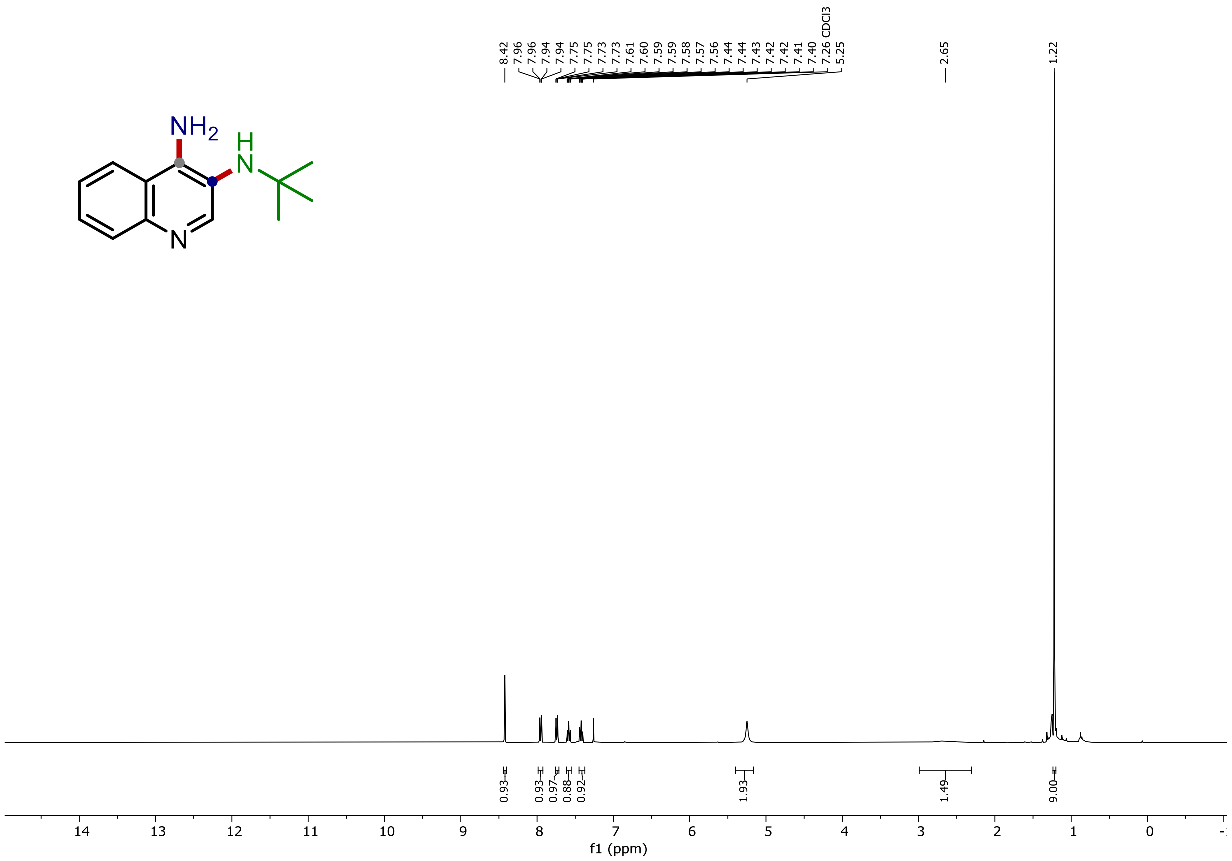

$^{13}C\{^{1}H\}$ NMR (101 MHz, $CDCl_3$) of 6

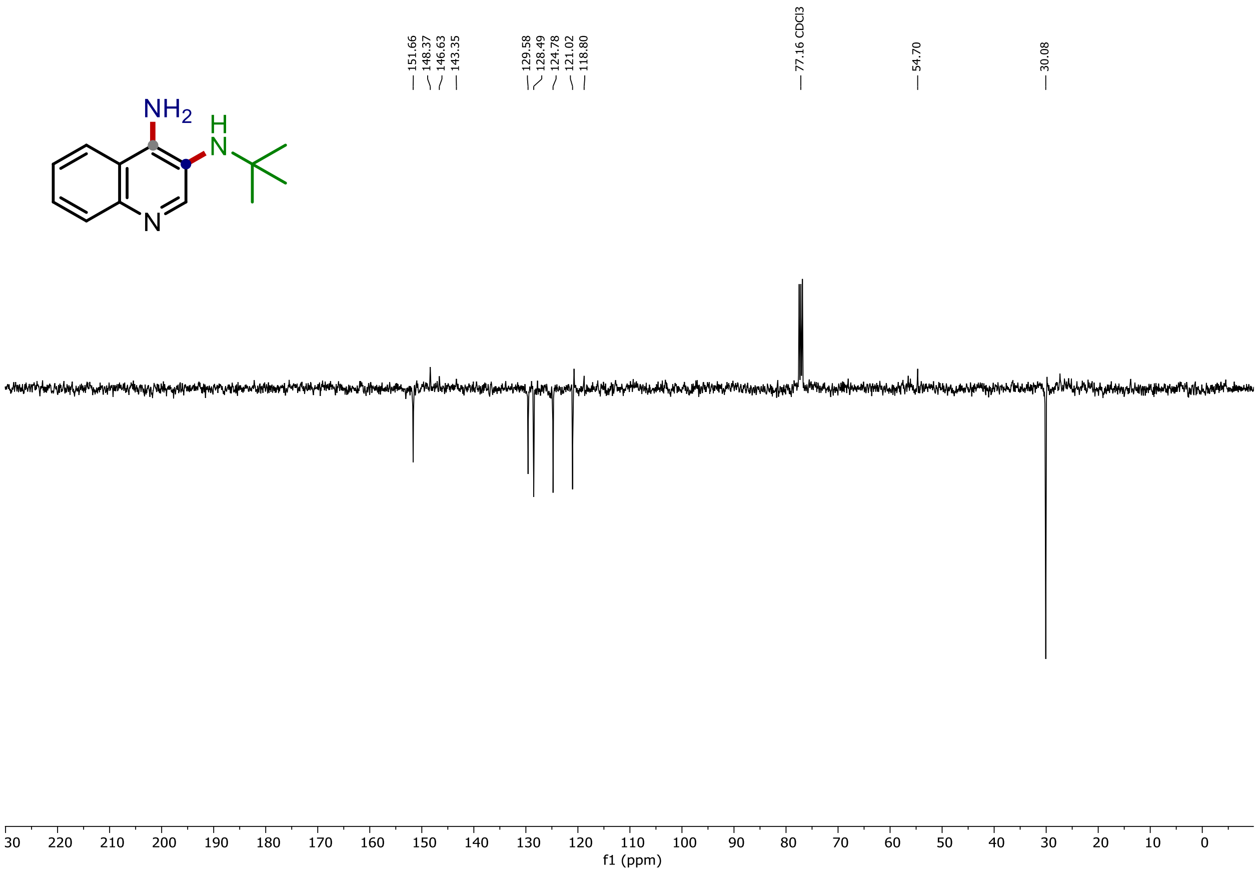

^{1}H NMR (400 MHz, $CDCl_3$) of 7 (see procedure)

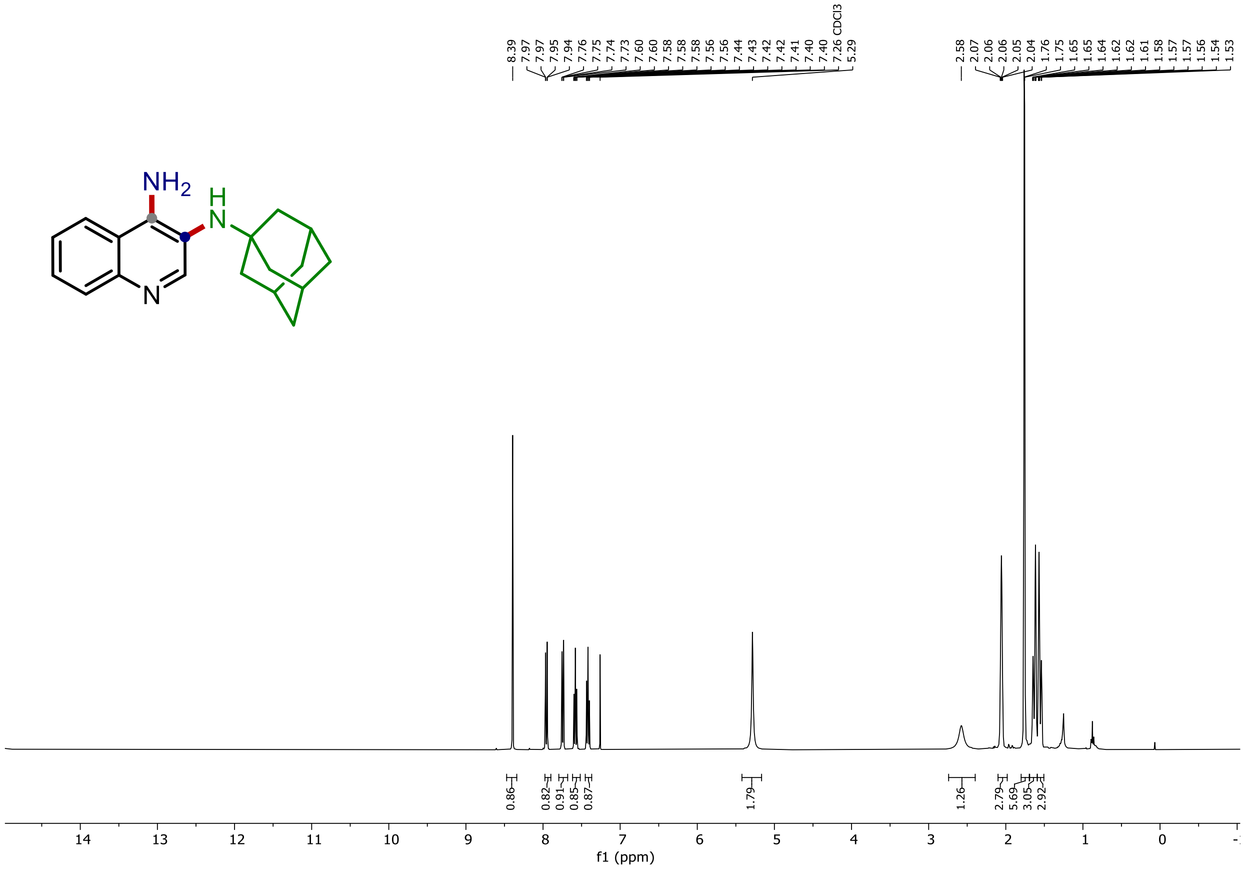

$^{13}C\{^{1}H\}$ NMR (101 MHz, $CDCl_3$) of 7

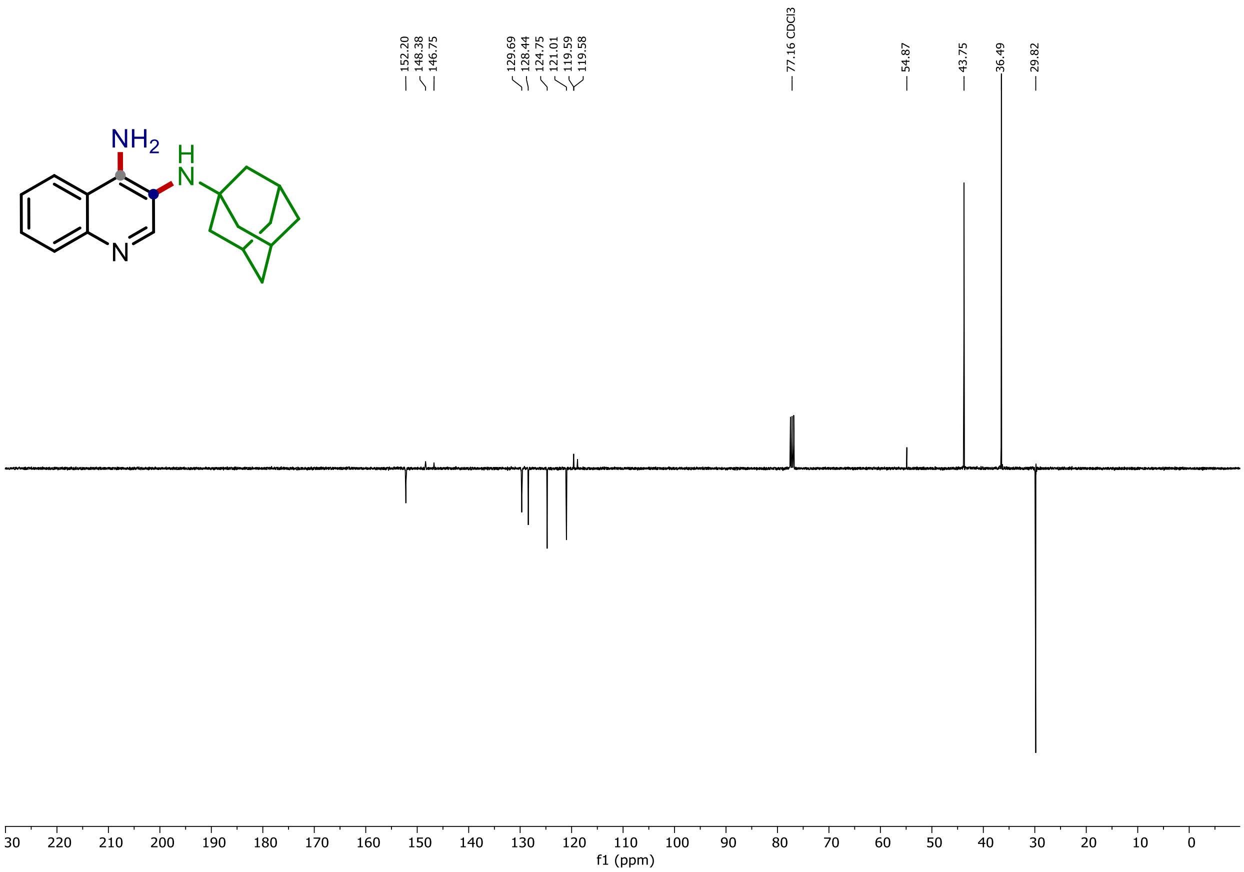

^{1}H NMR (400 MHz, $CDCl_3$) of 8 (see procedure)

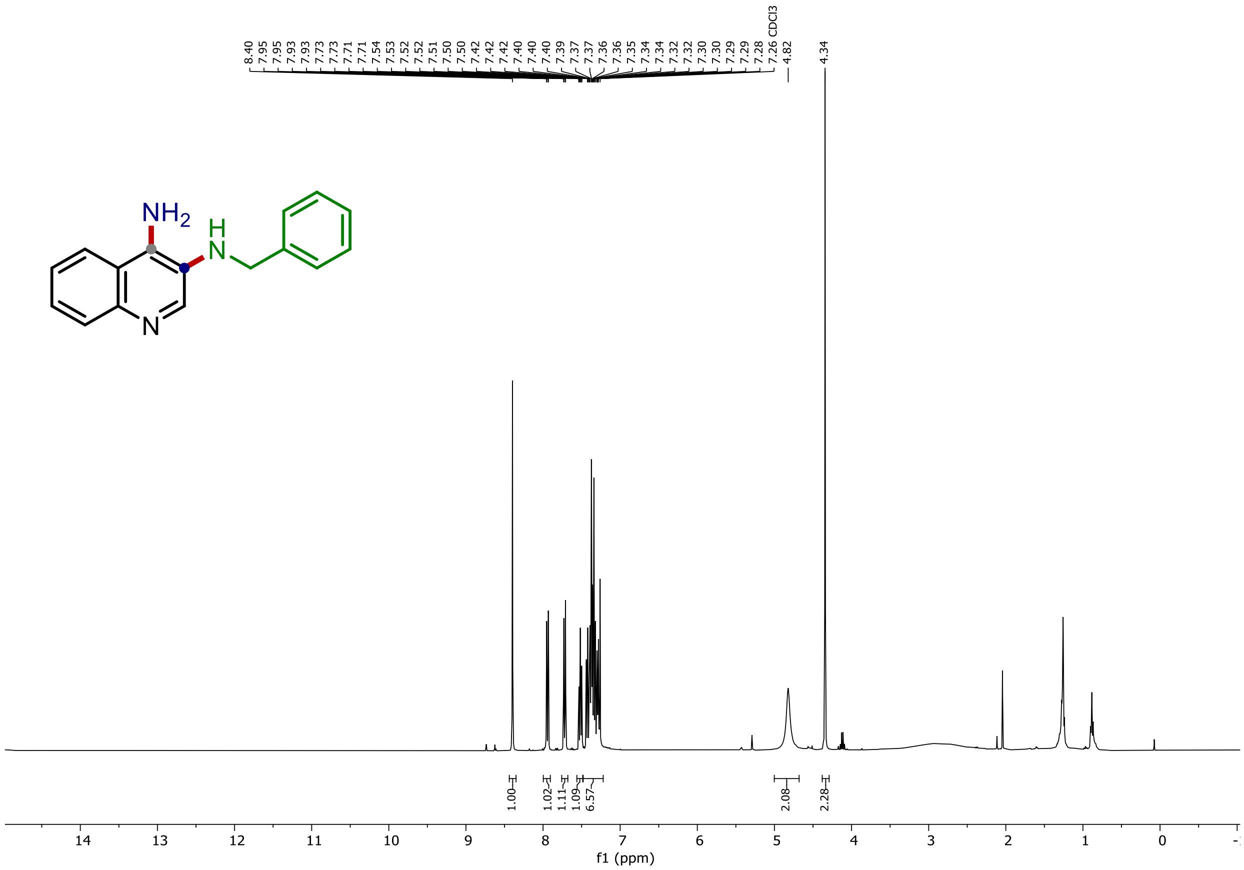

$^{13}C\{^{1}H\}$ NMR (101 MHz, $CDCl_3$) of 8

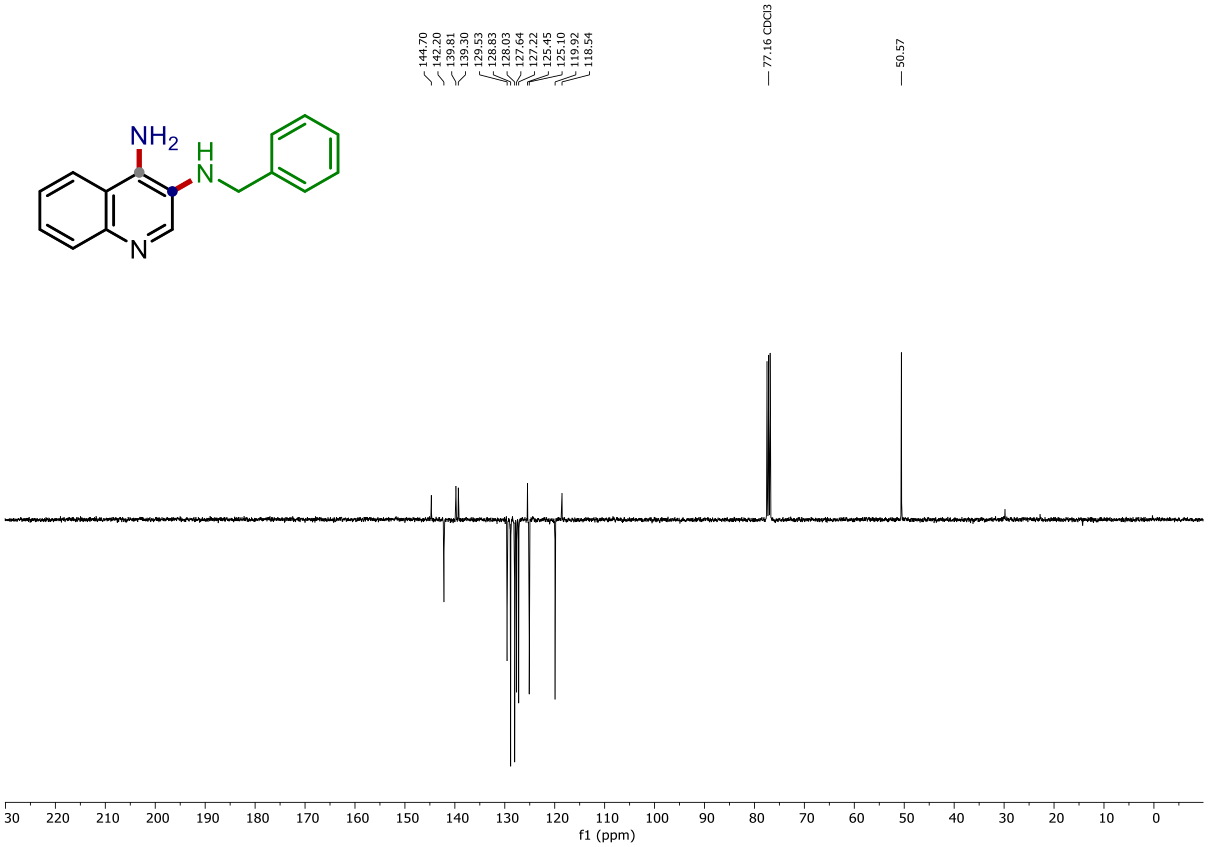

^{1}H NMR (400 MHz, $CDCl_3$) of 9 (see procedure)

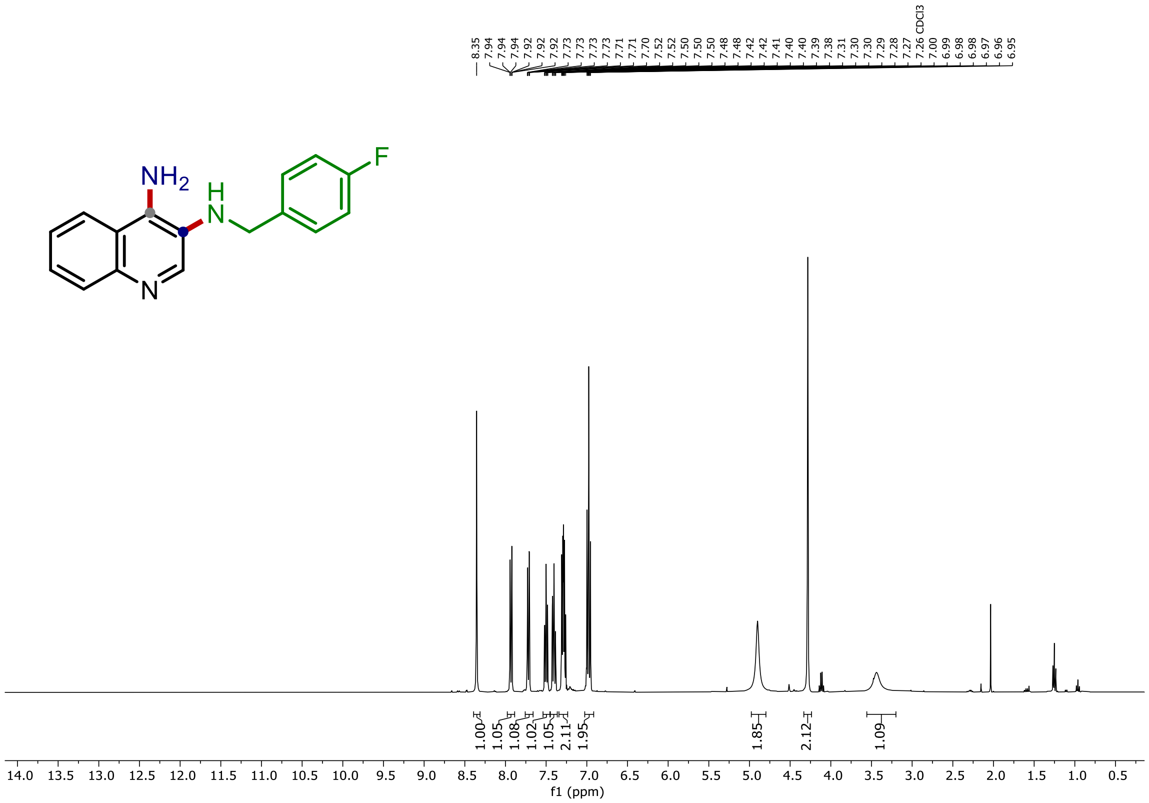

$^{13}C\{^{1}H\}$ NMR (101 MHz, $CDCl_3$) of 9

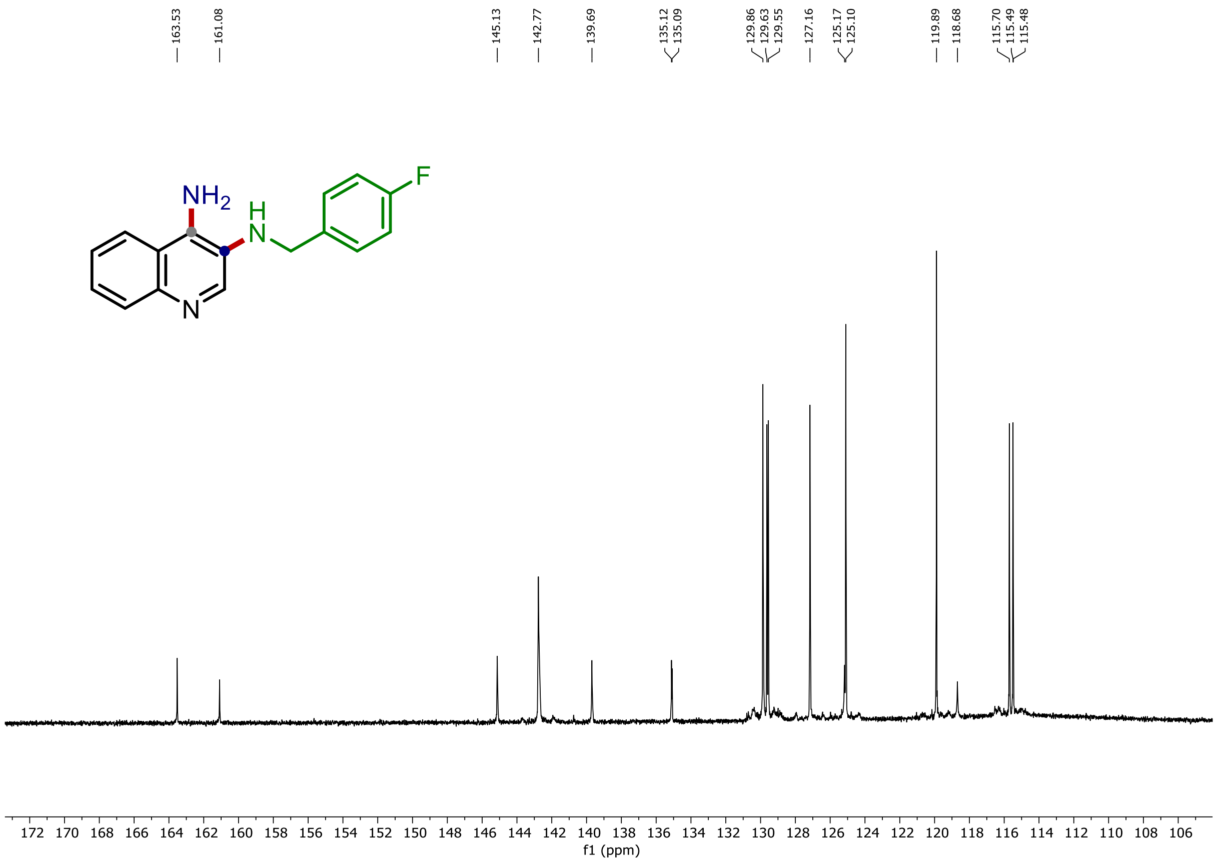

^{1}H NMR (400 MHz, $CDCl_3$) of 10 (see procedure)

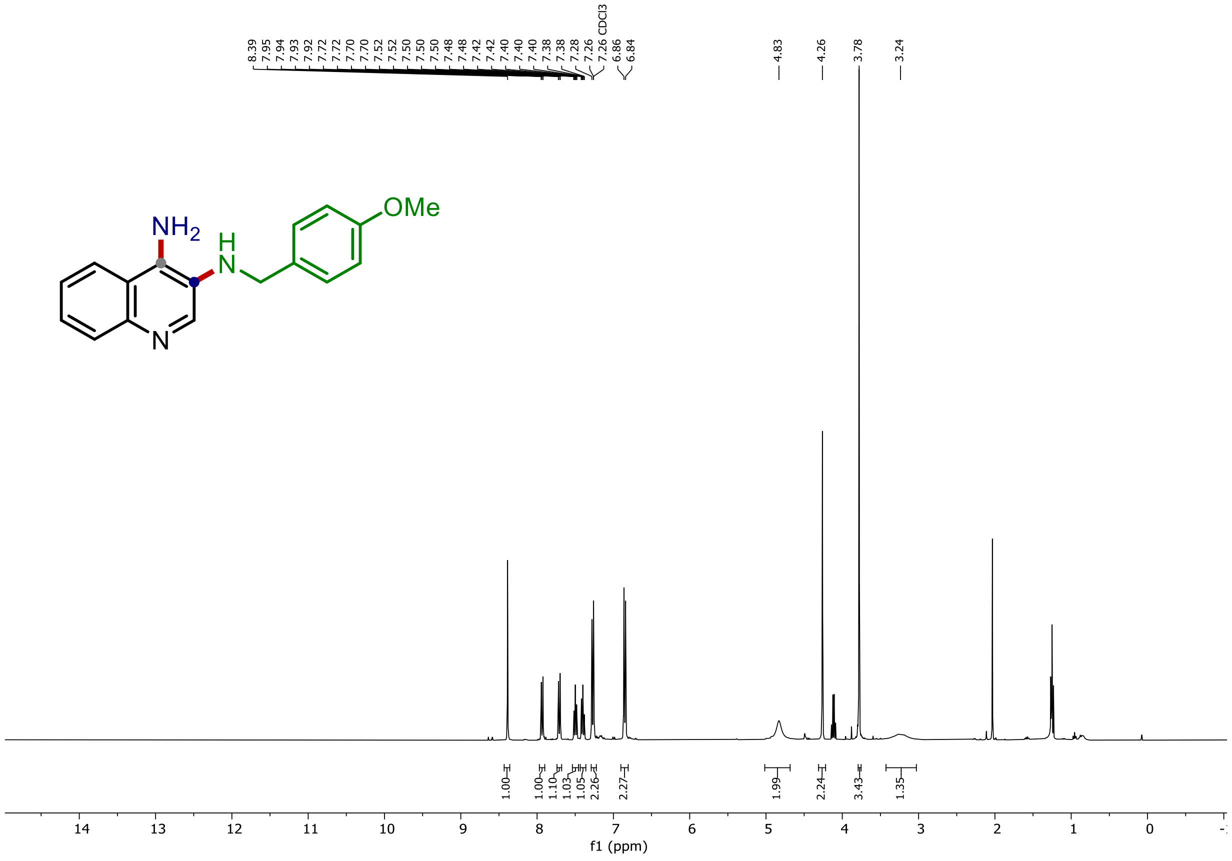

$^{13}C\{^1H\}$ NMR (101 MHz, $CDCl_3$) of 10

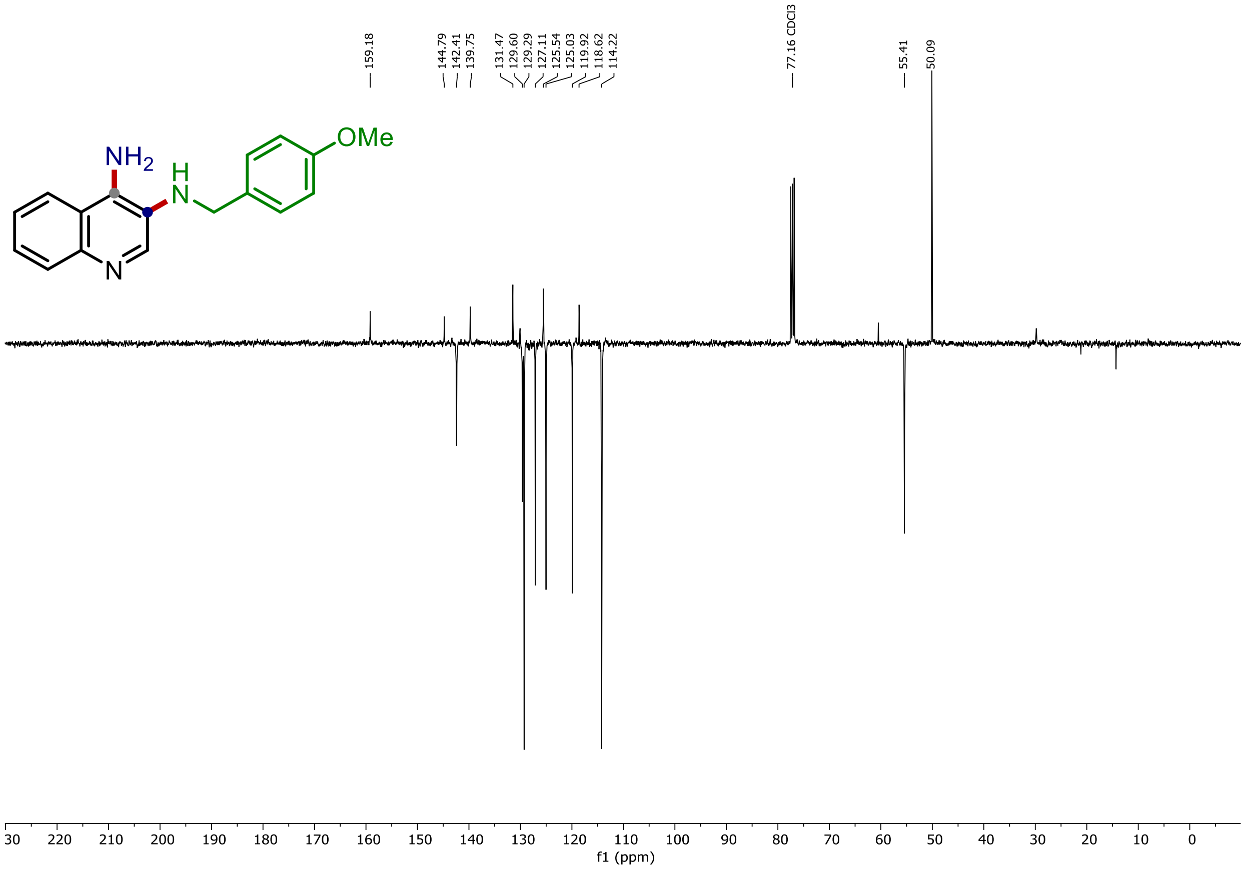

^1H NMR (400 MHz, $CDCl_3$) of 11 (see procedure)

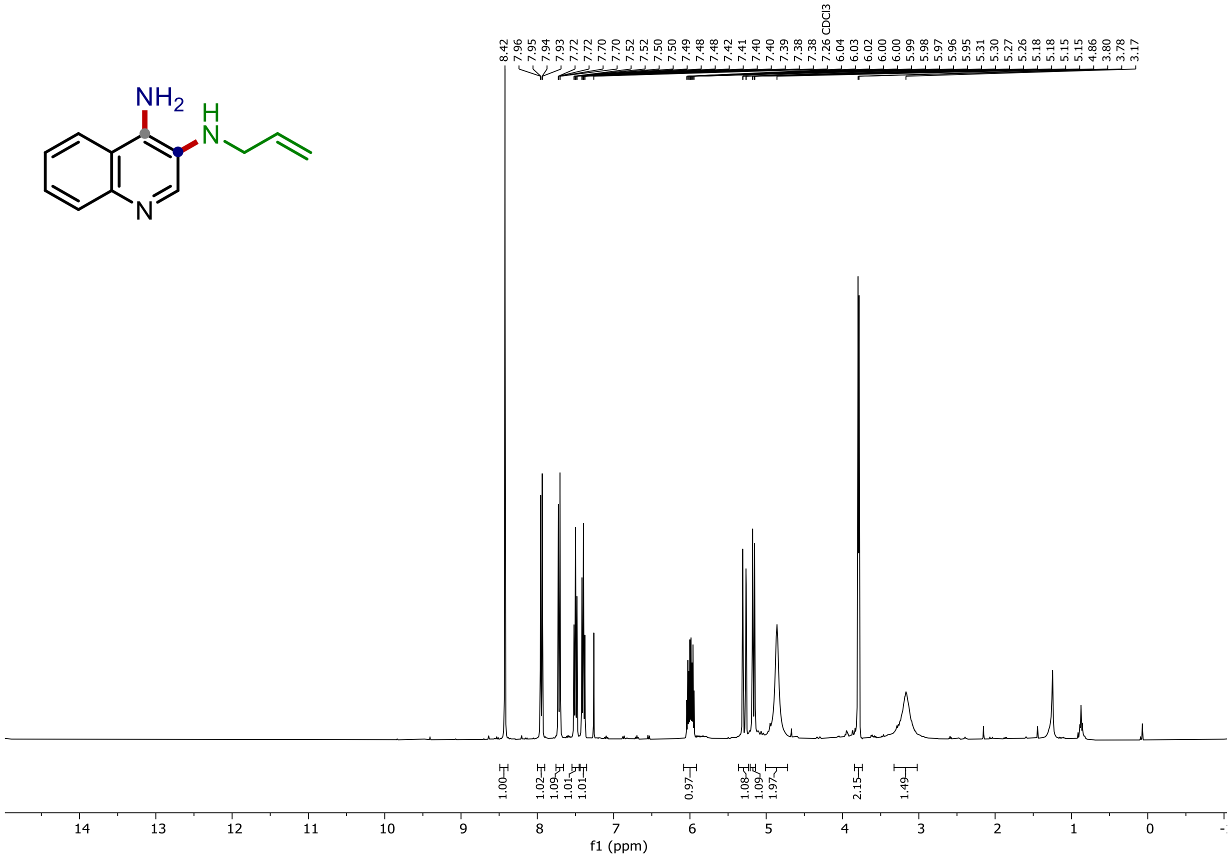

$^{13}C\{^1H\}$ NMR (101 MHz, $CDCl_3$) of 11

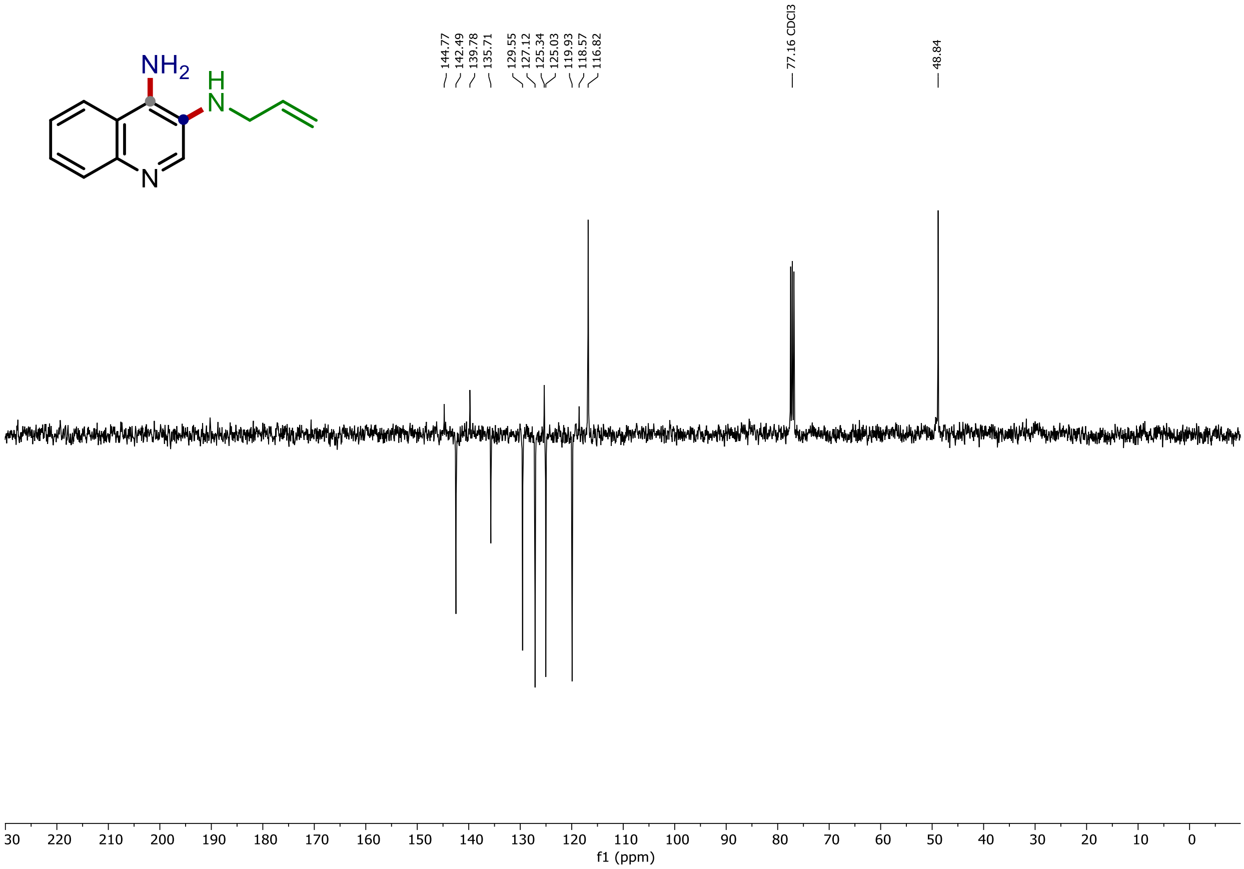

^1H NMR (400 MHz, $CDCl_3$) of 12 (see procedure)

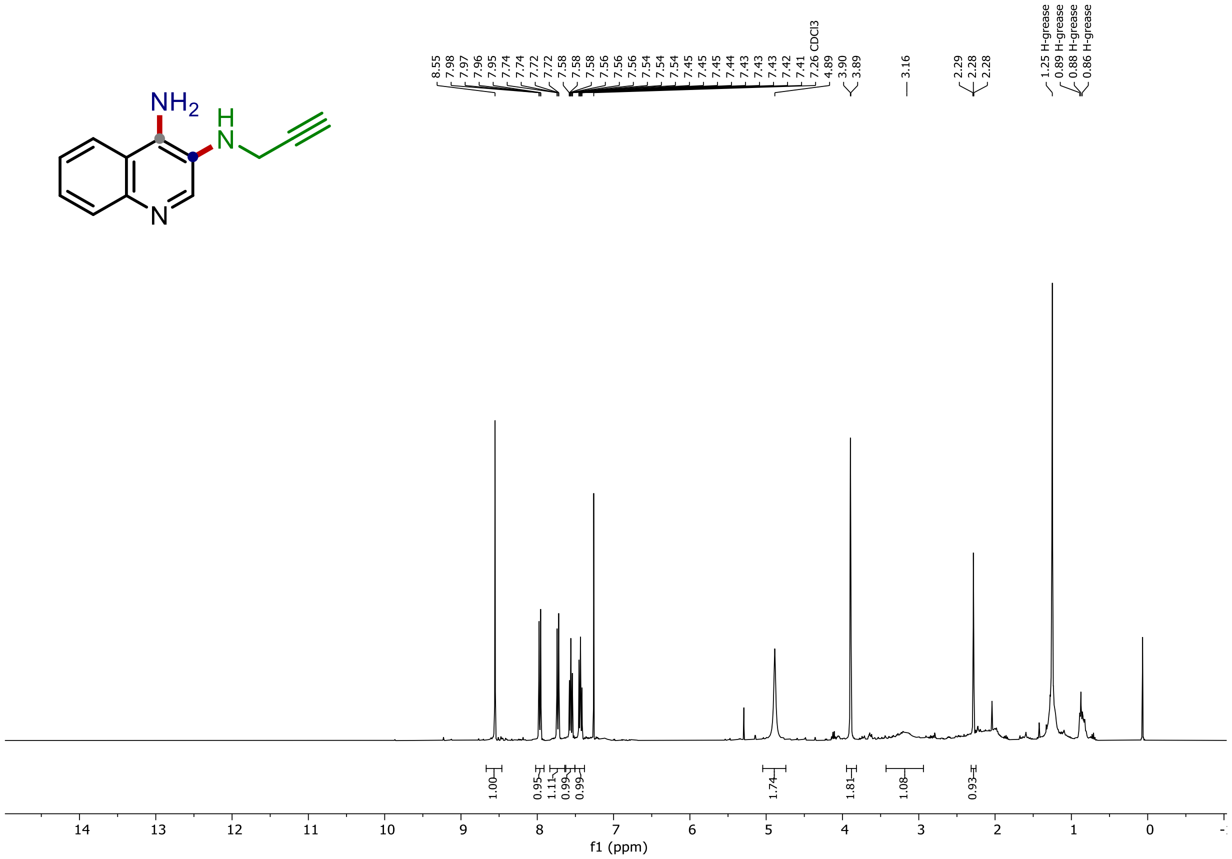

$^{13}C\{^1H\}$ NMR (101 MHz, $CDCl_3$) of 12

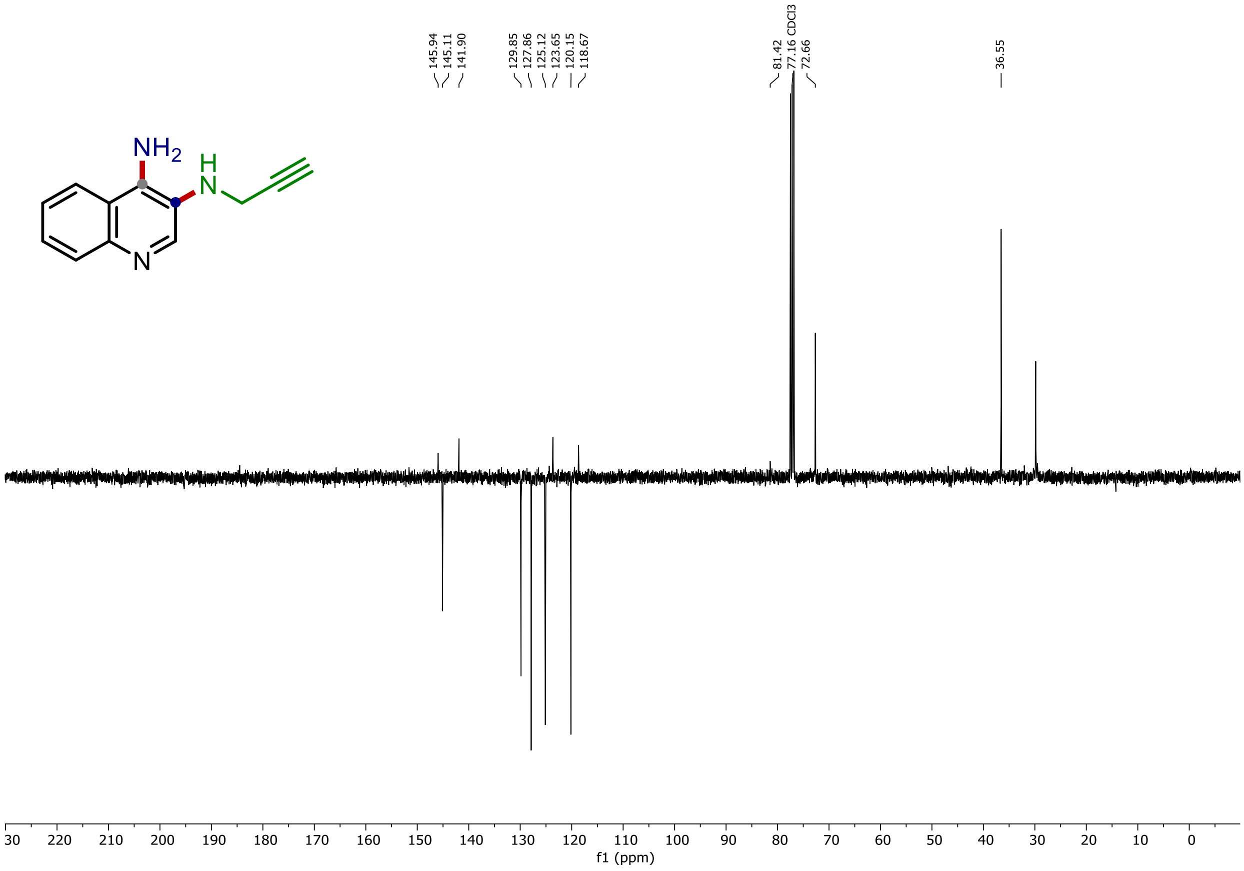

^1H NMR (400 MHz, $CDCl_3$) of 13 (see procedure)

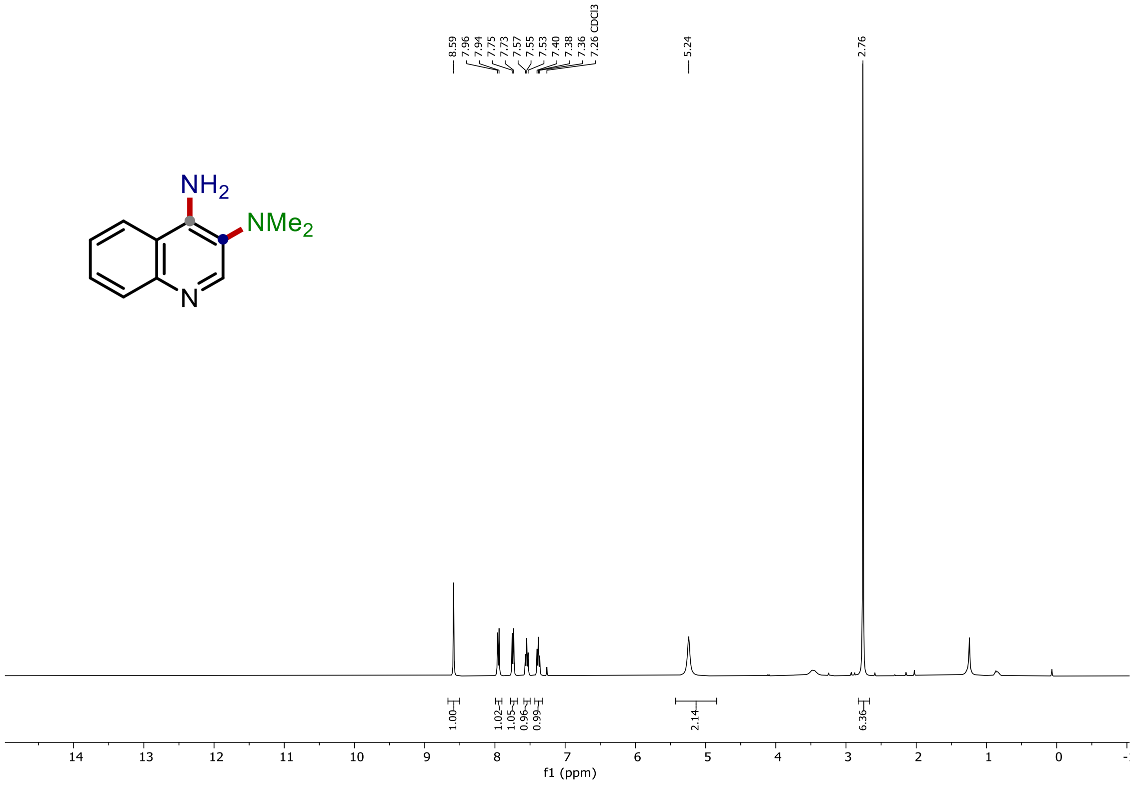

$^{13}C\{^{1}H\}$ NMR (101 MHz, $CDCl_3$) of 13

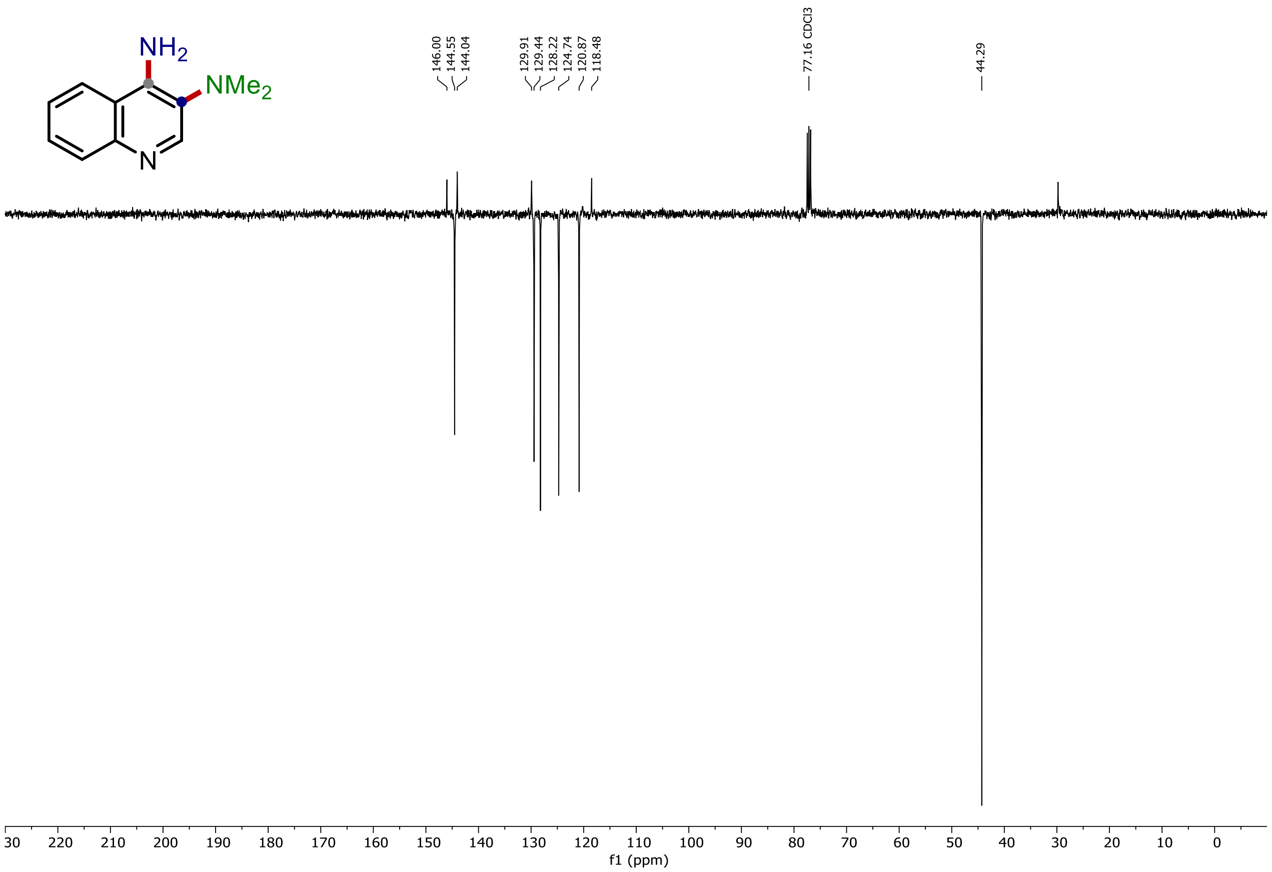

^{1}H NMR (400 MHz, $CDCl_3$) of 14 (see procedure)

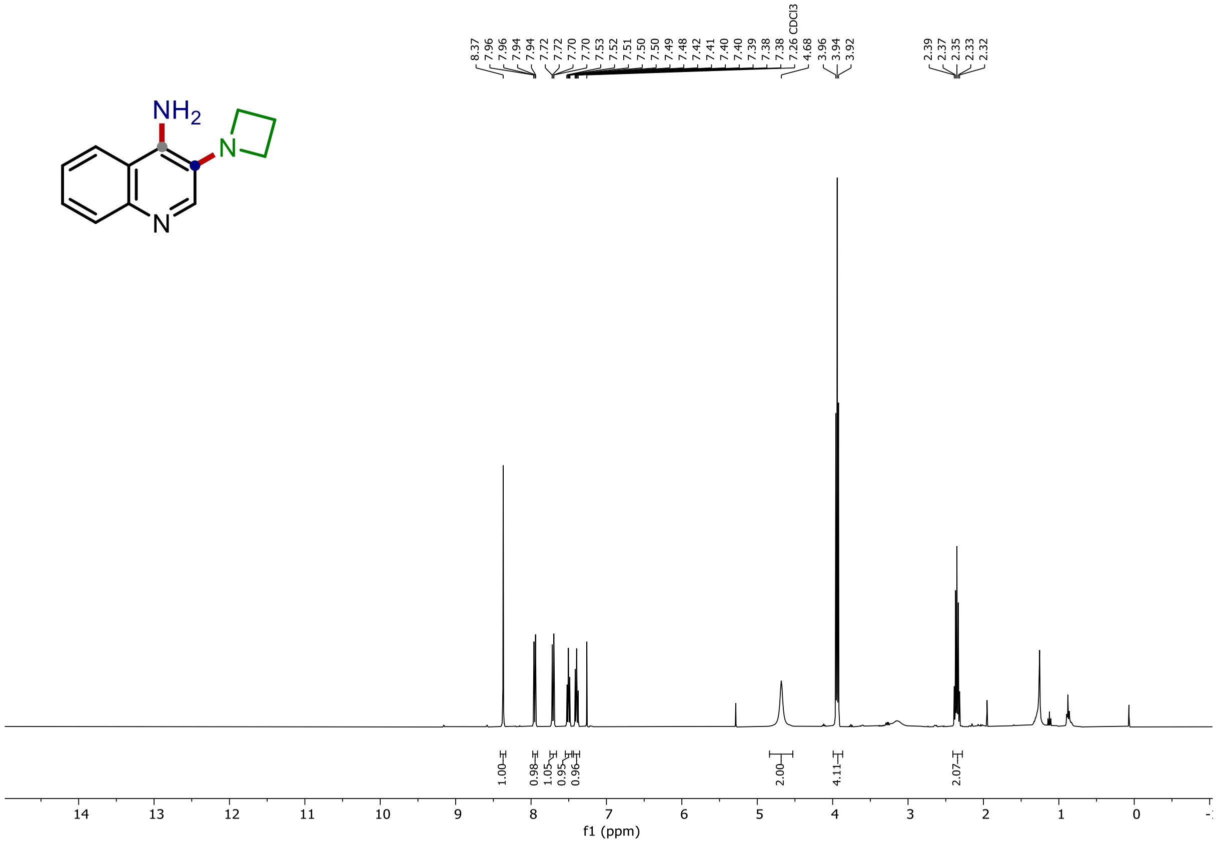

$^{13}C\{^{1}H\}$ NMR (101 MHz, $CDCl_3$) of 14

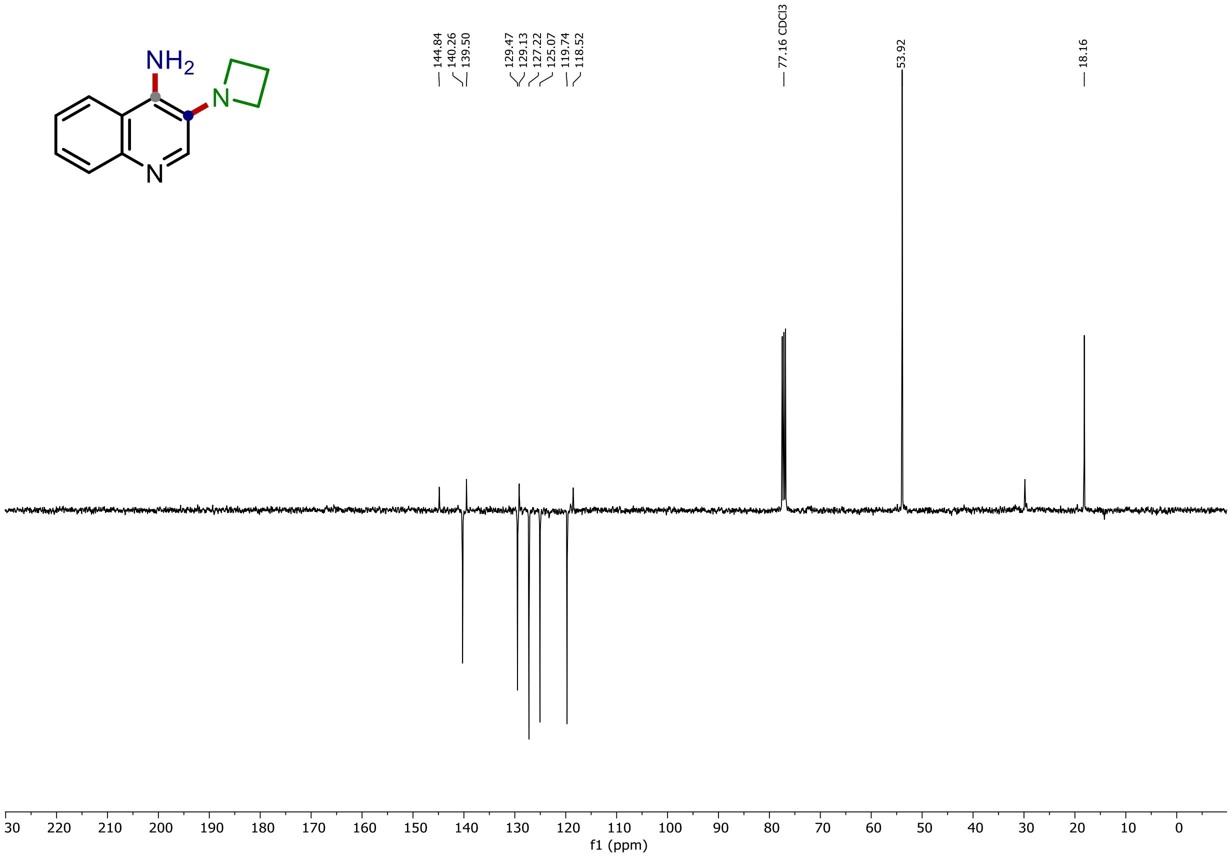

^{1}H NMR (400 MHz, $CDCl_3$) of 15 (see procedure)

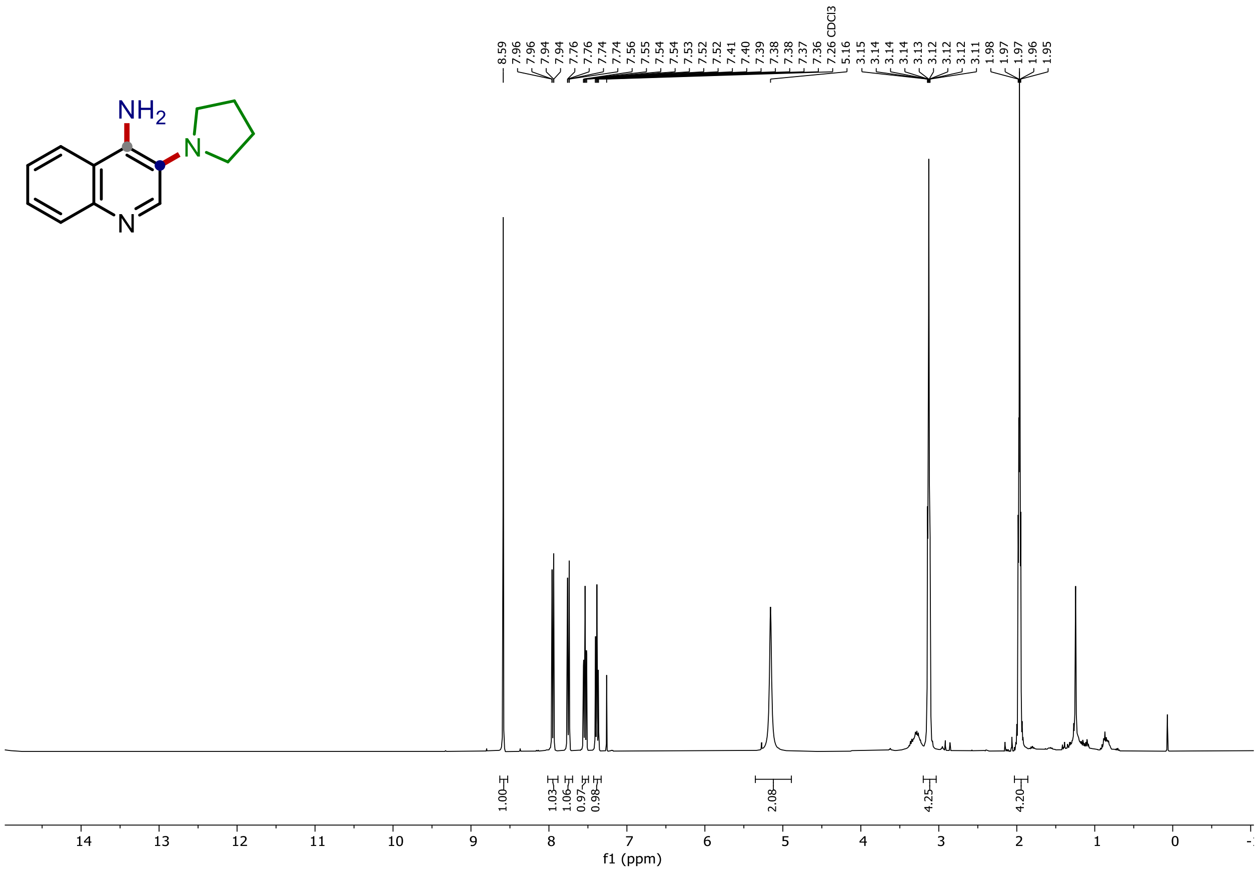

$^{13}C\{^{1}H\}$ NMR (101 MHz, $CDCl_3$) of 15

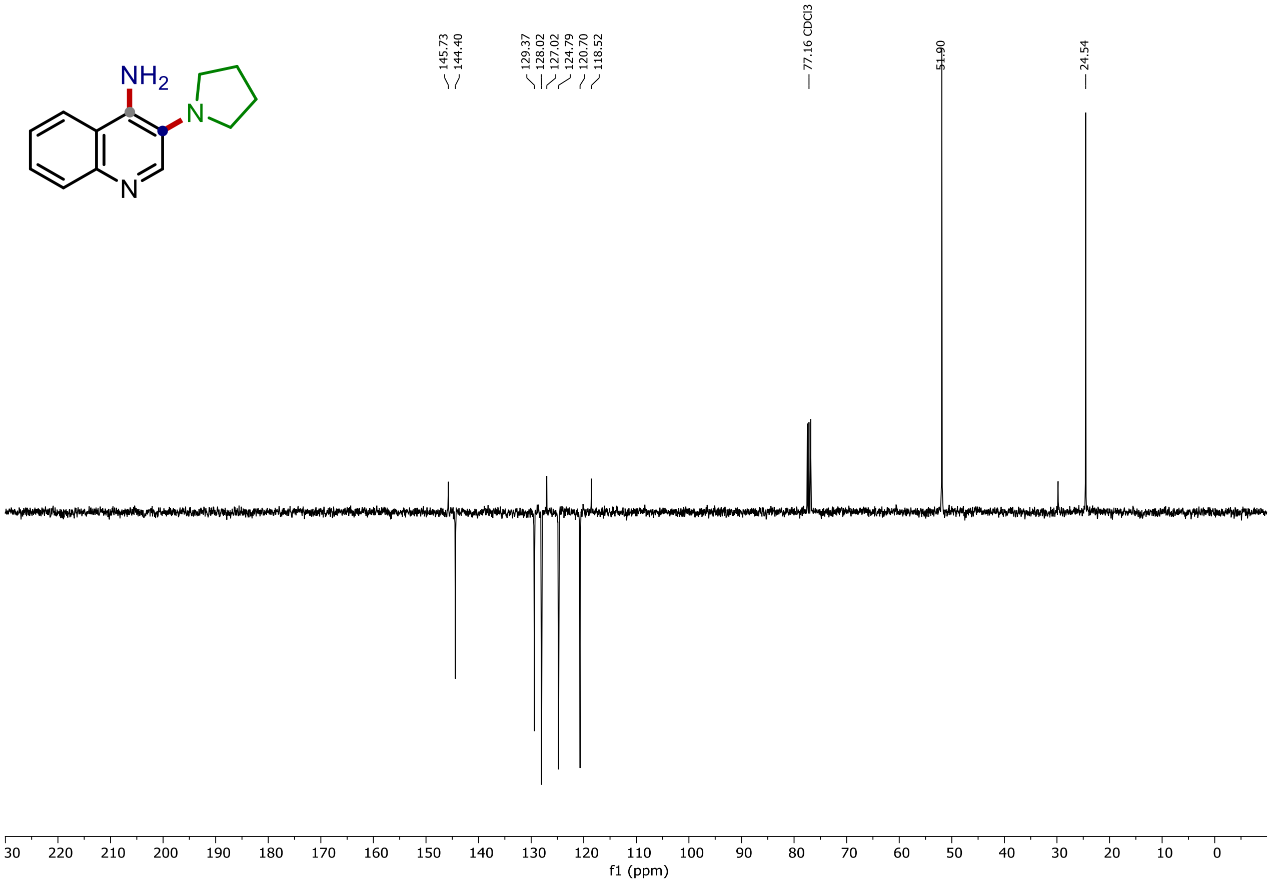

^{1}H NMR (400 MHz, $CDCl_3$) of 16 (see procedure)

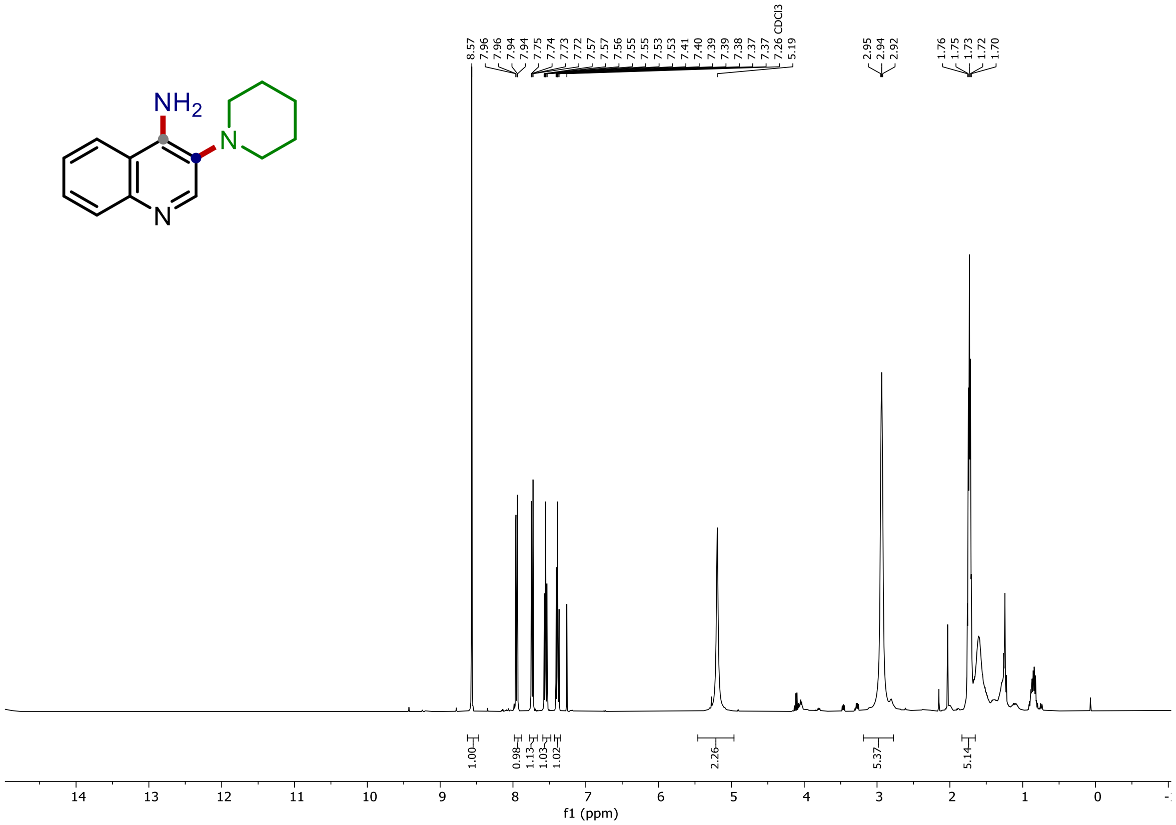

$^{13}C\{^1H\}$ NMR (101 MHz, $CDCl_3$) of 16

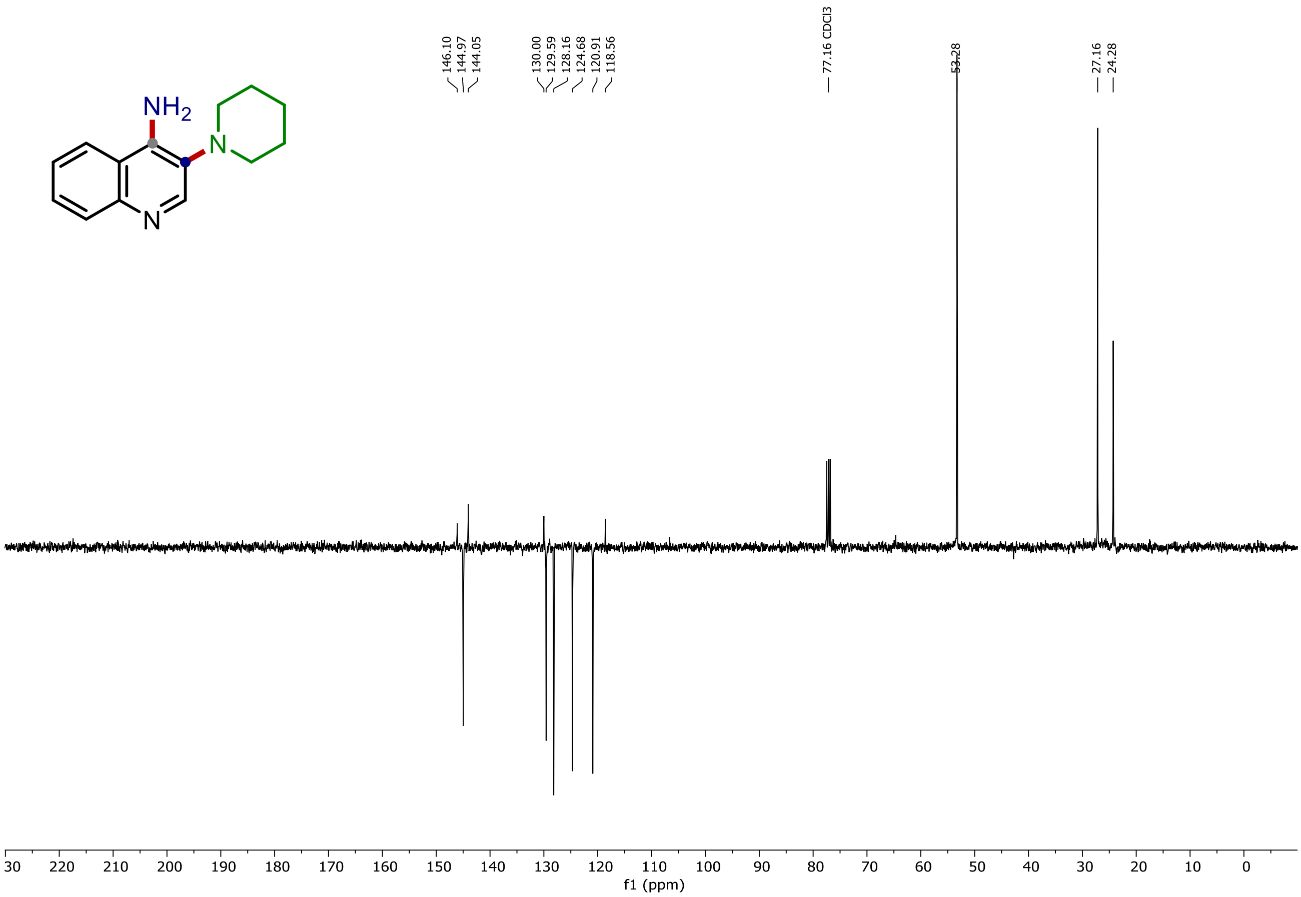

^1H NMR (400 MHz, $CDCl_3$) of 17 (see procedure)

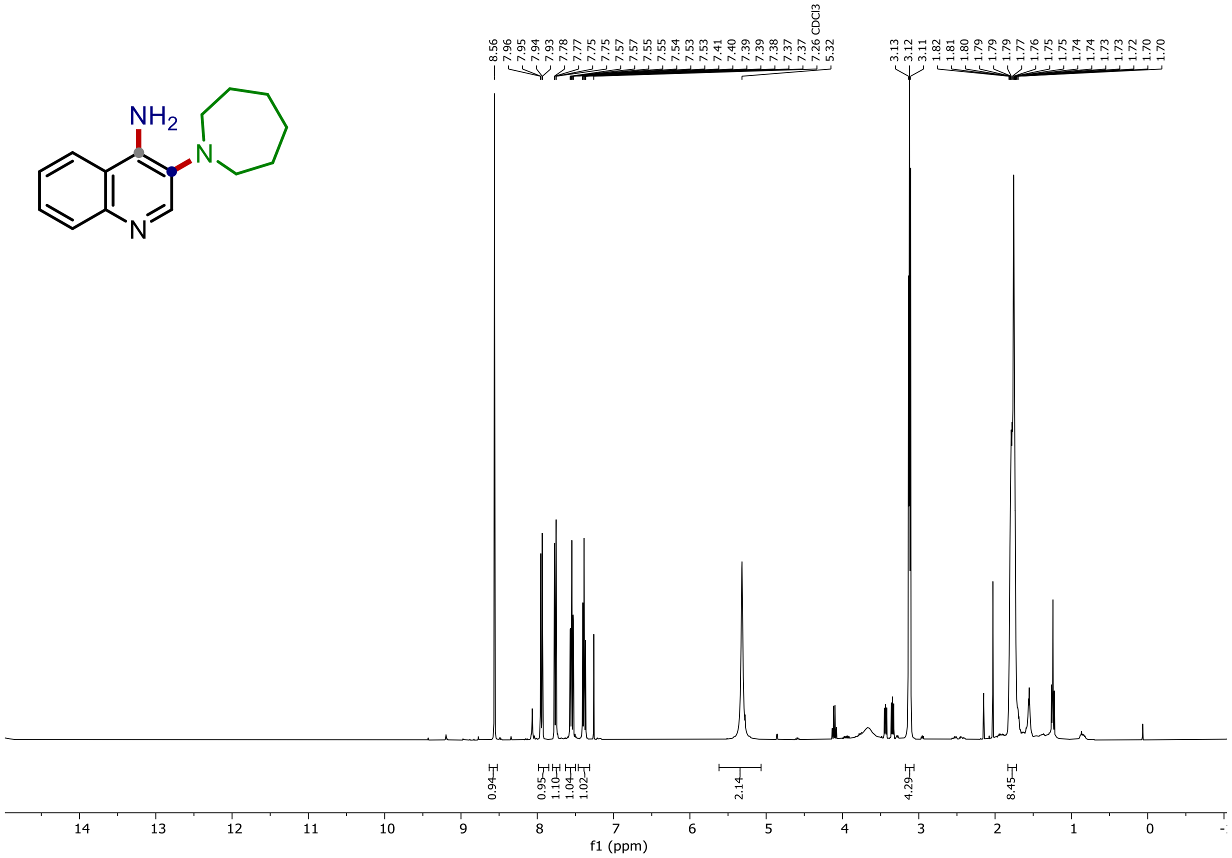

$^{13}C\{^1H\}$ NMR (101 MHz, $CDCl_3$) of 17

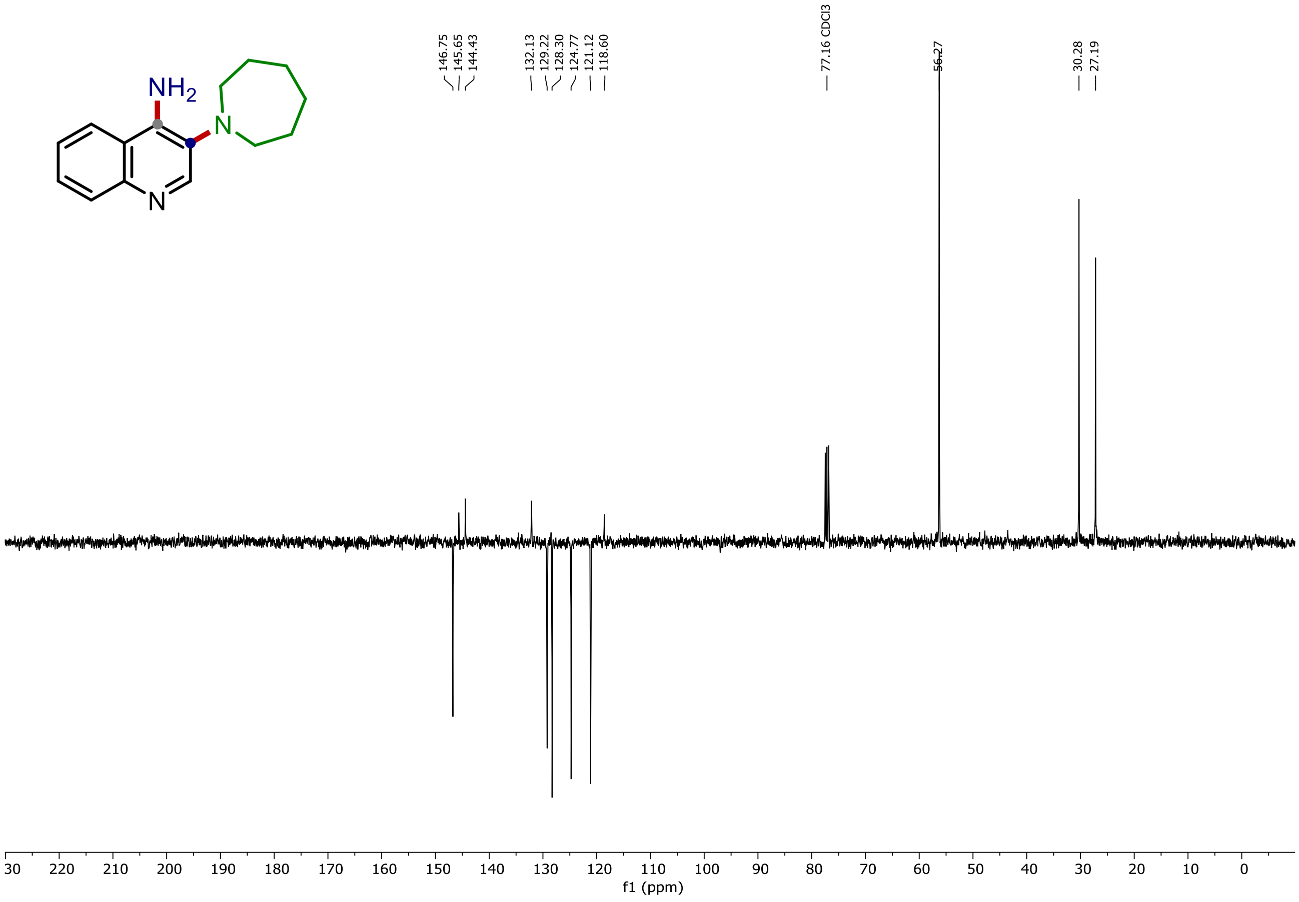

^1H NMR (400 MHz, $CDCl_3$) of 18 (see procedure)

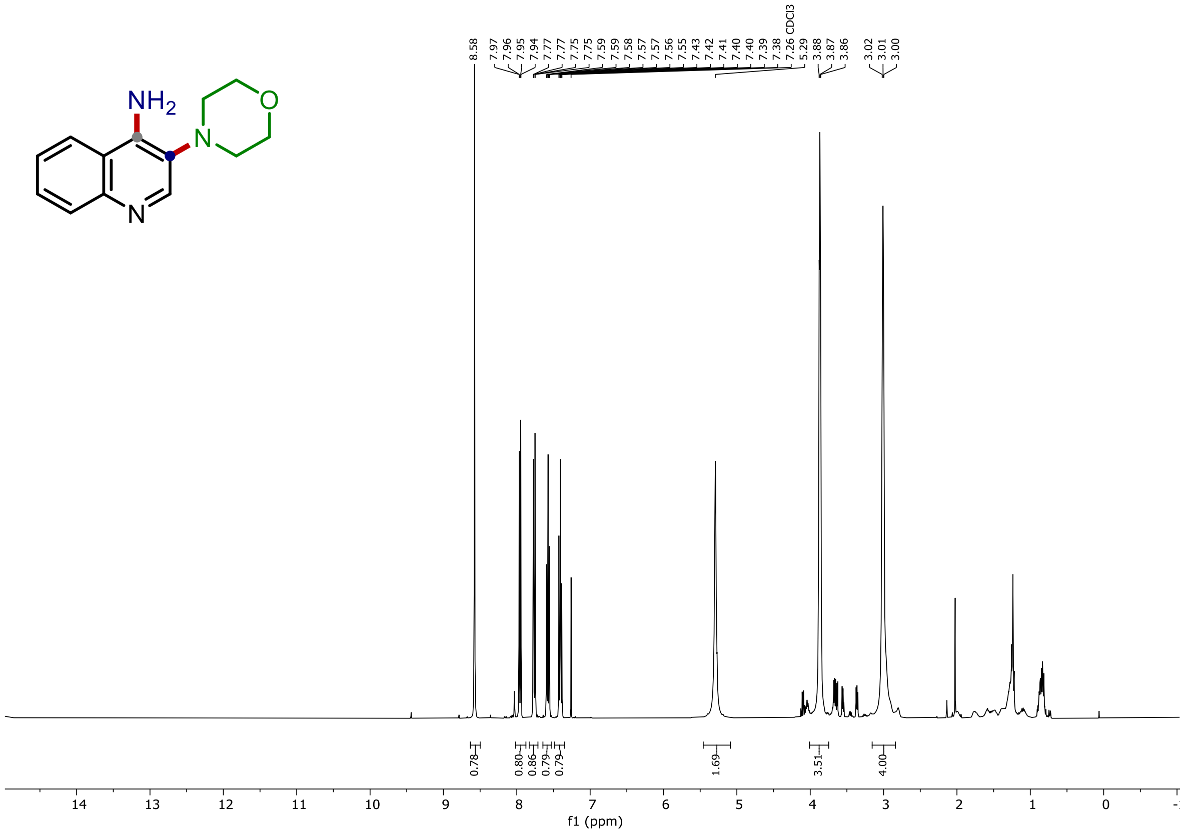

$^{13}C\{^{1}H\}$ NMR (101 MHz, $CDCl_3$) of 18

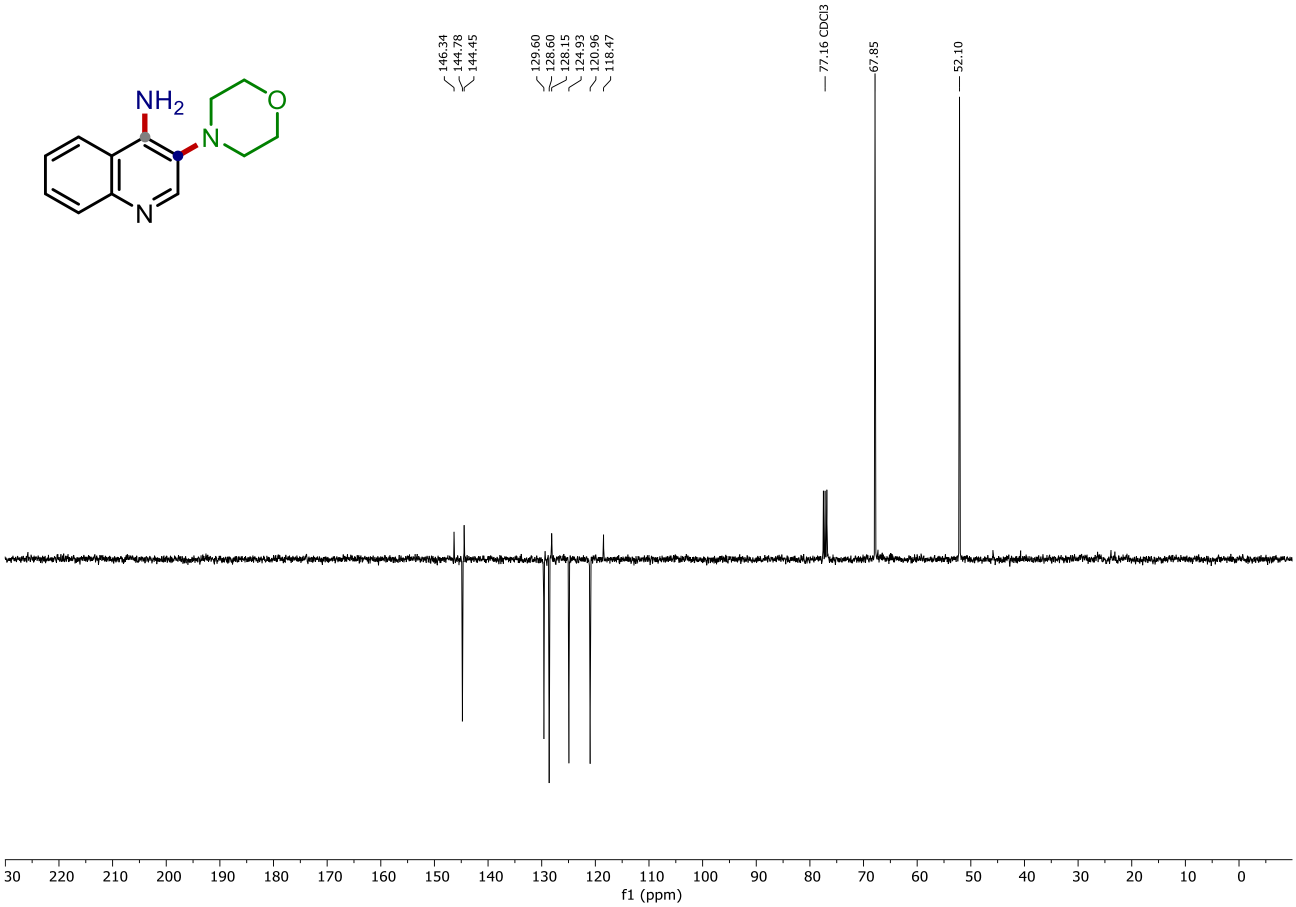

^{1}H NMR (400 MHz, $CDCl_3$) of 19 (see procedure)

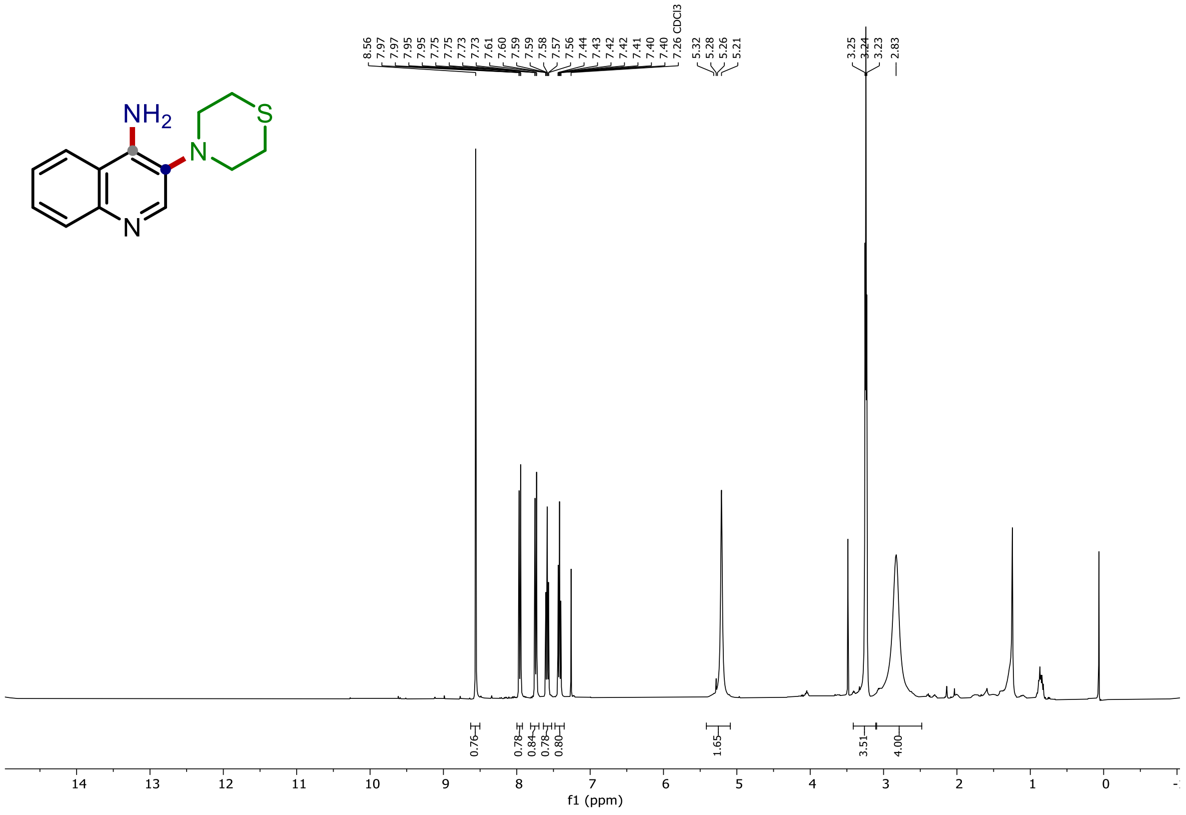

$^{13}C\{^{1}H\}$ NMR (101 MHz, $CDCl_3$) of 19

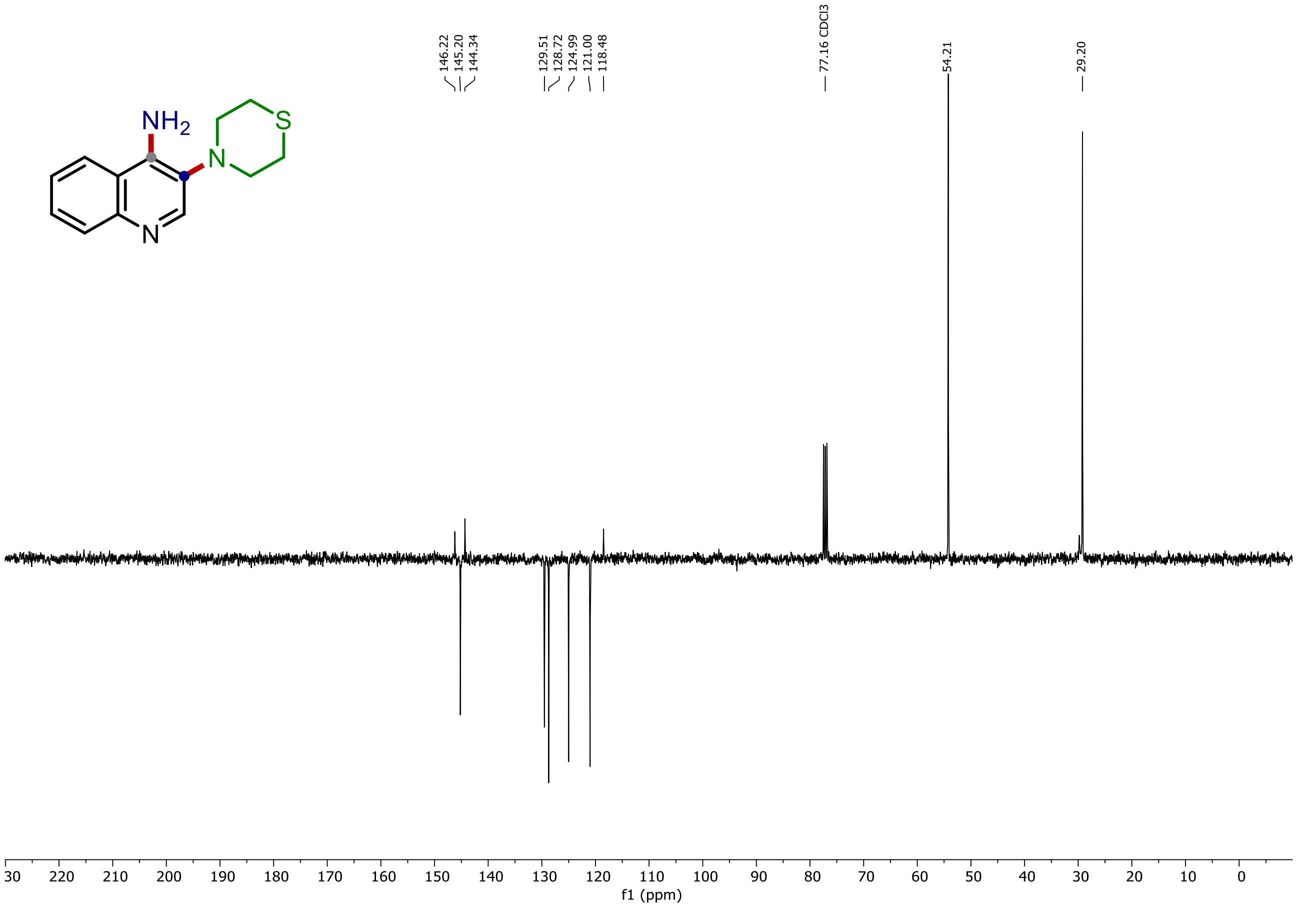

^{1}H NMR (400 MHz, $CDCl_3$) of 20 (see procedure)

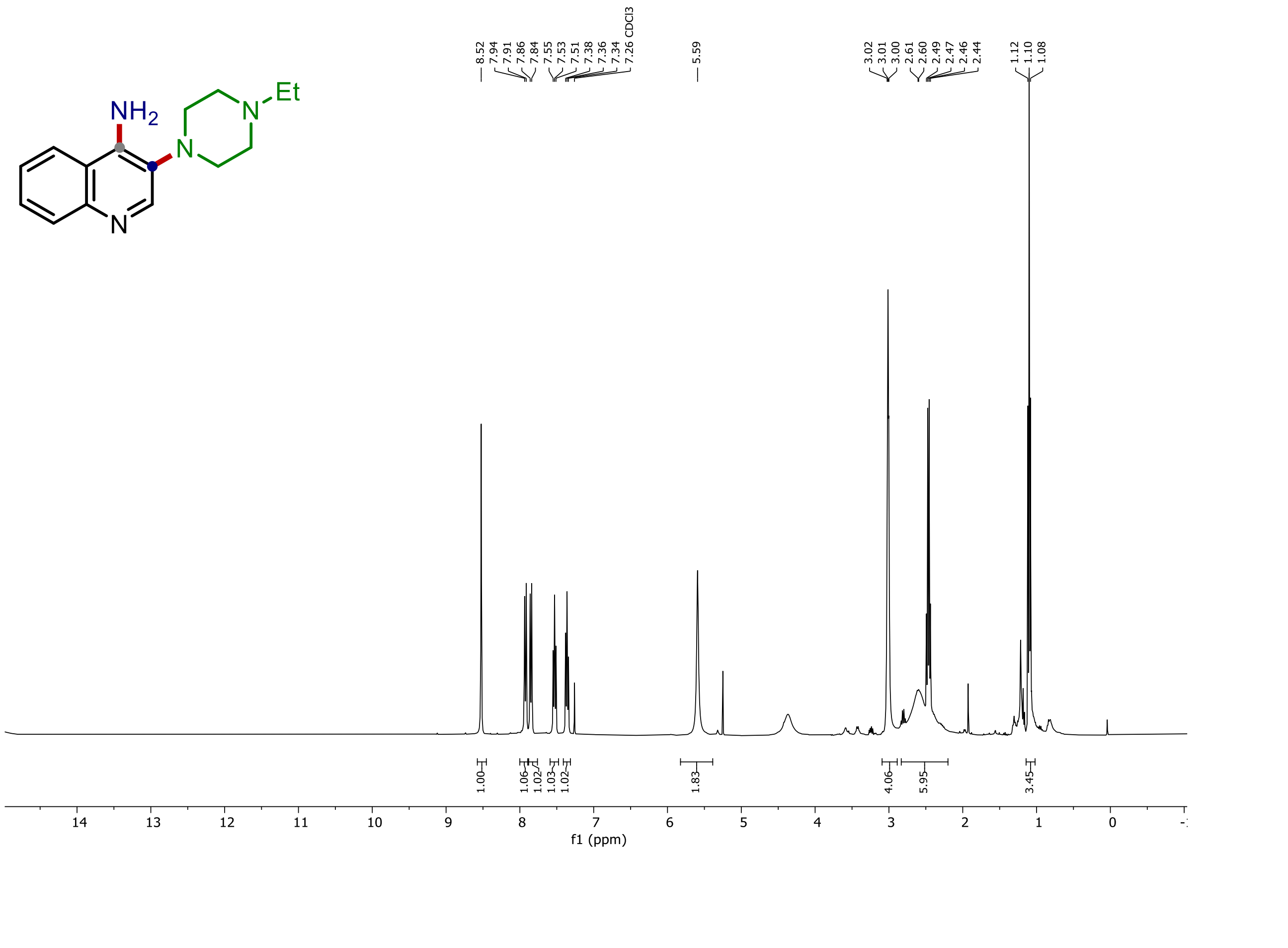

$^{13}C\{^1H\}$ NMR (101 MHz, $CDCl_3$) of 20

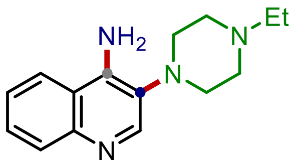

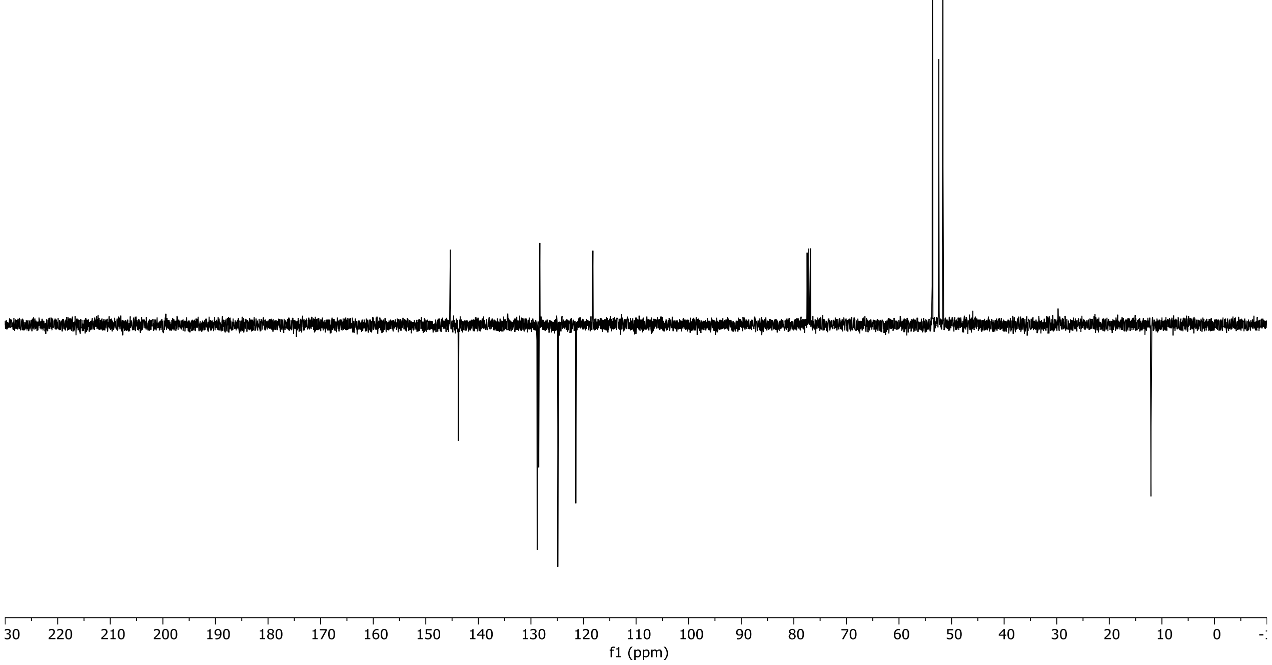

^{1}H NMR (400 MHz, $CDCl_3$) of 21 (see procedure)

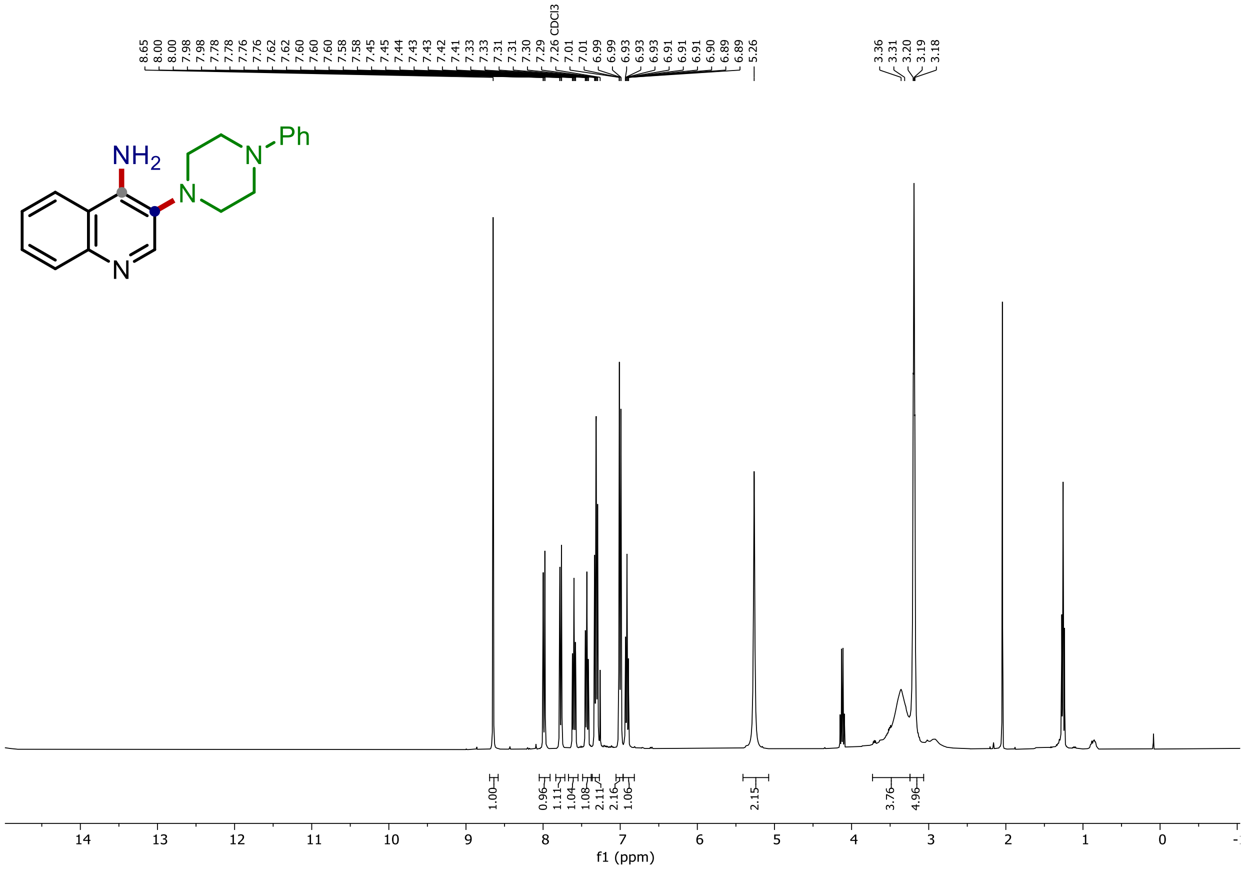

^{13}C{^{1}H} NMR (101 MHz, $CDCl_3$) of 21

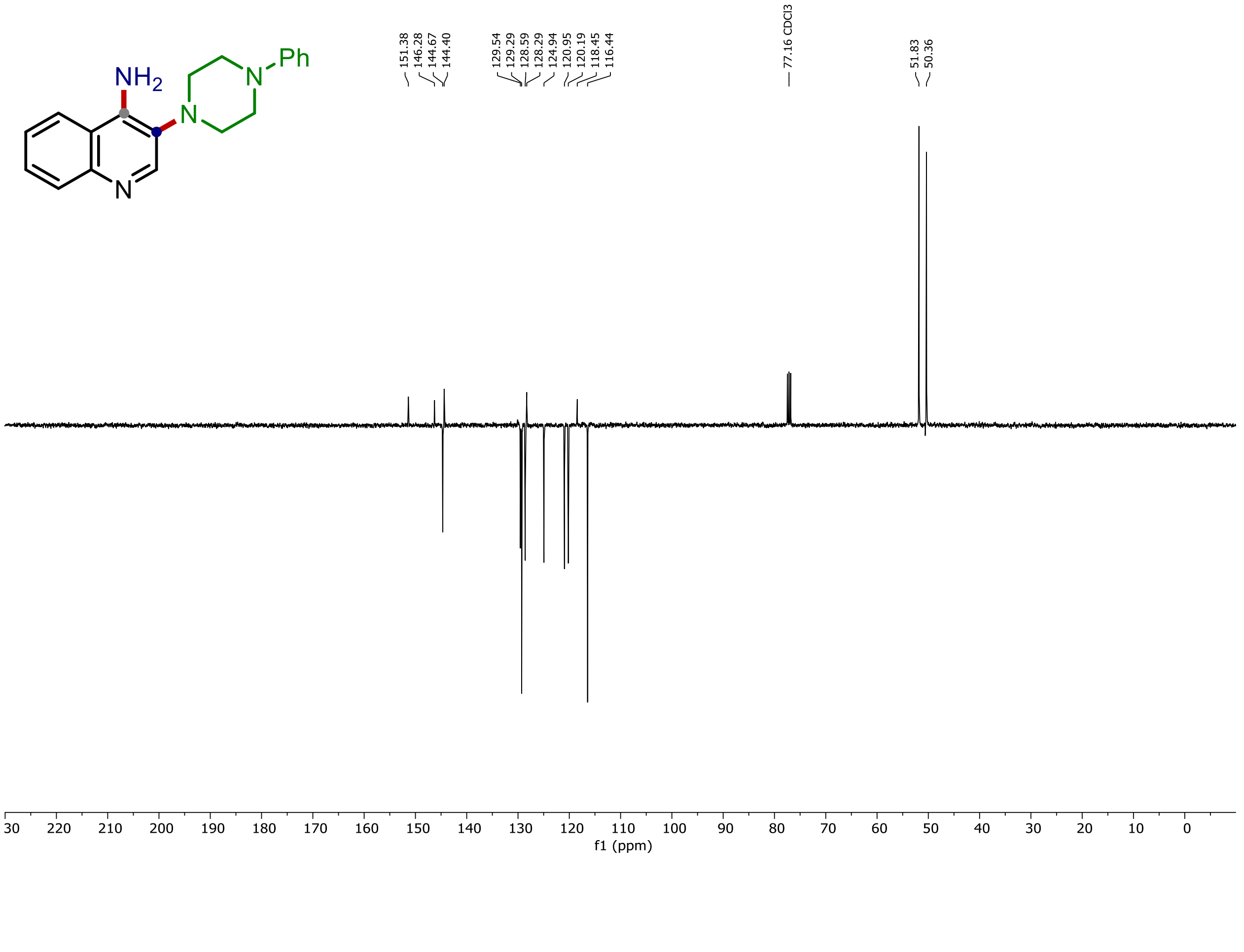

^{1}H NMR (400 MHz, $CDCl_3$) of 22 (see procedure)

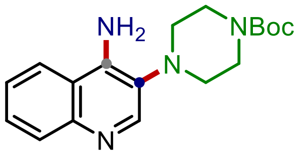

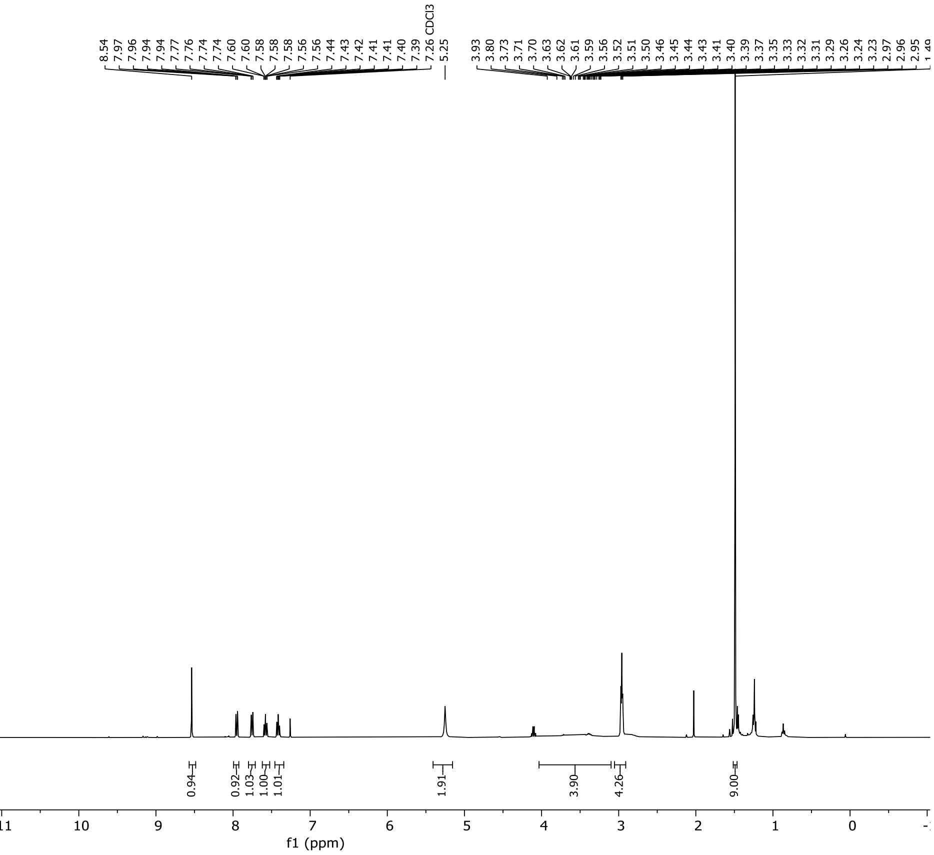

$^{13}C\{^1H\}$ NMR (400 MHz, $CDCl_3$) of 22

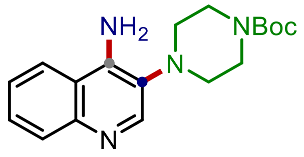

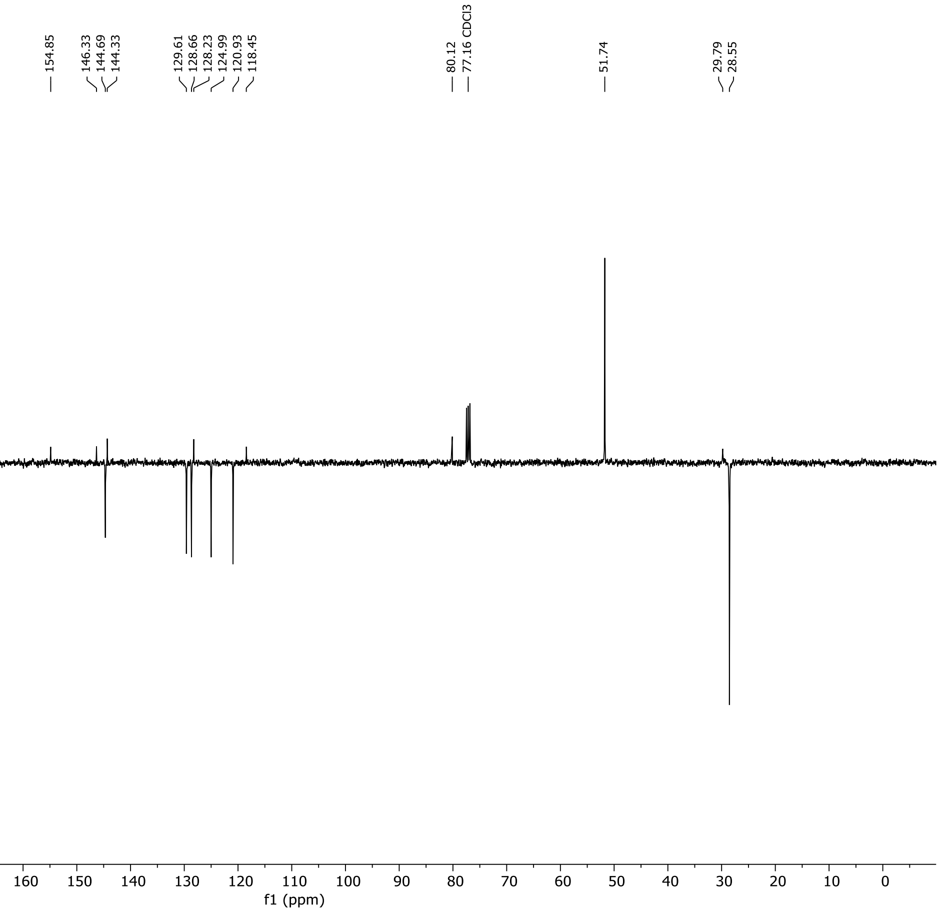

^{1}H NMR (400 MHz, $CDCl_3$) of 23 (see procedure)

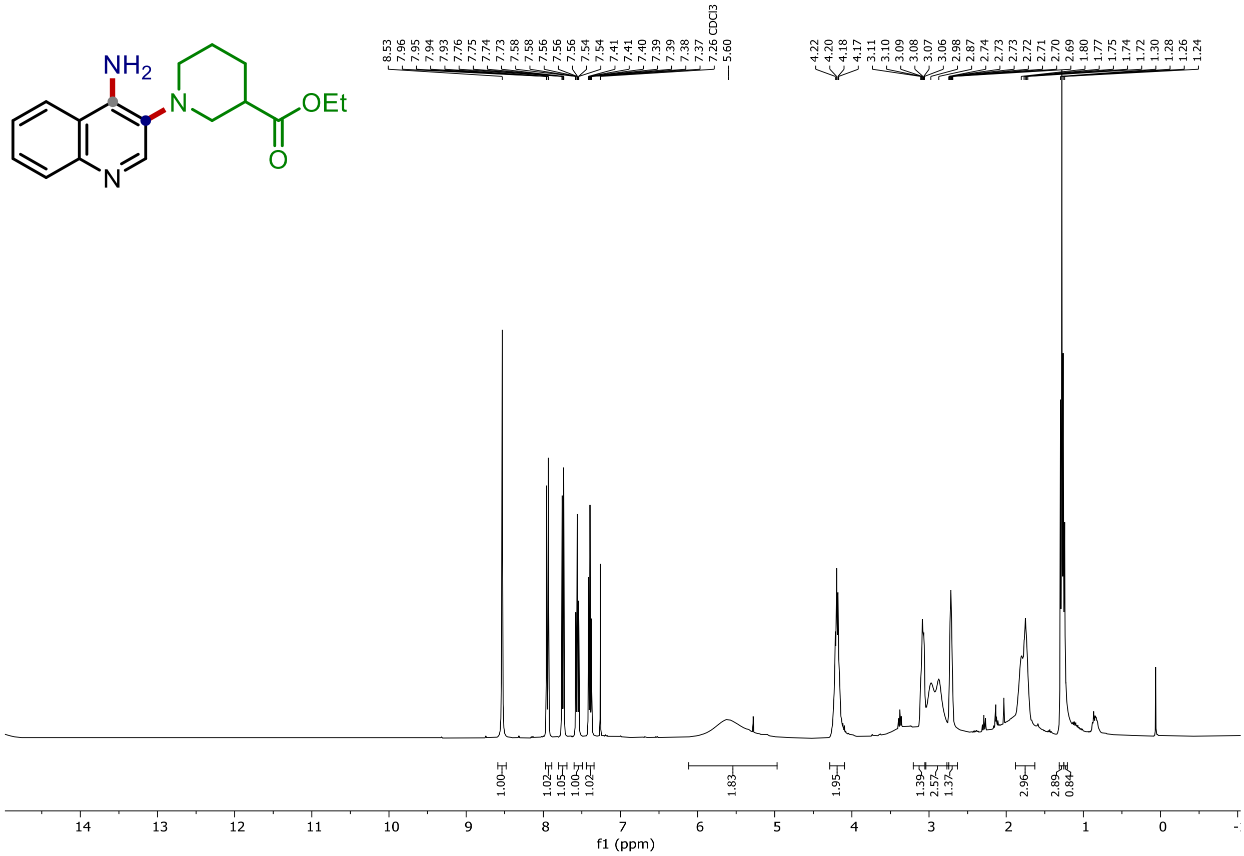

$^{13}C\{^1H\}$ NMR (101 MHz, $CDCl_3$) of 23

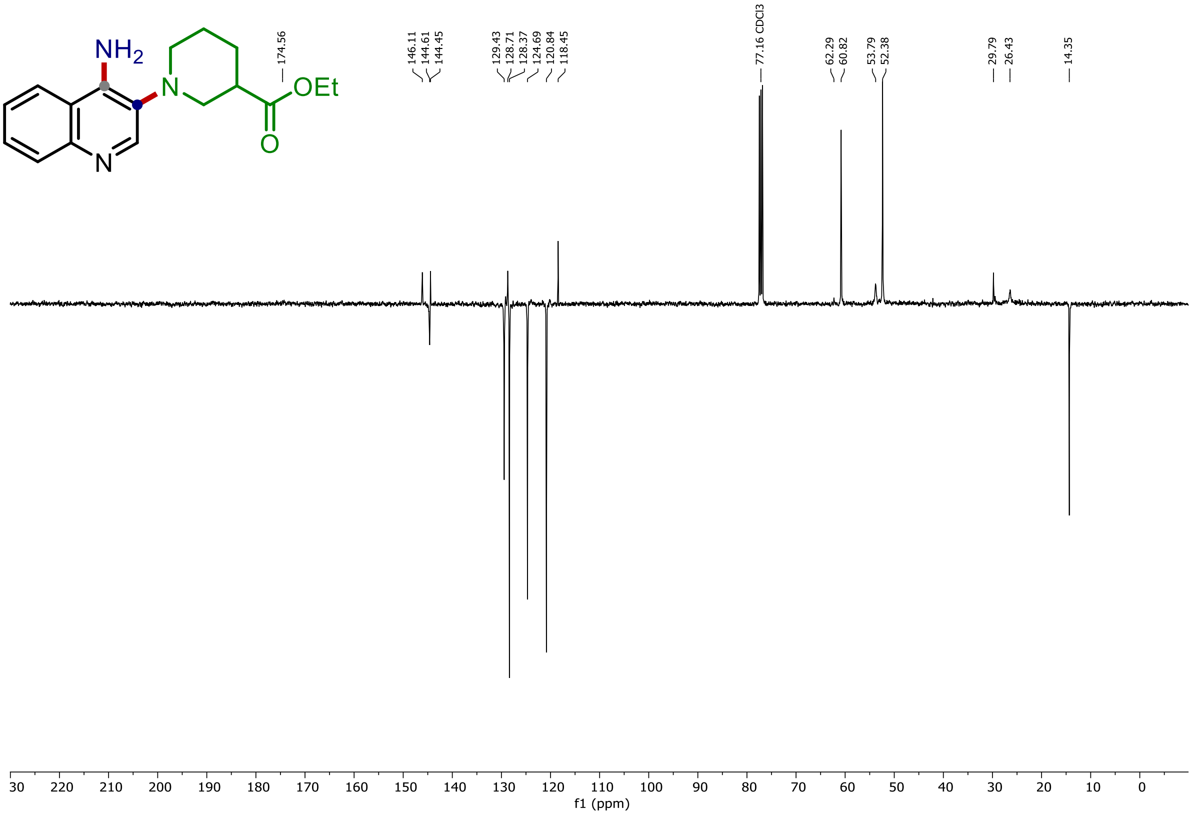

^{1}H NMR (400 MHz, MeOD) of 24 (see procedure)

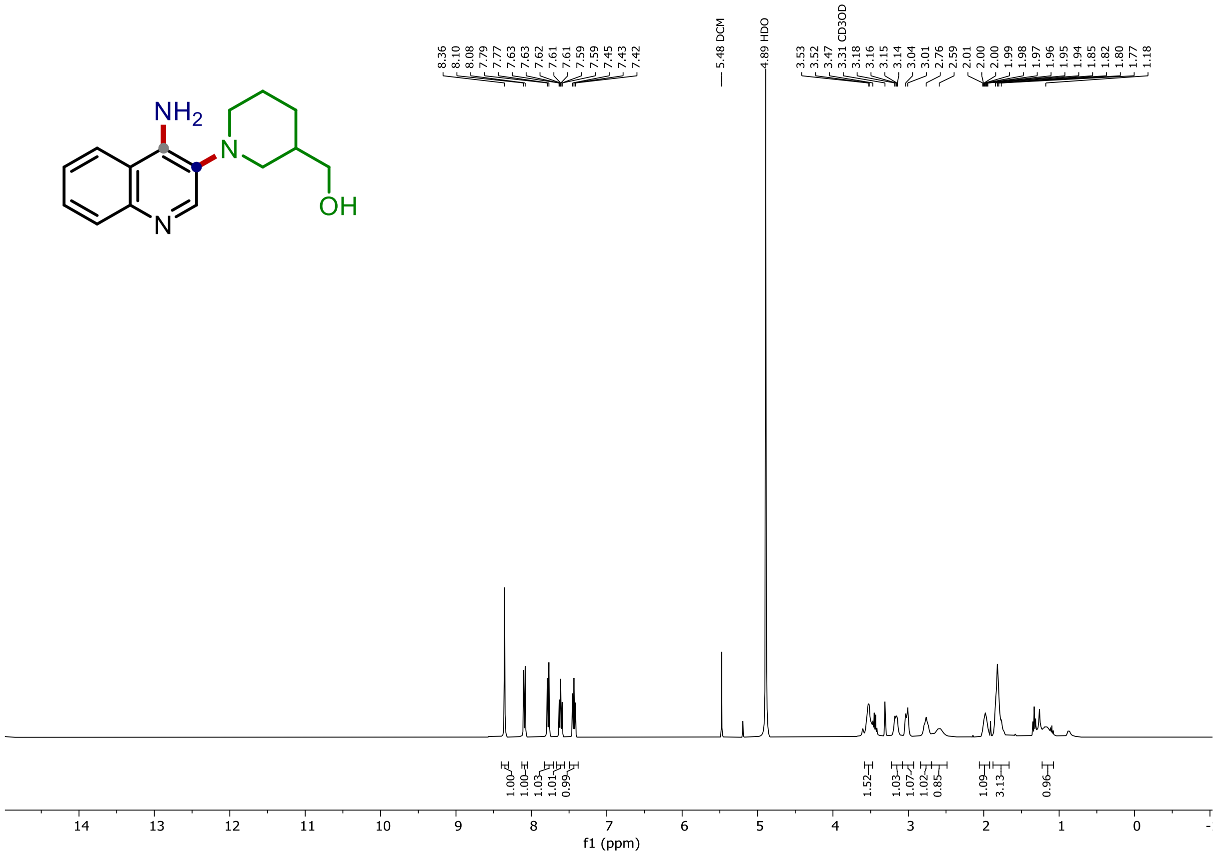

^{13}C{^{1}H} NMR (101 MHz, MeOD) of 24

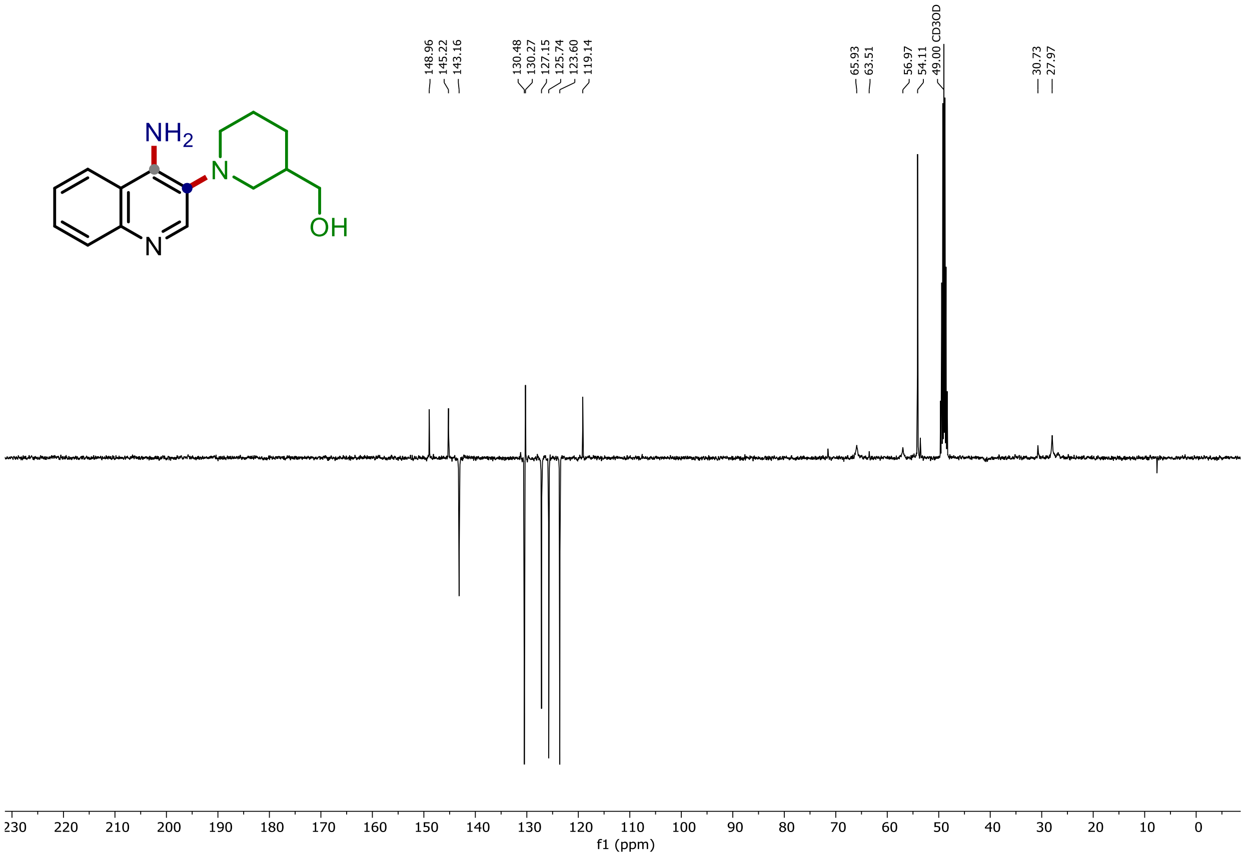

^{1}H NMR (400 MHz, $CDCl_3$) of 25 (see procedure)

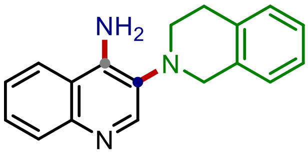

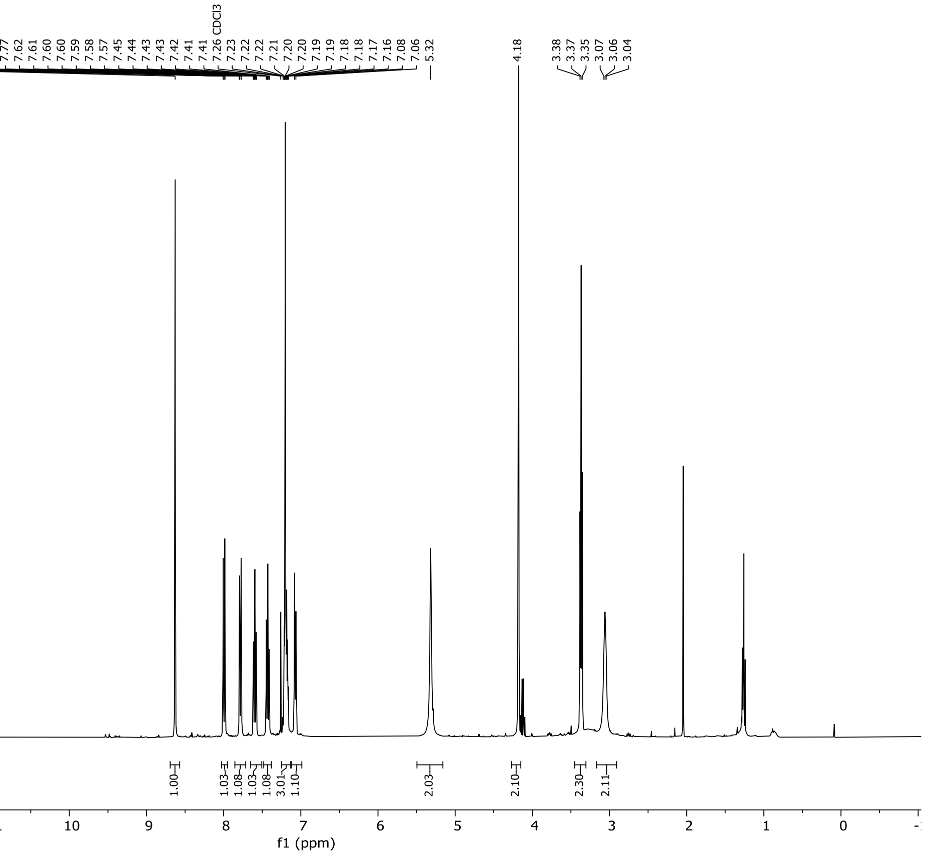

$^{13}C\{^1H\}$ NMR (101 MHz, $CDCl_3$) of 25

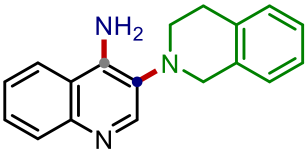

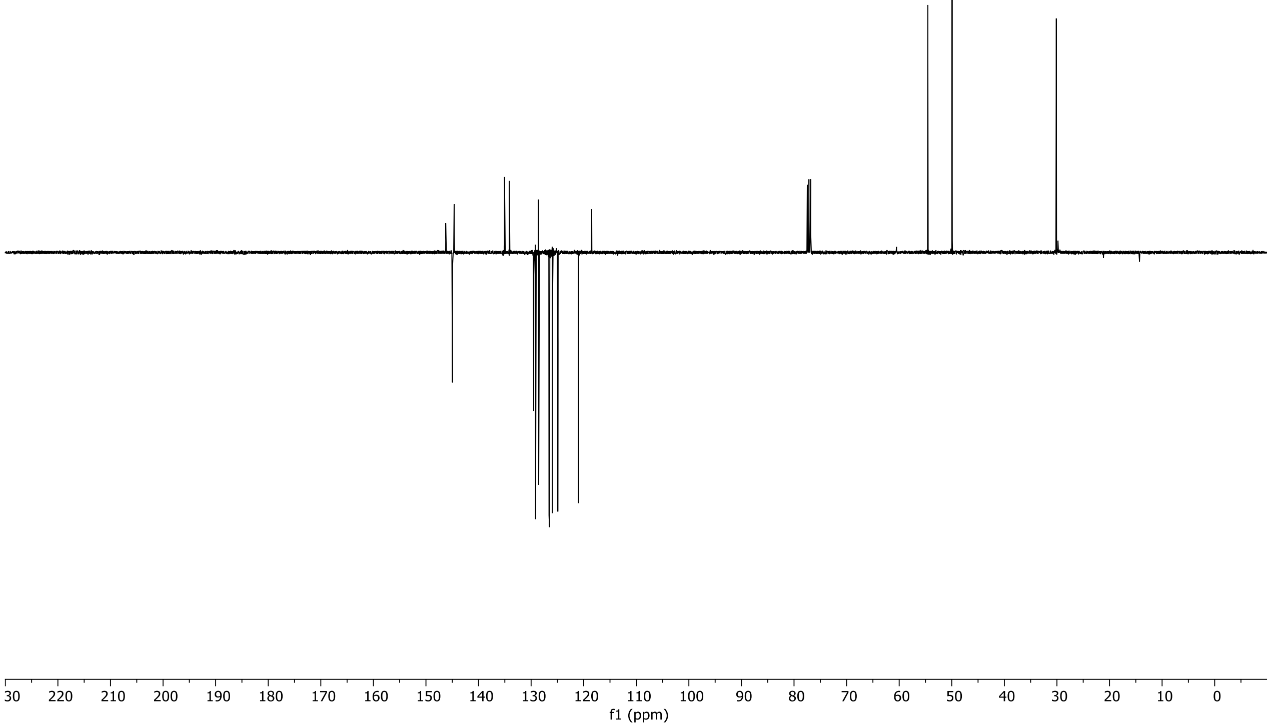

^{1}H NMR (400 MHz, $CDCl_3$) of 26 (see procedure)

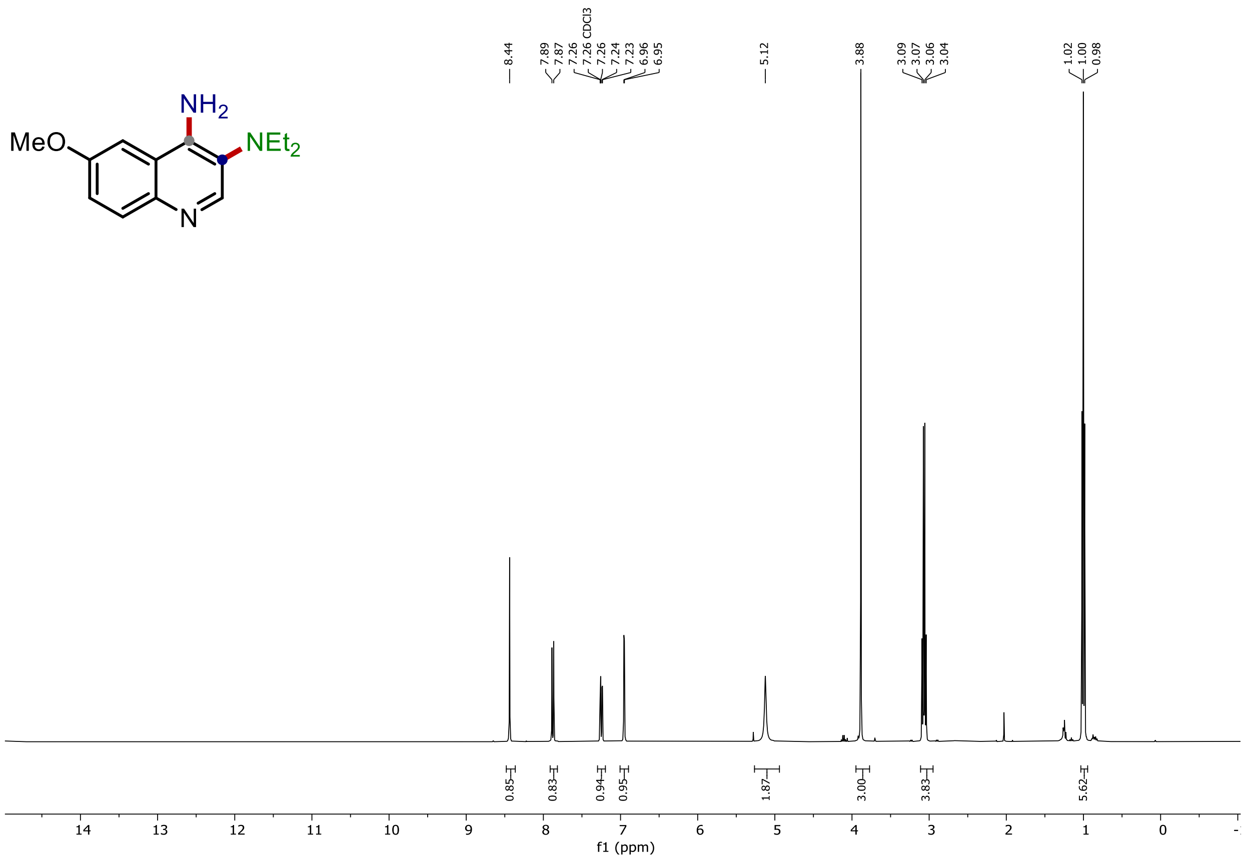

^{13}C{^{1}H} NMR (101 MHz, $CDCl_3$) of 26

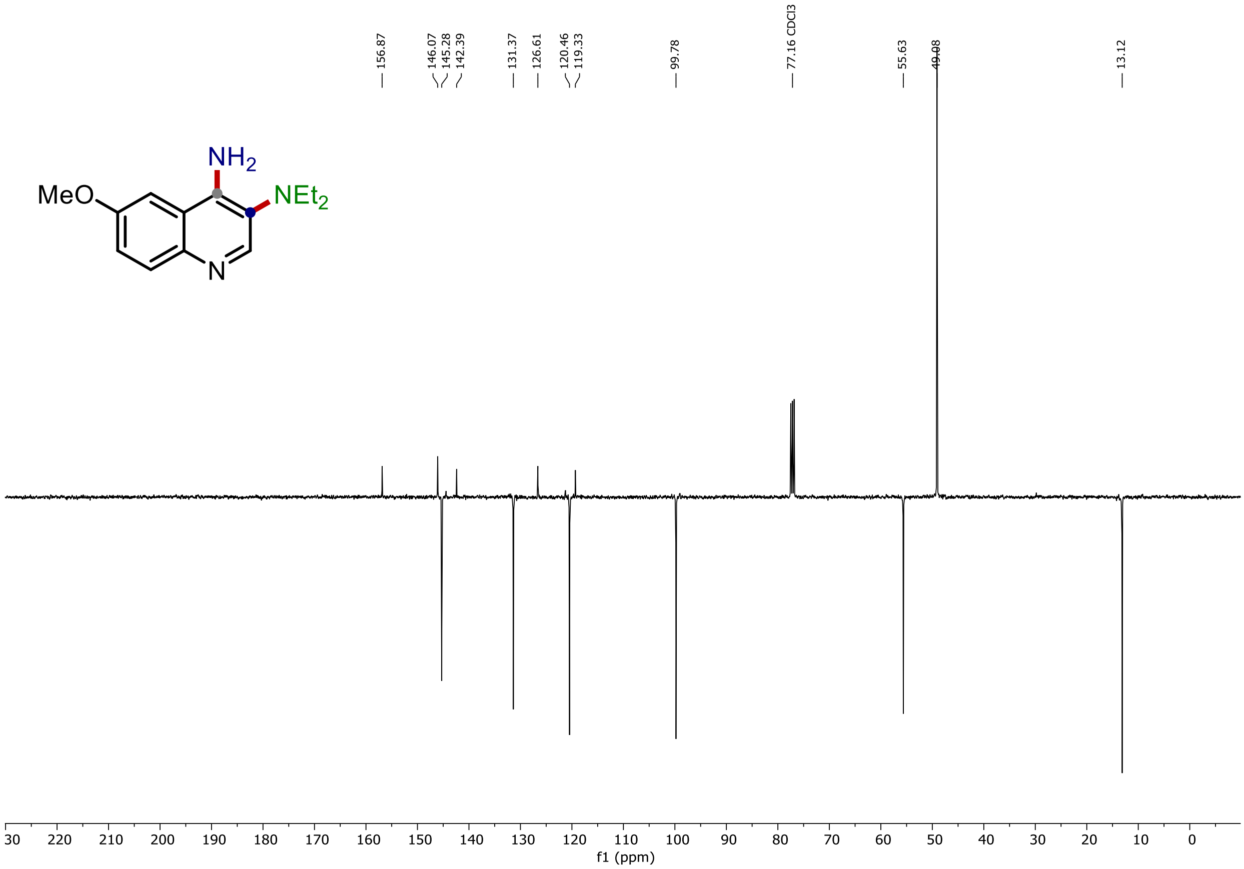

^{1}H NMR (400 MHz, $CDCl_3$) of 27 (see procedure)

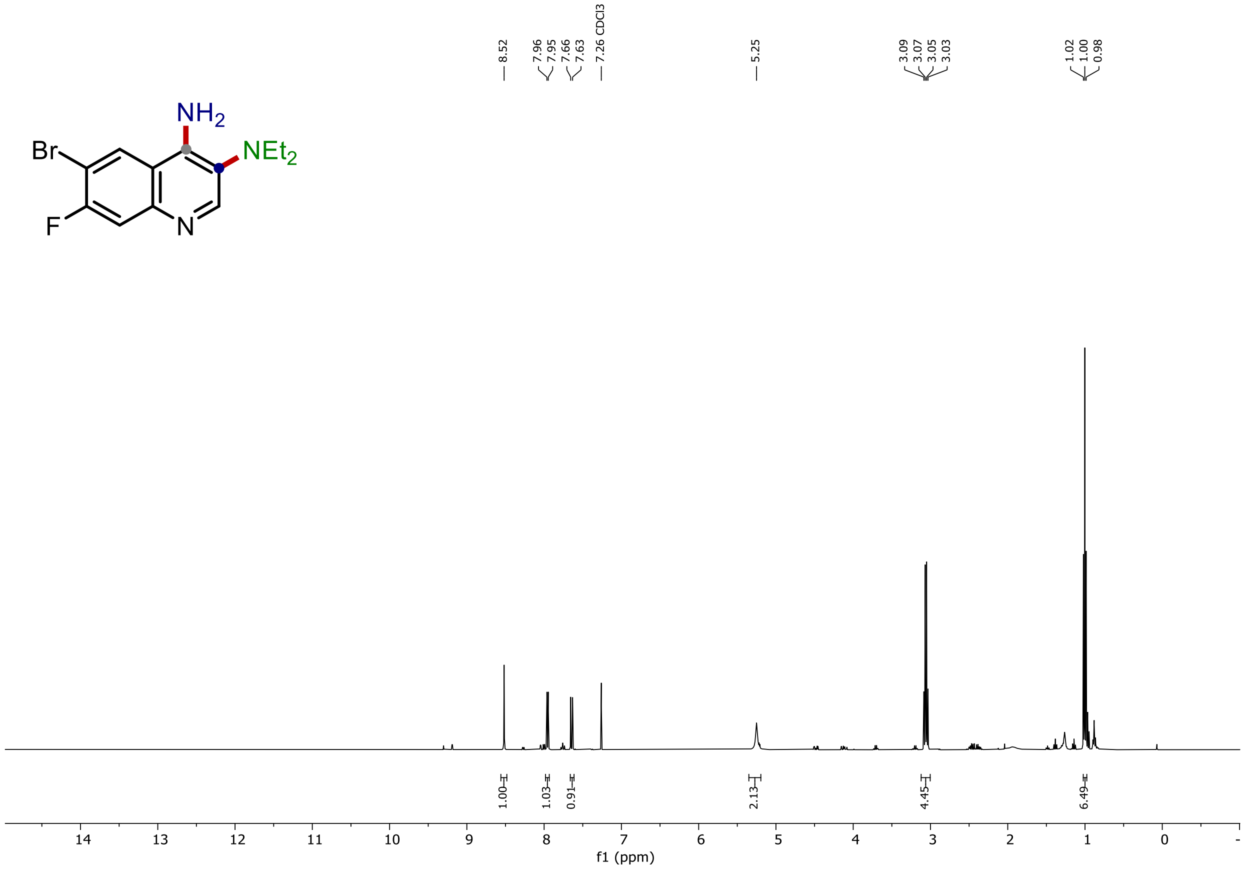

$^{13}C\{^{1}H\}$ NMR (101 MHz, $CDCl_3$) of 27

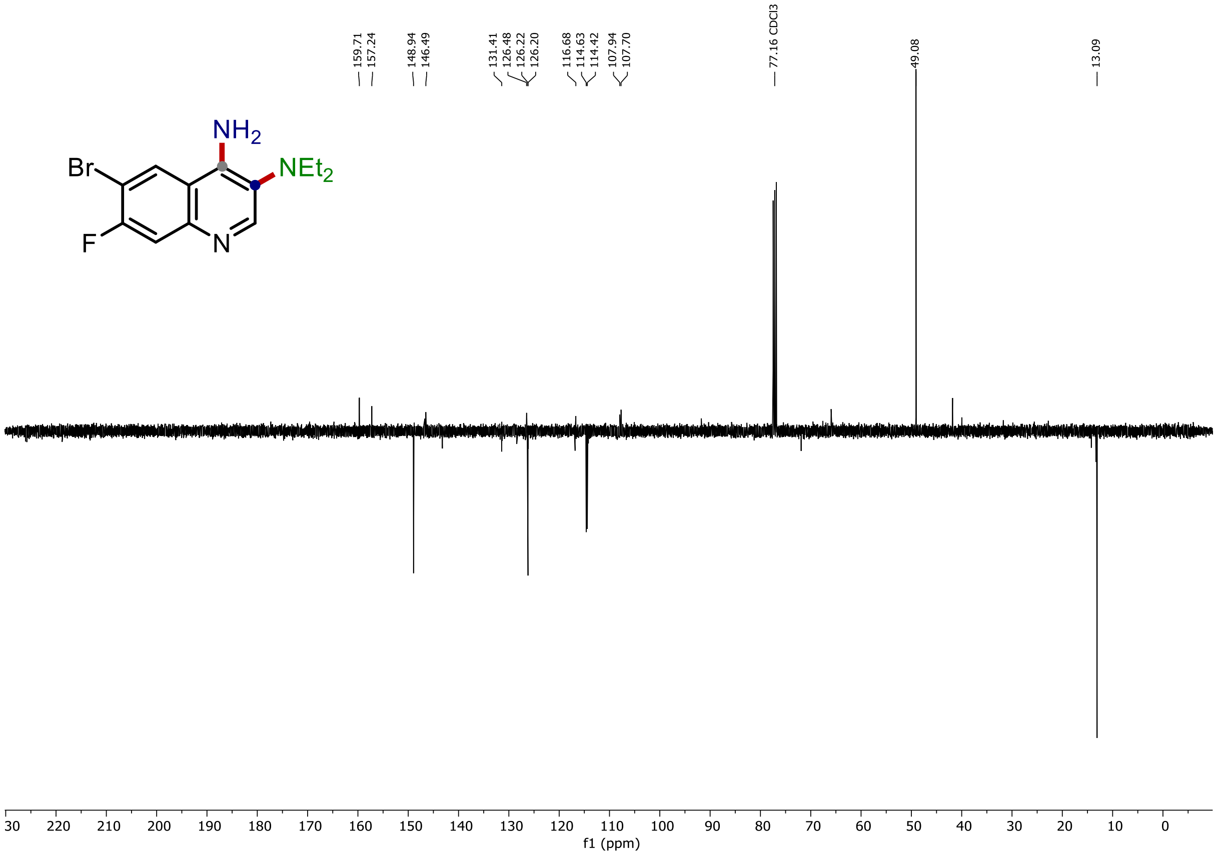

^{19}F NMR (282 MHz, $CDCl_3$) of 27

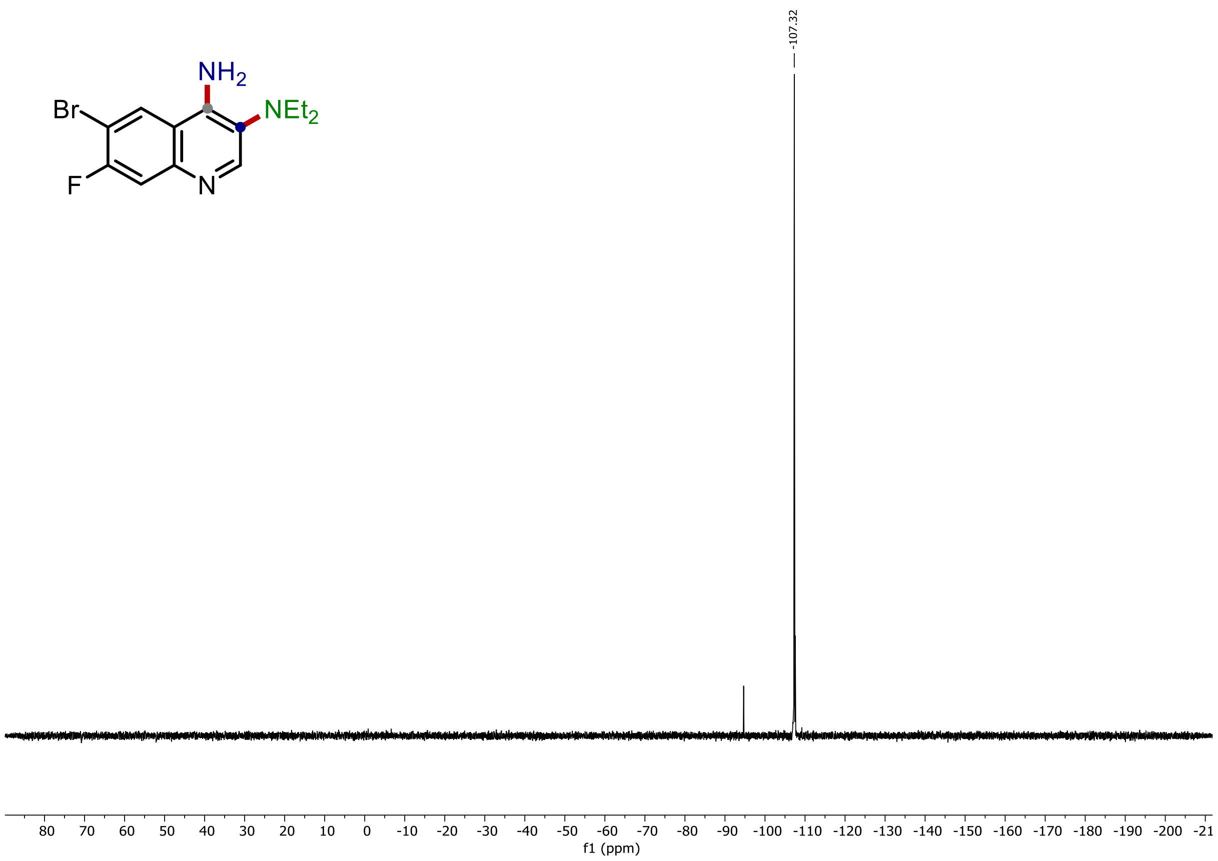

^{1}H NMR (400 MHz, $CDCl_3$) of 28 (see procedure)

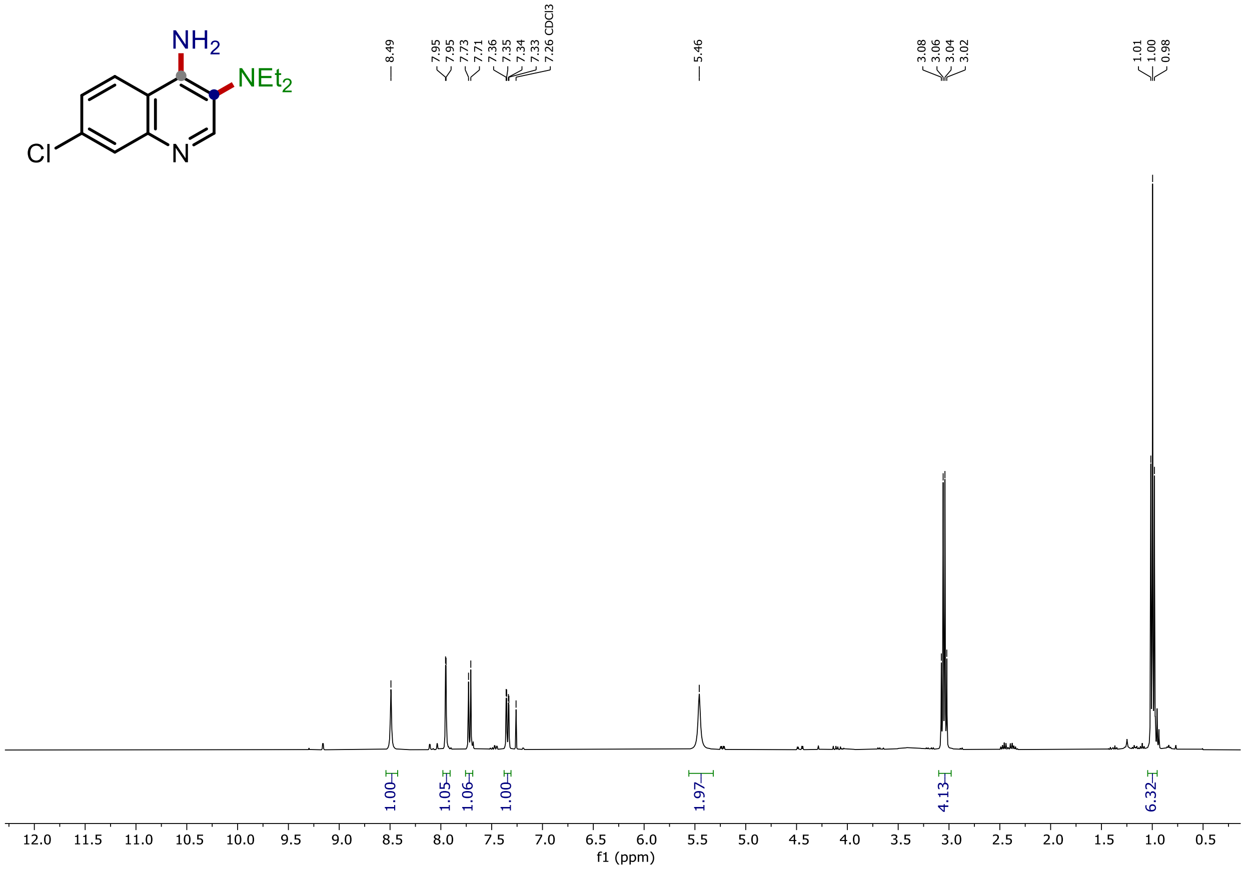

$^{13}C\{^{1}H\}$ NMR (101 MHz, $CDCl_3$) of 28

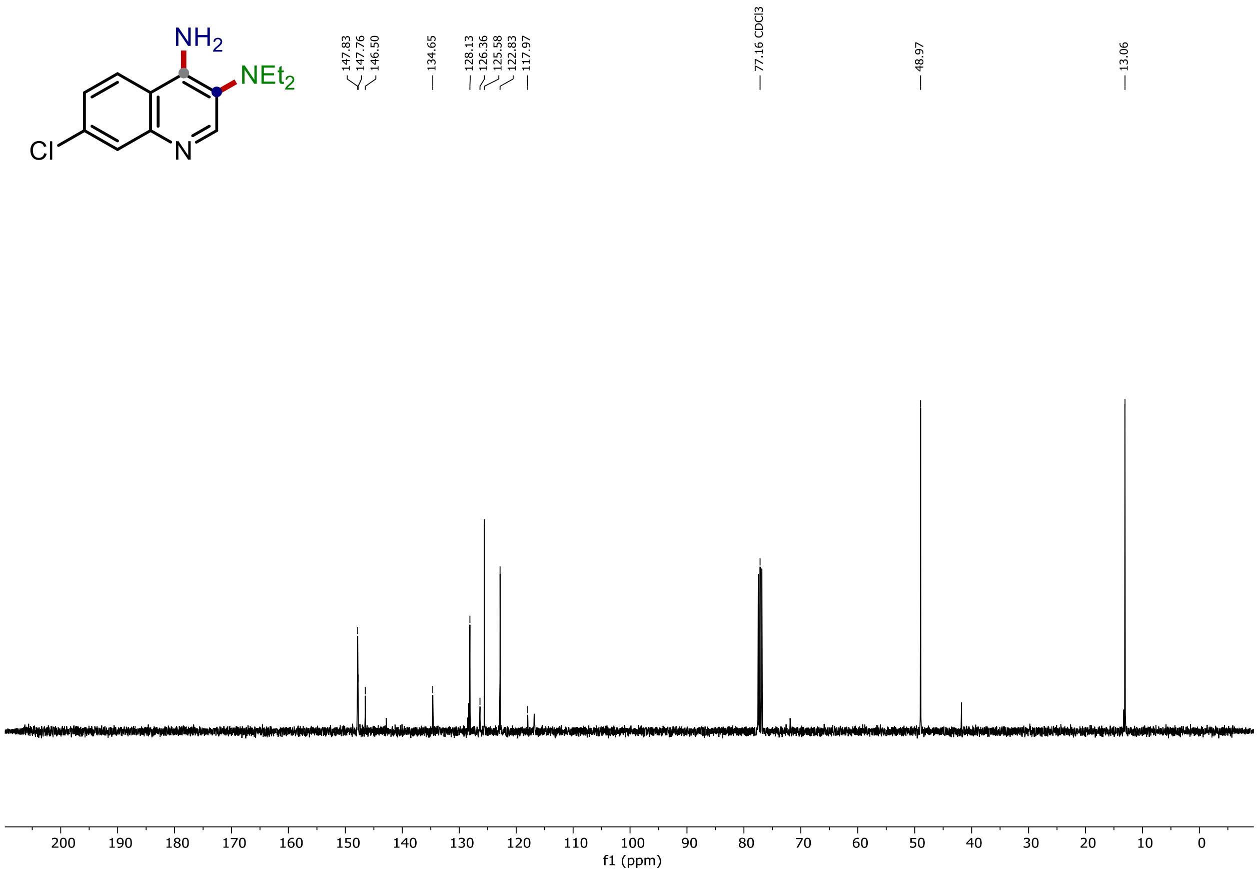

^{1}H NMR (400 MHz, $CDCl_3$) of 29 (see procedure)

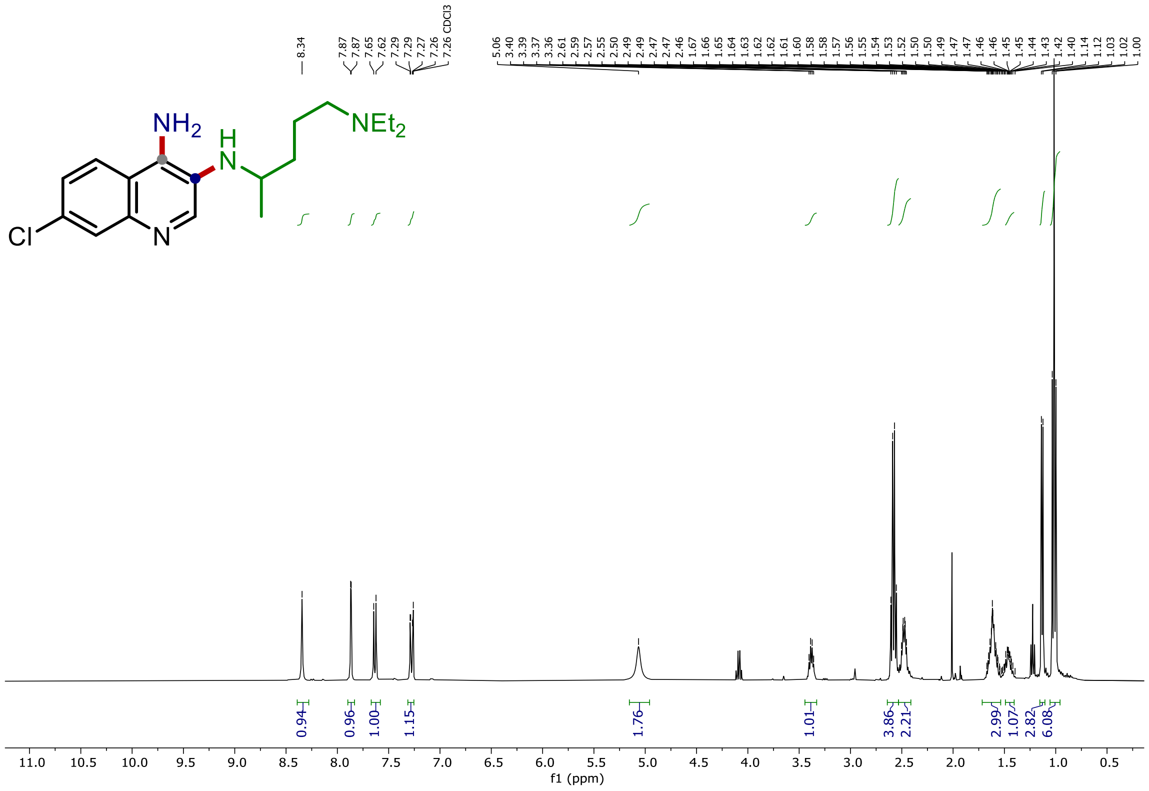

$^{13}C\{^1H\}$ NMR (101 MHz, $CDCl_3$) of 29

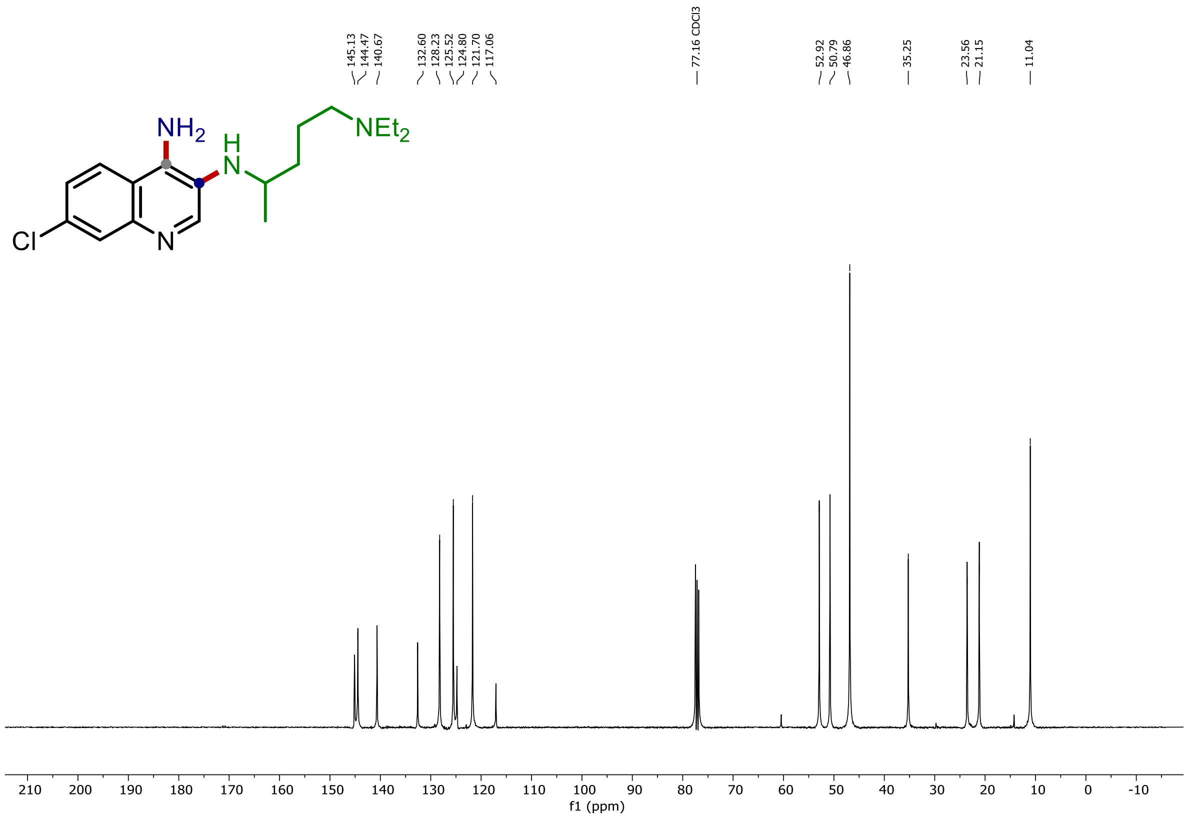

^1H NMR (400 MHz, $CDCl_3$) of 30 (see procedure)

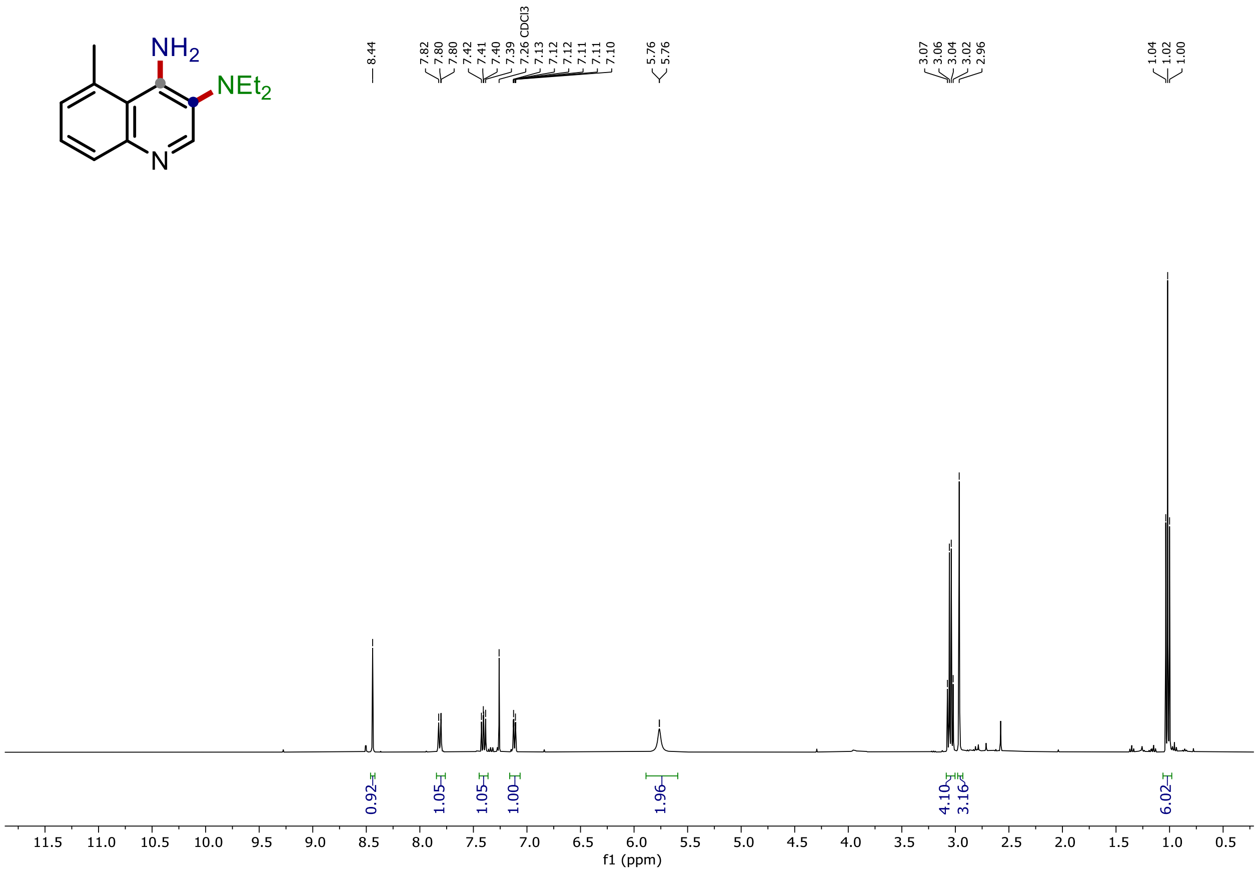

$^{13}C\{^{1}H\}$ NMR (101 MHz, $CDCl_3$) of 30

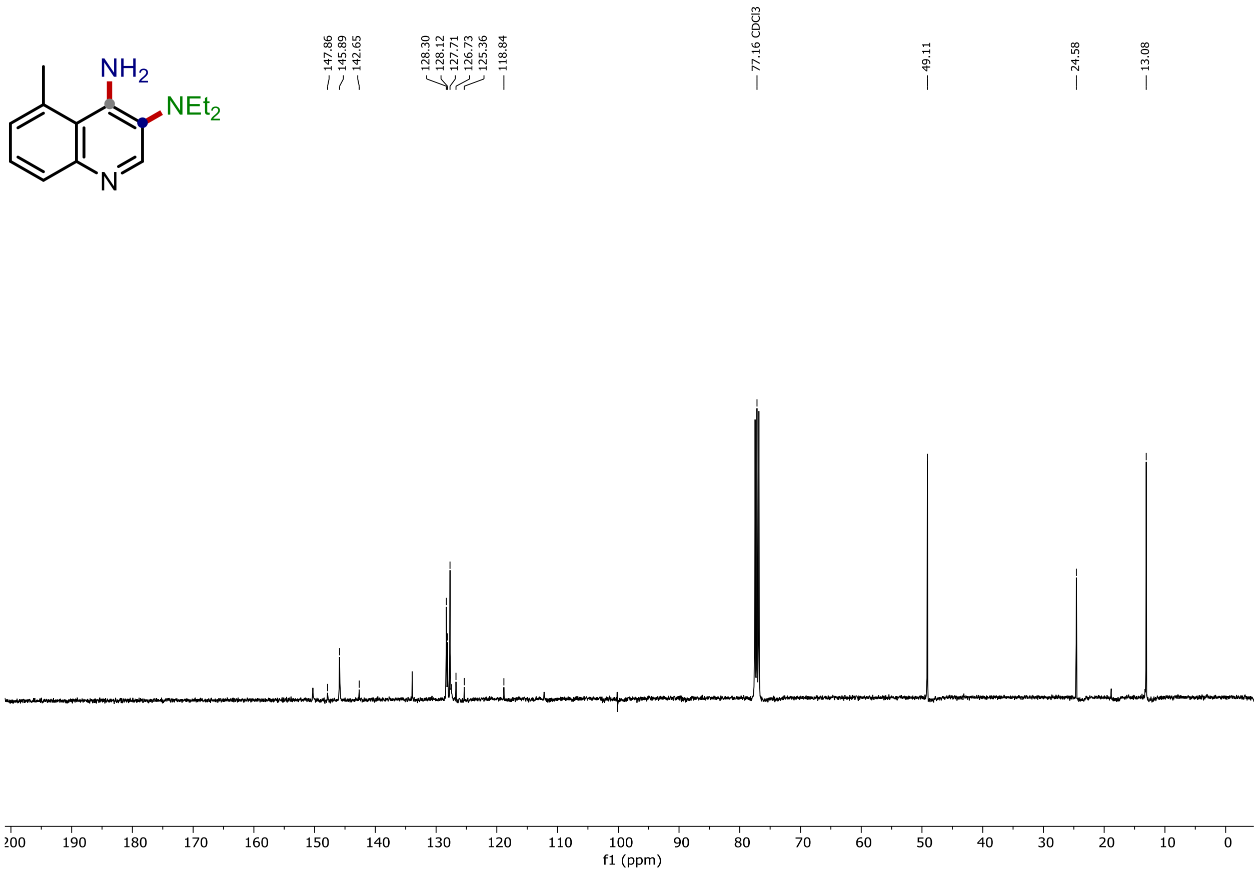

^{1}H NMR (400 MHz, $CDCl_3$) of 31 (see procedure)

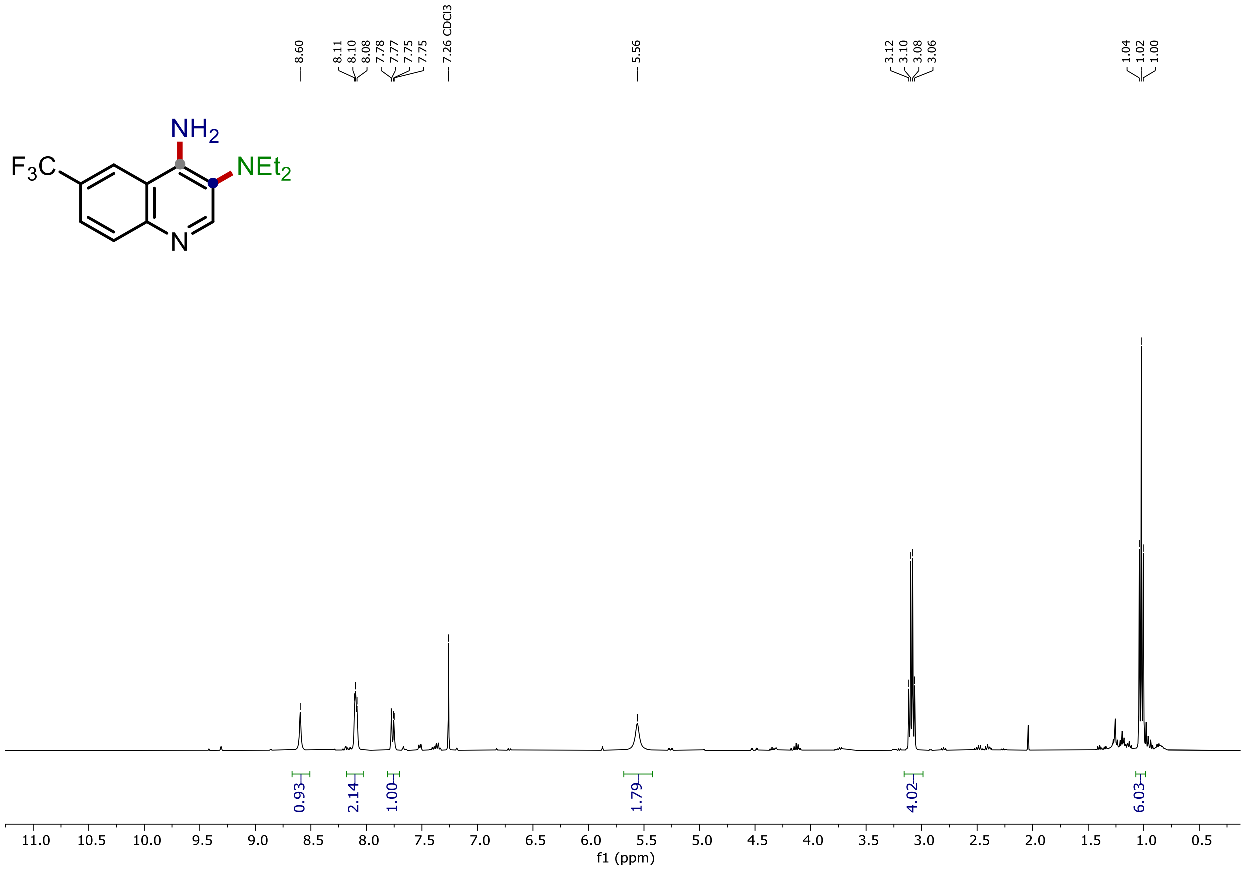

$^{13}C\{^1H\}$ NMR (101 MHz, $CDCl_3$) of 31

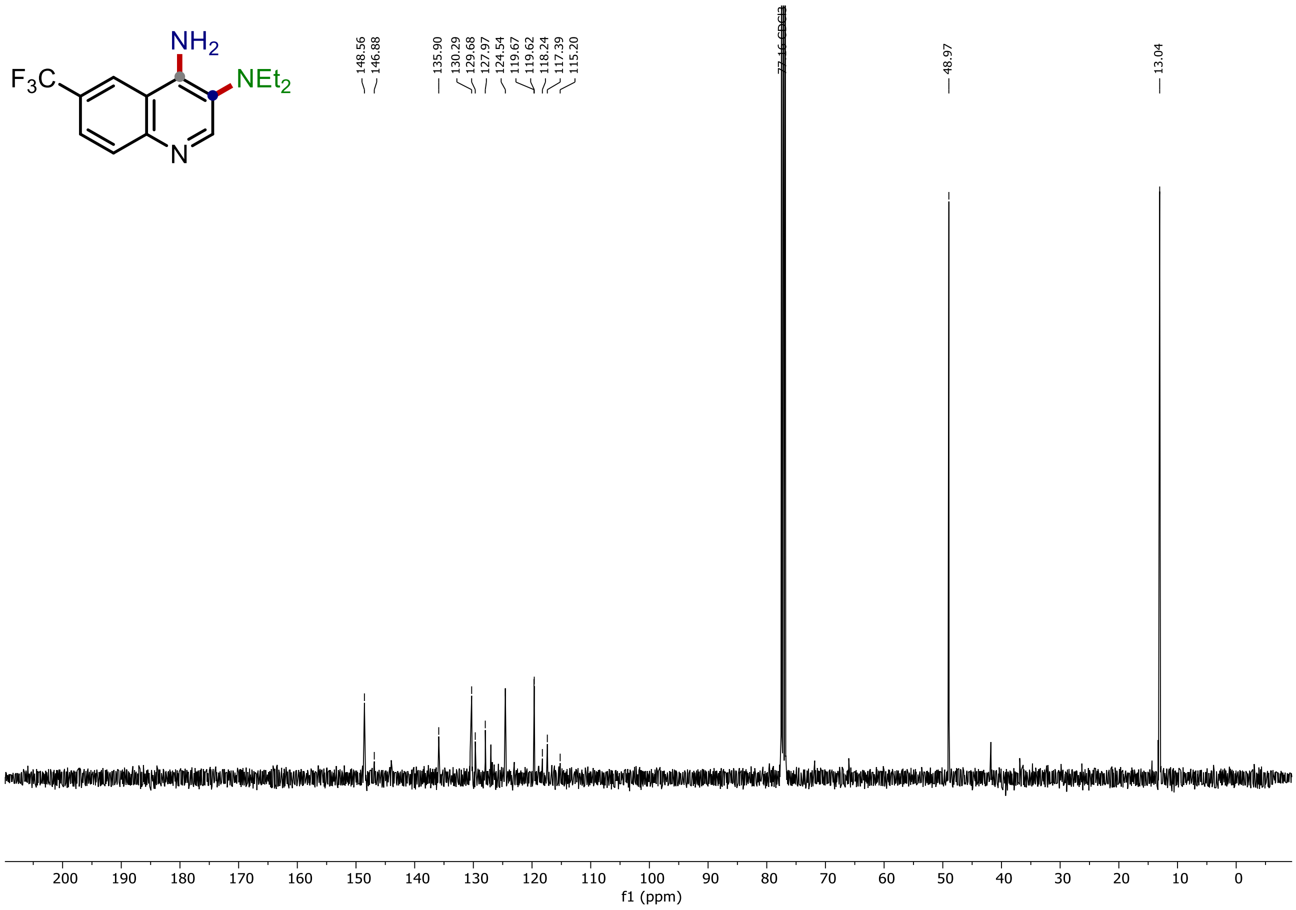

^1H NMR (400 MHz, $CDCl_3$) of 32 (see procedure)

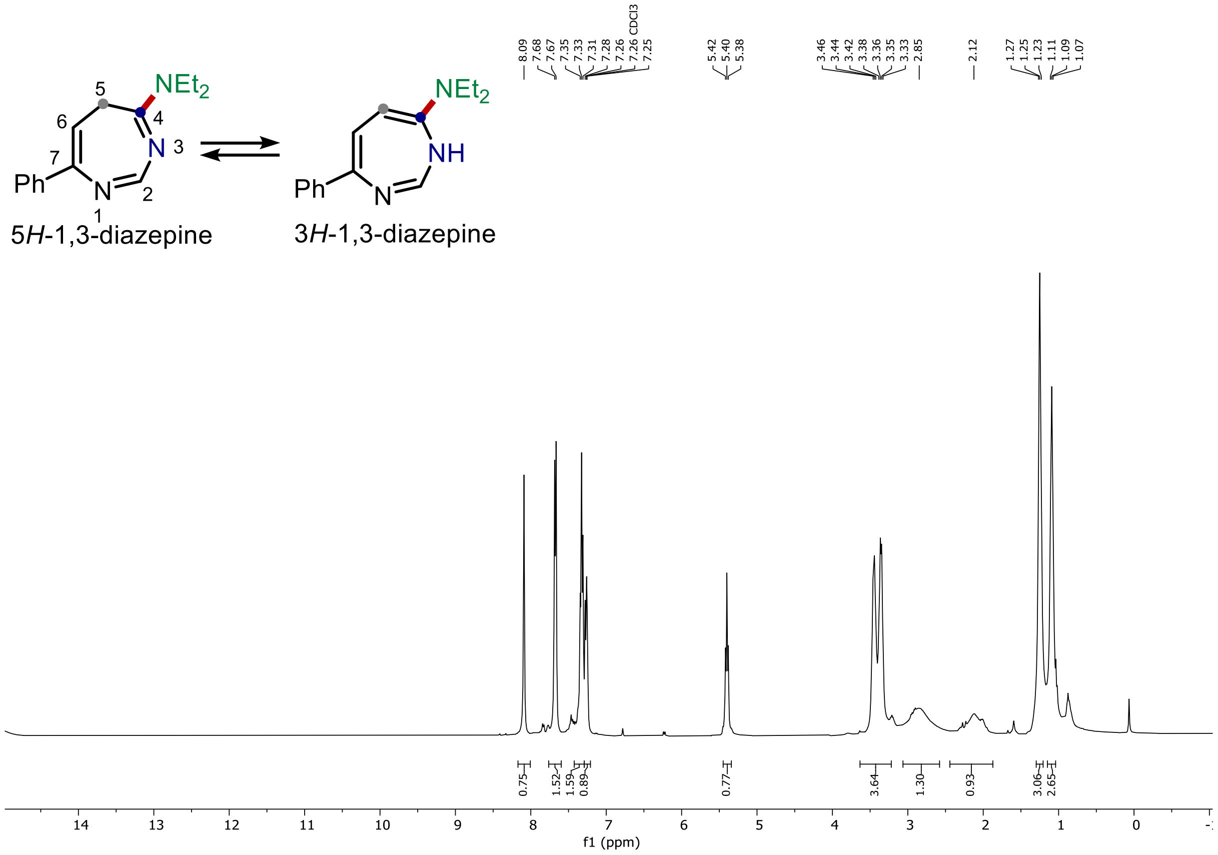

^{1}H NMR (400 MHz, CD_2Cl_2, −40°C) of 32

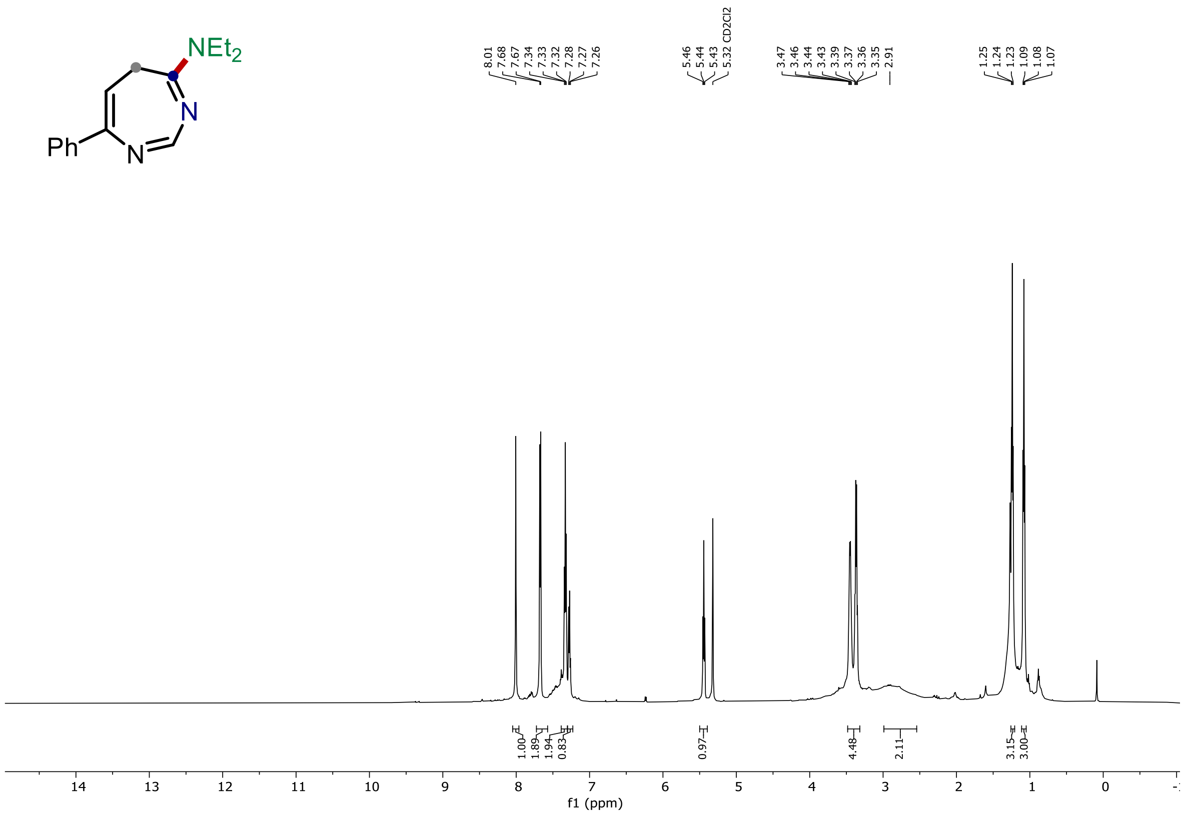

^{1}H NMR (400 MHz, $CDCl_3$) of 33 (see procedure)

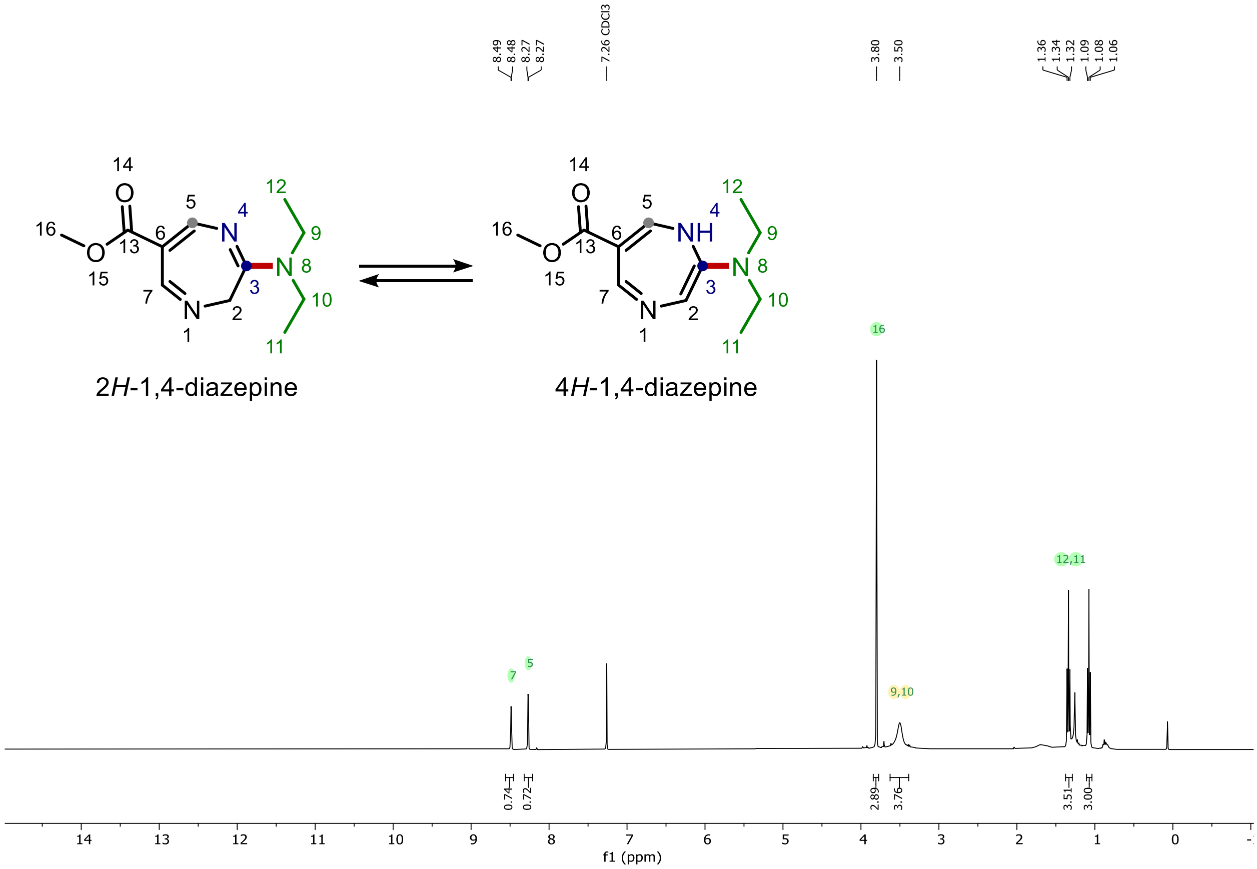

^{1}H NMR (400 MHz, CD_2Cl_2, −40°C) of 33

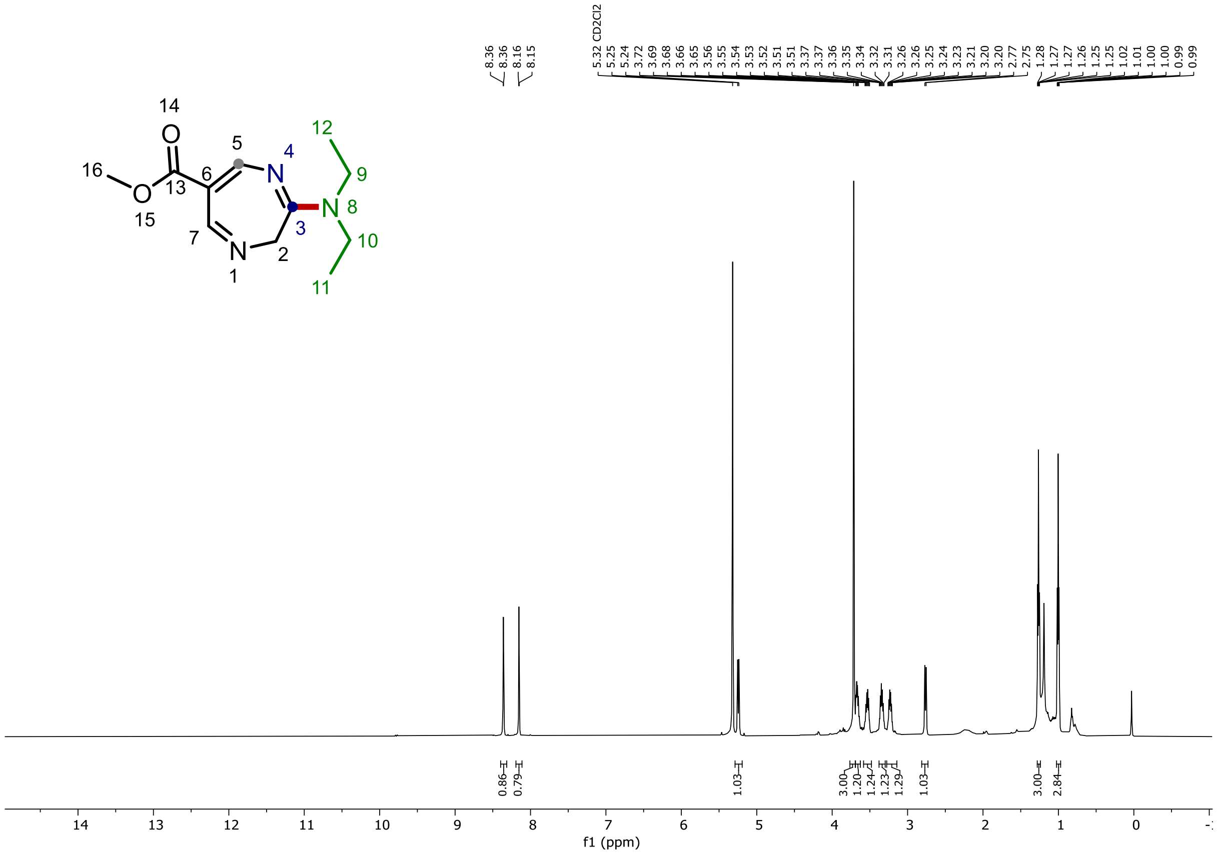

$^{13}C\{^{1}H\}$ NMR (101 MHz, $CDCl_3$) of 33

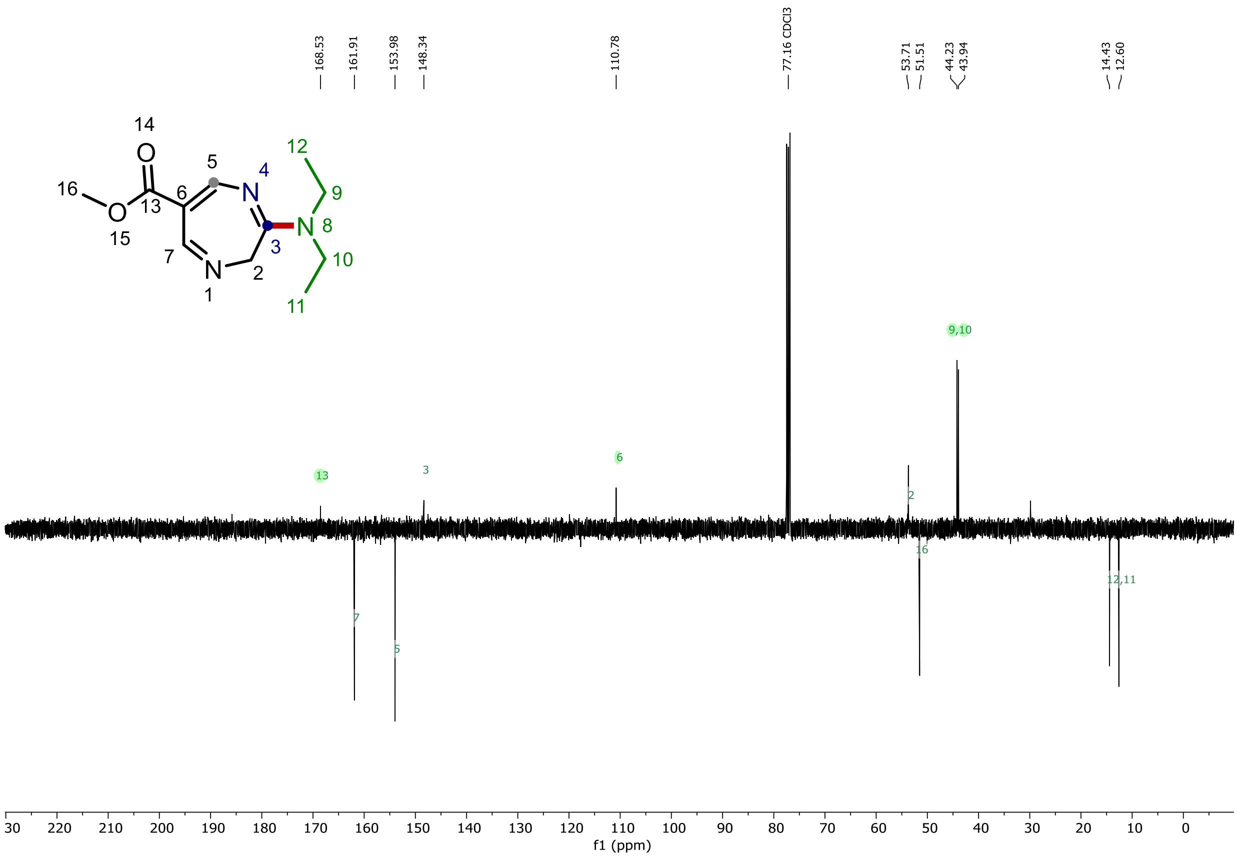

^{13}C-^{1}H HMBC (101 MHz, CDCl$_3$) of 33

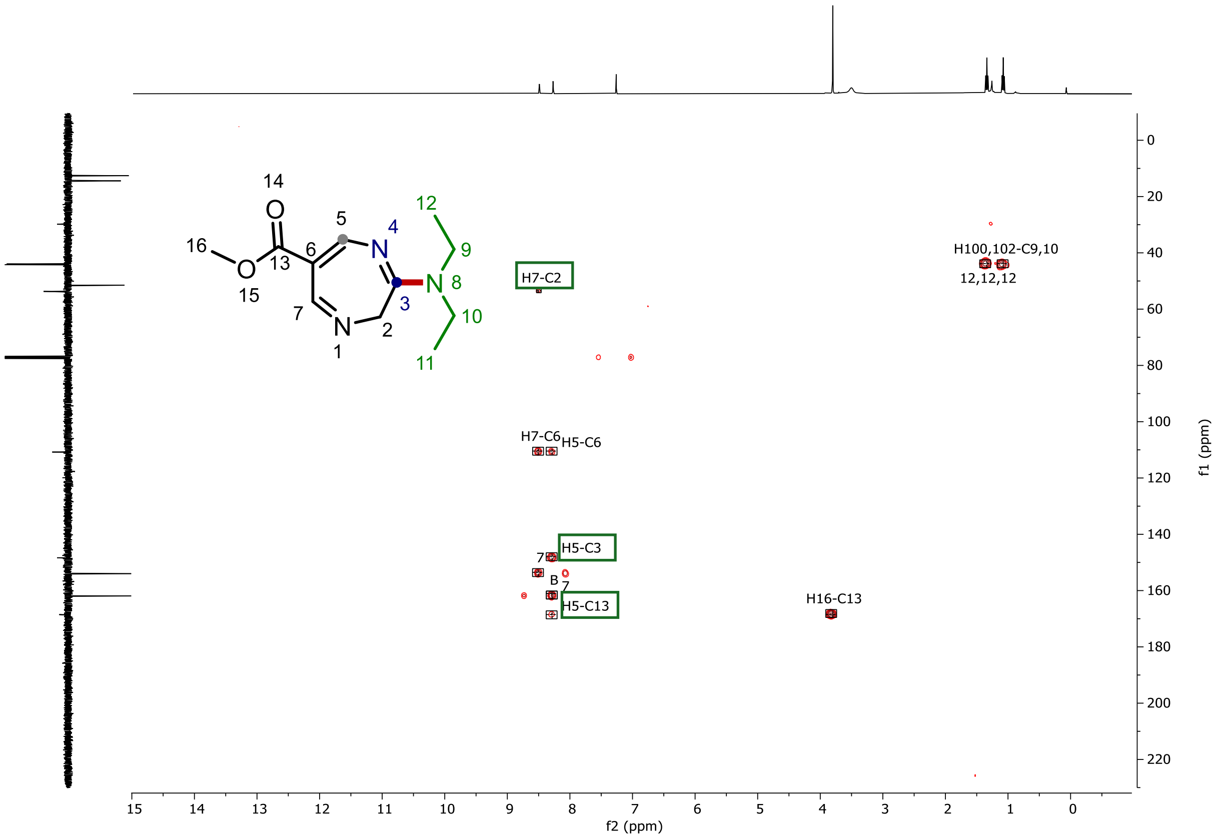

^{1}H NMR (400 MHz, CDCl$_3$) of 34 (see procedure)

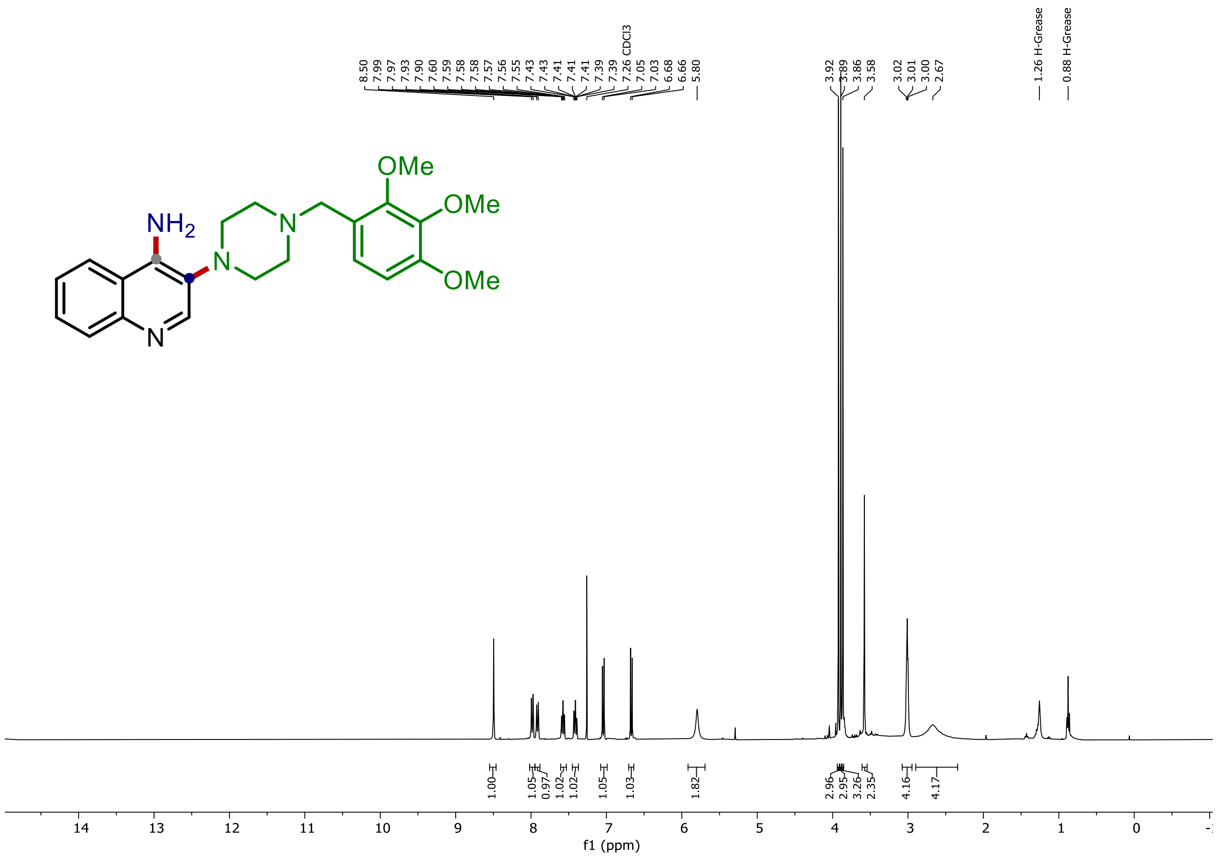

$^{13}C\{^{1}H\}$ NMR (101 MHz, $CDCl_3$) of 34

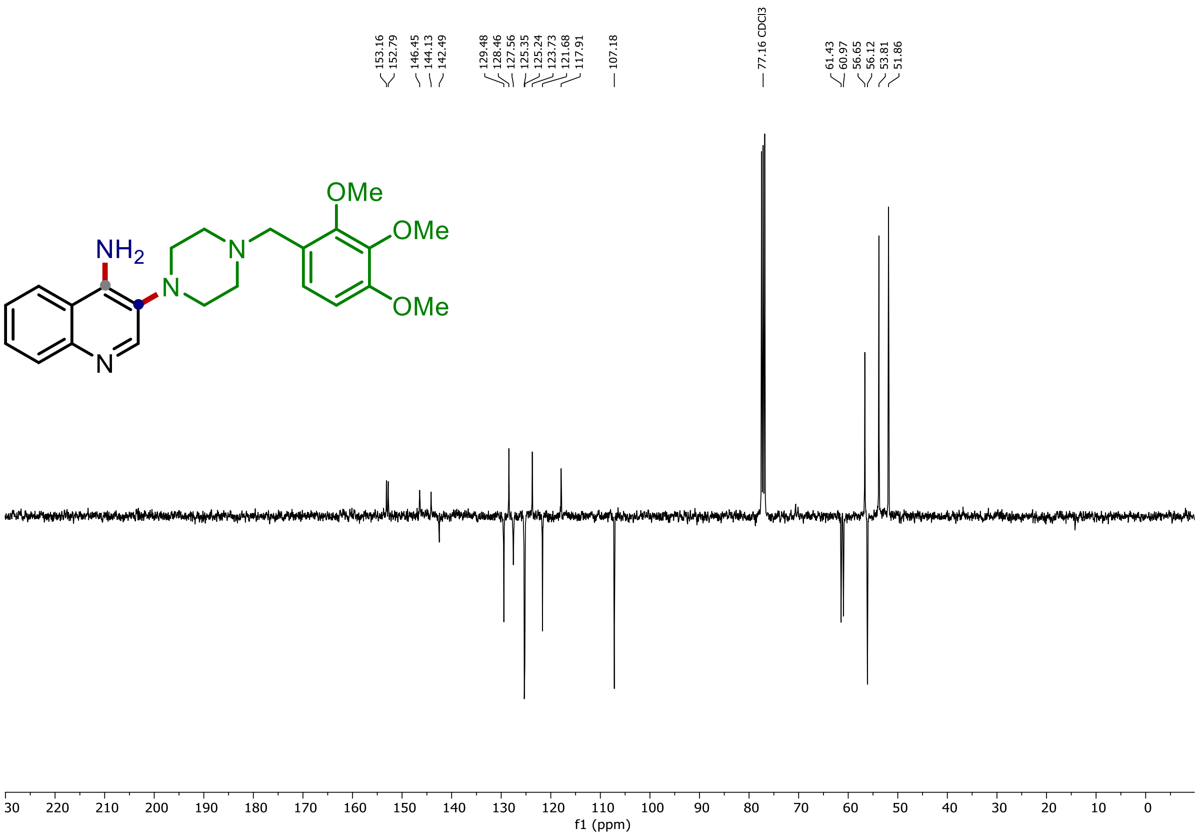

^{1}H NMR (400 MHz, $CDCl_3$) of 35 (see procedure)

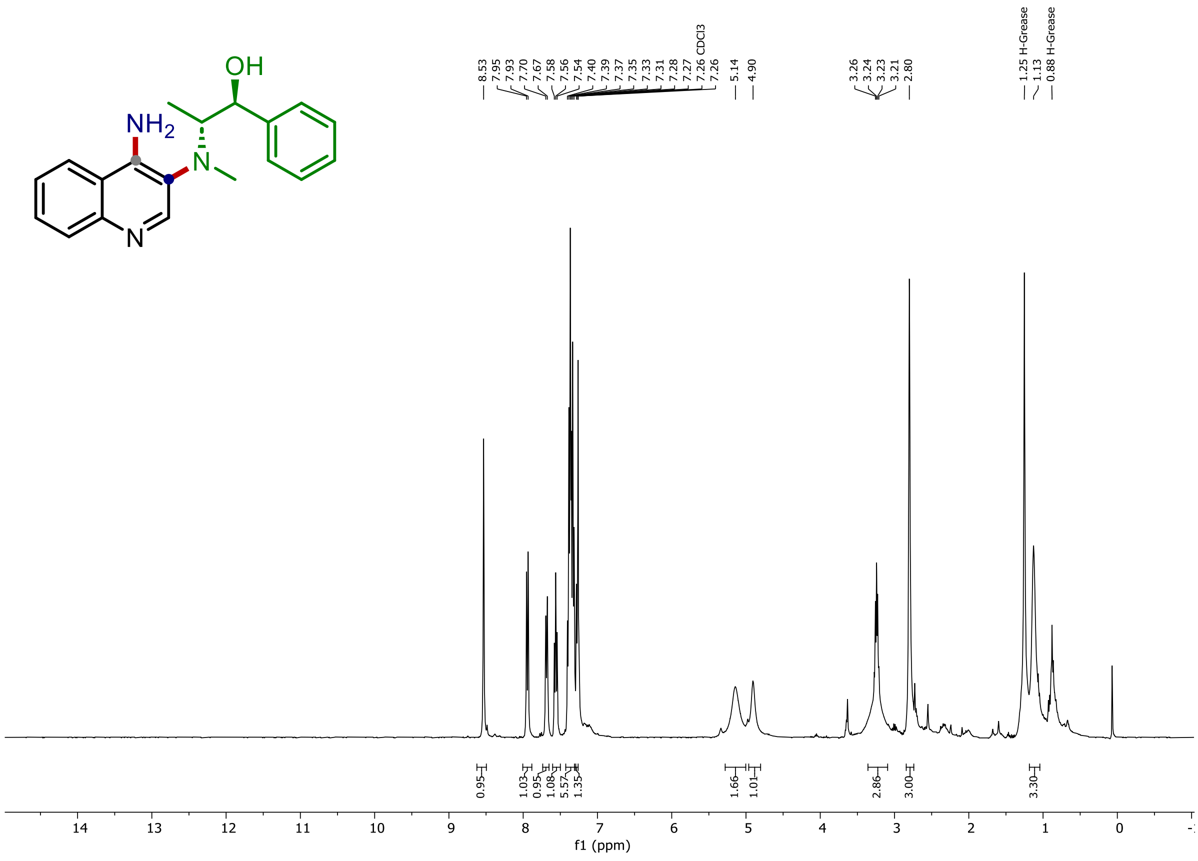

$^{13}C\{^1H\}$ NMR (101 MHz, $CDCl_3$) of 35

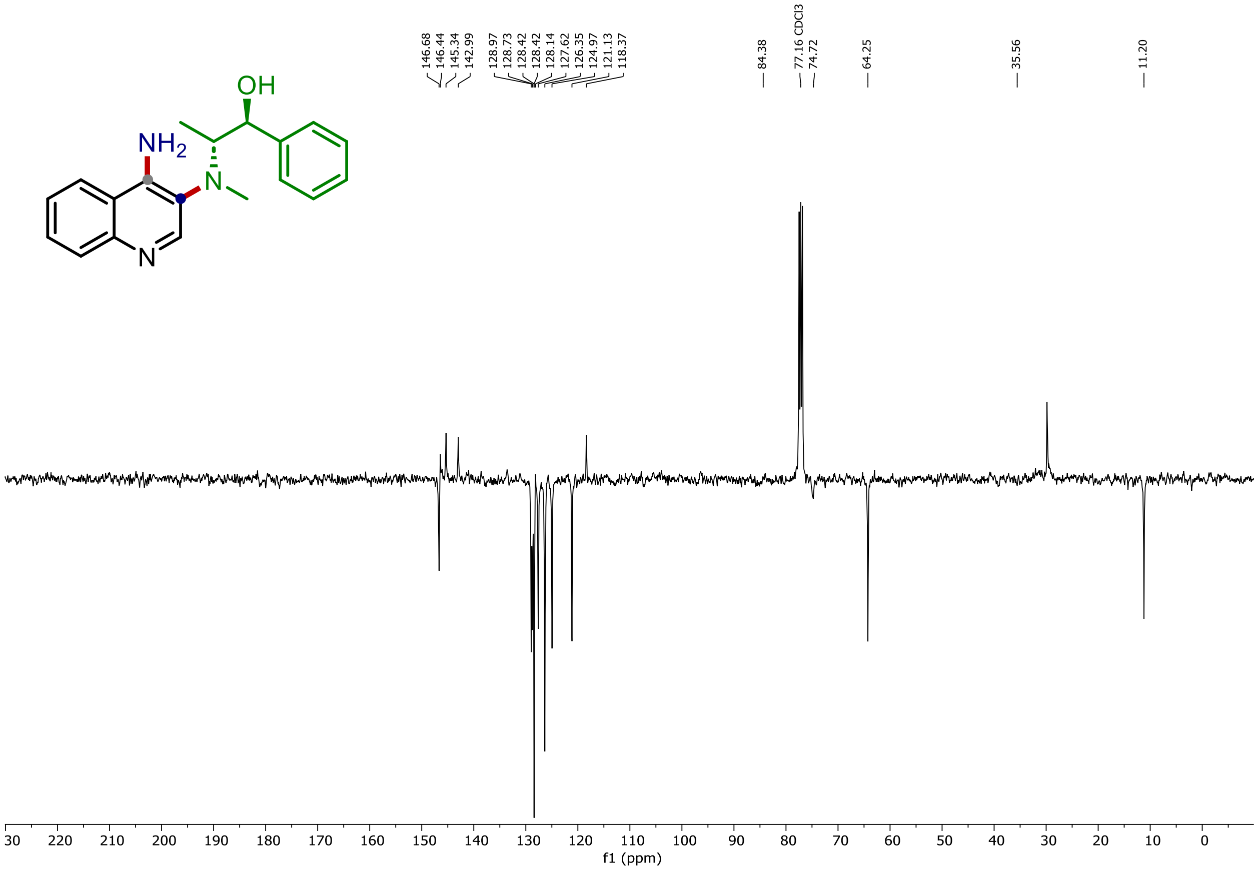

^1H NMR (400 MHz, $CDCl_3$) of 36 (see procedure)

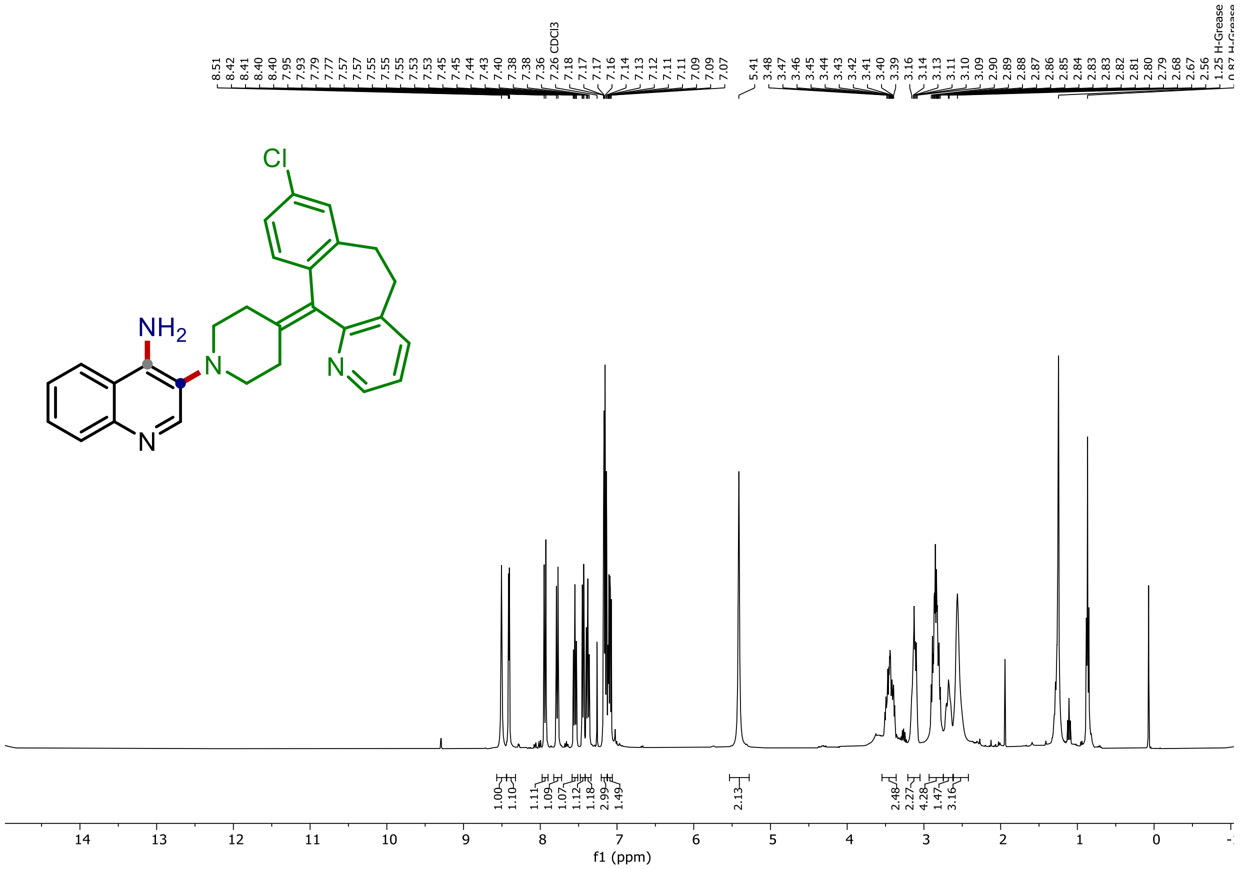

$^{13}C\{^{1}H\}$ NMR (101 MHz, $CDCl_3$) of 36

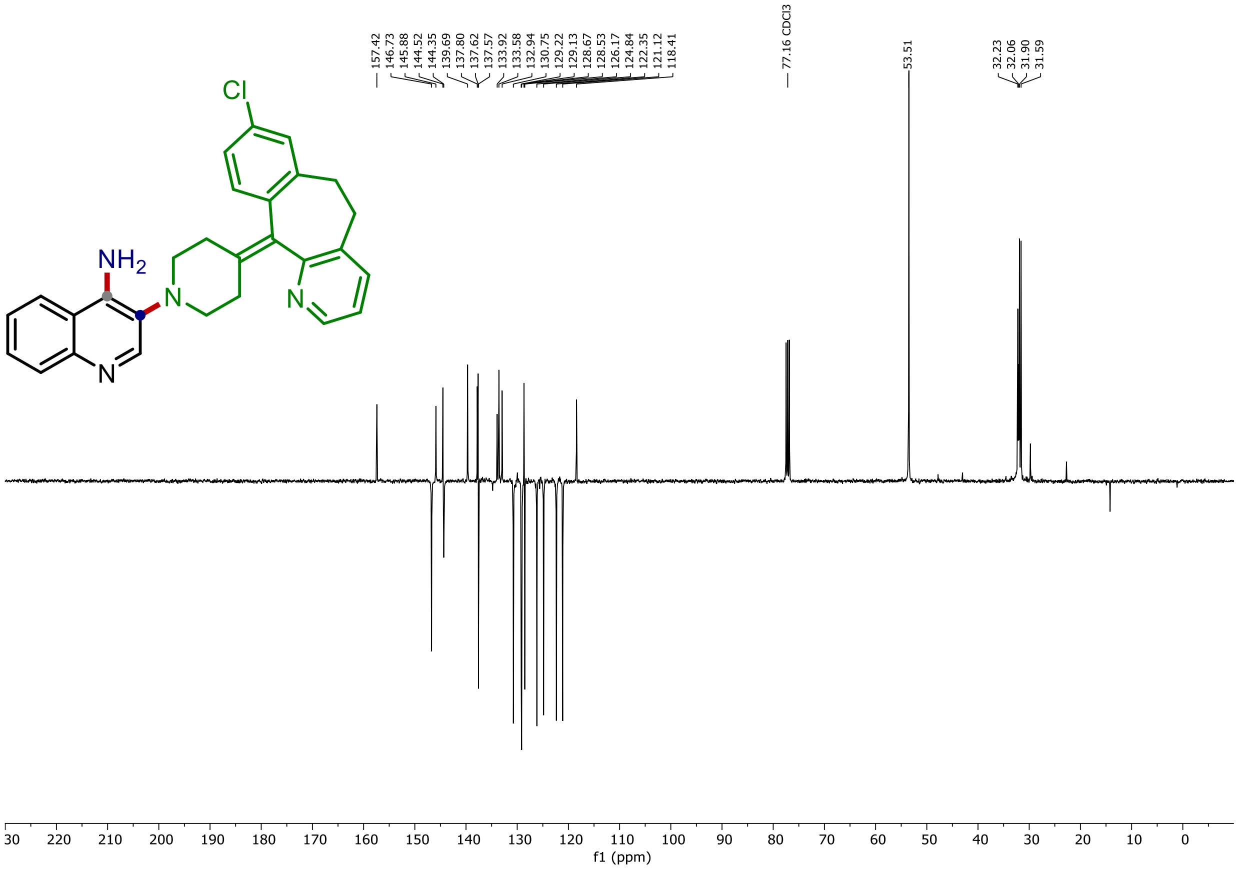

^{1}H NMR (400 MHz, DMSO-d_6) of 2a* (see procedure)

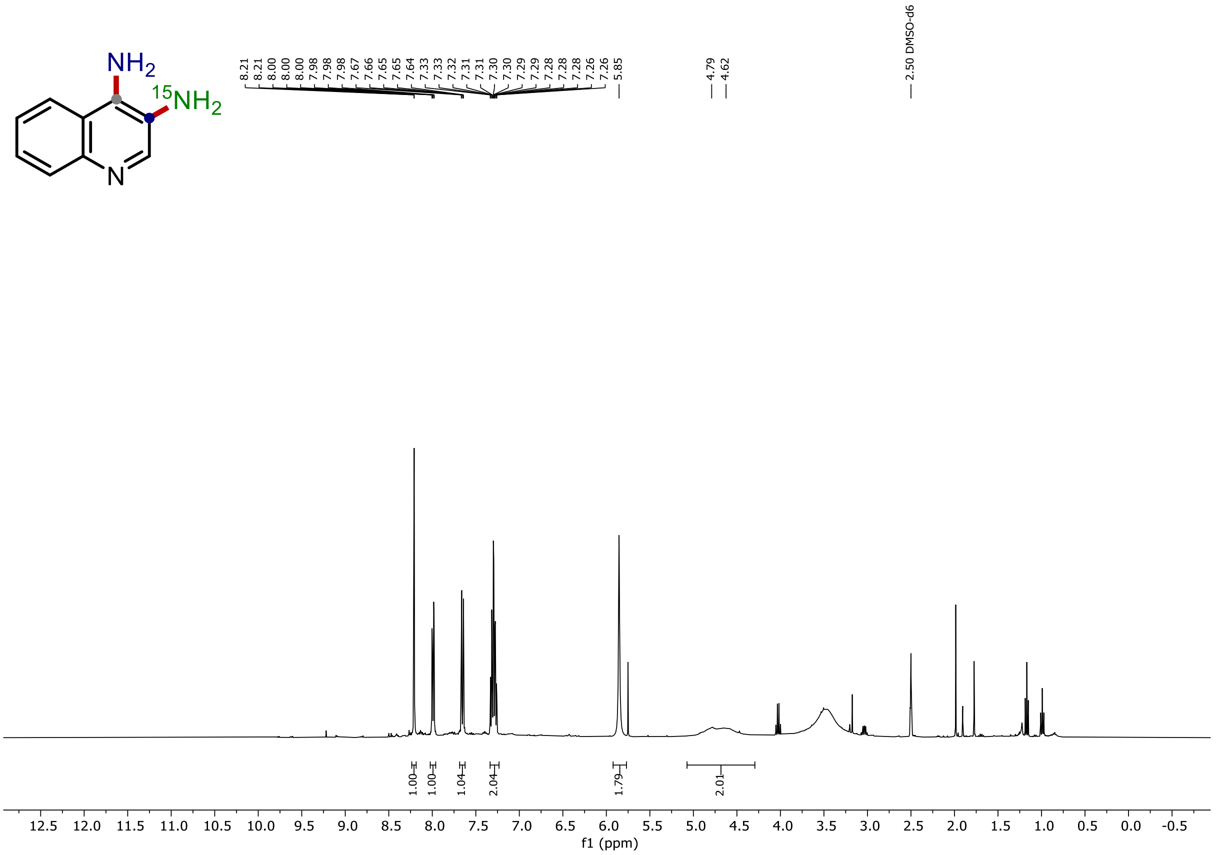

$^{13}C\{^{1}H\}$ NMR (400 MHz, DMSO-d_6) of 2a*

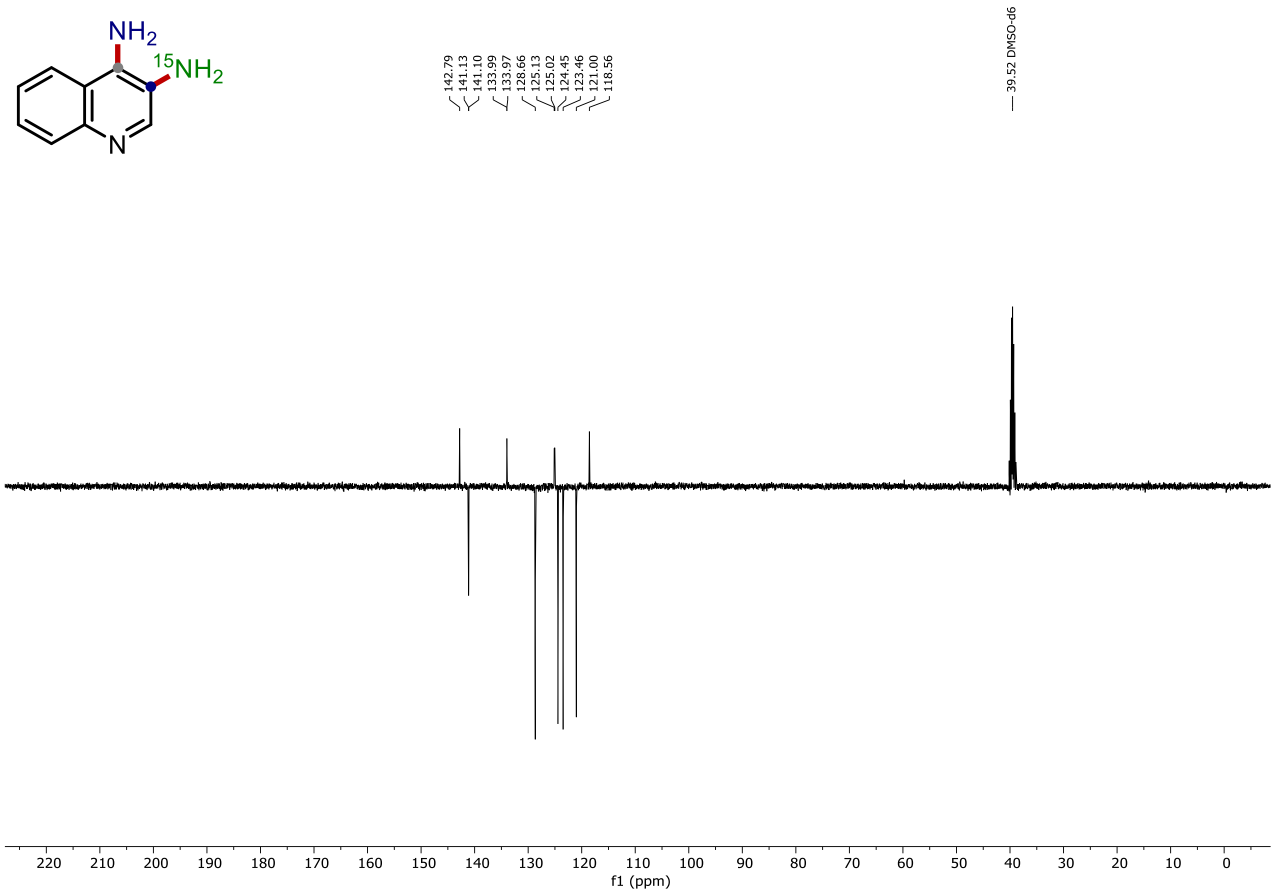

^{15}N NMR (41 MHz, DMSO-d_6) of 2a*

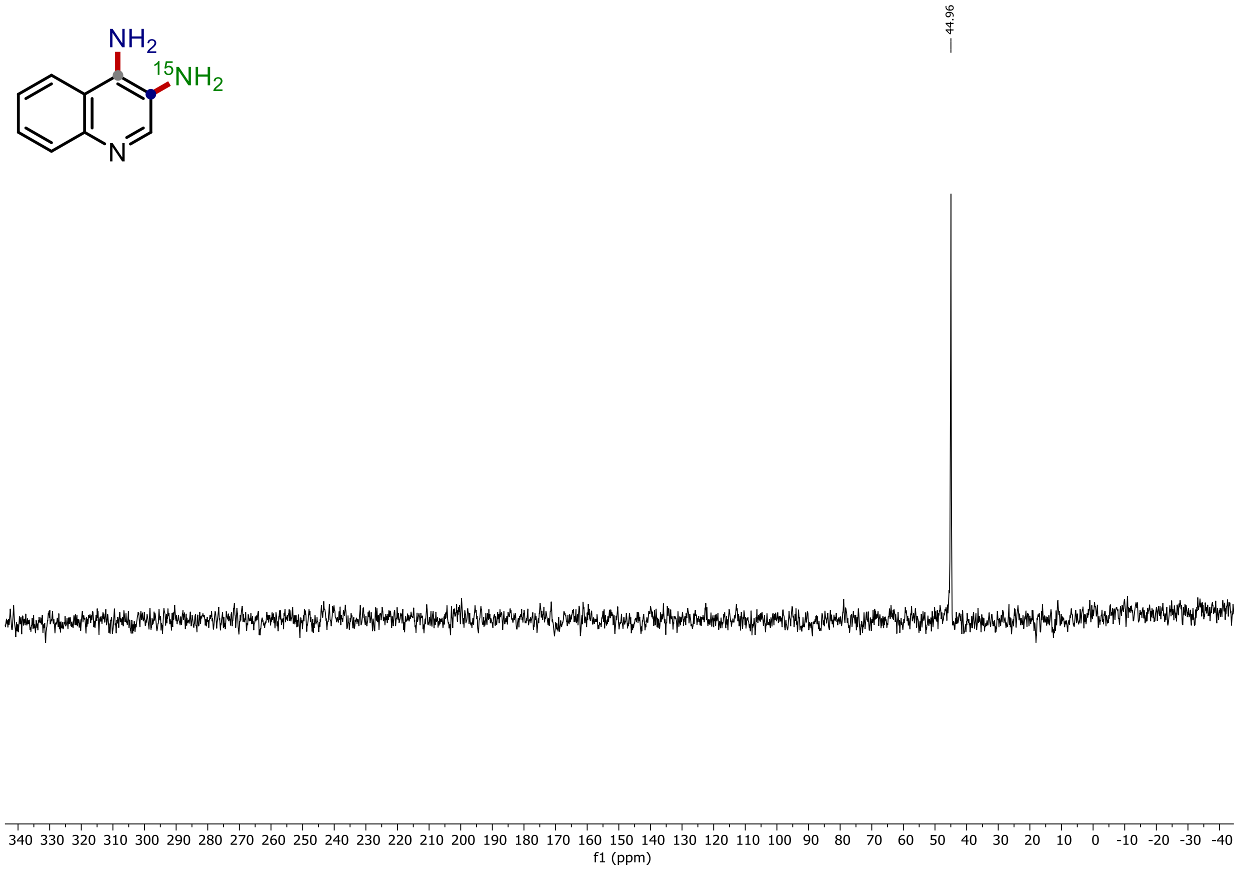

^{15}N-^{1}H HMBC (41 MHz, DMSO-d_6) of 2a*

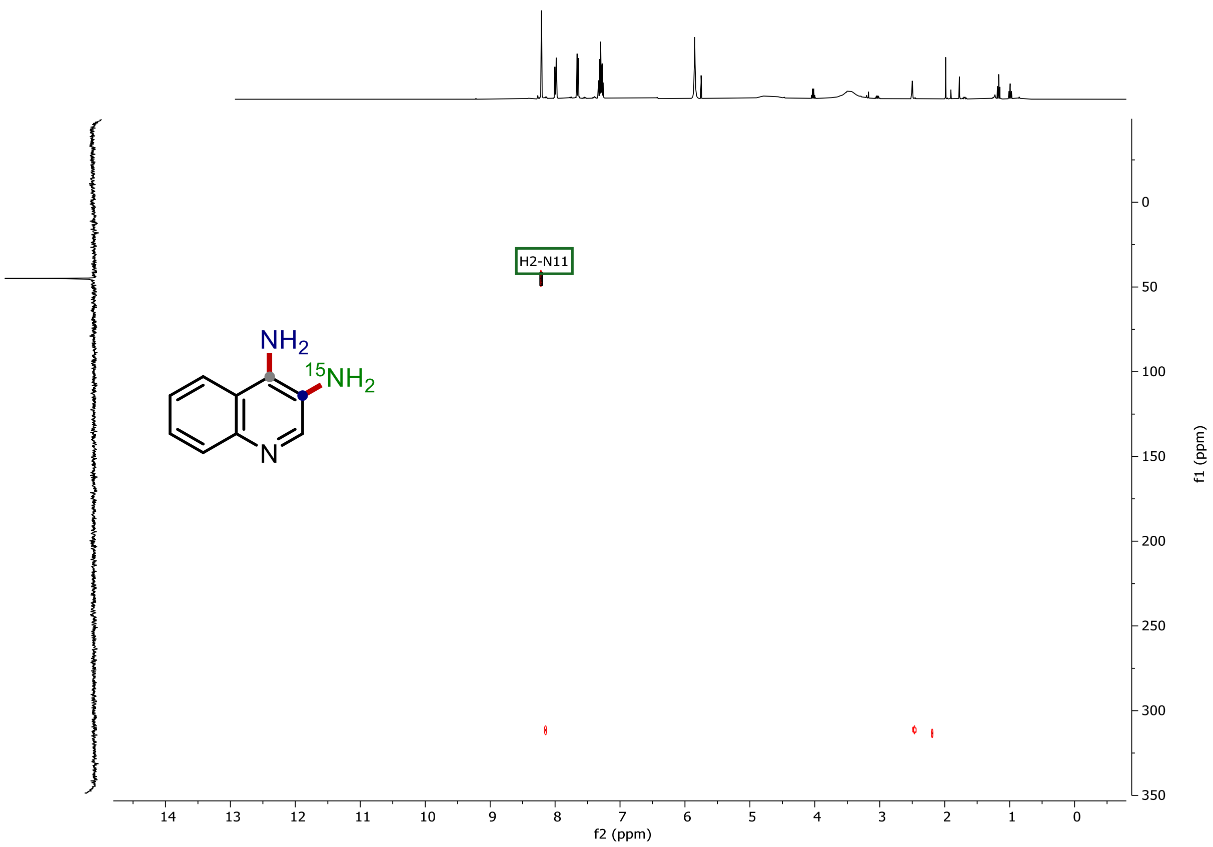

^{1}H NMR (400 MHz, DMSO-d_6) of 37* (see procedure)

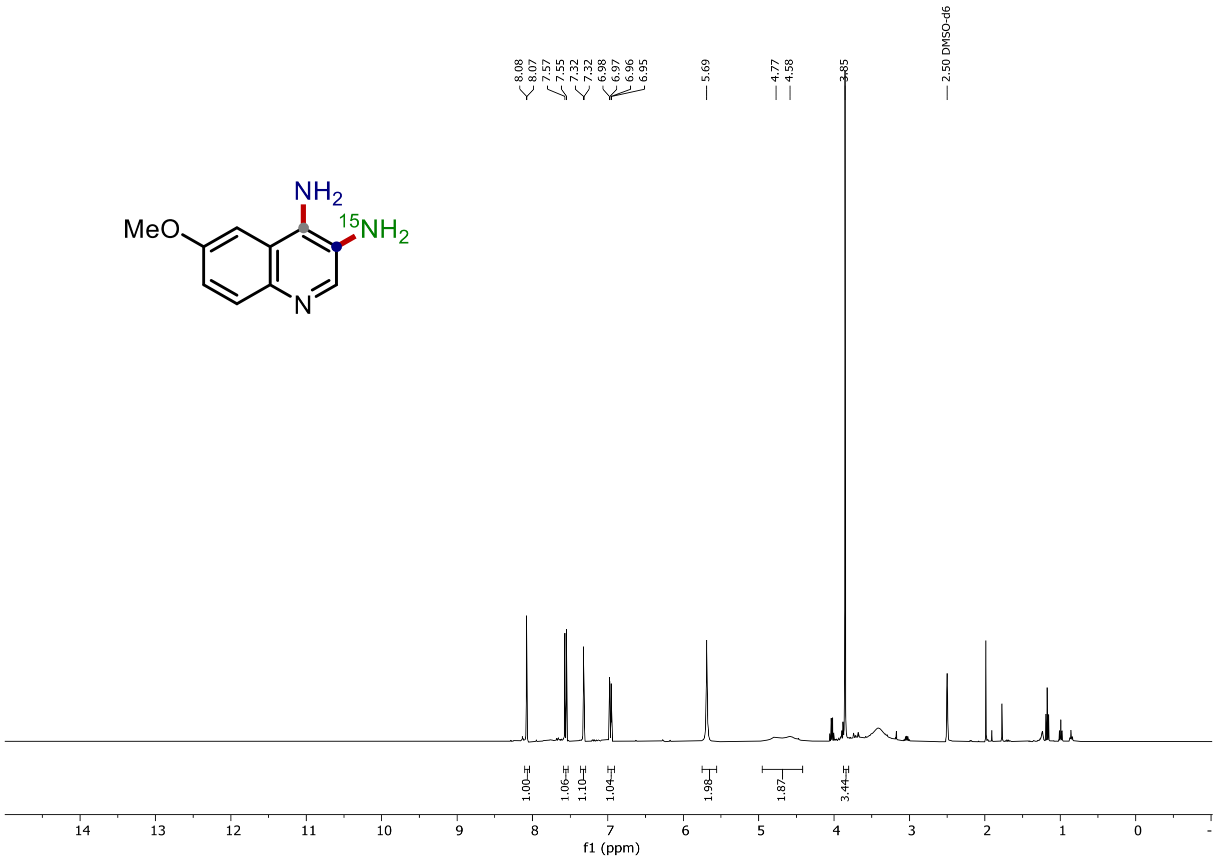

$^{13}C\{^{1}H\}$ NMR (101 MHz, DMSO-d_6) of 37*

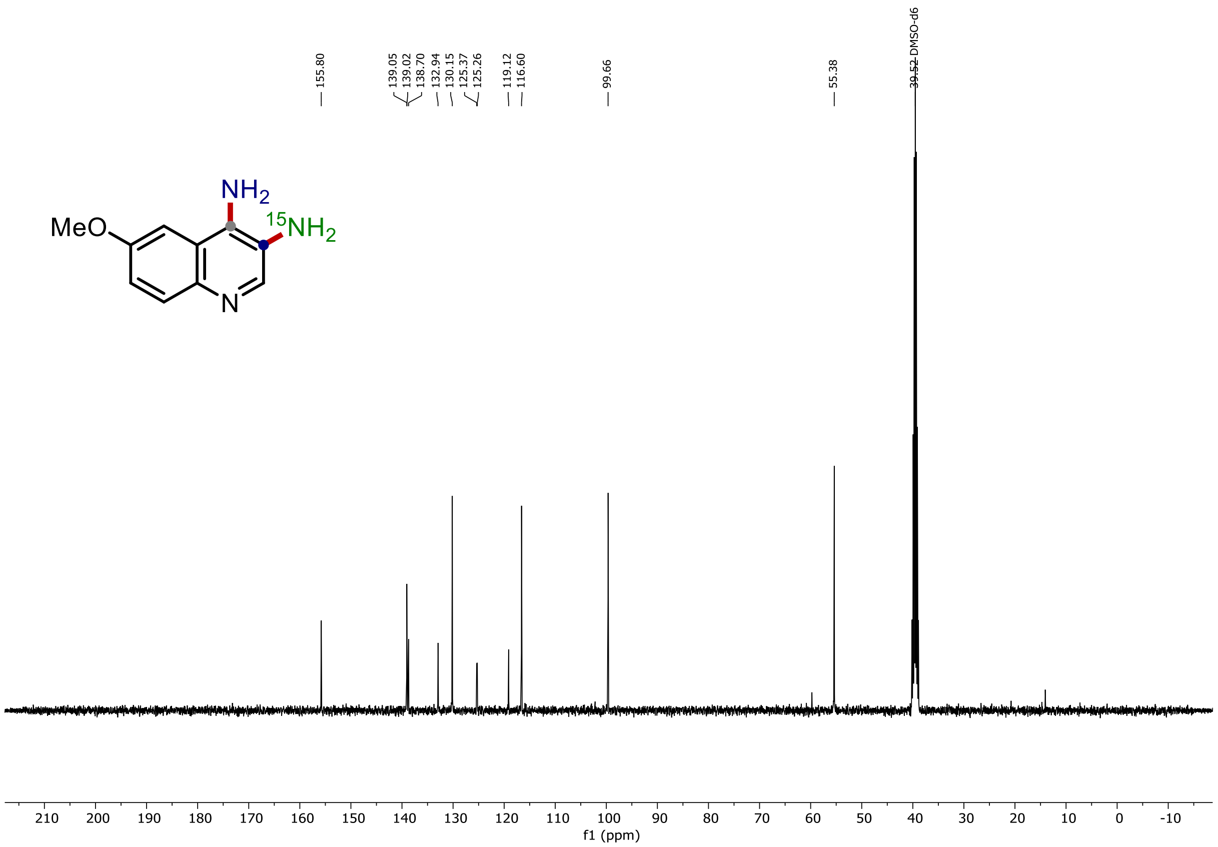

^{15}N NMR (41 MHz, DMSO-*d*6) of 37*

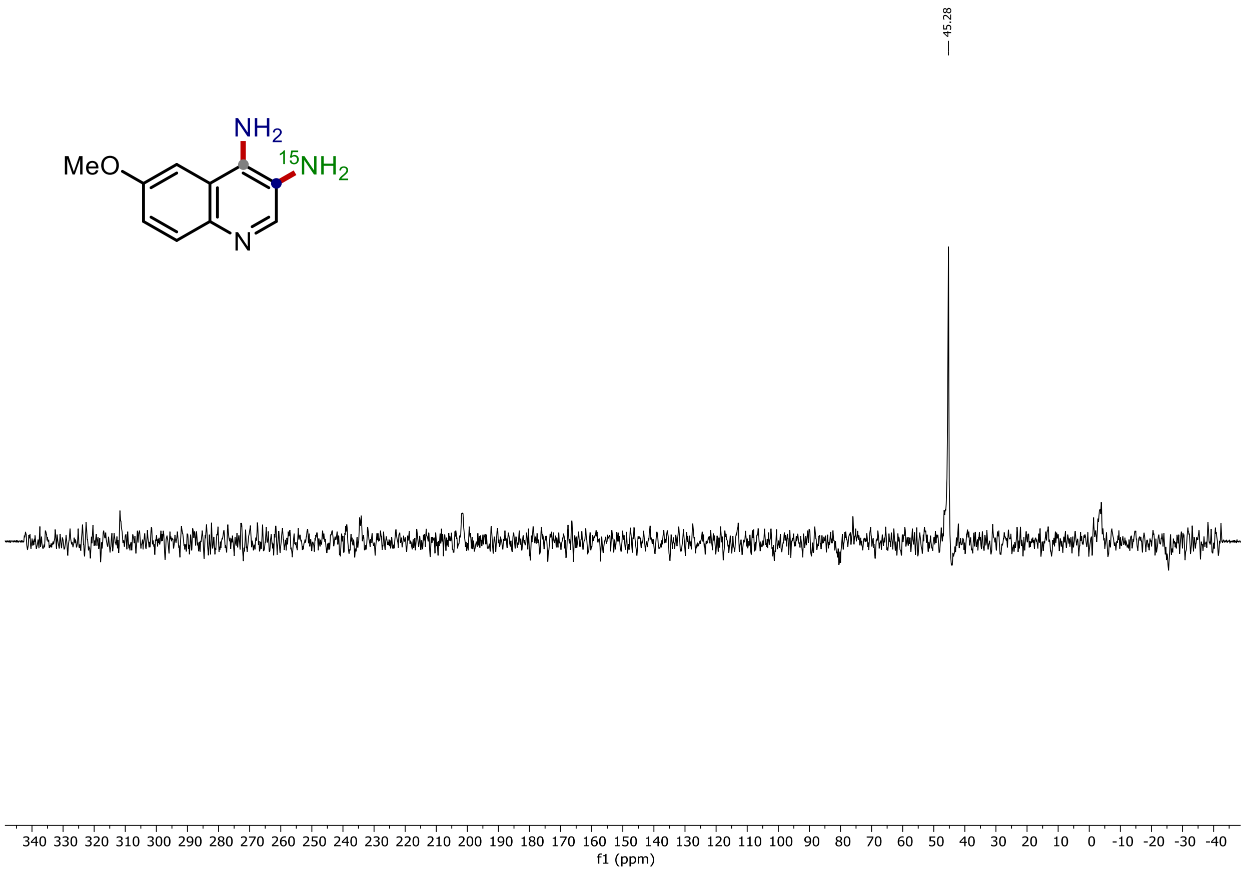

^{15}N-^{1}H HMBC (41 MHz, DMSO-d_6) of 37*

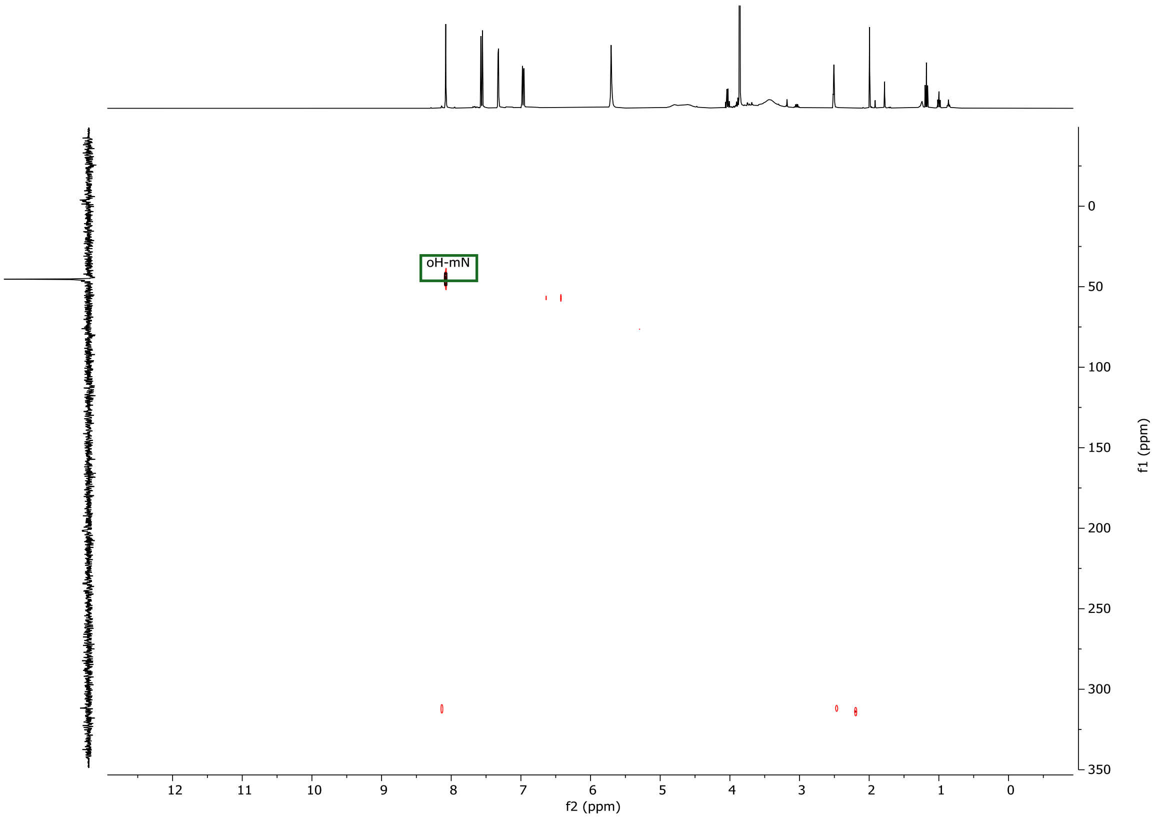

^{1}H NMR (400 MHz, $CDCl_3$) of A-1* (see procedure)

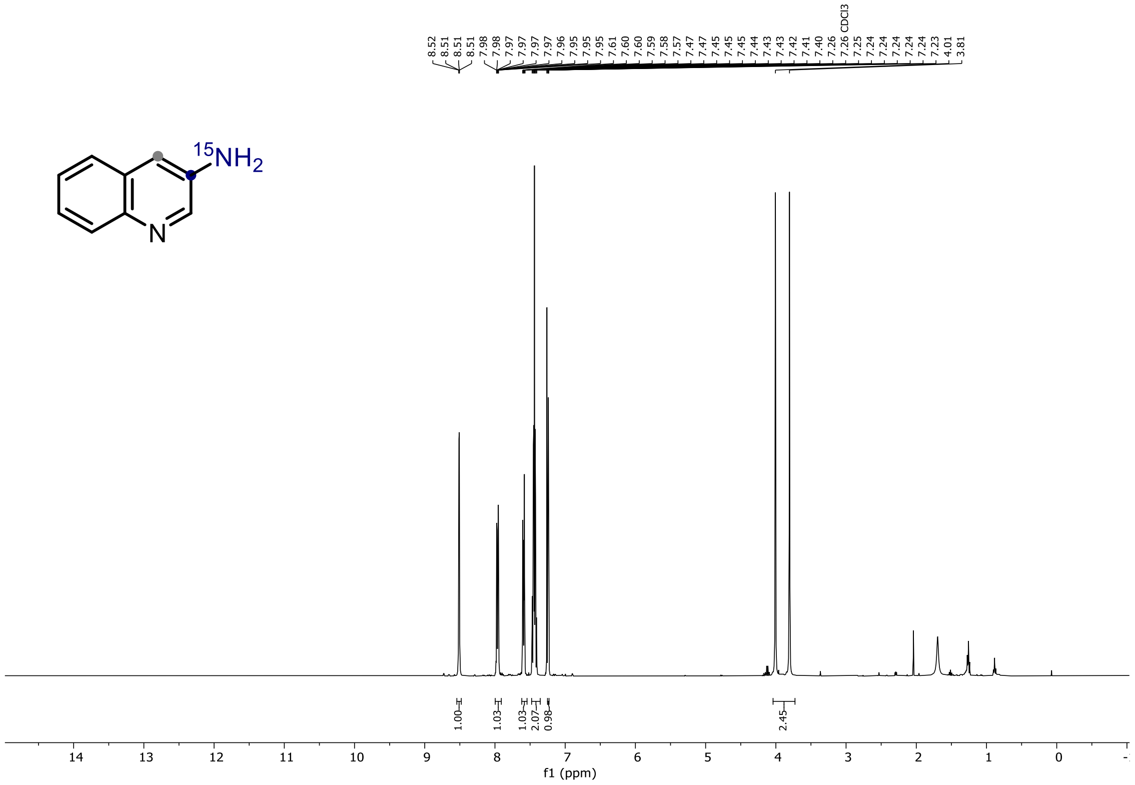

$^{13}C\{^{1}H\}$ NMR (101 MHz, $CDCl_3$) of **A-1***

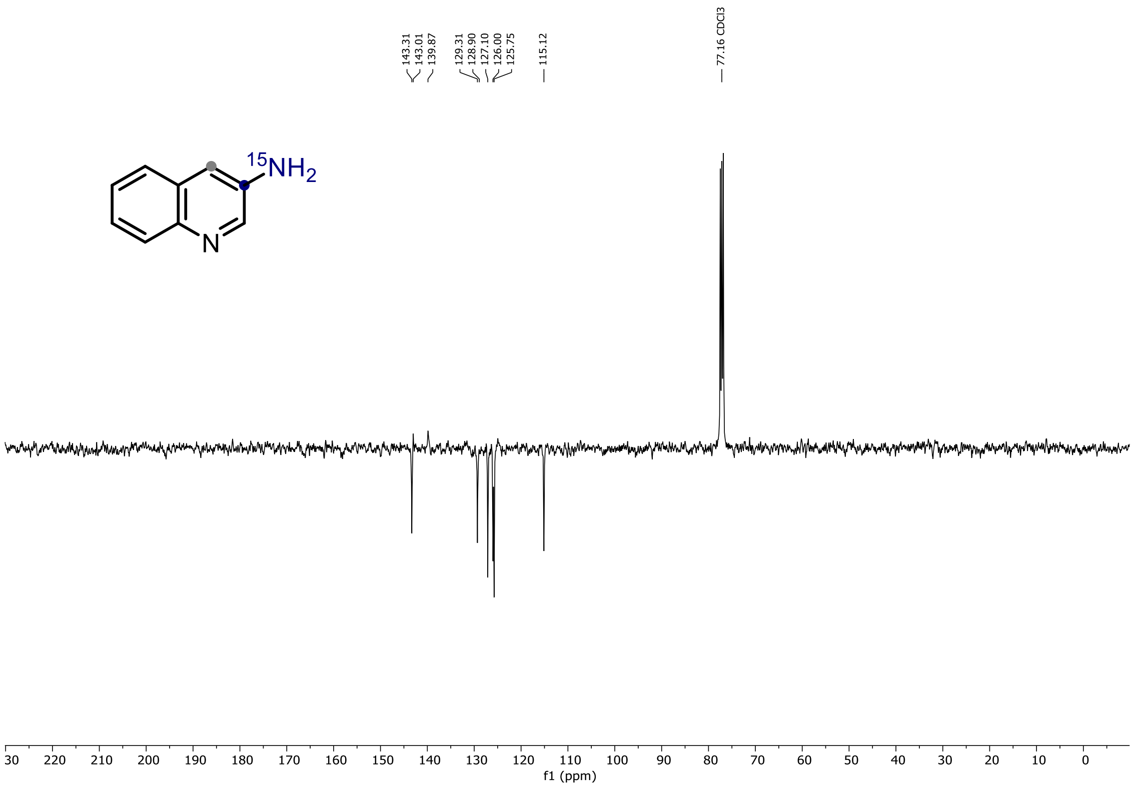

^{15}N NMR (41 MHz, $CDCl_3$) of A-1*

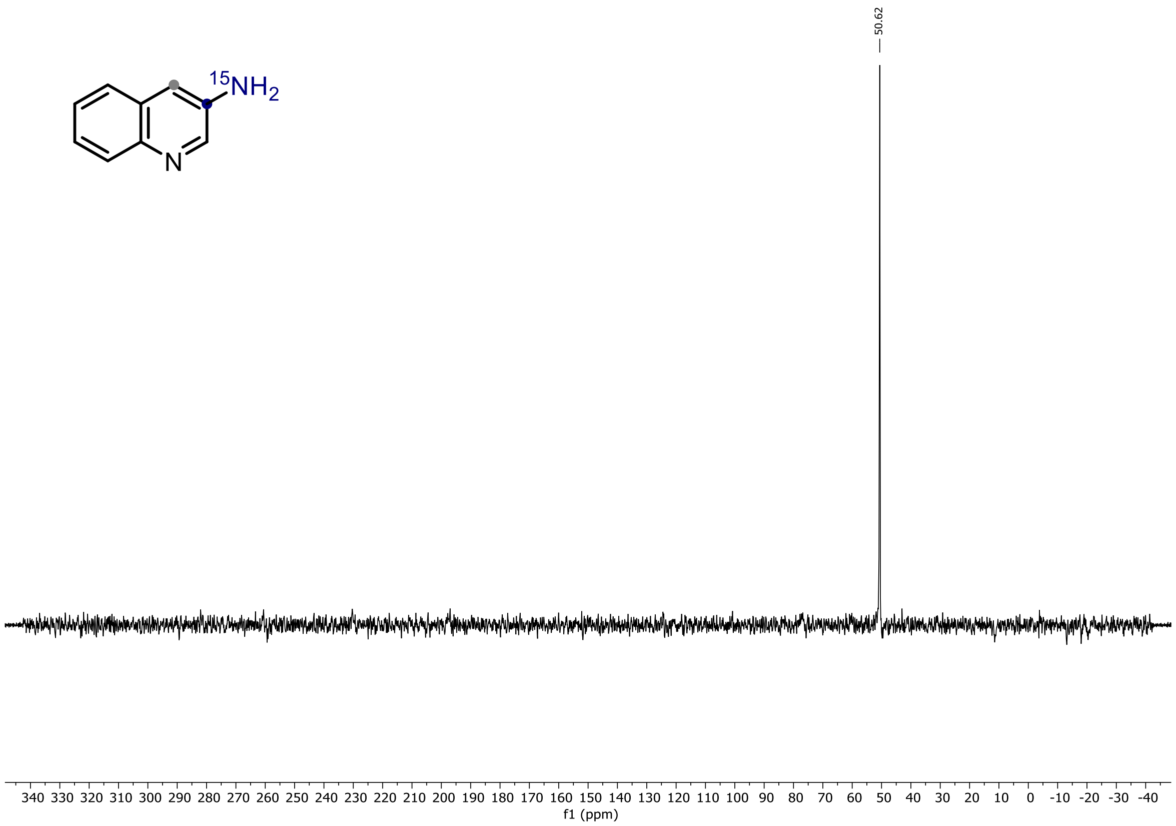

^{1}H NMR (400 MHz, $CDCl_3$) of Az-1* (see procedure)

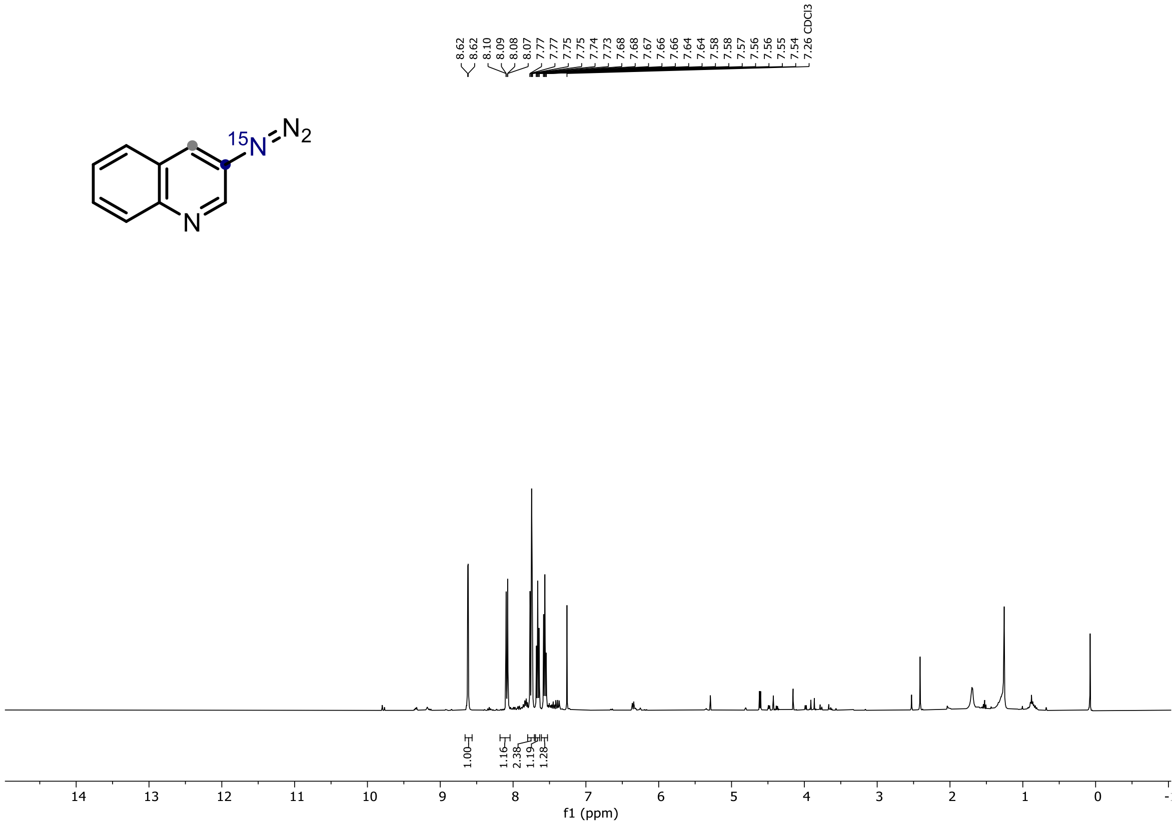

^{13}C{^{1}H} NMR (101 MHz, $CDCl_3$) of Az-1*

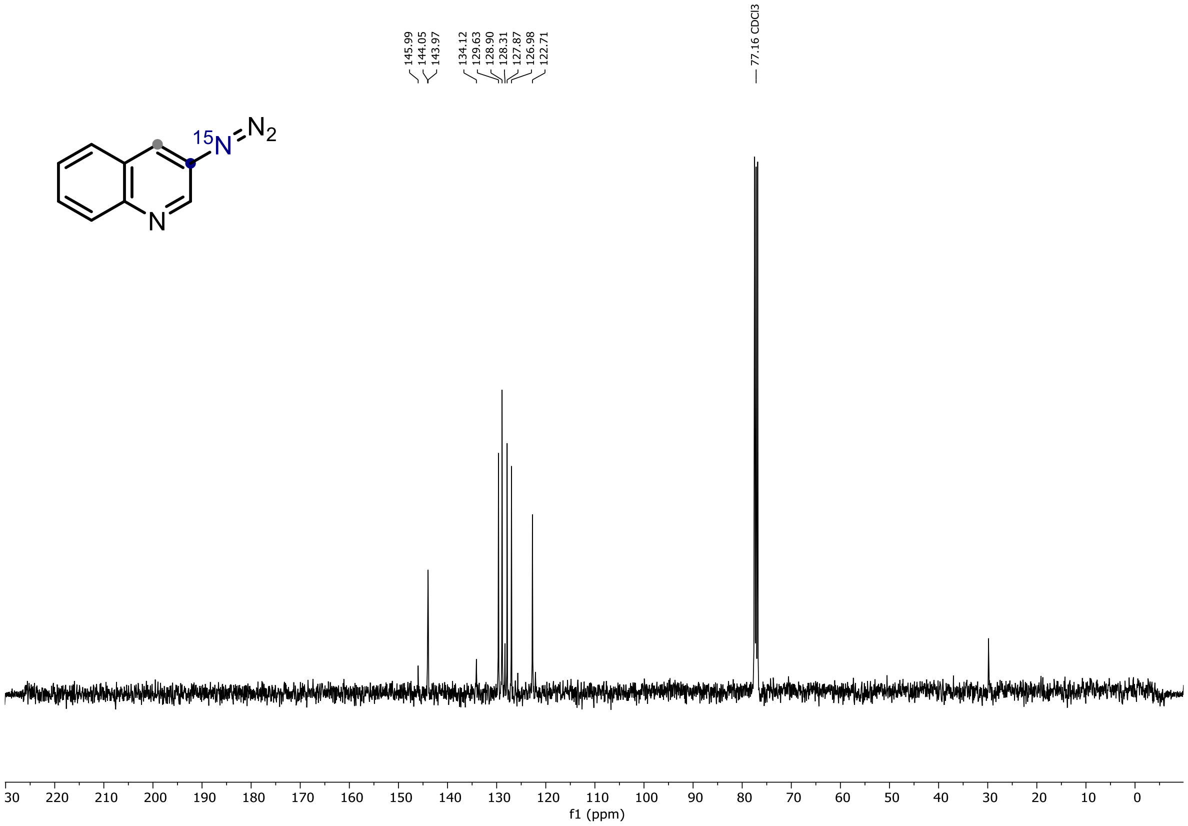

^{15}N NMR (41 MHz, $CDCl_3$) of Az-1*

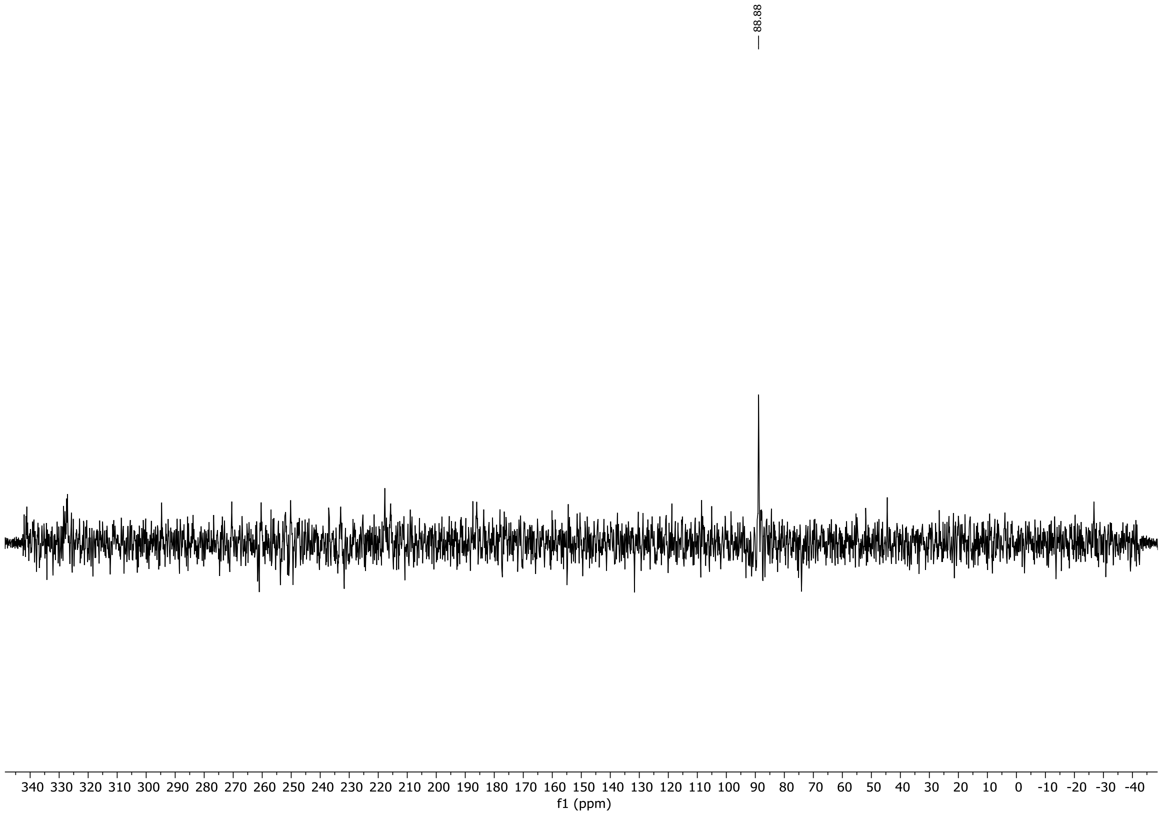

^{1}H NMR (400 MHz, DMSO-*d*$_6$) of 2b* (see procedure)

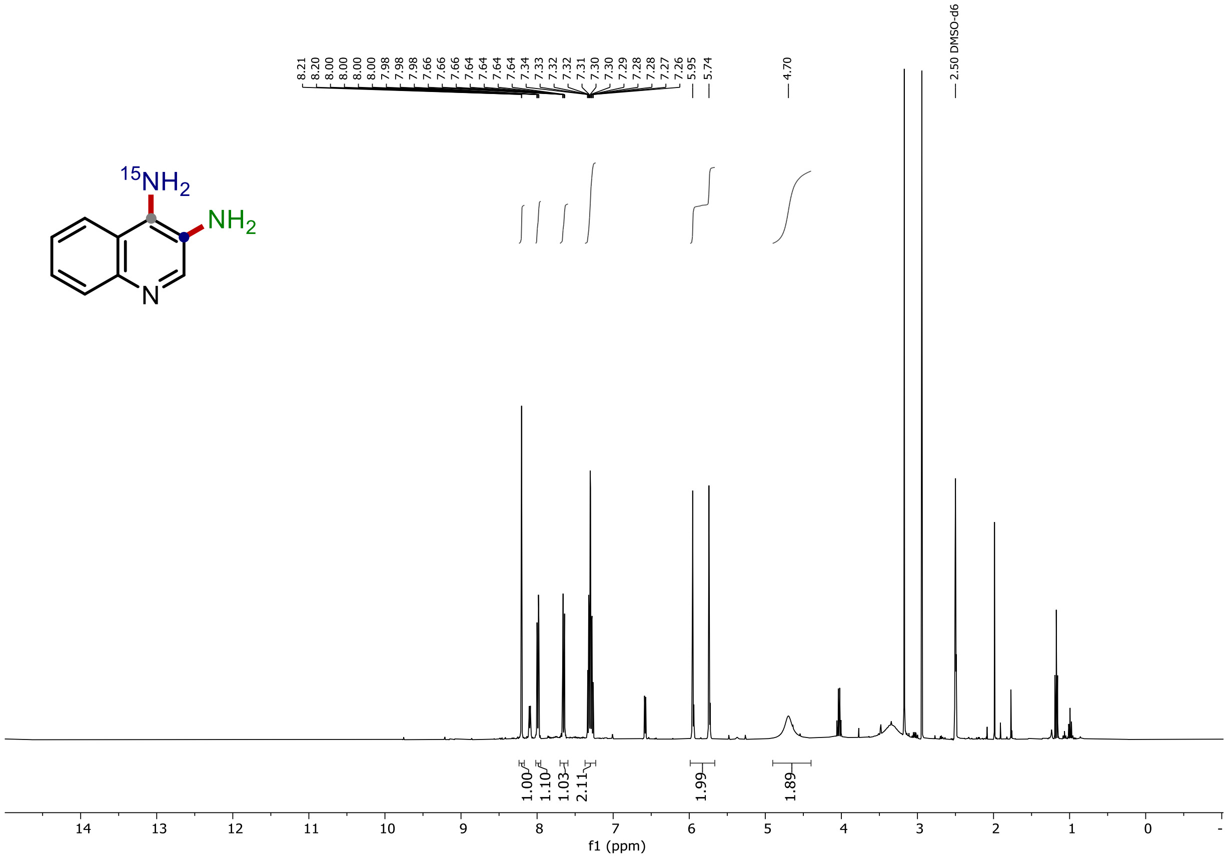

$^{13}C\{^{1}H\}$ NMR (101 MHz, DMSO-d_6) of 2b*

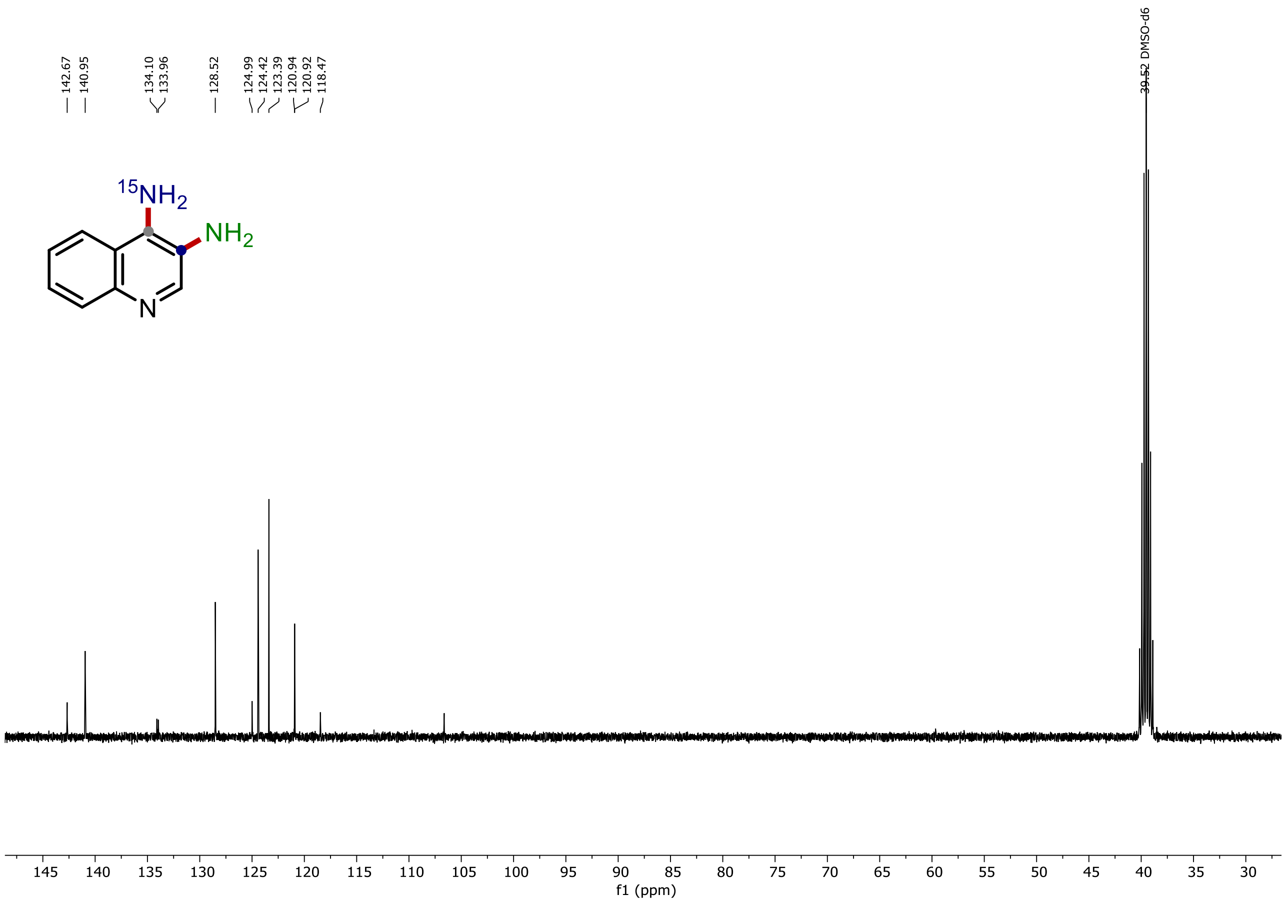

^{15}N NMR (41 MHz, DMSO-d_6) of 2b*

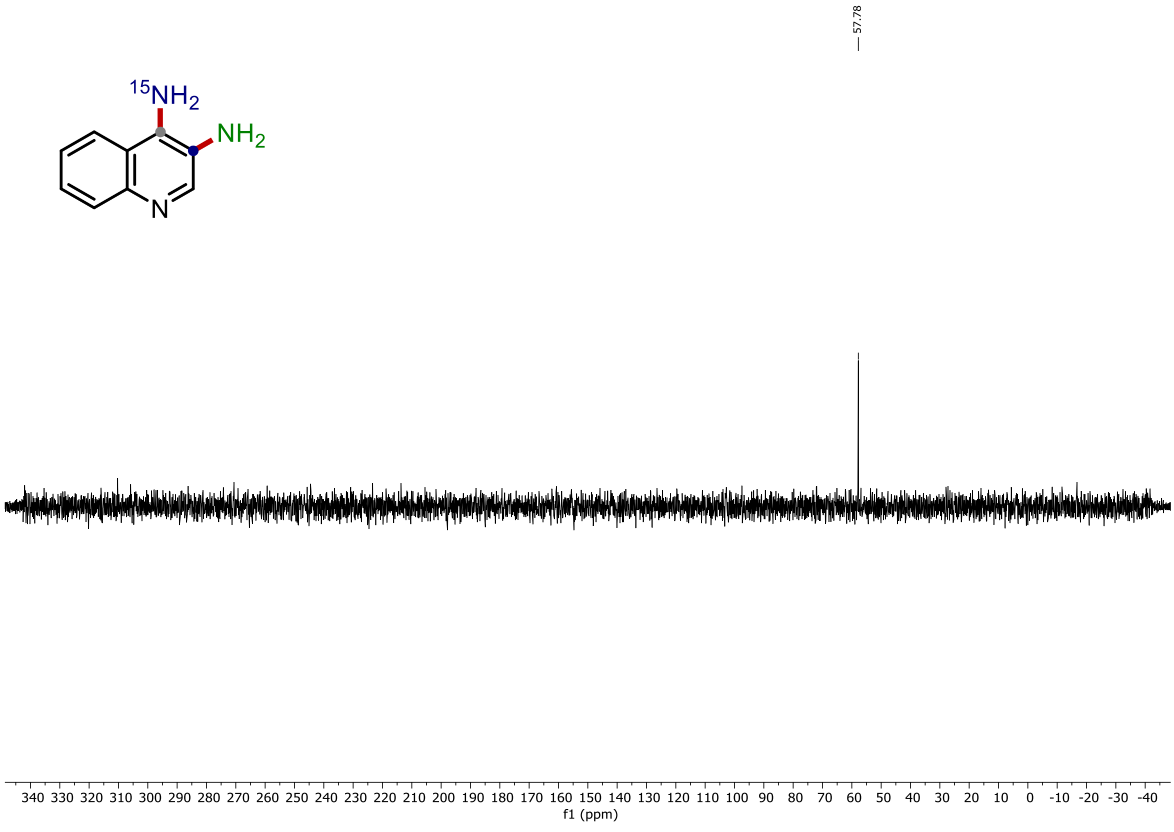

^{1}H NMR (400 MHz, $CDCl_3$) of 38 (see procedure)

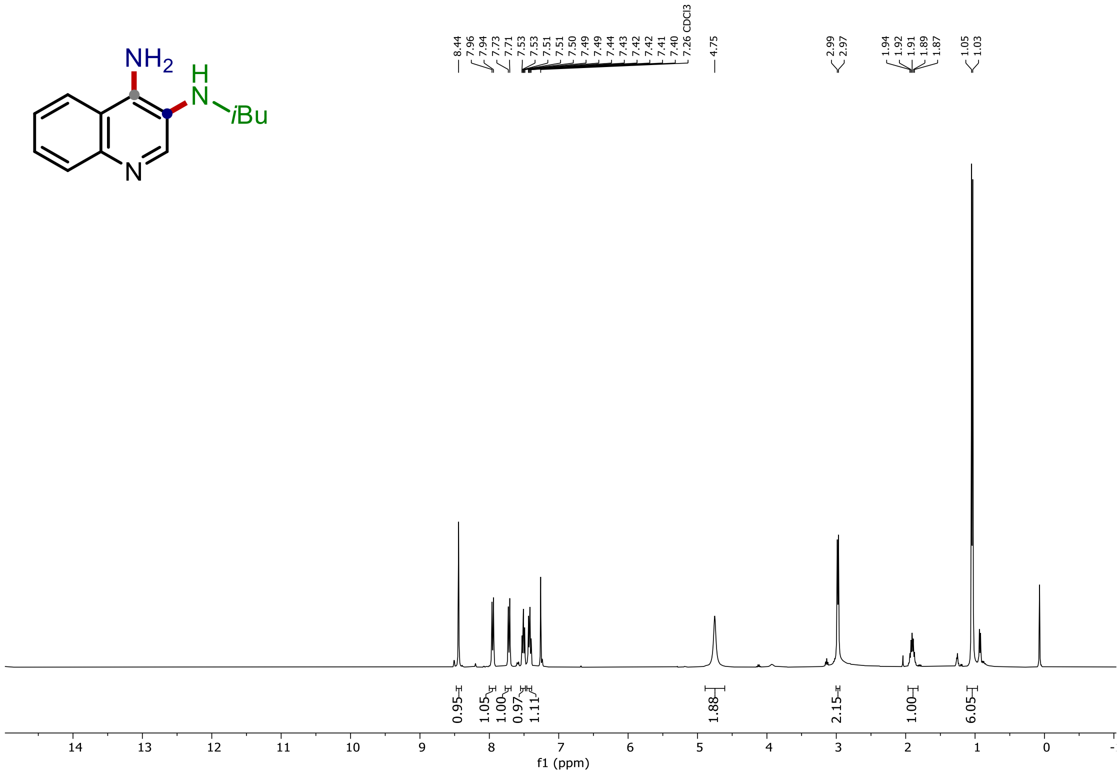

^{13}C{^{1}H} NMR (400 MHz, $CDCl_3$) of 38

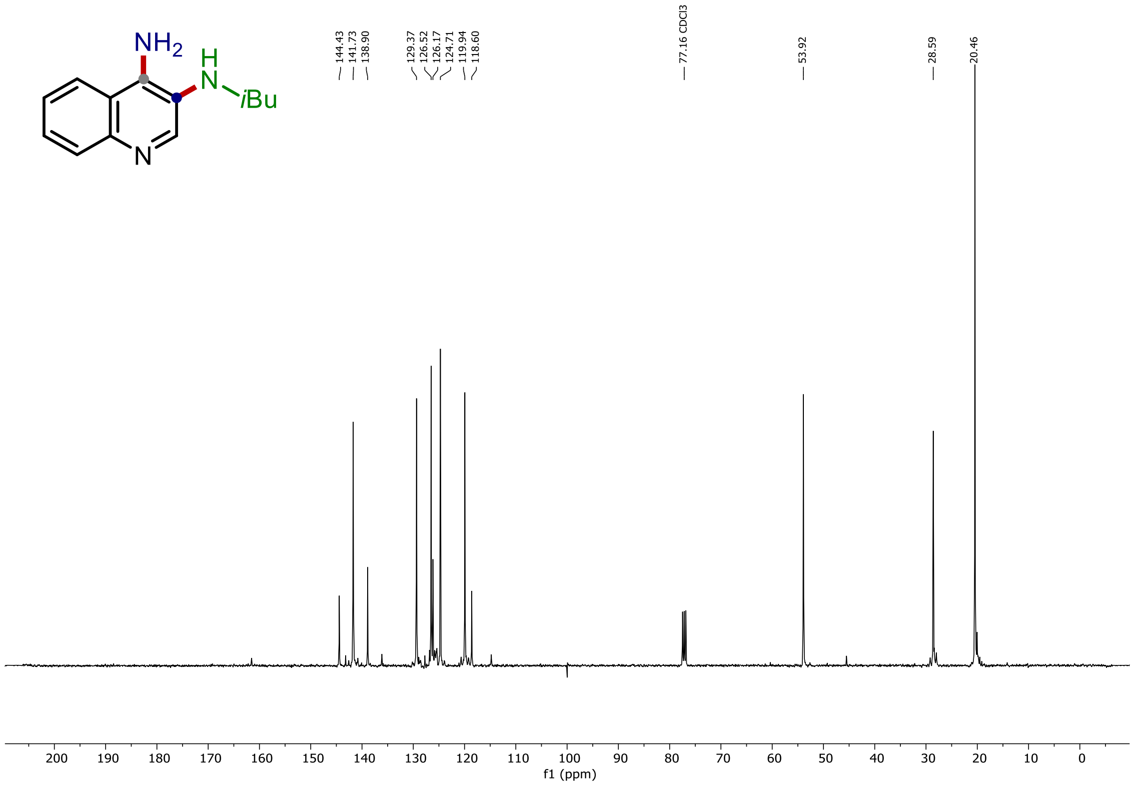

^{1}H NMR (400 MHz, $CDCl_3$) of 3-isobutyl-3*H*-imidazo[4,5-c]quinoline (see procedure)

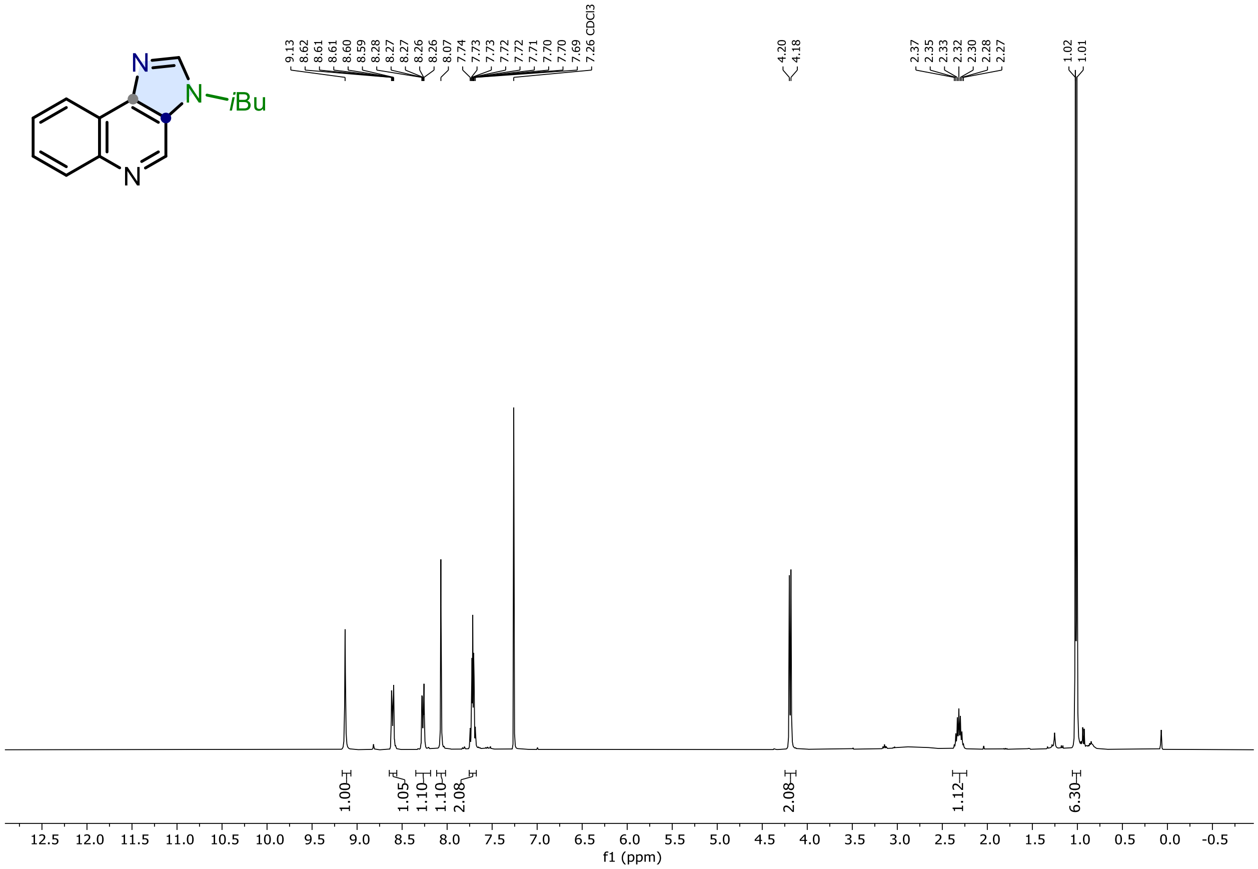

$^{13}C\{^1H\}$ NMR (400 MHz, $CDCl_3$) of 3-isobutyl-3*H*-imidazo[4,5-c]quinoline

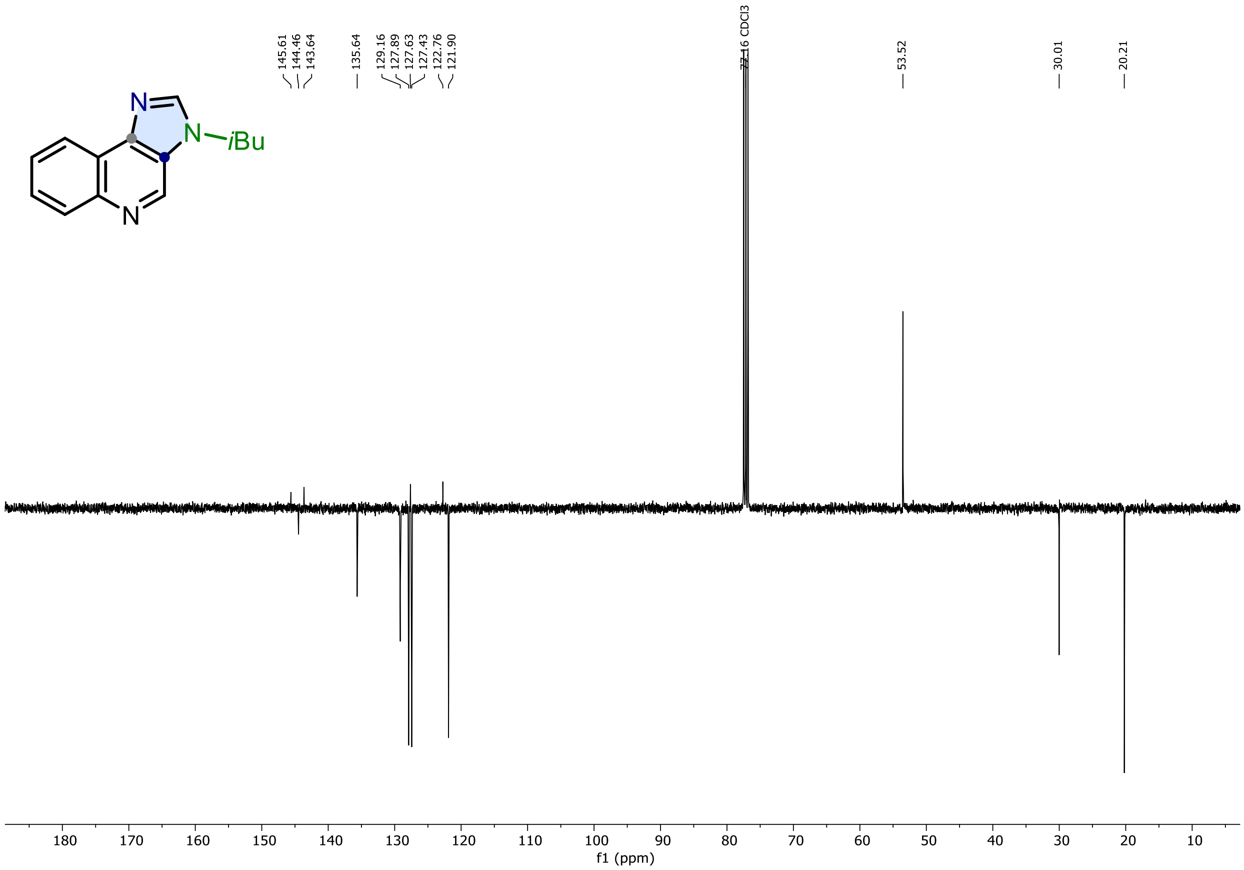

^{1}H NMR (400 MHz, CDCl$_3$) of 3-isobutyl-3*H*-imidazo[4,5-*c*]quinoline 5-oxide (see procedure)

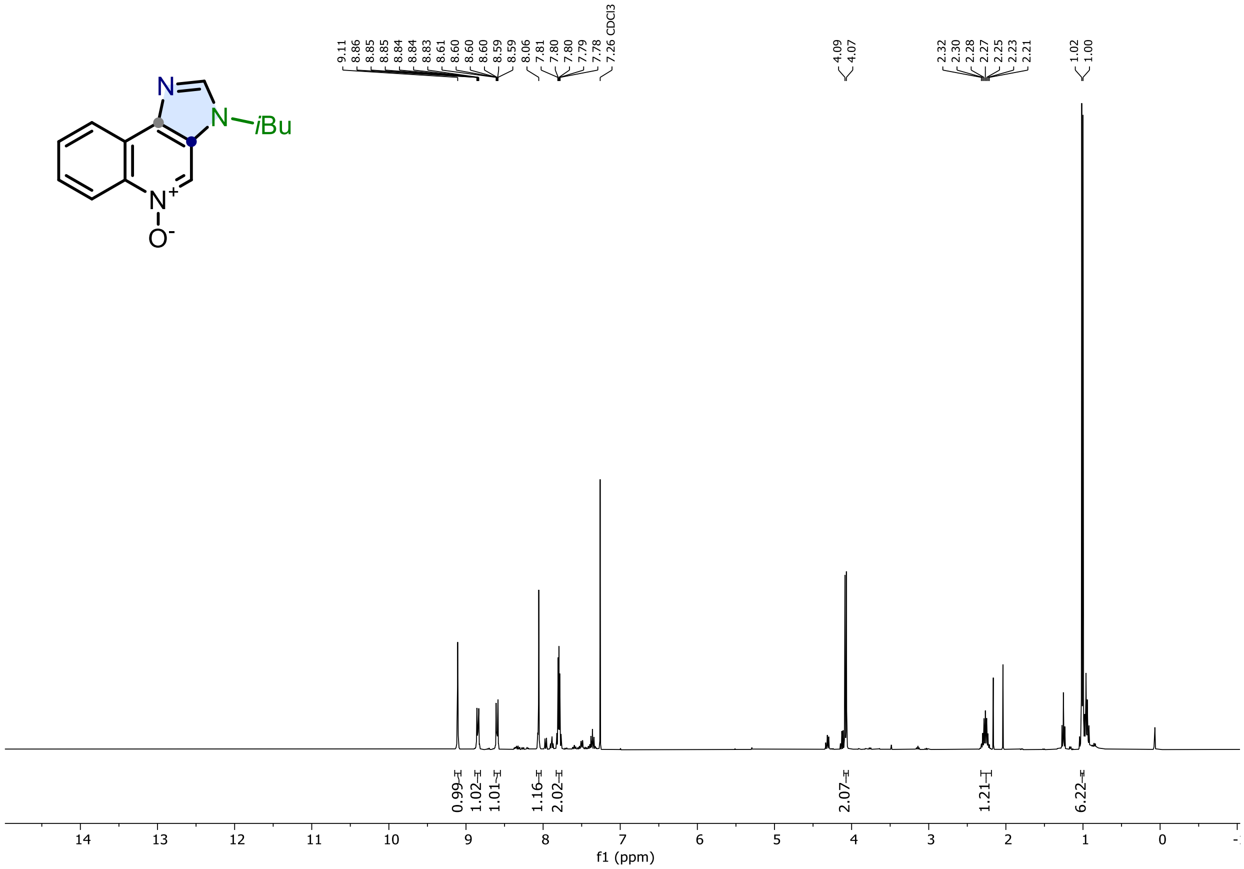

^{1}H NMR (400 MHz, CDCl$_3$) of 39 (see procedure)

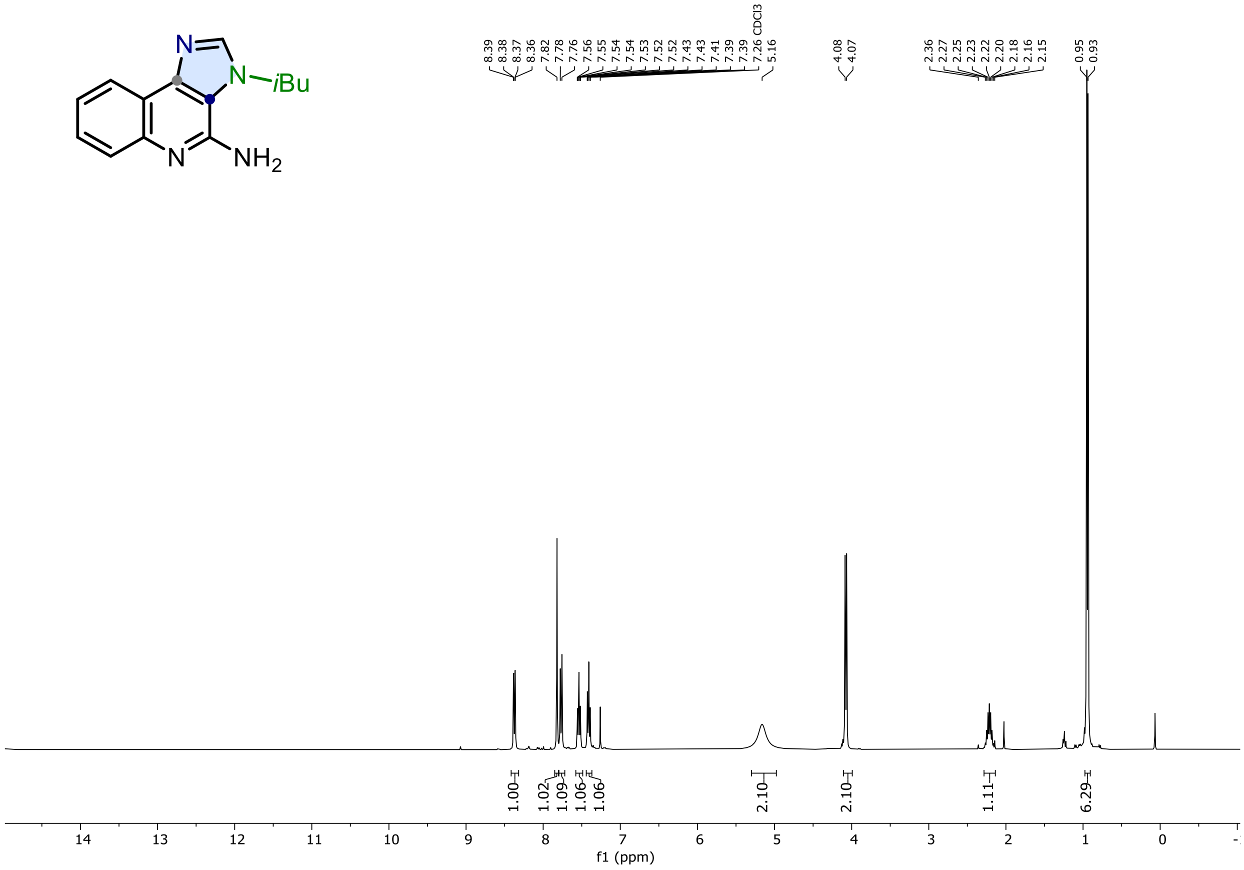

$^{13}C\{^1H\}$ NMR (400 MHz, $CDCl_3$) of 39

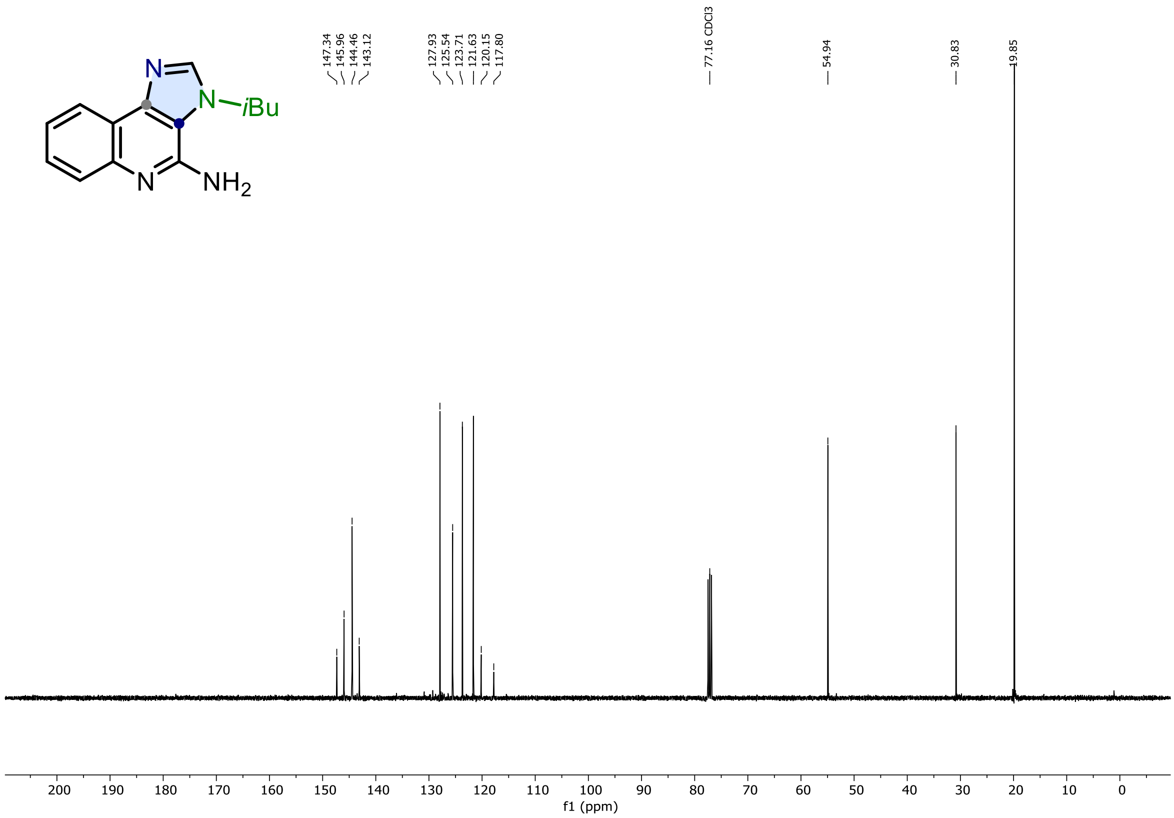

^1H NMR (400 MHz, $CDCl_3$) of 40 (see procedure)

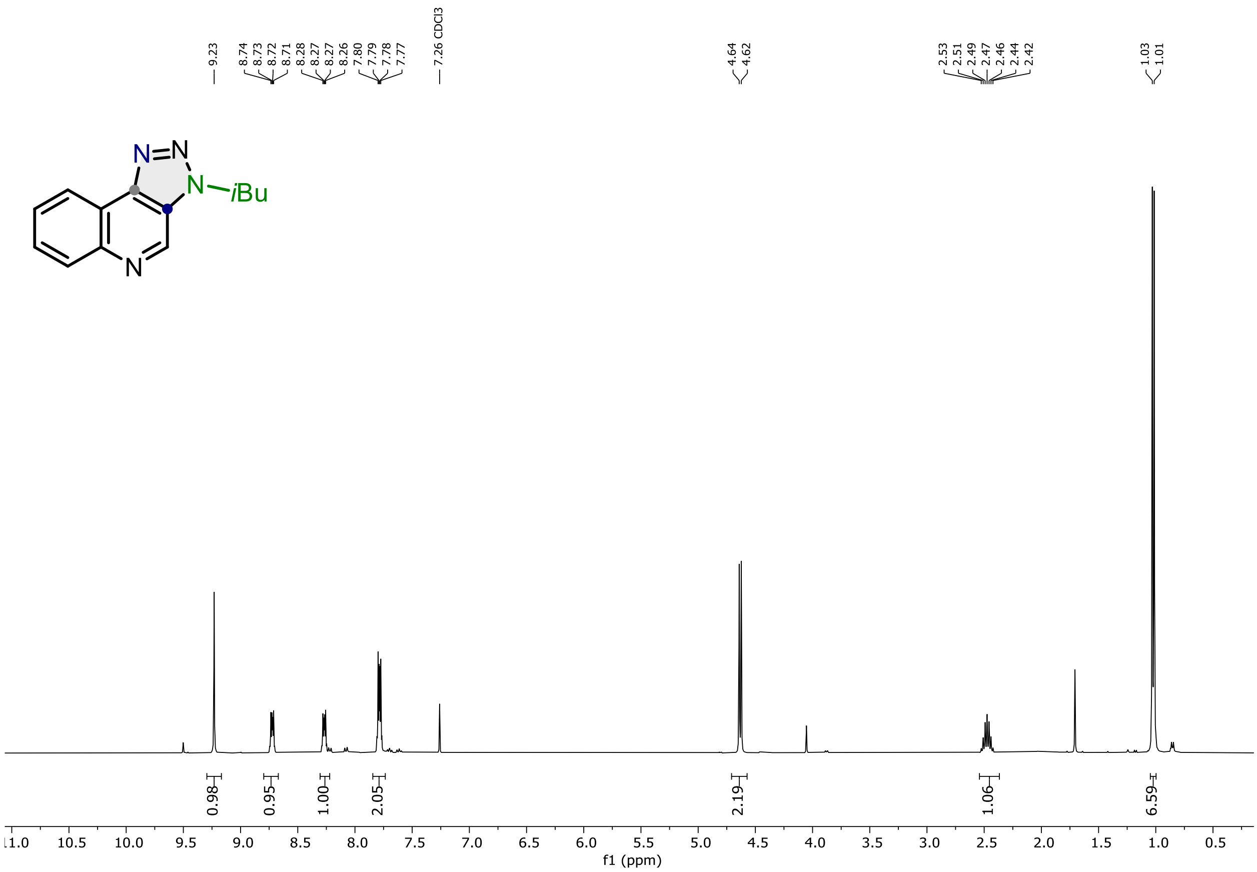

$^{13}C\{^1H\}$ NMR (400 MHz, $CDCl_3$) of 40

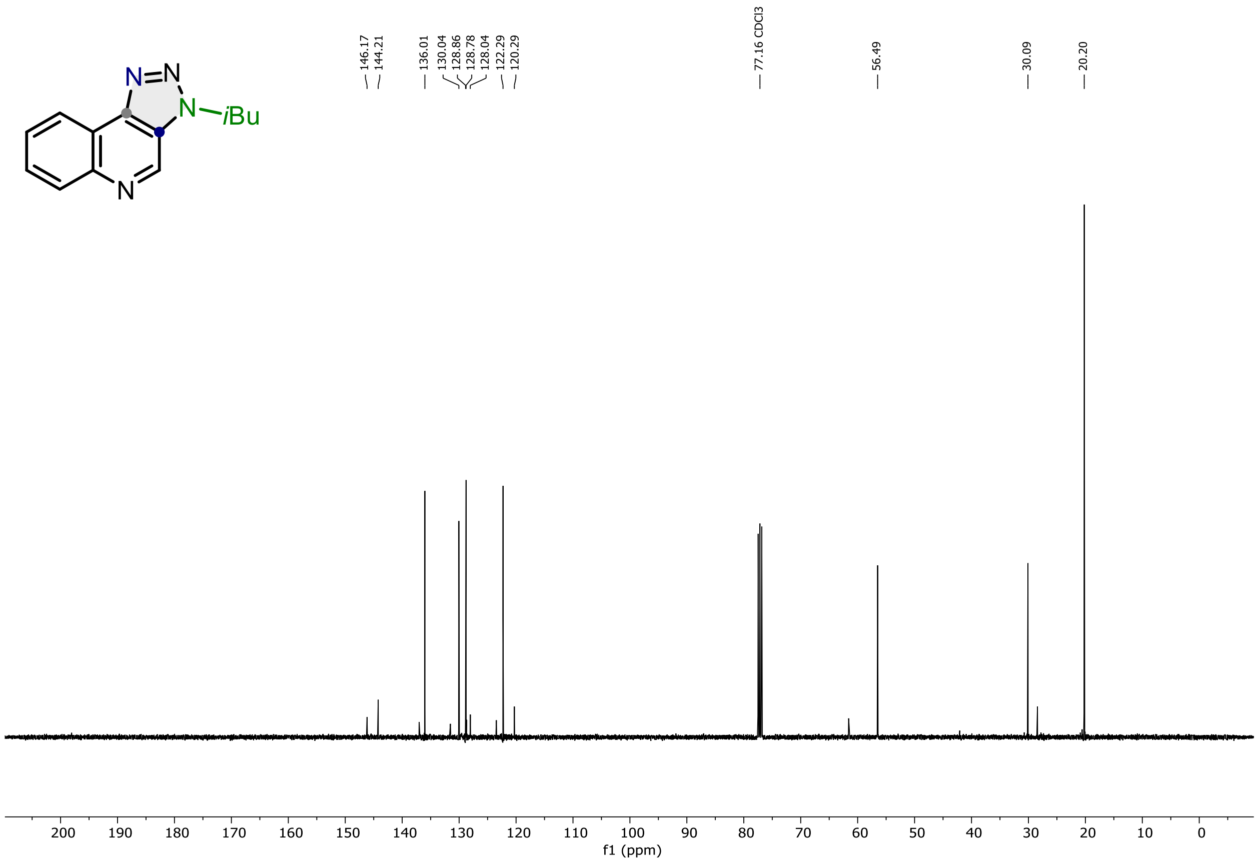

^1H NMR (400 MHz, DMSO-d_6) of 41 (see procedure)

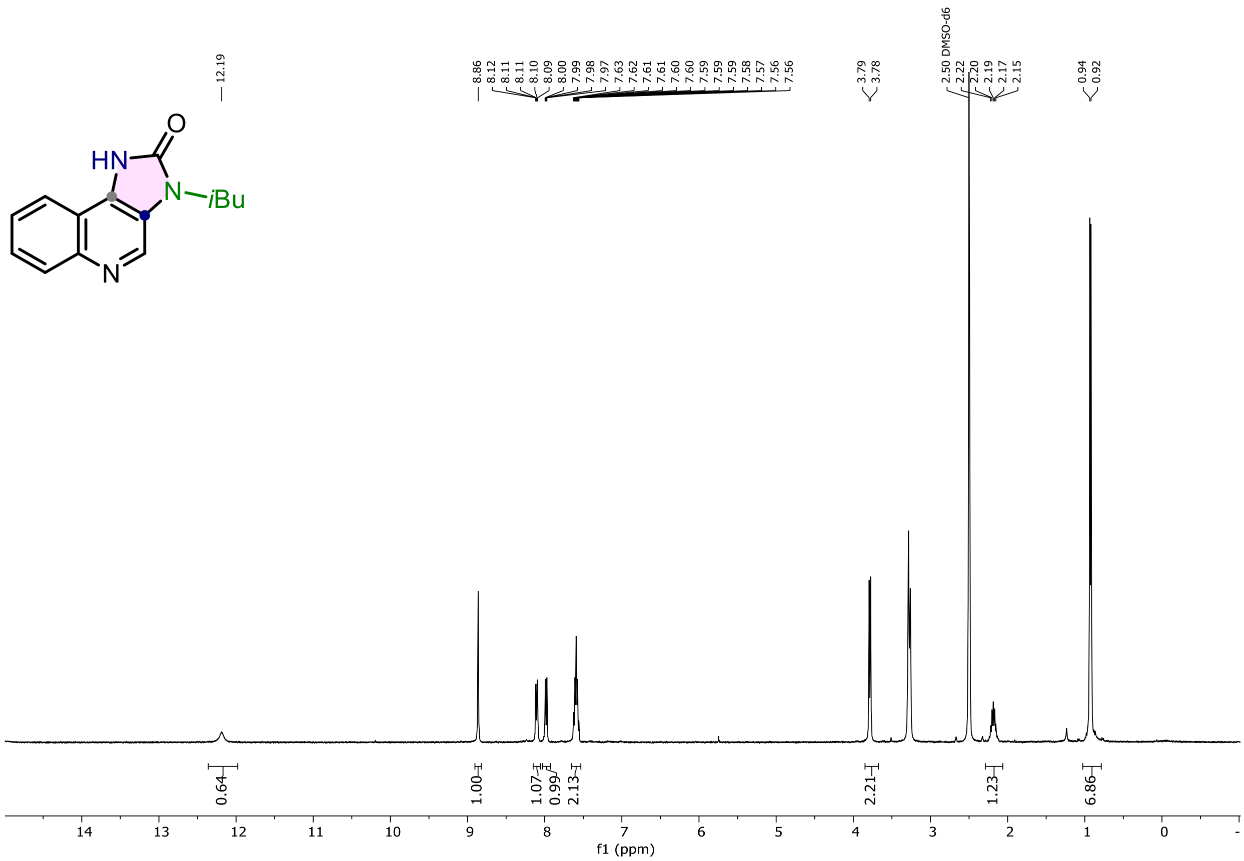

$^{13}C\{^1H\}$ NMR (400 MHz, DMSO-d_6) of 41

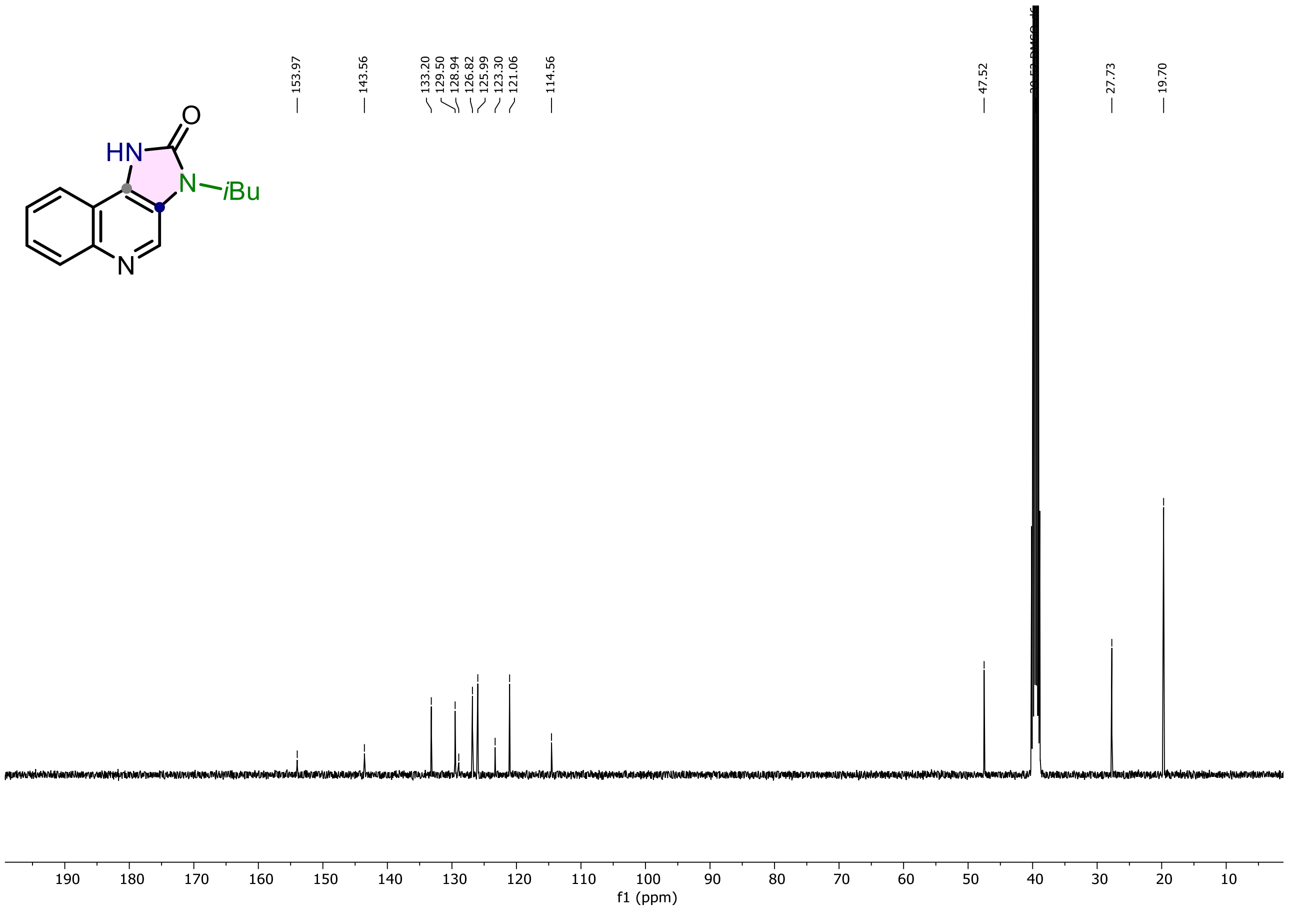

^1H NMR (400 MHz, DMSO-d_6) of 42 (see procedure)

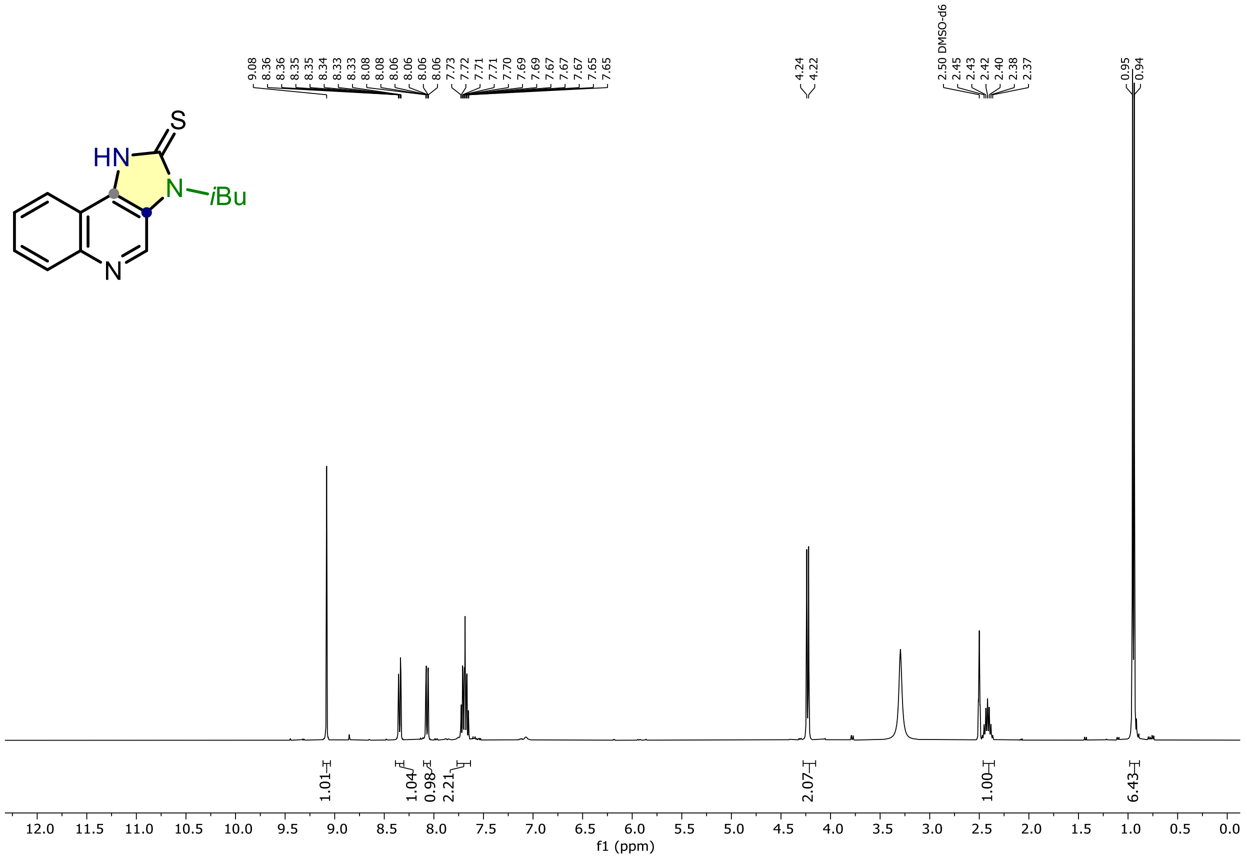

$^{13}C\{^1H\}$ NMR (400 MHz, DMSO-d_6) of 42

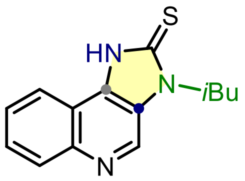

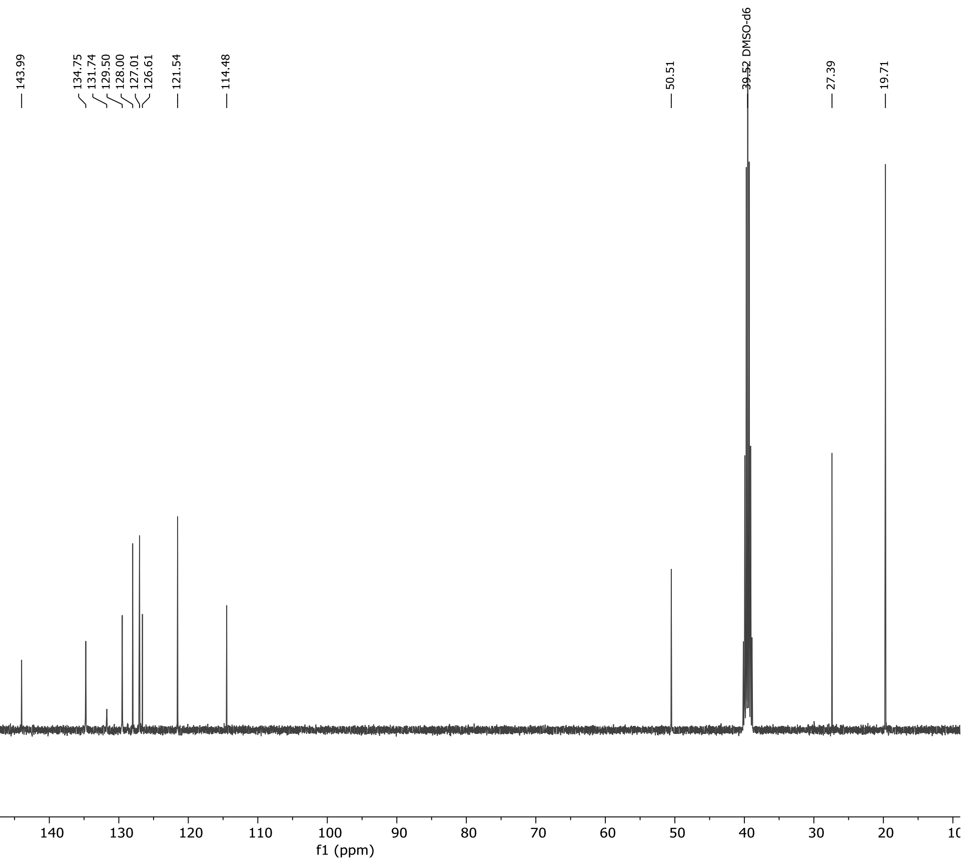

5. REFERENCES

[1] H. Schoenmakers, L. Spiegel, “Laboratory Distillation and Scale-up” *Distillation: Equipment and Processes* **2014**, 319–339.

[2] F. R. Bou-Hamdan, F. Lévesque, A. G. O’Brien, P. H. Seeberger, *Beilstein J. Org. Chem.* **2011**, *7*, 1124–1129.

[3] W. Liu, J. Zhou, T. Zhang, H. Zhu, H. Qian, H. Zhang, W. Huang, R. Gust, *Bioorg. Med. Chem. Lett.* **2012**, *22*, 2701–2704.

[4] J. Xu, J. Ai, S. Liu, X. Peng, L. Yu, M. Geng, F. Nan, *Org. Biomol. Chem.* **2014**, *12*, 3721–3734.

[5] M. M. Mahamudul Hassan, B. Mondal, S. Singh, C. Haldar, J. Chaturvedi, R. Bisht, R. B. Sunoj, B. Chattopadhyay, *J. Org. Chem.* **2022**, *87*, 4360–4375.

[6] C. Morales-Manrique, C. Bolm, D. Gamba-Sánchez, *J. Org. Chem.* **2025**, *90*, 13854–13861.

[7] J. Gao, S. Bhunia, K. Wang, L. Gan, S. Xia, D. Ma, *Org. Lett.* **2017**, *19*, 2809–2812.

[8] N. Terzić, J. Konstantinović, M. Tot, J. Burojević, O. Djurković-Djaković, J. Srbljanović, T. Štajner, T. Verbić, M. Zlatović, M. Machado, I. S. Albuquerque, M. Prudêncio, R. J. Sciotti, S. Pecic, S. D’Alessandro, D. Taramelli, B. A. Šolaja, *J. Med. Chem.* **2015**, *59*, 264–281.

[9] F. Lv, Z. F. Li, W. Hu, X. Wu, “Small molecules enhance functional O-mannosylation of Alpha-dystroglycan” *Bioorg. Med. Chem.* **2015**, *23*, 7661–7670.

[10] S. M. Guo, P. Xu, C. G. Daniliuc, A. Studer, *Nat. Commun.* **2025**, *16*, 9395-.

[11] M. Draskovits, D. Catorci, L. Wimmer, S. Rehman, D. C. B. Siebert, M. Ernst, M. Schnürch, M. D. Mihovilovic, *Monatsh. Chem.* **2022**, *154*, 1391–1404.

[12] E. D. Goddard-Borger, R. V. Stick, *Org. Lett.* **2007**, *9*, 3797–3800.

[13] J. F. Gerster, K. J. Lindstrom, R. L. Miller, M. A. Tomai, W. Birmachu, S. N. Bomersine, S. J. Gibson, L. M. Imbertson, J. R. Jacobson, R. T. Knafla, P. V. Maye, N. Nikolaides, F. Y. Oneyemi, G. J. Parkhurst, S. E. Pecore, M. J. Reiter, L. S. Scribner, T. L. Testerman, N. J. Thompson, T. L. Wagner, C. E. Weeks, J. D. Andre, D. Lagain, Y. Bastard, M. Lupu, “*J. Med. Chem.* **2005**, *48*, 3481–3491.

[14] J. J. Jackson, A. C. Siegmund, W. J. Bai, A. B. Reed, A. B. Birkholz, I. D. G. Campuzano, A. Créquer-Grandhomme, R. Hu, R. V. Modak, A. Sudom, N. Javier, C. Sanders, M. C. Lo, F. Xie, V. J. Cee, P. Manzanillo, J. G. Allen, *J. Med. Chem.* **2023**, *66*, 16120–16140.

[15] G. Lenardon, X. Yzeiri, G. Le Berre, D. B. Yildiz, D. Leonori, A. Ruffoni, *Chem. Sci*. **2025**, *16*, 21416–21422.

[16] M. J. Frisch, G. W. Trucks, H. B. Schlegel, G. E. Scuseria, M. A. Robb, J. R. Cheeseman, G. Scalmani, V. Barone, G. A. Petersson, H. Nakatsuji, X. Li, M. Caricato, A. V. Marenich, J. Bloino, B. G. Janesko, R. Gomperts, B. Mennucci, H. P. Hratchian, J. V. Ortiz, A. F. Izmaylov, J. L. Sonnenberg, F. Ding, F. Lipparini, F. Egidi, J. Goings, B. Peng, A. Petrone, T. Henderson, D. Ranasinghe, V. G. Zakrzewski, J. Gao, N. Rega, G. Zheng, W. Liang, M. Hada, M. Ehara, K. Toyota, R. Fukuda, J. Hasegawa, M. Ishida, T. Nakajima, Y. Honda, O. Kitao, H. Nakai, T. Vreven, K. Throssell, J. A. Montgomery Jr., J. E. Peralta, F. Ogliaro, M. J. Bearpark, J. J. Heyd, E. N. Brothers, K. N. Kudin, V. N. Staroverov, T. A. Keith, R. Kobayashi, J. Normand, K. Raghavachari, A. P. Rendell, J. C. Burant, S. S. Iyengar, J. Tomasi, M. Cossi, J. M. Millam, M. Klene, C. Adamo, R. Cammi, J. W. Ochterski, R. L. Martin, K. Morokuma, O. Farkas, J. B. Foresman, D. J. Fox, Gaussian 16, Revision C.01, Gaussian, Inc., Wallingford CT, 2016.

[17] Y. Zhao, D. G. Truhlar, *Theor. Chem. Acc*. **2008**, *120*, 215–241.

[18] F. Weigend, R. Ahlrichs, *Phys. Chem. Chem. Phys.* **2005**, *7*, 3297–3305.

[19] G. Luchini, J. V. Alegre-Requena, I. Funes-Ardoiz, R. S. Paton, *F1000Research* **2020**, *9*, 291.
